# Meal Timing and Anthropometric and Metabolic Outcomes: A Systematic Review and Meta-Analysis

**DOI:** 10.1001/jamanetworkopen.2024.42163

**Published:** 2024-11-01

**Authors:** Hiu Yee Liu, Ashley A. Eso, Nathan Cook, Hayley M. O’Neill, Loai Albarqouni

**Affiliations:** 1 Faculty of Health Sciences and Medicine, Bond University, Robina, Australia; 2 Institute for Evidence-Based Healthcare, Bond University, Robina, Australia

## Abstract

**Importance:**

Meal timing strategies, such as time-restricted eating (TRE), reducing meal frequency, or altering calorie distribution across the day, have gained interest for their potential to enhance weight loss and metabolic health, particularly in managing chronic diseases, yet their long-term benefits are not known.

**Objective:**

To evaluate the association between meal timing strategies (≥12 weeks) and anthropometric and metabolic indicators.

**Data Sources:**

Medline, Embase, CINAHL, and Cochrane CENTRAL were searched from inception to October 17, 2023.

**Study Selection:**

Randomized clinical trials, regardless of language and publication date, involving adults 18 years and older, evaluating within-day meal timing patterns for 12 or more weeks, and reporting anthropometric measures were included. Studies were excluded if participants had eating disorders, prior significant weight change, underwent bariatric surgery, were pregnant, or if controlled variables differed between groups.

**Data Extraction and Synthesis:**

Study quality was determined via Risk of Bias 2.0 tool. Data were extracted independently by multiple reviewers. Preferred Reporting Items for Systematic Reviews and Meta-analyses guidelines were used. Meta-analysis was performed using random-effects model on pooled continuous outcomes with 2 or more studies.

**Main Outcome and Measures:**

Weight change in kilograms, reported as between-group mean difference with 95% CIs.

**Results:**

Sixty-nine reports of 29 randomized clinical trials including 2485 individuals (1703 [69%] female; mean [SD] age, 44 [9.5] years; and mean [SD] body mass index, 33 [3.5]) were included. Study interventions included TRE (17 studies), meal frequency (8 studies), and calorie distribution (4 studies). There were some concerns of risk of bias for 7 studies and high concerns for 22 studies. Statistically significant weight change was observed in TRE when compared with control (–1.37 kg; 95% CI, –1.99 to –0.75 kg). Lower meal frequency and earlier caloric distribution were also both associated with greater change (–1.85 kg; 95% CI, –3.55 to –0.13 kg; and –1.75 kg; 95% CI, –2.37 to –1.13 kg, respectively).

**Conclusions and Relevance:**

The findings of this meta-analysis suggest that TRE, lower meal frequency, and earlier caloric distribution in the day may reduce weight compared with standard care and/or nutritional advice; however, the effect sizes found were small and of uncertain clinical importance. High heterogeneity and risk of bias among included studies led to concerns about the certainty of the underpinning evidence. Further research, including trials with larger sample sizes, standardized interventions with prescribed or matched energy intake, and longer follow-up, are needed.

## Key Points

**Question:**

What is the association between meal timing strategies and anthropometric and metabolic indicators?

**Findings:**

In this systematic review and meta-analysis of 29 randomized clinical trials involving 2485 individuals, greater weight loss was achieved with time-restricted eating, lower meal frequency, and earlier caloric distribution in the day.

**Meaning:**

This meta-analysis suggests that meal timing strategies, especially time-restricted eating, lower meal frequency, and consuming calories earlier in the day, may help an individual achieve greater weight loss when implemented for a minimum duration of 12 weeks, informing dietary recommendations for better weight management and improved metabolic health.

## Teaser

This systematic review and meta-analysis evaluates the association between meal timing strategies and anthropometric and metabolic outcomes.

## Introduction

One in 8 people are living with obesity and 43% are overweight.^1^ Overweight and obesity are associated with increased risk of type 2 diabetes, heart disease, cancers, and premature death.^2^ Dietary modification is a key element of obesity management and includes reducing calorie intake or altering macronutrient composition or dietary patterns.^3,4,5^ Long-term adherence is a major challenge for many dietary approaches to weight loss. While calorie reduction is fundamental for weight loss, recent interest has emerged in meal timing strategies (ie, temporal distribution of meals throughout the day), such as time-restricted eating (TRE), reducing meal frequency, and altering calorie distribution across the day, for their potential to provide an alternative strategy for individuals who find daily demands of counting calories in traditional continuous energy restriction approaches challenging.^6,7,8,9,10,11,12^

Many individuals eat for more than 14 hours a day and snack late at night, which can worsen glycemic control and increase the risk of type 2 diabetes.^13^ Therefore, intermittent fasting is a popular weight loss strategy. TRE, a form of intermittent fasting, involves fasting and eating within a 24-hour cycle, typically consolidating calorie intake to 6- to 10-hour periods during the active phase of the day, without necessarily altering diet quality and quantity.

Meal timing approaches (including TRE) might represent a promising, attractive, and easy-to-adapt strategy for the management of obesity and prevention of metabolic disorders. To date, research efforts including systematic reviews have focused on randomized clinical trials (RCTs) in adults with overweight or obesity with a primary focus on single meal timing approaches—reducing calories in evening meals, varying eating windows across the day, and follow-up durations (typically <12 weeks) limiting long-term efficacy of such interventions since short-term benefits are often not sustained in the long-term.^3^ Subsequent trials in diverse populations with longer follow-up have been published, showing mixed beneficial effects on weight loss and metabolic health.^14,15,16,17,18,19,20,21^ Therefore, the aim of this systematic review was to evaluate existing evidence and determine the long-term (beyond 12 weeks) association between meal timing strategies and anthropometric and metabolic outcomes in adults with or without metabolic disease.

## Methods

This systematic review adhered to the Cochrane methods^22^ and the Preferred Reporting Items for Systematic Reviews and Meta-analyses (PRISMA) reporting guideline.^23^ We prospectively registered the protocol with PROSPERO (CRD42023474391). The Bond University human research ethics committee deemed this study exempt from approval and the need for informed consent because it collected and synthesized publicly available nonidentifiable data from previously published studies.

### Data Sources and Search Strategy

We searched Medline (via Ovid), Embase (via Elsevier), CINAHL (via EBSCO host), and Cochrane CENTRAL on October 17, 2023. We designed the search strategy using free text and key terms including *meal frequency*, *meal timing*, *time-restricted eating*, and *intermittent fasting*, with no language restrictions. We used systematic review accelerator tools^24^ to refine and translate the search for other databases (eTable 1 in Supplement 1). Forward-backward citation analysis was performed using SpiderCite.^24^ Duplicate citations were removed using Deduplicator^24^ and Covidence.^22^

### Eligibility Criteria and Study Selection

We included RCTs involving adults aged 18 years and older with or without comorbidities and evaluated the association between temporal distribution of isocaloric meals throughout the biological day (including TRE, meal frequency, and calorie distribution) and weight or body mass index (BMI; calculated as weight in kilograms divided by height in meters squared) over 12 or more weeks. Studies were excluded if they involved participants with eating disorders, who had underwent bariatric surgery, or who were pregnant. We excluded non-RCTs and observational studies. Two reviewers (A.E., N.C., V.L., and L.A.) independently screened titles and abstracts and full texts in duplicate using Covidence.^22^ Discrepancies were resolved through consensus.

### Data Extraction

Two reviewers (A.E., N.C., and V.L.) extracted data independently using a prospectively developed data extraction template. Discrepancies were resolved through consensus or in consultation with a third reviewer (L.A.). Extracted data included: (1) study characteristics (eg, country and study design); (2) participant characteristics (eg, comorbidities and BMI); and (3) details of intervention(s) (eg, intensity and delivery). Primary outcomes were anthropometric measures (ie, body weight, BMI, and waist circumference). Secondary outcomes were metabolic (ie, glycated hemoglobin; HbA_1c_, fasting glucose, low-density lipoprotein cholesterol; LDL, blood pressure; BP, and energy intake).

For each outcome, we extracted first the within-group pre-post mean difference (MD) from baseline to the latest follow-up and SD. If not reported, baseline and latest follow-up measures were extracted, and the pre-post MD and SD were calculated.^25^ When SDs were not reported, we estimated SD from SEs, CIs, and *P* values.^25^ We converted median (IQR) to mean (SD).^26^ When data were not available, we extracted missing data from the studies’ other published reports. If still missing, we contacted authors for necessary data. If data were not received within 6 weeks from the initial request date, the data were not included.

### Risk of Bias Assessment and Certainty of Evidence

Two reviewers (A.E., N.C., V.L., and L.A.) independently assessed the risk of bias in the effect of assignment of intervention (ie, intention-to-treat analysis) on primary and secondary outcomes for each included RCT using the Cochrane Risk of Bias 2 tool.^27^ Discrepancies were resolved through consensus. We assessed the risk of bias as low, some concerns, or high for each of the following domains: bias due to randomization, deviations from the intended intervention, missing outcome data, bias in measurement of the outcome, and bias in selection of the reported results.

We rated the overall certainty of evidence for each outcome using the Grading of Recommendations Assessment, Development, and Evaluation (GRADE) approach in 5 domains: risk of bias, inconsistency, indirectness, imprecision, and publication bias.^28^ Certainty of evidence was rated as high, moderate, low, or very low certainty. Disagreements were resolved with consensus and, as necessary, in consultation with a third reviewer (L.A.). We reported intervention effects using GRADE-recommended language.^29^

### Statistical Analysis

#### Data Synthesis and Analysis

We performed meta-analyses when 2 or more studies reported data for the same outcome. We used an inverse-variance random effects model to calculate the pooled effect estimates using Review Manager RevMan version 5.4 (Cochrane Collaboration)^30^ and R version 4.3.1, meta package (R Project for Statistical Computing).

We presented pooled effect estimates as MDs with 95% CIs for continuous outcomes. All data analyzed were converted to conventional units.^31^ When trials had multiple treatment groups, we divided the number of participants in the placebo group by the number of treatment groups.

We assessed statistical heterogeneity using the Cochrane Q χ^2^ test and quantified using the *I*^2^ statistic, where lower than 25% indicated low heterogeneity, 25% to 50% indicated moderate heterogeneity, and greater than 50% indicated high heterogeneity. We assessed publication bias and/or small studies effect using visual inspection of a funnel plot when 10 or more RCTs were available within an analysis. Two-sided *P *values less than .05 were considered statistically significant.

#### Subgroups and Sensitivity Analysis

We conducted subgroup analyses by gender (≥80% women vs <80% women), obesity status (healthy, overweight, or obese BMI), comorbid conditions (healthy vs metabolic), intervention nature and intensity (eg, frequency of sessions), and follow-up duration (≥6 months vs <6 months). We conduced sensitivity analysis by risk of bias limiting to low-risk RCTs.

## Results

**Figure 1.**
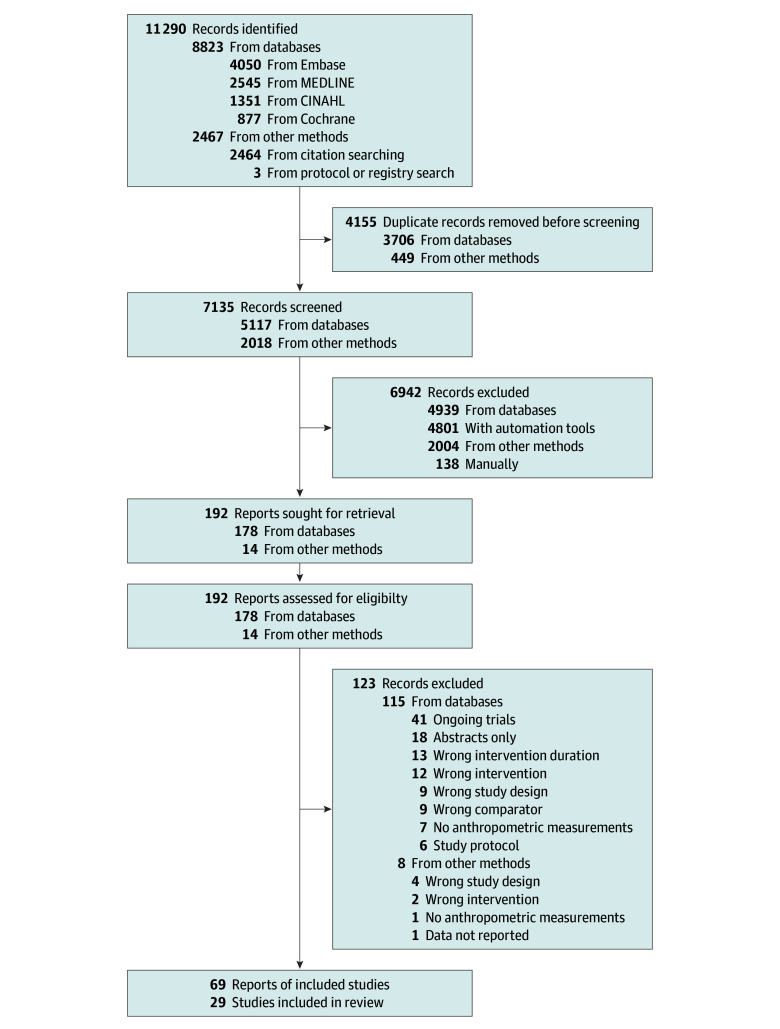
Flow Diagram of Included Randomized Clinical Trials

A total of 11 290 records were retrieved, of which 4155 were duplicates. A total of 7135 titles and abstracts were screened, and 192 potentially relevant full texts were screened for eligibility (Figure 1). Of those, we excluded 123 articles with reasons for exclusion recorded (eTable 2 in Supplement 1). Overall, we included 29 trials reported in 69 articles^14,15,16,17,18,19,20,21,32,33,34,35,36,37,38,39,40,41,42,43,44,45,46,47,48,49,50,51,52^ (eTable 3 in Supplement 1), which reported on weight (26 articles),^14,15,16,17,18,19,20,21,33,34,35,36,37,38,39,40,41,42,43,44,45,46,47,49,50,51^ BMI (21 articles),^14,15,16,17,19,21,32,33,34,35,38,41,42,43,44,45,46,47,49,50,52^ lean and/or fat-free mass (13 articles),^14,15,18,19,21,32,34,40,42,43,45,49,51^ waist circumference (14 articles),^14,15,16,17,19,21,35,38,40,41,43,44,47,49^ HbA_1c _(19 articles),^15,16,17,18,19,20,21,33,34,39,40,41,42,45,46,47,48,49,51^ fasting glucose (24 articles),^14,15,16,17,18,19,20,21,33,34,37,38,40,41,42,43,44,45,46,47,51,52^ LDL (22 articles),^14,15,16,17,18,19,20,21,33,34,37,38,40,41,42,43,44,45,46,47,51,52^ systolic and diastolic BP (16 articles),^15,18,19,20,21,34,35,37,38,40,42,43,44,45,46,49^ and energy intake (13 articles).^15,16,19,20,21,32,33,40,41,42,43,46,51^

### Characteristics of Included Studies

The 29 included RCTs^14,15,16,17,18,19,20,21,32,33,34,35,36,37,38,39,40,41,42,43,44,45,46,47,48,49,50,51,52^ enrolled a total of 2485 participants (a median [IQR] of 73 [49-110] per RCT), with a median (IQR) follow-up duration of 12 (12-26) weeks. Most study populations were of middle age (mean [SD] age 44 [9.5] years), predominantly female (1703 female [69%]); and overweight or obese (mean [SD] BMI 33 [3.5]). Most (27 trials [90%])^14,15,16,17,18,19,20,21,32,33,34,35,36,37,38,39,40,42,43,44,45,46,49,50,51,52^ of the included RCTs were parallel RCTs and one-third were conducted in the US (10 trials [34%]).^15,19,32,34,36,40,45,46,50,51^ Half of included RCTs (16 trials [55%])^16,17,20,35,37,38,39,40,41,43,44,46,47,48,50,51^ recruited participants from outpatient and/or community settings. Two-thirds of RCTs (22 trials [76%])^14,15,16,17,20,21,32,33,34,35,36,37,38,40,41,42,43,44,45,48,49,52^ enrolled healthy overweight or obese populations. Sixteen RCTs (55%)^15,16,17,19,32,35,37,38,39,40,42,44,46,47,48,51^ engaged clinicians who were specifically trained in nutrition or dietetics to deliver the interventions. The median (IQR) number of sessions required to administer the intervention in the included RCTs was 16 (10-19). Table 1 and eTable 4 in Supplement 1 provide details of included RCTs.

**Table 1.** Study Characteristics of Included Studies

Source, y, and country	Duration, RCT type^a^	Baseline participant characteristics				Intervention groups	Energy balance, macronutrient distribution and cointerventions	Outcomes measured
		No. of participants (% female)	Age, mean (SD), y	Population health	BMI, mean (SD)^b^			
Time-restricted eating								
Che et al,^33^ 2021 China	12 wk, parallel	120 (45.0)	C: 48.7 (9.5) I: 48.2 (9.3)	T2D overweight and obese	C: 26.1 (2.1) I: 26.4 (2.0)	C: Eat ad libitum per usual habits I: 10 h TRE (ad libitum), 8 am-6 pm	No instruction provided on energy intake or macronutrient distribution	Weight, fasting glucose, HbA_1c_, LDL-C, energy intake, adherence
Chow et al,^34^ 2020 US	12 wk, parallel	20 (85.0)	C: 44.2 (12.3) I: 46.5 (12.4)	Healthy overweight and obese	C: 34.4 (7.8) I: 33.8 (7.6)	C: Eat ad libitum per usual habits I: 8 h TRE (ad libitum during self-selected eating window)	No instruction provided on energy intake or macronutrient distribution	Weight, FFM, lean mass, HbA_1c_, fasting glucose, LDL-C, BP, adherence, energy intake^c^
de Oliveira Maranhao Pureza et al,^35^ 2021 Brazil	52 wk, parallel	58 (100.0)	C: 31.0 (7.1) I: 31.8 (6.9)	Healthy obese women	C: 33.1 (3.6) I: 33.5 (4.5)	C: Daily energy restriction I: 12 h TRE (self-selected eating window)	ER = TEE – 500-1000 kcal/d Individualized meal plan based on usual diet.	Weight, BMI, WC, BP, energy intake
Dhurandhar et al,^36^ 2014 Denmark and US	16 wk, parallel	309 (75.7)	C: 42.1 (11.2) I: 42.0 (12.4)	Healthy overweight and obese	Not stated	C: Usual eating habits; followed general good nutrition habits I: No energy intake before 11 am	Each group provided with USDA pamphlet with instructions related to their specific intervention	Weight, adherence
Jamshed et al,^40^ 2022 US	14 wk, parallel	90 (80.0)	C: 43.0 (11.0) I: 43.0 (10.0)	Healthy obese	C: 39.2 (6.8) I: 40.1 (6.6)	C: Self-selected ≥12 h eating window I: 8 h TRE (7 am-3 pm)	ER = REE – 500 kcal/d Encouraged to increased exercise to 75-150 min/wk	Weight, WC, FFM, fasting glucose, HbA_1c_, LDL-C, BP, adherence, energy intake, hunger and satiety (VAS)^c^
Kunduraci et al,^42^ 2020 Turkey	12 wk, parallel	70 (51.4)	C: 48.7 (2.1) I: 47.4 (2.2)	Metabolic syndrome overweight and obese	C: 32.8 (4.1) I: 36.5 (5.3)	C: Daily energy restriction I: 8 h TRE (self-selected eating window)	ER = −25% of habitual energy intake Personalized meal plan based on Turkey National Dietary Guidelines (Mediterranean diet)	Weight, BMI, WC, FFM, BP, LDL-C, fasting glucose, HbA_1c_, energy intake^c^
Lin et al,^15^ 2023 US	52 wk, parallel	60 (83.3)	C: 44.0 (13.0) I: 44.0 (12.0)	Healthy obese	C: 38.0 (5.0) I: 37.0 (6.0)	C: Maintained weight, physical activity habits and ≥10 h baseline eating window I: 8 h TRE (ad libitum 12-8 pm); at 26 wk, eating window widened to 10 h	No instruction provided on energy intake or macronutrient distribution	Weight, BMI, WC, lean mass, fasting glucose, HbA_1c_, LDL-C, BP, adherence, energy intake^c^
Liu et al,^43^ 2022 China	52 wk, parallel	139 (48.9)	C: 32.3 (8.8) I: 31.6 (9.3)	Healthy overweight and obese	C: 31.3 (2.6) I: 31.8 (2.9)	C: No time restriction I: 8 h TRE (8 am-4 pm)	ER = 1500-1800 kcal/d for men and 1200-1500 kcal/d for women Macronutrient distribution (40%-55% carbohydrate, 15%-20% protein, 20%-30% fat)	Weight, BMI, WC, lean mass, fasting glucose, LDL-C, BP, energy intake, adherence^c^
Lowe et al,^45^ 2020 US	12 wk, parallel	116 (39.7)	C: 46.1 (10.3) I: 46.8 (10.8)	Healthy overweight and obese	C: 32.6 (3.4) I: 32.9 (4.9)	C: 3 meals daily (7-11 am, 11 am-3 pm, 4-10 pm); snacking between meals was permitted I: 8 h TRE (ad libitum, 12-8 pm)	No instruction provided on energy intake or macronutrient distribution	Weight, BMI, WC, lean mass, fasting glucose, HbA_1c_, LDL-C, BP, adherence, energy intake^c^
Manoogian et al,^46^ 2022 US	12 wk, parallel	137 (9.0)	C: 39.6 (9.4) I: 41.1 (8.7)	Healthy fire fighters	C: 27.7 (3.9) I: 27.8 (3.6)	C: No time restriction I: 10 h TRE (ad libitum during self-selected window)	No ER Mediterranean diet (60% carbohydrates, 15% protein and 25% fat)	Weight, BMI, HbA_1c_, fasting glucose, BP, LDL-C, energy intake, adherence
Montero et al,^18^ 2023 Spain	12 wk, parallel	197 (50.0)	C: 46.7 (6.0) I: 46.8 (6.3)	Overweight and obese with ≥1 cardiometabolic risk factor	C: 33.4 (3.6) I: 32.9 (3.3)	C: Usual care I_1_: 8 h TRE (starting by 10:00 am) I_2_: 8 h TRE (starting by 1:00 pm) I_3_: 8 h TRE (self-selected eating window)	No ER Education on Mediterranean diet	Weight, lean mass, BP, fasting glucose, LDL-C, HbA_1c_^c^
Pavlou et al,^19^ 2023 US	26 wk, parallel	75 (71.0)	C:54.0 (11.0) I:56.0 (13.0)	Obese and T2D	C: 39.0 (7.0) I: 39.0 (9.0)	C: Usual care I:8 h TRE (ad libitum 12-8 pm)	No ER General healthy eating instructions	Weight, HbA_1c_, lean mass, WC, BMI, BP, LCL-C, energy intake, adherence^c^
Philips et al,^49^ 2021 Switzerland	26 wk, parallel	54 (NA)	C: 42.5 (14.0) I: 44.3 (12.8)	Metabolic syndrome	C: 27.0 (4.0) I: 28.0 (4.1)	C: Usual care I: 12 h TRE (ad libitum, self-selected eating window)	No instruction provided on energy intake or macronutrient distribution	Weight, BMI, WC, BP, fasting glucose, HbA_1c_
Roman et al,^50^ 2020 US	26 wk, parallel	24 (NA)	C and I: 41.6 (11.3)	Relapse Remitting Multiple Sclerosis	25.1 (4.9)	C: Usual diet I: 8 h TRE (ad libitum, self-selected eating window)	No instruction provided on energy intake or macronutrient distribution	Weight, BMI, adherence
Suthutvoravut et al,^20^ 2023 Thailand	12 wk, parallel	46 (69.6)	C: 52.2 (7.9) I: 55.5 (7.2)	Prediabetes overweight and obese	C: 30.3 (3.2) I: 29.2 (2.9)	C: Usual care I: 9 h TRE (ab libitum 8 am-5 pm)	No instruction provided on energy intake or macronutrient distribution	Weight, fasting glucose, HbA_1c_, LDL-C, BP
Thomas et al,^51^ 2022 US	39 wk, parallel	81 (85.2)	C: 37.8 (7.8) I: 38.3 (7.9)	Healthy overweight and obese	C: 35.2 (4.7) I: 34.8 (6.4)	C: No time restriction I: 10 h TRE (starting within 3 h of waking)	ER = REE – 10% Encouraged to perform 150 min/wk of moderate activity	Weight, BMI, FFM, fasting glucose, HbA_1c_, LDL-C, BP, adherence, energy intake, hunger and satiety (VAS and TFEQ)^c^
Wei et al,^21^ 2023 China	52 wk, parallel	88 (44.3)	C: 31.7 (8.3) I: 32.3 (10.5)	NAFLD obese	C: 32.2 (3.2) I: 32.2 (3.4)	C: No time restriction I: 8 h TRE (8 am-4 pm)	ER = 1500-1800 kcal/d for men and 1200-1500 kcal/d for women Macronutrient composition (40% to 55% carbohydrate, 15% to 20% protein, and 20% to 30% fat)	Weight, BMI, WC, FFM, fasting glucose, HbA_1c_, LDL-C, BP, adherence, energy intake^c^
Meal frequency								
Bachman et al,^32^ 2012 US	26 wk, parallel	51 (57.8)	I_1_: 51.8 (9.1) I_2_: 50.2 (10.8)	Healthy overweight or obese	I_1_: 34.9 (4.3) I_2_: 36.1 (5.2)	I_1_: 3Mdiet I_2_: Grazing group: ≥100kcal every 2-3 h (approximately 10 snacks)	ER = 1200 kcal/d for participants ≤200 lbs 1500 kcal/d for participants >200 lbs Fat intake restricted to <30% energy Encouraged to increase physical activity to 200 min moderate intensity per wk	BMI, FFM, energy intake^c^
Forslund et al,^37^ 2008 Sweden	52 wk, parallel	140 (74.0)	I_1_: 38.7 (11.6) I_2_: 40.1 (11.5)	Healthy obese	I_1_: 38.3 (5.3) I_2_: 38.4 (6.0)	I_1_: 3Mdiet I_2_: 3 snacks plus 3 meals per day	Energy restriction = TEE – 30% (minimum 1400 kcal/d)	Weight, BMI, fasting glucose, LDL-C, BP, energy intake
Grangeiro et al,^14^ 2021 Brazil	13 wk, parallel	47 (100.0)	I_1 _[n = 19]: 29.05 (9.18) I_2 _[n = 21]: 30.33 (6.72)	Healthy obese women	I_1_: 34.9 (1.6) I_2_: 35.2 (3.9)	I_1_: 6Mdiet I_2_: 3Mdiet	ER = TEE – 700 kcal Macronutrient distribution: 57% carbohydrates, 23% fat, 20% protein	Weight, BMI, WC, FFM, fasting glucose, LDL-C, energy intake, adherence^c^
Jakubowicz et al,^39^ 2019 Israel	12 wk, parallel	35 (61.0)	I_1_: 68 (8.6) I_2_: 69.5 (5.6)	Insulin-treated T2D	I_1_: 32.1 (5) I_2_: 32.6 (5)	I_1_:3Mdiet, large breakfast and smaller dinner I_2_: 6M diet (breakfast, lunch, dinner, and 3 snacks, even distribution)	ER = REE – 500 kcal Macronutrient distribution: 40% carbohydrates, 35% fat, 25% protein	Weight, fasting glucose, HbA_1c_, hunger (VAS)
Kahleova et al,^41^ 2014 Czech Republic	12 wk, crossover	54 (46.0)	59.4 (7.0)	T2D and overweight or obese	32.6 (4.9)	I_1_: 6Mdiet I_2_: 2Mdiet (breakfast and lunch).	ER = REE – 500 kcal Macronutrient distribution: 50%-55% carbohydrates, 20%-25% protein, <30% fat (<7% saturated fat) Meals provided for 50% of participants in each group	Weight, BMI, WC, fasting glucose, HbA_1c_, LDL-C, energy intake
Papakonstantinou et al,^47^ 2016 Greece	12 wk, crossover	40 (100.0)	27.0 (1.0)	Women with PCOS	I_1_: 27.3 (1.0) I_2_: 27.2 (0.9)	I_1_: 3Mdiet I_2_: 6Mdiet	Energy maintenance Macronutrient distribution: 40% carbohydrates, 25% protein, 35% fat	Weight, BMI, WC, HbA_1c_, fasting glucose, LDL-C, energy intake, hunger, satiety
Papakonstantinou et al,^48^ 2018 Greece	12 wk, crossover	35 (57.1)	I_1_: 48.5 (3.2) I_2_: 52.1 (2.7)	Prediabetes with overweight or obesity	I_1_: 32.6 (1.4) I_2_: 32.5 (1.2)	I_1_: 3Mdiet I_2_: 6Mdiet	Energy maintenance Macronutrient distribution: 45% carbohydrates, 20% protein, 35% fat	Weight, BMI, WC, hunger, satiety, energy intake, HbA_1c_, fasting glucose, LDL-C
Papakonstantinou et al,^48^ 2018 Greece	12 wk, crossover	12 (41.7)	I_1_: 52.1 (2.7) I_2_: 51.7 (3.5)	Newly diagnosed treatment-naïve T2D with overweight or obesity	I_1_: 32.5 (1.2) I_2_: 32.2 (1.5)	I_1_: 3Mdiet I_2_: 6Mdiet	Energy maintenance Macronutrient distribution: 45% carbohydrates, 20% protein, 35% fat	Weight, BMI, WC, hunger, satiety, energy intake, HbA_1c_, fasting glucose, LDL-C
Zargaran et al,^52^ 2014 Iran	12 wk, parallel	90 (80.0)	NA (20-60 y)	Healthy overweight	C: 30.3 (4.7) I: 30.9 (5.2)	C: Normal diet (most consisted of 3 meals and 2 snacks) I: 6Mdiet (iso-caloric meals)	ER = TEE – 400 kcal	BMI, LDL-C
Calorie distribution								
Jakubowicz et al,^38^ 2013 Israel	12 wk, parallel	93 (100.0)	I_1_: 45.1 (7.46) I_2_: 46.5 (6.86)	Metabolic syndrome overweight and obese	I_1_: 32.3 (0.2) I_2_: 32.2 (0.2)	I_1_: HCB (breakfast 700 kcal, lunch 500 kcal, dinner 200 kcal) I_2_: HCD (breakfast 200 kcal, lunch 500 kcal, dinner 700 kcal)	ER = 1400 ± 25 kcal	Weight, BMI, WC, BP, LDL-C, fasting glucose, hunger, satiety, adherence
Lombardo et al,^44^ 2014 Italy	12 wk, parallel	42 (100.0)	C: 43.0 (16.0) I_1_: 39.0 (17.0)	Healthy overweight and obese	C: 35.1 (4.5) I_1_: 35.8 (5.2)	C: Equal energy distribution (55% energy from breakfast, morning snack and lunch; 45% energy from afternoon snack and dinner) I: Front loading energy distribution (70% energy from breakfast, morning snack and lunch; 30% energy from afternoon snack and dinner)	ER = TEE – 600 kcal Macronutrient composition: 16% protein, 25% fat, 59% carbohydrates	Weight, BMI, WC, lean mass, fasting glucose, LDL-C, BP, energy intake^c^
Madjd et al,^16^ 2016 Iran	12 wk, parallel	80 (100.0)	I_1_: 33.9 (7.3) I_2_: 33.3 (6.7)	Healthy overweight and obese women	I_1_: 32.2 (2.2) I_2_: 32.1 (2.3)	I_1_: Middle loading (energy distribution: 15% breakfast, 15% snacks, 50% lunch, 20% dinner) I_2_: Backloading (energy distribution: 15% breakfast, 15% snacks, 20% lunch, 50% dinner)	ER (specifics unknown) Macronutrient composition: 17% protein, 23% fat (<10% saturated fat), 60% carbohydrate) Encouraged to increase physical activity to 60 min moderate activity 5 d/wk	Weight, BMI, WC, LDL-C, fasting glucose, HbA_1c_, energy intake
Madjd et al,^17^ 2021 Iran	12 wk, parallel	82 (100.0)	I_1_: 35.1 (7.4) I_2_: 34.9 (7.1)	Healthy overweight and obese	I_1_: 32.7 (2) I_2_: 32.7 (2)	I_1_: Early dinner eaten between 7-7:30 pm I_2_: Late dinner eaten between 10:30-11 pm	ER = TEE – 500-1000 kcal Energy distribution: 15% breakfast, 15% snacks, 50% lunch, 20% dinner Macronutrient composition: 17% protein, 23% fat (<10% saturated fat), 60% carbohydrate Encouraged to increase physical activity to 60 min moderate activity 5 d/wk	Weight, BMI, WC, fasting glucose, HbA_1c_, LDL-C, energy intake, adherence

Abbreviations: 2Mdiet, 2 meals per day; 3Mdiet, 3 meals per day; 6Mdiet, 6 meals per day; BMI, body mass index; BP, blood pressure; C, control group; ER, energy restriction; FFM, fat-free mass; HbA_1c_, glycated hemoglobin; HCB, high-calorie breakfast; HCD, high-calorie dinner; I, intervention group; LDL-C, low-density lipoprotein cholesterol; NA, not available; NAFLD, nonalcoholic fatty liver disease; PCOS, polycystic ovary syndrome; RCT, randomized clinical trial; REE, resting energy expenditure; T2D, type 2 diabetes; TEE, total energy expenditure; TFEQ, Three Factor Eating Questionnaire; TRE, time-restricted eating; USDA, United States Department of Agriculture; VAS, visual analog scale; WC, waist circumference.

^a^ Follow-up duration in most studies was equal to intervention duration.

^b^ Calculated as weight in kilograms divided by height in meters squared.

^c^ Lean mass and FFM have both been reported as lean mass in this study.

Of 17 RCTs (59%)^15,18,19,20,21,33,34,35,36,40,42,43,45,46,49,50,51^ that evaluated TRE, 10 (59%)^15,18,19,21,34,40,42,43,45,50^ implemented an 8-hour feeding window, and 11 (65%)^15,18,19,20,33,34,36,45,46,49,50^ instructed all participants to eat freely (TRE plus ad libitum vs ad libitum). Eight RCTs (28%)^14,32,37,39,41,47,48,52^ evaluated meal frequency; two-thirds (5 trials [63%]^14,37,39,47,48^) compared 3 meals per day with 6 meals per day. Four RCTs (14%)^16,17,38,44^ compared calorie distribution across the biological day.

### Risk of Bias

The overall risk of bias in the results of the effect of meal timing for the primary outcome (ie, weight measurement) was deemed high for two-thirds of included RCTs (22 trials [76%]),^14,16,17,18,20,32,33,34,36,37,38,39,41,42,44,46,47,48,49,50,51,52^ mostly because of bias in measurement of outcomes and missing outcome data. Seven RCTs^15,19,21,35,40,43,45^ were judged to have some concerns for overall risk of bias. None of the included RCTs judged low overall risk of bias (eFigures 1 and 2 in Supplement 1).

### Main Findings

#### TRE

##### Anthropometric Measures

**Figure 2.**
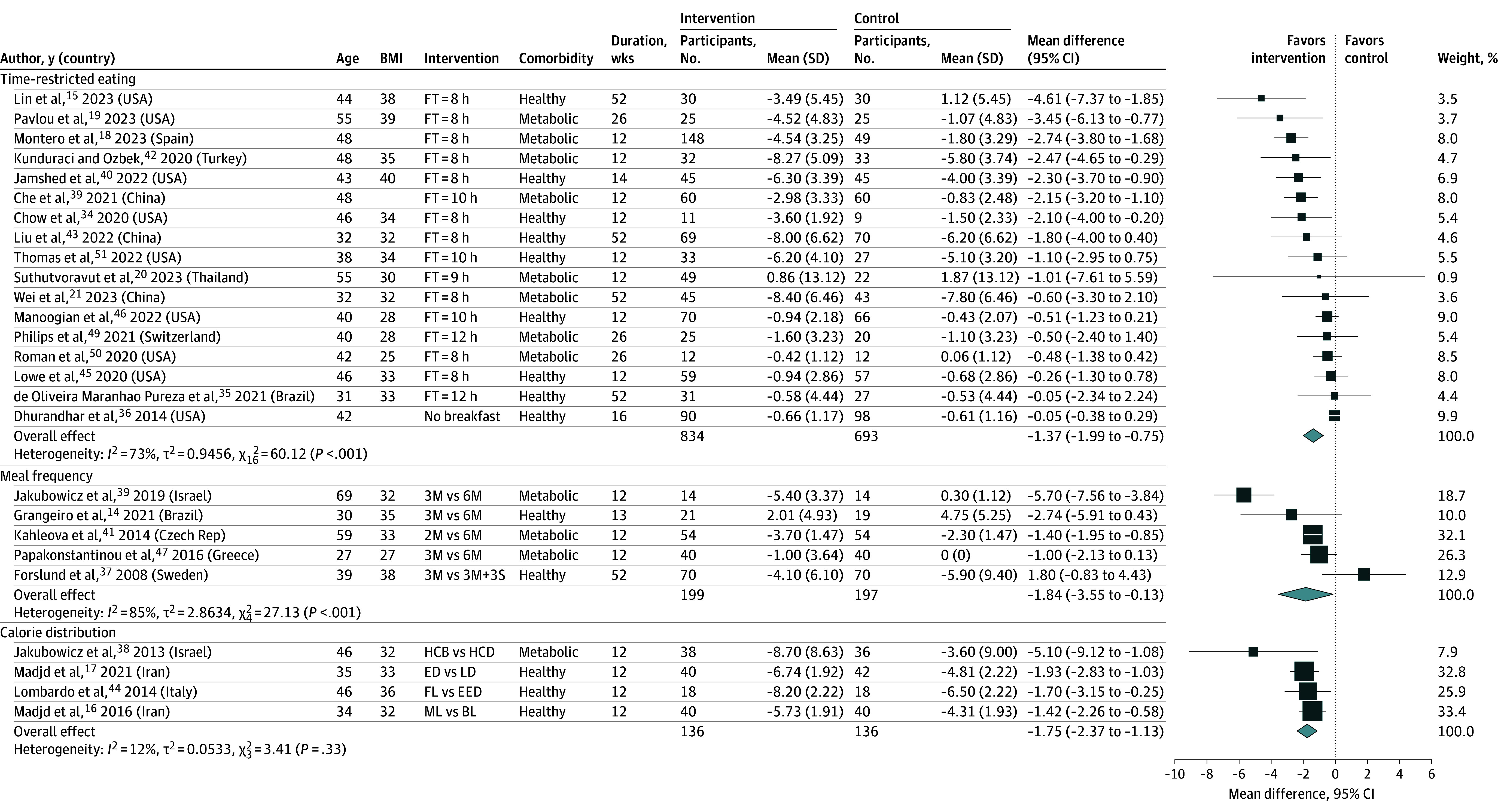
Meta-Analysis of Difference in Mean Difference (95% CIs) for the Effect of Meal Timing Interventions on Weight, Grouped by the Nature of the Meal Timing Intervention The forest plot shows effect estimates (squares) and 95% CIs (horizontal lines) for each randomized clinical trial (RCT). Larger squares indicate a larger weight has been assigned to that RCT. Left of the 0 line shows a finding in favor of interventions, whereas right of the 0 line shows a finding in favor of control. The diamond at the base of each plot demonstrates the pooled effect estimates and confidence intervals from all RCTs included in the meta-analysis. 2M/3M/6M, 2, 3, or 6 meals; 3M+3S, 3 meals and 3 snacks; BL, back loading (eating the most substantial/calorie-dense meal toward the end of the day); BMI, body mass index (calculated as weight in kilograms divided by height in meters squared); FT, feeding time; ED, early dinner; EED, equal energy distribution (spreading calorie intake evenly throughout the day’s meals); FL, front loading (consuming the largest or most calorie-dense meal early in the day, typically at breakfast or breakfast and lunch); HCB, high-calorie breakfast; HCD, high-calorie dinner; LD, late dinner; ML, middle loading (eating the most substantial/calorie-dense meal in the middle of the day, usually at lunch).

Overall, 17 RCTs^15,18,19,20,21,33,34,35,36,40,42,43,45,46,49,50,51^ (1527 participants) were included in the meta-analysis of the effect of TRE on weight change. TRE may reduce weight (MD, –1.37 kg; 95% CI, –1.99 to –0.75 kg; *I*^2^ = 73%; low-certainty evidence) (Figure 2 and Table 2). Substantial heterogeneity was partly explained by baseline BMI status (*P* for interaction = .02) and intervention intensity (*P* for interaction = .04). Participants with higher baseline BMI lost more weight than those with lower BMI (eFigure 3 in Supplement 1). Furthermore, RCTs with feeding times 8 hours or less per day were associated with greater weight loss (MD, –1.88 kg; 95% CI, –2.72 to –1.04 kg) compared with RCTs with feeding times more than 8 hours per day (MD, –0.71 kg; 95% CI, –1.42 to 0.00 kg), suggesting a dose-response association (eFigure 4 in Supplement 1).

**Table 2.** Grading of Recommendations Assessment, Development, and Evaluation (GRADE) Summary of Findings and Certainty of Evidence for Meal Timing for Anthropometric and Metabolic Measures

Outcomes	Follow-up	No. of participants (studies)	Certainty of the evidence (GRADE)^a^	Mean difference (95% CI)
Time-restricted eating				
Weight, kg	Follow-up: range 12 to 52 wk	1527 (17 RCTs)	Low^b^^,^^c^	–1.37 (–1.99 to –0.75)
Relative weight loss, %	Follow-up: range 12 to 52 wk	770 (8 RCTs)	Low^b^^,^^c^	−1.82 (−2.81 to −0.83 )
BMI^d^	Follow-up: range 12 to 52 wk	993 (14 RCTs)	Low^b^^,^^c^	–0.44 (–0.67 to –0.2)
Lean mass, kg	Follow-up: range 12 to 52 wk	858 (11 RCTs)	Moderate^b^	–0.42 (–0.7 to –0.15)
Waist circumference, cm	Follow-up: range 14 to 52 wk	530 (7 RCTs)	Low^b^	–1.96 (–3.24 to –0.68)
HbA_1c_, %	Follow-up: range 12 to 52 wk	1022 (13 RCTs)	Low^b^^,^^c^	–0.08 (–0.15 to –0.01)
Fasting plasma glucose, mg/dL	Follow-up: range 12 to 52 wk	1161 (14 RCTs)	Moderate^b^	–1.15 (–1.77 to –0.53)
LDL, mg/dL	Follow-up: range 12 to 52 wk	1151 (13 RCTs)	Low^b^^,^^d^	–1.51 (–1.3 to 4.32)
Systolic blood pressure, mmHg	Follow-up: range 12 to 52 wk	1065 (13 RCTs)	Low^b^^,^^c^^,^^d^	–0.54 (–2.42 to 1.33)
Diastolic blood pressure, mmHg	Follow-up: range 12 to 52 wk	1065 (13 RCTs)	Low^b^^,^^c^^,^^d^	–1.14 (–2.41 to 0.14)
Energy intake, kcal/d	Follow-up: range 12 to 52 wk	843 (10 RCTs)	Low^b^^,^^c^	–164 (–242.21 to –84.85)
Meal frequency, lower frequency vs higher frequency				
Weight, kg	Follow-up: range 12 to 52 wk	396 (5 RCTs)	Very low^e^^,^^f^	–1.84 (–3.55 to –0.13)
BMI^d^	Follow-up: range 12 to 26 wk	369 (5 RCTs)	Very low^e^^,^^f^	–0.65 (–1.09 to– 0.21)
Lean mass, kg	Follow-up: range 13 to 26 wk	91 (2 RCTs)	Very low^e^^,^^f^	1.35 (–0.18 to 2.88)
Waist circumference, cm	Follow-up: range 12 to 13 wk	228 (3 RCTs)	Very low^e^^,^^f^^,^^g^	–0.83 (–4.34 to 2.68)
HbA_1c_, %	Follow-up: range 12 to 12 wk	310 (4 RCTs)	Very low^e^^,^^f^^,^^g^	–0.14 (–0.39 to 0.11)
Fasting glucose, mg/dL	Follow-up: range 12 to 52 wk	490 (6 RCTs)	Very low^e^^,^^f^^,^^g^	–5.4 (–17.22 to 6.42)
LDL, mg/dL	Follow-up: range 12 to 52 wk	458 (5 RCTs)	Very low^e^^,^^f^^,^^g^	4.27 (–3.34 to 11.87)
Systolic blood pressure, mmHg	Follow-up: mean 52 wk	140 (1 RCT)	Very low^e^^,^^g^	0.7 (–3.28 to 4.68)
Diastolic blood pressure, mmHg	Follow-up: mean 52 wk	140 (1 RCT)	Very low^e^^,^^g^	–0.1 (–3.45 to 3.25)
Energy intake, kcal/d	Follow-up: range 12 to 26 wk	159 (2 RCTs)	Very low^f^^,^^g^	–0.64 (–208.34 to 207.07)
Calorie distribution, distribution of calories earlier vs later in the biological day				
Weight, kg	Follow-up: range 12 to 12 wk	272 (4 RCTs)	Low^e^	–1.75 (–2.37 to –1.13)
BMI^d^	Follow-up: range 12 to 12 wk	272 (4 RCTs)	Very low^e^^,^^f^	–1.06 (–1.82 to –0.3)
Waist circumference, cm	Follow-up: range 12 to 12 wk	272 (4 RCTs)	Very low^c^^,^^e^	–1.77 (–2.89 to –0.65)
HbA_1c_, %	Follow-up: range 12 to 12 wk	162 (2 RCTs)	Very low^b^^,^^g^	–0.01% (–0.06 to 0.04)
Fasting glucose, mg/dL	Follow-up: range 12 to 12 wk	272 (4 RCTs)	Very low^e^^,^^f^^,^^g^	–3.06 (–6.73 to 0.6)
LDL, mg/dL	Follow-up: range 12 to 12 wk	272 (4 RCTs)	Very low^e^^,^^f^^,^^g^	–3.95 (–11.67 to 3.77)
Systolic blood pressure, mmHg	Follow-up: range 12 to 12 wk	110 (2 RCTs)	Very low^e^^,^^h^	–4.96 (–8.54 to –1.38)
Diastolic blood pressure, mmHg	Follow-up: range 12 to 12 wk	110 (2 RCTs)	Very low^c^^,^^e^^,^^g^	–4.64 (–10.79 to 1.51)
Energy intake, kcal/d	Follow-up: range 12 to 12 wk	80 (1 RCT)	Very low^b^^,^^c^^,^^g^	–51 (–96.6 to –5.4)

Abbreviations: BMI, body mass index; HbA_1c_, glycated hemoglobin; LDL, low-density lipoprotein cholesterol; RCT, randomized clinical trial.

SI conversion factors: To convert fasting glucose to mmol/L, multiply by 0.0555. To convert LDL-cholesterol to mmol/L, multiply by 0.0259.

^a^ GRADE Working Group grades of evidence: high certainty: high confidence that the true effect lies close to that of the estimate of the effect; moderate certainty: moderate confidence that the true effect is likely to be close to the estimate of the effect, but there is a possibility that it is substantially different; low certainty: the true effect may be substantially different from the estimate of the effect; very low certainty: the true effect is likely to be substantially different from the estimate of effect. RCTs were downgraded from an initial high rating if a serious flaw was present in any of the following domains: risk of bias (eg, large proportion of information from studies at high risk of bias that is sufficient to affect the interpretation of results), inconsistency (ie, substantial unexplained heterogeneity *I*^2^>75%), indirectness (ie, major limitations of the generalizability of the results), imprecision (ie, 95% CIs overlap with minimally important difference for benefits or harms), and publication bias (or small study effect, where >25% of participants were from small studies with <100 participants).

^b^ Risk of bias was assessed as serious due to many trials with concerns primarily related to blinding and missing data; these studies were rated down by 1 level for risk of bias.

^c^ Inconsistency was assessed as serious due to dissimilarities in point estimates, lack of overlap in CIs, and statistical evidence of heterogeneity; these studies were rated down by 1 level for inconsistency.

^d^ Calculated as weight in kilograms divided by height in meters squared.

^e^ Risk of bias was assessed as very serious due to many trials with concerns primarily related to blinding and missing data; these studies were rated down by 2 levels for risk of bias.

^f^ Inconsistency was assessed as very serious due to dissimilarities in point estimates, lack of overlap in CIs, and statistical evidence of heterogeneity; these studies were rated down by 2 levels for inconsistency.

^g^ Imprecision was assessed as very serious because the 95% CI included a point of no difference and failed to exclude important benefits; these studies were rated down by 2 levels for imprecision.

^h^ Imprecision was assessed as serious because the 95% CI included a point of no difference and failed to exclude important benefits; these studies were rated down by 1 level for imprecision.

A meta-analysis of 12 RCTs^15,19,21,33,34,35,42,43,45,46,49,50^ (851 participants) found an association between TRE and reduced BMI (MD, –0.52; 95% CI, –0.78 to –0.26; *I*^2^ = 48%; low-certainty evidence) (eFigure 5 in Supplement 1). Baseline BMI status can partly explain the heterogeneity (*P* for interaction = .01) (eFigure 6 in Supplement 1).

A meta-analysis of 11 RCTs^15,18,19,21,34,40,42,43,45,49,51^ (858 participants) that evaluated the association of TRE with lean mass found reduced lean mass (MD, –0.42 kg; 95% CI, –0.69 to –0.10 kg; *I*^2^ = 0%; moderate-certainty evidence) (eFigure 7 in Supplement 1). Similarly, a meta-analysis of 7 RCTs^15,19,21,35,40,43,49^ (530 participants) showed that TRE was associated with reduced waist circumference (MD, –1.96 cm; 95% CI, –3.24 to –0.68 cm; *I*^2^ = 42%; low-certainty evidence) (eFigure 8 in Supplement 1). No subgroup differences were observed (eFigures 9-28 in Supplement 1). A meta-analysis of 8 RCTs (770 participants) showed that TRE was associated with reduced percentage of weight loss by an MD of 1.82% (95% CI, –2.81% to –0.83%; *I*^2^ = 66%; low-certainty evidence) (eFigure 29 in Supplement 1).

##### Metabolic Measures

**Figure 3.**
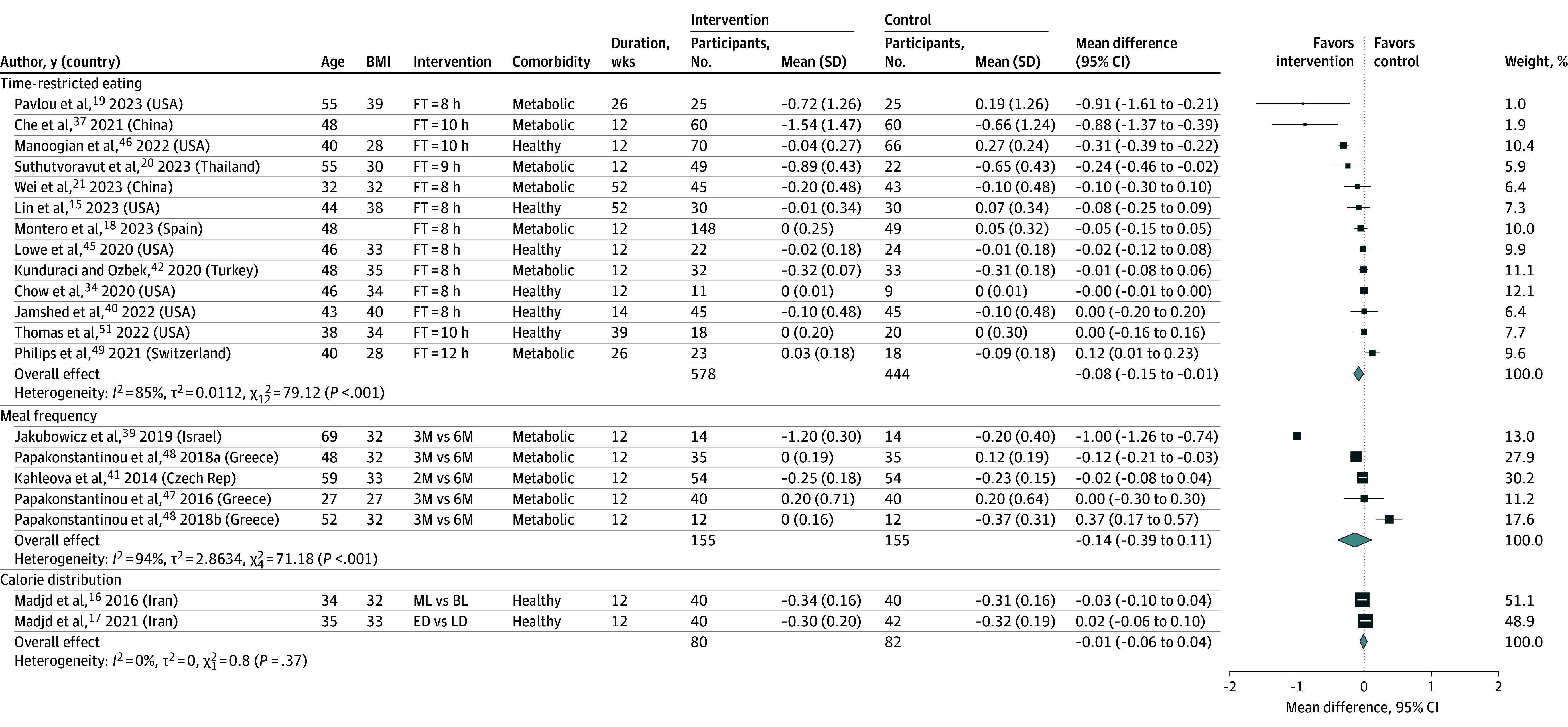
Meta-Analysis of Difference in Mean Difference (95% CIs) for the Effect of Meal Timing Interventions on HbA_1c_ (%), Grouped by the Nature of the Meal Timing Intervention The forest plot shows effect estimates (squares) and 95% CIs (horizontal lines) for each randomized clinical trial (RCT). Larger squares indicate a larger weight has been assigned to that RCT. Left of the 0 line shows a finding in favor of interventions, whereas right of the 0 line shows a finding in favor of control. The diamond at the base of each plot demonstrates the pooled effect estimates and confidence intervals from all RCTs included in the meta-analysis. 2M/3M/6M indicates 2, 3, or 6 meals; BL, back loading (eating the heaviest/most calorie-dense meal toward the end of the day); BMI, body mass index (calculated as weight in kilograms divided by height in meters squared); ED, early dinner; FT, feeding time; duration, follow-up duration in weeks; LD, late dinner; ML, middle loading (having the most substantial/calorie-dense meal in the middle of the day, usually at lunch).

Overall, 13 RCTs^15,18,19,20,21,33,34,40,42,45,46,49,51^ (1022 participants) and 14 RCTs^15,18,19,20,21,33,34,40,42,43,45,46,49,51^ (1151 participants) were included in the meta-analysis of the association of TRE with HbA_1c_ and fasting glucose, respectively. TRE was associated with reductions in both HbA_1c_ (MD, –0.08%; 95% CI, –0.15% to –0.01%; *I*^2^ = 85%; low-certainty evidence) (Figure 3)^15,18,19,20,21,33,34,40,42,45,46,49,51^ and plasma glucose (MD, –1.15 mg/dL; 95% CI, –1.77 to −0.53 mg/dL; *I*^2^ = 0%; low-certainty evidence) (eFigure 30 in Supplement 1).^15,18,19,20,21,33,34,40,42,43,45,46,49,51^ The gender of the participants (*P* for interaction = .03) and the clinicians who delivered the intervention *(P* for interaction = .03) can partly explain the heterogeneity on the effect of TRE on HbA_1c_ (eFigures 31 and 32 in Supplement 1).

A meta-analysis of 13 RCTs^15,18,19,20,21,33,34,40,42,43,45,46,51^ (1151 participants) showed that TRE was associated with reduced LDL (MD, –1.51 mg/dL; 95% CI, –1.30 to 4.32 mg/dL; *I*^2^ = 0%; low-certainty evidence) (eFigure 33 in Supplement 1). No subgroup differences were observed (eFigures 34-43 in Supplement 1). Similarly, a meta-analysis of 10 RCTs^15,19,20,21,33,40,42,43,46,51^ (843 participants) showed that TRE was associated with reduced energy intake (MD, –164 kcal/d; 95% CI, –242 to –85 kcal/d; *I*^2^ = 45%; low-certainty evidence) (eFigure 44 in Supplement 1). A subgroup analysis showed that RCTs where participants were allowed to eat freely in both groups resulted in greater reductions in energy intake compared with those with energy-restricted diets (*P* for interaction = .01) (eFigure 45 in Supplement 1).

Thirteen RCTs^15,18,19,20,21,34,35,40,42,43,45,46,49^ (1065 participants) reported on the effect of TRE on systolic (SBP) and diastolic blood pressure (DBP). TRE was not associated with change in SBP (MD, –0.54 mm Hg; 95% CI, –2.42 to 1.33 mm Hg; *I*^2^ = 38%; low-certainty evidence) (eFigure 46 in Supplement 1) and DBP (MD, –1.14 mm Hg; 95% CI, –2.41 to 0.14 mm Hg; *I*^2^ = 22%; low-certainty evidence) (eFigure 47 in Supplement 1). No subgroup differences were observed except for larger reductions in SBP among RCTs that involved clinicians trained specifically in nutrition to deliver the intervention (*P* for interaction = .02) (eFigures 48-67 and eTables 5-8 in Supplement 1).

#### Meal Frequency

##### Anthropometric Measures

Five RCTs were included in the meta-analyses of meal frequency on weight change^14,37,39,41,47^ and BMI.^14,32,41,47,52^ Lower meal frequency was associated with small reductions in weight (MD, –1.84 kg; 95% CI, –3.55 to –0.13 kg; *I*^2^ = 85%; low-certainty evidence) (Figure 2)^14,37,39,41,47^ and BMI (MD, 0.65; 95% CI, –1.09 to –0.21; *I*^2^ = 76%; low-certainty evidence) (eFigure 3 in Supplement 1).^14,32,41,47,52^ In subgroup analysis, we found that comorbidity (*P* for interaction < .001) and intervention intensity (*P* for interaction = .01) can partly explain the heterogeneity on the effect of meal frequency on BMI (eFigures 68 and 69 in Supplement 1).

We included 2 RCTs^14,32^ (91 participants) and 3 RCTs^14,41,47^ (228 participants) that reported on the effect of meal frequency on lean mass and waist circumference, respectively. No clear association was found between meal frequency and lean mass (MD, 1.35 kg; 95% CI, –0.18 to 2.88 kg; *I*^2^ = 0%; very low-certainty evidence) (eFigure 7 in Supplement 1)^14,32^ and waist circumference (MD, –0.83 cm; 95% CI, –4.34 to 2.68 cm; *I*^2^ = 97%; very low-certainty evidence)^14,41,47^ (eFigure 8 in Supplement 1).

##### Metabolic Measures

No clear association was found between meal frequency and HbA_1c_ (MD, −0.14%; 95% CI, –0.39 to 0.11; *I*^2^ = 94%; 310 participants; 4 RCTs; very low-certainty evidence) (Figure 3),^39,41,47,48^ fasting glucose (MD, –5.4 mg/dL; 95% CI, –17.2 to 6.4 mg/dL; *I*^2^ = 100%; 490 participants; 6 RCTs; very low-certainty evidence) (eFigure 30 in Supplement 1),^14,37,39,41,47,48^ LDL (MD, 4.27 mg/dL; 95% CI, –3.34 to 11.87 mg/dL; *I*^2^ = 82%; 458 participants; 5 RCTs; very low-certainty evidence) (eFigure 33 in Supplement 1),^14,37,41,47,52^ SBP (MD, 0.7 mm Hg; 95% CI, –3.28 to 4.68 mm Hg; *I*^2^ = NA; 140 participants; 1 RCT; very low-certainty evidence) (eFigure 46 in Supplement 1),^37^ DBP (MD, –0.1 mm Hg; 95% CI, –3.45 to 3.25 mm Hg; *I*^2^ = NA; 140 participants; 1 RCT; very low-certainty evidence) (eFigure 47 in Supplement 1),^37^ and energy intake (MD, –0.64 kcal/d; 95% CI, –208.3 to 207.1 kcal/d; I^2^ = 0%; 159 participants; 2 RCTs; very low-certainty evidence) (eFigure 44 in Supplement 1).^32,41^

#### Calorie Distribution

##### Anthropometric Measures

A meta-analysis of 4 RCTs^16,17,38,44^ (272 participants) that evaluated the association of calorie distribution across the biological day with weight showed that consuming the majority of calories earlier in the day resulted in more weight loss compared with consuming them later in the day (MD, –1.75 kg; 95% CI, –2.37 to –1.13 kg; *I*^2^ = 12%; low-certainty evidence) (Figure 2). Calorie distribution (eg, consuming most calorie-dense meal[s] earlier vs later in the biological day, also known as front-loading vs back-loading calories) was associated with reduced BMI (MD, –1.06; 95% CI, –1.82 to –0.30; *I*^2^ = 91%; 272 participants; 4 RCTs; very low-certainty evidence) (eFigure 3 in Supplement 1)^16,17,38,44^ and waist circumference (MD, –1.77 cm; 95% CI, –2.89 to –0.65; *I*^2^ = 53%; 272 participants; 4 RCTs; very low-certainty evidence) (eFigure 8 in Supplement 1)^16,17,38,44^; however, the evidence is very uncertain.

##### Metabolic Measures

No clear association was found between calorie distribution and HbA_1c_ (MD, –0.01%; 95% CI, –0.06 to 0.04; *I*^2^ = 0%; 162 participants; 2 RCTs; very low-certainty evidence) (eFigure 30 in Supplement 1),^16,17^ fasting glucose (MD, –3.06 mg/dL; 95% CI, –6.73 to 0.60 mg/dL; *I*^2^ = 95%; 272 participants; 4 RCTs; very low-certainty evidence) (eFigure 30 in Supplement 1),^16,17,38,44^ LDL (MD, –3.95 mg/dL; 95% CI, –11.67 to 3.77 mg/dL; *I*^2^ = 95%; 272 participants; 4 RCTs; very low-certainty evidence) (eFigure 33 in Supplement 1),^16,17,38,44^ SBP (MD, –4.96 mm Hg; 95% CI, –8.54 to –1.38 mm Hg; *I*^2^ = 22%; 110 participants; 2 RCTs; very low-certainty evidence) (eFigure 46 in Supplement 1),^38,44^ DBP (MD, –4.64 mm Hg; 95% CI, –10.79 to 1.51 mm Hg; *I*^2^ = 51%; 110 participants; 2 RCTs; very low-certainty evidence) (eFigure 47 in Supplement 1),^38,44^ and energy intake (MD, –51 kcal/d; 95% CI, –97 to –5 kcal/d; *I*^2^ = NA; 80 participants; 1 RCT; very low-certainty evidence) (eFigure 44 in Supplement 1).^16^ We could not draw robust conclusions regarding the association of calorie distribution with metabolic measures (eFigures 70-185 in Supplement 1).

## Discussion

In this meta-analysis study of RCTs, meal timing strategies were associated with small reductions in body weight, BMI, and waist circumference over more than 12 weeks (low-certainty evidence). Furthermore, our findings suggest that TRE might improve diabetes indicators such as HbA_1c_ and fasting glucose (low-certainty evidence).

Previous systematic reviews of RCTs in adults with obesity found similar results for the effect of TRE and meal frequency on weight loss, but not calorie distribution.^4,5,6,7,8,9^ Meal frequency and calorie distribution may enhance weight loss by aligning with circadian rhythms, improving metabolic efficiency, regulating appetite hormones, and reducing late-day snacking behaviors.^53^

We also found TRE-mediated effects on metabolic indicators were mostly consistent with previous systematic reviews whereby TRE significantly reduced fasting glucose but not LDL or BP, although none of the previous reviews reported improvement in HbA_1c_.^7,8,11,12^ A recent review^6^ found no significant impact of meal frequency on glycemic control, but it did observe an effect on LDL levels. A systematic review^54^ estimated that for each kilogram of weight loss, HbA_1c_ reduced by a mean of 0.1%. Collectively, our findings provide new insights on the longer-term effects of these interventions on weight loss and/or management.

The rigid nature of calorie counting in traditional weight loss interventions is often associated with higher disinhibition, energy intake, and BMI.^55^ Therefore, regular dietetic counselling is considered important for sustained weight management.^56^ However, results from our subgroup analysis showed that the benefits of meal timing on weight loss outcomes were not altered by how trained clinicians were or frequency of support provided. Although weight loss was not clinically significant (<5%),^57^ TRE may provide a simpler and more flexible approach for health care clinicians to support behavioral change in adults with BMI 25 to 40.

### Limitations

Our review has several limitations. First, most studies recruited participants from clinical settings (only 2 from community^43,46^) and involved clinicians with nutrition training, which might limit generalizability. Additionally, all studies on calorie distribution involved female participants only, which limited the generalizability to male populations.

Second, the overall quality of evidence was low because of the risk of bias and inconsistency. Most included studies rated as high risk^14,16,17,18,20,32,33,34,36,37,38,39,41,42,44,46,47,48,49,50,51,52^ because of the difficulty in blinding dietary interventions and the use of self-reported outcome measures. Our results were limited by the high heterogeneity, which was partly explained by subgroup analyses. TRE was also the dominant intervention, with limited studies on meal frequency and calorie distribution. Finally, some studies did not adequately report outcome data. However, we extracted necessary data from figures or requested it from authors. Further trials without prescribed energy restriction, larger sample sizes, similar intervention designs, and longer follow-up periods are needed.

## Conclusions

This meta-analysis found that TRE, lower meal frequency, or consuming calories earlier in the day was associated with a small amount of weight loss and improved metabolic function. Although effect sizes were small, these strategies may be plausible for sustained weight reduction.

## Notes

Supplement 1. **eTable 1.** Search strategy and key concepts/MeSH **eTable 2.** Notable studies excluded with reasoning **eTable 3.** Included studies **eTable 4.** Details of Intervention Delivery **eTable 5. **Adherence outcomes **eTable 6. **Hunger outcomes **eTable 7.** Satiety outcomes **eTable 8. **Notable clinical trials excluded **eFigure 1. **Risk of summary for included studies **eFigure 2. **Risk of bias graph for included studies **eFigure 3.** Meta-analysis of difference in mean difference (95% CIs) for the effect of time-restricted eating on weight (kg), grouped by baseline BMI status **eFigure 4.** Meta-analysis of difference in mean difference (95% CIs) for the effect of time-restricted eating interventions on weight (kg), grouped by eating window **eFigure 5. **Meta-analysis of difference in mean difference (95% CIs) for the effect of meal timing interventions on BMI (kg/m2), grouped by intervention type **eFigure 6.** Meta-analysis of difference in mean difference (95% CIs) for the effect of time-restricted eating on BMI (kg/m2), grouped by baseline BMI status **eFigure 7. **Meta-analysis of difference in mean difference (95% CIs) for the effect of meal timing interventions on lean mass (kg), grouped by the nature of the meal timing intervention **eFigure 8. **Meta-analysis of difference in mean difference (95% CIs) for the effect of meal timing interventions on waist circumference (cm), grouped by meal timing intervention **eFigure 9. **Meta-analysis of difference in mean difference (95% CIs) for the effect of time-restricted eating on lean mass (kg), grouped by gender proportion **eFigure 10.** Meta-analysis of difference in mean difference (95% CIs) for the effect of time-restricted eating on lean mass (kg), grouped by baseline BMI status **eFigure 11. **Meta-analysis of difference in mean difference (95% CIs) for the effect of time-restricted eating on lean mass (kg), grouped by energy prescription **eFigure 12. **Meta-analysis of difference in mean difference (95% CIs) for the effect of time-restricted eating on lean mass (kg), grouped by follow duration **eFigure 13.** Meta-analysis of difference in mean difference (95% CIs) for the effect of time-restricted eating on lean mass (kg), grouped by health status **eFigure 14.** Meta-analysis of difference in mean difference (95% CIs) for the effect of time-restricted eating on lean mass (kg), grouped by eating window **eFigure 15.** Meta-analysis of difference in mean difference (95% CIs) for the effect of time-restricted eating on lean mass (kg), grouped by delivery personnel **eFigure 16.** Meta-analysis of difference in mean difference (95% CIs) for the effect of time-restricted eating on lean mass (kg), grouped by frequency of contact **eFigure 17.** Meta-analysis of difference in mean difference (95% CIs) for the effect of time-restricted eating on lean mass (kg), grouped by resource provision **eFigure 18. **Meta-analysis of difference in mean difference (95% CIs) for the effect of time-restricted eating on lean mass (kg), grouped by risk of bias **eFigure 19. **Meta-analysis of difference in mean difference (95% CIs) for the effect of time-restricted eating on waist circumference (cm), grouped by gender proportion **eFigure 20.** Meta-analysis of difference in mean difference (95% CIs) for the effect of time-restricted eating on waist circumference (cm), grouped by baseline BMI status **eFigure 21. **Meta-analysis of difference in mean difference (95% CIs) for the effect of time-restricted eating on waist circumference (cm), grouped by health status **eFigure 22. **Meta-analysis of difference in mean difference (95% CIs) for the effect of time-restricted eating on waist circumference (cm), grouped by energy prescription **eFigure 23. **Meta-analysis of difference in mean difference (95% CIs) for the effect of time-restricted eating on waist circumference (cm), grouped by follow duration **eFigure 24. **Meta-analysis of difference in mean difference (95% CIs) for the effect of time-restricted eating on waist circumference (cm), grouped by eating window **eFigure 25.** Meta-analysis of difference in mean difference (95% CIs) for the effect of time-restricted eating on waist circumference (cm), grouped by frequency of contact **eFigure 26. **Meta-analysis of difference in mean difference (95% CIs) for the effect of time-restricted eating on waist circumference (cm), grouped by delivery personnel **eFigure 27. **Meta-analysis of difference in mean difference (95% CIs) for the effect of time-restricted eating on waist circumference (cm), grouped by resource provision **eFigure 28.** Meta-analysis of difference in mean difference (95% CIs) for the effect of time-restricted eating on waist circumference (cm), grouped by risk of bias **eFigure 29.** Meta-analysis of difference in mean difference (95% CIs) for the effect of meal timing interventions on HbA1c (%), grouped by the meal timing intervention **eFigure 30.** Meta-analysis of difference in mean difference (95% CIs) for the effect of meal timing interventions on fasting plasma glucose (mg/dL), grouped by the meal timing intervention **eFigure 31.** Meta-analysis of difference in mean difference (95% CIs) for the effect of time-restricted eating on HbA1c (%), grouped by gender proportion **eFigure 32.** Meta-analysis of difference in mean difference (95% CIs) for the effect of time-restricted eating on HbA1c (%), grouped by delivery personnel **eFigure 33. **Meta-analysis of difference in mean difference (95% CIs) for the effect of meal timing interventions on LDL (mg/dL), grouped by the meal timing intervention **eFigure 34.** Meta-analysis of difference in mean difference (95% CIs) for the effect of time-restricted eating on LDL (mg/dL), grouped by gender proportion **eFigure 35.** Meta-analysis of difference in mean difference (95% CIs) for the effect of time-restricted eating on LDL (mg/dL), grouped by baseline BMI status **eFigure 36. **Meta-analysis of difference in mean difference (95% CIs) for the effect of time-restricted eating on LDL (mg/dL), grouped by health status **eFigure 37. **Meta-analysis of difference in mean difference (95% CIs) for the effect of time-restricted eating on LDL (mg/dL), grouped by energy prescription **eFigure 38. **Meta-analysis of difference in mean difference (95% CIs) for the effect of time-restricted eating on LDL (mg/dL), grouped by follow duration **eFigure 39. **Meta-analysis of difference in mean difference (95% CIs) for the effect of time-restricted eating on LDL (mg/dL), grouped by eating window **eFigure 40.** Meta-analysis of difference in mean difference (95% CIs) for the effect of time-restricted eating on LDL (mg/dL), grouped by frequency of contact **eFigure 41. **Meta-analysis of difference in mean difference (95% CIs) for the effect of time-restricted eating on LDL (mg/dL), grouped by delivery personnel **eFigure 42.** Meta-analysis of difference in mean difference (95% CIs) for the effect of time-restricted eating on LDL (mg/dL), grouped by resource provision **eFigure 43. **Meta-analysis of difference in mean difference (95% CIs) for the effect of time-restricted eating on LDL (mg/dL), grouped by risk of bias **eFigure 44.** Meta-analysis of difference in mean difference (95% CIs) for the effect of meal timing interventions on energy intake (kcal/day), grouped by the meal timing intervention **eFigure 45. **Meta-analysis of difference in mean difference (95% CIs) for the effect of time-restricted eating on energy intake (kcal/day), grouped by energy prescription **eFigure 46. **Meta-analysis of difference in mean difference (95% CIs) for the effect of meal timing interventions on systolic blood pressure (mmHg), grouped by the meal timing intervention **eFigure 47. **Meta-analysis of difference in mean difference (95% CIs) for the effect of meal timing interventions on diastolic blood pressure (mmHg), grouped by the meal timing intervention **eFigure 48.** Meta-analysis of difference in mean difference (95% CIs) for the effect of time-restricted eating on systolic blood pressure (mmHg), grouped by gender proportion **eFigure 49.** Meta-analysis of difference in mean difference (95% CIs) for the effect of time-restricted eating on systolic blood pressure (mmHg), grouped by baseline BMI status **eFigure 50.** Meta-analysis of difference in mean difference (95% CIs) for the effect of time-restricted eating on systolic blood pressure (mmHg), grouped by health status **eFigure 51.** Meta-analysis of difference in mean difference (95% CIs) for the effect of time-restricted eating on systolic blood pressure (mmHg), grouped by energy prescription **eFigure 52.** Meta-analysis of difference in mean difference (95% CIs) for the effect of time-restricted eating on systolic blood pressure (mmHg), grouped by follow duration **eFigure 53. **Meta-analysis of difference in mean difference (95% CIs) for the effect of time-restricted eating on systolic blood pressure (mmHg), grouped by eating window **eFigure 54. **Meta-analysis of difference in mean difference (95% CIs) for the effect of time-restricted eating on systolic blood pressure (mmHg), grouped by frequency of contact **eFigure 55.** Meta-analysis of difference in mean difference (95% CIs) for the effect of time-restricted eating on systolic blood pressure (mmHg), grouped by delivery personnel **eFigure 56. **Meta-analysis of difference in mean difference (95% CIs) for the effect of time-restricted eating on systolic blood pressure (mmHg), grouped by resource provision **eFigure 57.** Meta-analysis of difference in mean difference (95% CIs) for the effect of time-restricted eating on systolic blood pressure (mmHg), grouped by risk of bias **eFigure 58.** Meta-analysis of difference in mean difference (95% CIs) for the effect of time-restricted eating on diastolic blood pressure (mmHg), grouped by gender proportion **eFigure 59. **Meta-analysis of difference in mean difference (95% CIs) for the effect of time-restricted eating on diastolic blood pressure (mmHg), grouped by baseline BMI status **eFigure 60.** Meta-analysis of difference in mean difference (95% CIs) for the effect of time-restricted eating on diastolic blood pressure (mmHg), grouped by health status **eFigure 61.** Meta-analysis of difference in mean difference (95% CIs) for the effect of time-restricted eating on Diastolic blood pressure (mmHg), grouped by energy prescription **eFigure 62.** Meta-analysis of difference in mean difference (95% CIs) for the effect of time-restricted eating on Diastolic blood pressure (mmHg), grouped by follow duration **eFigure 63. **Meta-analysis of difference in mean difference (95% CIs) for the effect of time-restricted eating on Diastolic blood pressure (mmHg), grouped by eating window **eFigure 64. **Meta-analysis of difference in mean difference (95% CIs) for the effect of time-restricted eating on Diastolic blood pressure (mmHg), grouped by frequency of contact **eFigure 65. **Meta-analysis of difference in mean difference (95% CIs) for the effect of time-restricted eating on Diastolic blood pressure (mmHg), grouped by delivery personnel **eFigure 66.** Meta-analysis of difference in mean difference (95% CIs) for the effect of time-restricted eating on Diastolic blood pressure (mmHg), grouped by resource provision **eFigure 67.** Meta-analysis of difference in mean difference (95% CIs) for the effect of time-restricted eating on Diastolic blood pressure (mmHg), grouped by risk of bias **eFigure 68. **Meta-analysis of difference in mean difference (95% CIs) for the effect of meal frequency on BMI (kg/m2), grouped by health status **eFigure 69.** Meta-analysis of difference in mean difference (95% CIs) for the effect of meal frequency on BMI (kg/m2), grouped by intervention intensity **eFigure 70.** Meta-analysis of difference in mean difference (95% CIs) for the effect of time restricted eating on weight (kg), grouped by gender proportion **eFigure 71.** Meta-analysis of difference in mean difference (95% CIs) for the effect of time restricted eating on weight (kg), grouped by health status **eFigure 72.** Meta-analysis of difference in mean difference (95% CIs) for the effect of time restricted eating on weight (kg), grouped by energy prescription **eFigure 73.** Meta-analysis of difference in mean difference (95% CIs) for the effect of time restricted eating on weight (kg), grouped by follow duration **eFigure 74.** Meta-analysis of difference in mean difference (95% CIs) for the effect of time restricted eating on weight (kg), grouped by frequency of contact **eFigure 75.** Meta-analysis of difference in mean difference (95% CIs) for the effect of time restricted eating on weight (kg), grouped by delivery personnel **eFigure 76.** Meta-analysis of difference in mean difference (95% CIs) for the effect of time restricted eating on weight (kg), grouped by resource provision **eFigure 77.** Meta-analysis of difference in mean difference (95% CIs) for the effect of time restricted eating on weight (kg), grouped by risk of bias **eFigure 78.** Meta-analysis of difference in mean difference (95% CIs) for the effect of meal frequency on weight (kg), grouped by gender proportion **eFigure 79. **Meta-analysis of difference in mean difference (95% CIs) for the effect of meal frequency on weight (kg), grouped by baseline BMI status **eFigure 80.** Meta-analysis of difference in mean difference (95% CIs) for the effect of meal frequency on weight (kg), grouped by health status **eFigure 81. **Meta-analysis of difference in mean difference (95% CIs) for the effect of meal frequency on weight (kg), grouped by follow duration **eFigure 82. **Meta-analysis of difference in mean difference (95% CIs) for the effect of meal frequency on weight (kg), grouped by intervention intensity **eFigure 83.** Meta-analysis of difference in mean difference (95% CIs) for the effect of meal frequency on weight (kg), grouped by frequency of contact **eFigure 84.** Meta-analysis of difference in mean difference (95% CIs) for the effect of meal frequency on weight (kg), grouped by delivery personnel **eFigure 85. **Meta-analysis of difference in mean difference (95% CIs) for the effect of meal distribution on weight (kg), grouped by baseline BMI status **eFigure 86.** Meta-analysis of difference in mean difference (95% CIs) for the effect of meal distribution on weight (kg), grouped by health status **eFigure 87.** Meta-analysis of difference in mean difference (95% CIs) for the effect of meal distribution on weight (kg), grouped by delivery personnel **eFigure 88.** Meta-analysis of difference in mean difference (95% CIs) for the effect of meal distribution on weight (kg), grouped by resource provision **eFigure 89.** Meta-analysis of difference in mean difference (95% CIs) for the effect of time restricted eating on BMI (kg/m2), grouped by gender proportion **eFigure 90. **Meta-analysis of difference in mean difference (95% CIs) for the effect of time restricted eating on BMI (kg/m2), grouped by health status **eFigure 91.** Meta-analysis of difference in mean difference (95% CIs) for the effect of time restricted eating on BMI (kg/m2), grouped by energy prescription **eFigure 92.** Meta-analysis of difference in mean difference (95% CIs) for the effect of time restricted eating on BMI (kg/m2), grouped by follow duration **eFigure 93. **Meta-analysis of difference in mean difference (95% CIs) for the effect of time restricted eating on BMI (kg/m2), grouped by eating window **eFigure 94. **Meta-analysis of difference in mean difference (95% CIs) for the effect of time restricted eating on BMI (kg/m2), grouped by frequency of contact **eFigure 95. **Meta-analysis of difference in mean difference (95% CIs) for the effect of time restricted eating on BMI (kg/m2), grouped by delivery personnel **eFigure 96. **Meta-analysis of difference in mean difference (95% CIs) for the effect of time restricted eating on BMI (kg/m2), grouped by resource provision **eFigure 97. **Meta-analysis of difference in mean difference (95% CIs) for the effect of time restricted eating on BMI (kg/m2), grouped by risk of bias **eFigure 98. **Meta-analysis of difference in mean difference (95% CIs) for the effect of meal frequency on BMI (kg/m2), grouped by gender proportion **eFigure 99.** Meta-analysis of difference in mean difference (95% CIs) for the effect of meal frequency on BMI (kg/m2), grouped by baseline BMI status **eFigure 100. **Meta-analysis of difference in mean difference (95% CIs) for the effect of meal frequency on BMI (kg/m2), grouped by follow duration **eFigure 101. **Meta-analysis of difference in mean difference (95% CIs) for the effect of meal frequency on BMI (kg/m2), grouped by frequency of contact **eFigure 102. **Meta-analysis of difference in mean difference (95% CIs) for the effect of meal frequency on BMI (kg/m2), grouped by delivery personnel **eFigure 103.** Meta-analysis of difference in mean difference (95% CIs) for the effect of meal frequency on BMI (kg/m2), grouped by resource provision **eFigure 104.** Meta-analysis of difference in mean difference (95% CIs) for the effect of meal distribution on BMI (kg/m2), grouped by baseline BMI status **eFigure 105.** Meta-analysis of difference in mean difference (95% CIs) for the effect of meal distribution on BMI (kg/m2), grouped by health status **eFigure 106. **Meta-analysis of difference in mean difference (95% CIs) for the effect of meal distribution on BMI (kg/m2), grouped by delivery personnel **eFigure 107.** Meta-analysis of difference in mean difference (95% CIs) for the effect of meal distribution on BMI (kg/m2), grouped by resource provision **eFigure 108.** Meta-analysis of difference in mean difference (95% CIs) for the effect of meal frequency on lean mass (kg), grouped by gender proportion **eFigure 109. **Meta-analysis of difference in mean difference (95% CIs) for the effect of meal frequency on lean mass (kg), grouped by follow duration **eFigure 110.** Meta-analysis of difference in mean difference (95% CIs) for the effect of meal frequency on lean mass (kg), grouped by intervention intensity **eFigure 111.** Meta-analysis of difference in mean difference (95% CIs) for the effect of meal frequency on lean mass (kg), grouped by frequency of contact **eFigure 112. **Meta-analysis of difference in mean difference (95% CIs) for the effect of meal frequency on lean mass (kg), grouped by resource provision **eFigure 113. **Meta-analysis of difference in mean difference (95% CIs) for the effect of meal frequency on waist circumference (cm), grouped by gender proportion **eFigure 114.** Meta-analysis of difference in mean difference (95% CIs) for the effect of meal frequency on waist circumference (cm), grouped by baseline BMI status **eFigure 115. **Meta-analysis of difference in mean difference (95% CIs) for the effect of meal frequency on waist circumference (cm), grouped by health status **eFigure 116. **Meta-analysis of difference in mean difference (95% CIs) for the effect of meal frequency on waist circumference (cm), grouped by intervention intensity **eFigure 117.** Meta-analysis of difference in mean difference (95% CIs) for the effect of meal frequency on waist circumference (cm), grouped by delivery personnel **eFigure 118. **Meta-analysis of difference in mean difference (95% CIs) for the effect of meal distribution on waist circumference (cm), grouped by baseline BMI status **eFigure 119.** Meta-analysis of difference in mean difference (95% CIs) for the effect of meal distribution on waist circumference (cm), grouped by health status **eFigure 120. **Meta-analysis of difference in mean difference (95% CIs) for the effect of meal distribution on waist circumference (cm), grouped by delivery personnel **eFigure 121.** Meta-analysis of difference in mean difference (95% CIs) for the effect of meal distribution on waist circumference (cm), grouped by resource provision **eFigure 122.** Meta-analysis of difference in mean difference (95% CIs) for the effect of time-restricted eating on energy intake (kcal/day), grouped by gender proportion **eFigure 123.** Meta-analysis of difference in mean difference (95% CIs) for the effect of time-restricted eating on energy intake (kcal/day), grouped by baseline BMI status **eFigure 124.** Meta-analysis of difference in mean difference (95% CIs) for the effect of time-restricted eating on energy intake (kcal/day), grouped by health status **eFigure 125.** Meta-analysis of difference in mean difference (95% CIs) for the effect of time-restricted eating on energy intake (kcal/day), grouped by follow duration **eFigure 126.** Meta-analysis of difference in mean difference (95% CIs) for the effect of time-restricted eating on energy intake (kcal/day), grouped by eating window **eFigure 127. **Meta-analysis of difference in mean difference (95% CIs) for the effect of time-restricted eating on energy intake (kcal/day), grouped by frequency of contact **eFigure 128.** Meta-analysis of difference in mean difference (95% CIs) for the effect of time-restricted eating on energy intake (kcal/day), grouped by delivery personnel **eFigure 129. **Meta-analysis of difference in mean difference (95% CIs) for the effect of time-restricted eating on energy intake (kcal/day), grouped by resource provision **eFigure 130.** Meta-analysis of difference in mean difference (95% CIs) for the effect of time-restricted eating on energy intake (kcal/day), grouped by risk of bias **eFigure 131.** Meta-analysis of difference in mean difference (95% CIs) for the effect of time-restricted eating on HbA1c (%), grouped by baseline BMI status **eFigure 132.** Meta-analysis of difference in mean difference (95% CIs) for the effect of time-restricted eating on HbA1c (%), grouped by health status **eFigure 133. **Meta-analysis of difference in mean difference (95% CIs) for the effect of time-restricted eating on HbA1c (%), grouped by energy prescription **eFigure 134. **Meta-analysis of difference in mean difference (95% CIs) for the effect of time-restricted eating on HbA1c (%), grouped by follow duration **eFigure 135. **Meta-analysis of difference in mean difference (95% CIs) for the effect of time-restricted eating on HbA1c (%), grouped by eating window **eFigure 136.** Meta-analysis of difference in mean difference (95% CIs) for the effect of time-restricted eating on HbA1c (%), grouped by frequency of contact **eFigure 137.** Meta-analysis of difference in mean difference (95% CIs) for the effect of time-restricted eating on HbA1c (%), grouped by resource provision **eFigure 138.** Meta-analysis of difference in mean difference (95% CIs) for the effect of time-restricted eating on HbA1c (%), grouped by risk of bias **eFigure 139. **Meta-analysis of difference in mean difference (95% CIs) for the effect of meal frequency on HbA1c (%), grouped by gender proportion **eFigure 140.** Meta-analysis of difference in mean difference (95% CIs) for the effect of meal frequency on HbA1c (%), grouped by baseline BMI status **eFigure 141.** Meta-analysis of difference in mean difference (95% CIs) for the effect of meal frequency on HbA1c (%), grouped by intervention intensity **eFigure 142.** Meta-analysis of difference in mean difference (95% CIs) for the effect of meal frequency on HbA1c (%), grouped by frequency of contact **eFigure 143.** Meta-analysis of difference in mean difference (95% CIs) for the effect of meal frequency on HbA1c (%), grouped by delivery personnel **eFigure 144. **Meta-analysis of difference in mean difference (95% CIs) for the effect of time-restricted eating on fasting glucose (mg/dL), grouped by gender proportion **eFigure 145.** Meta-analysis of difference in mean difference (95% CIs) for the effect of time-restricted eating on fasting glucose (mg/dL), grouped by baseline BMI status **eFigure 146.** Meta-analysis of difference in mean difference (95% CIs) for the effect of time-restricted eating on fasting glucose (mg/dL), grouped by health status **eFigure 147. **Meta-analysis of difference in mean difference (95% CIs) for the effect of time-restricted eating on fasting glucose (mg/dL), grouped by energy prescription **eFigure 148.** Meta-analysis of difference in mean difference (95% CIs) for the effect of time-restricted eating on fasting glucose (mg/dL), grouped by follow duration **eFigure 149. **Meta-analysis of difference in mean difference (95% CIs) for the effect of time-restricted eating on fasting glucose (mg/dL), grouped by eating window **eFigure 150. **Meta-analysis of difference in mean difference (95% CIs) for the effect of time-restricted eating on fasting glucose (mg/dL), grouped by frequency of contact **eFigure 151.** Meta-analysis of difference in mean difference (95% CIs) for the effect of time-restricted eating on fasting glucose (mg/dL), grouped by delivery personnel **eFigure 152. **Meta-analysis of difference in mean difference (95% CIs) for the effect of time-restricted eating on fasting glucose (mg/dL), grouped by resource provision **eFigure 153. **Meta-analysis of difference in mean difference (95% CIs) for the effect of time-restricted eating on fasting glucose (mg/dL), grouped by risk of bias **eFigure 154. **Meta-analysis of difference in mean difference (95% CIs) for the effect of meal frequency on fasting glucose (mg/dL), grouped by gender proportion **eFigure 155.** Meta-analysis of difference in mean difference (95% CIs) for the effect of meal frequency on fasting glucose (mg/dL), grouped by baseline BMI status **eFigure 156. **Meta-analysis of difference in mean difference (95% CIs) for the effect of meal frequency on fasting glucose (mg/dL), grouped by health status **eFigure 157. **Meta-analysis of difference in mean difference (95% CIs) for the effect of meal frequency on fasting glucose (mg/dL), grouped by follow duration **eFigure 158.** Meta-analysis of difference in mean difference (95% CIs) for the effect of meal frequency on fasting glucose (mg/dL), grouped by intervention intensity **eFigure 159.** Meta-analysis of difference in mean difference (95% CIs) for the effect of meal frequency on fasting glucose (mg/dL), grouped by frequency of contact **eFigure 160.** Meta-analysis of difference in mean difference (95% CIs) for the effect of meal frequency on fasting glucose (mg/dL), grouped by delivery personnel **eFigure 161. **Meta-analysis of difference in mean difference (95% CIs) for the effect of meal distribution on fasting glucose (mg/dL), grouped by baseline BMI status **eFigure 162.** Meta-analysis of difference in mean difference (95% CIs) for the effect of meal distribution on fasting glucose (mg/dL), grouped by health status **eFigure 163.** Meta-analysis of difference in mean difference (95% CIs) for the effect of meal distribution on fasting glucose (mg/dL), grouped by delivery personnel **eFigure 164.** Meta-analysis of difference in mean difference (95% CIs) for the effect of meal distribution on fasting glucose (mg/dL), grouped by resource provision **eFigure 165.** Meta-analysis of difference in mean difference (95% CIs) for the effect of meal frequency on LDL (mg/dL), grouped by gender proportion **eFigure 166.** Meta-analysis of difference in mean difference (95% CIs) for the effect of meal frequency on LDL (mg/dL), grouped by baseline BMI status **eFigure 167.** Meta-analysis of difference in mean difference (95% CIs) for the effect of meal frequency on LDL (mg/dL), grouped by health status **eFigure 168.** Meta-analysis of difference in mean difference (95% CIs) for the effect of meal frequency on LDL (mg/dL), grouped by follow duration **eFigure 169. **Meta-analysis of difference in mean difference (95% CIs) for the effect of meal frequency on LDL (mg/dL), grouped by intervention intensity **eFigure 170.** Meta-analysis of difference in mean difference (95% CIs) for the effect of meal frequency on LDL (mg/dL), grouped by frequency of contact **eFigure 171.** Meta-analysis of difference in mean difference (95% CIs) for the effect of meal frequency on LDL (mg/dL), grouped by delivery personnel **eFigure 172. **Meta-analysis of difference in mean difference (95% CIs) for the effect of meal frequency on LDL (mg/dL), grouped by resource provision **eFigure 173.** Meta-analysis of difference in mean difference (95% CIs) for the effect of meal distribution on LDL (mg/dL), grouped by baseline BMI status **eFigure 174.** Meta-analysis of difference in mean difference (95% CIs) for the effect of meal distribution on LDL (mg/dL), grouped by health status **eFigure 175.** Meta-analysis of difference in mean difference (95% CIs) for the effect of meal distribution on LDL (mg/dL), grouped by delivery personnel **eFigure 176.** Meta-analysis of difference in mean difference (95% CIs) for the effect of meal distribution on LDL (mg/dL), grouped by resource provision **eFigure 177.** Funnel plots for weight (TRE vs control) **eFigure 178.** Funnel plots for BMI (TRE vs control) **eFigure 179.** Funnel plots for lean mass (TRE vs control) **eFigure 180. **Funnel plots for energy intake (TRE vs control) **eFigure 181. **Funnel plots for HbA1c (TRE vs control) **eFigure 182. **Funnel plots for fasting glucose (TRE vs control) **eFigure 183. **Funnel plots for LDL (TRE vs control) **eFigure 184.** Funnel plots for systolic blood pressure (TRE vs control) **eFigure 185. **Funnel plots for diastolic blood pressure (TRE vs control)

Supplement 2.
Data Sharing Statement
