# Supplementary material for: Meal Timing and Anthropometric and Metabolic Outcomes: A Systematic Review and Meta-Analysis
Source: JAMA Netw Open. 2024 Nov 1;7(11):e2442163. doi: 10.1001/jamanetworkopen.2024.42163 (PMC11530941; doi:10.1001/jamanetworkopen.2024.42163)
Supplement: Supplement 1. — eTable 1. Search strategy and key concepts/MeSH eTable 2. Notable studies excluded with reasoning eTable 3. Included studies eTable 4. Details of Intervention Delivery eTable 5. Adherence outcomes eTable 6. Hunger outcomes eTable 7. Satiety outcomes eTable 8. Notable clinical trials excluded eFigure 1. Risk of summary for included studies eFigure 2. Risk of bias graph for included studies eFigure 3. Meta-analysis of difference in mean difference (95% CIs) for the effect of time-restricted eating on weight (kg), grouped by baseline BMI status eFigure 4. Meta-analysis of difference in mean difference (95% CIs) for the effect of time-restricted eating interventions on weight (kg), grouped by eating window eFigure 5. Meta-analysis of difference in mean difference (95% CIs) for the effect of meal timing interventions on BMI (kg/m2), grouped by intervention type eFigure 6. Meta-analysis of difference in mean difference (95% CIs) for the effect of time-restricted eating on BMI (kg/m2), grouped by baseline BMI status eFigure 7. Meta-analysis of difference in mean difference (95% CIs) for the effect of meal timing interventions on lean mass (kg), grouped by the nature of the meal timing intervention eFigure 8. Meta-analysis of difference in mean difference (95% CIs) for the effect of meal timing interventions on waist circumference (cm), grouped by meal timing intervention eFigure 9. Meta-analysis of difference in mean difference (95% CIs) for the effect of time-restricted eating on lean mass (kg), grouped by gender proportion eFigure 10. Meta-analysis of difference in mean difference (95% CIs) for the effect of time-restricted eating on lean mass (kg), grouped by baseline BMI status eFigure 11. Meta-analysis of difference in mean difference (95% CIs) for the effect of time-restricted eating on lean mass (kg), grouped by energy prescription eFigure 12. Meta-analysis of difference in mean difference (95% CIs) for the effect of time-restricted eating on lean mass (kg), grouped [file jamanetwopen-e2442163-s001.pdf]

Supplemental Online Content

Liu HY, Eso A, Cook N, O'Neill H, Albarqouni L. Effect of meal timing on anthropometric and metabolic outcomes: a systematic review and meta-analysis. *JAMA Netw Open*. 2024;7(11):e2442163. doi:10.1001/jamanetworkopen.2024.42163

eTable 1. Search strategy and key concepts/MeSH

eTable 2. Notable studies excluded with reasoning

eTable 3. Included studies

eTable 4. Details of Intervention Delivery

eTable 5. Adherence outcomes

eTable 6. Hunger outcomes

eTable 7. Satiety outcomes

eTable 8. Notable clinical trials excluded

eFigure 1. Risk of summary for included studies

eFigure 2. Risk of bias graph for included studies

eFigure 3. Meta-analysis of difference in mean difference (95% CIs) for the effect of time-restricted eating on weight (kg), grouped by baseline BMI status

eFigure 4. Meta-analysis of difference in mean difference (95% CIs) for the effect of time-restricted eating interventions on weight (kg), grouped by eating window

eFigure 5. Meta-analysis of difference in mean difference (95% CIs) for the effect of meal timing interventions on BMI (kg/m2), grouped by intervention type

eFigure 6. Meta-analysis of difference in mean difference (95% CIs) for the effect of time-restricted eating on BMI (kg/m2), grouped by baseline BMI status

eFigure 7. Meta-analysis of difference in mean difference (95% CIs) for the effect of meal timing interventions on lean mass (kg), grouped by the nature of the meal timing intervention

eFigure 8. Meta-analysis of difference in mean difference (95% CIs) for the effect of meal timing interventions on waist circumference (cm), grouped by meal timing intervention

eFigure 9. Meta-analysis of difference in mean difference (95% CIs) for the effect of time-restricted eating on lean mass (kg), grouped by gender proportion

eFigure 10. Meta-analysis of difference in mean difference (95% CIs) for the effect of time-restricted eating on lean mass (kg), grouped by baseline BMI status

© 2024 Liu HY et al. *JAMA Network Open.*

eFigure 11. Meta-analysis of difference in mean difference (95% CIs) for the effect of time-restricted eating on lean mass (kg), grouped by energy prescription

eFigure 12. Meta-analysis of difference in mean difference (95% CIs) for the effect of time-restricted eating on lean mass (kg), grouped by follow duration

eFigure 13. Meta-analysis of difference in mean difference (95% CIs) for the effect of time-restricted eating on lean mass (kg), grouped by health status

eFigure 14. Meta-analysis of difference in mean difference (95% CIs) for the effect of time-restricted eating on lean mass (kg), grouped by eating window

eFigure 15. Meta-analysis of difference in mean difference (95% CIs) for the effect of time-restricted eating on lean mass (kg), grouped by delivery personnel

eFigure 16. Meta-analysis of difference in mean difference (95% CIs) for the effect of time-restricted eating on lean mass (kg), grouped by frequency of contact

eFigure 17. Meta-analysis of difference in mean difference (95% CIs) for the effect of time-restricted eating on lean mass (kg), grouped by resource provision

eFigure 18. Meta-analysis of difference in mean difference (95% CIs) for the effect of time-restricted eating on lean mass (kg), grouped by risk of bias

eFigure 19. Meta-analysis of difference in mean difference (95% CIs) for the effect of time-restricted eating on waist circumference (cm), grouped by gender proportion

eFigure 20. Meta-analysis of difference in mean difference (95% CIs) for the effect of time-restricted eating on waist circumference (cm), grouped by baseline BMI status

eFigure 21. Meta-analysis of difference in mean difference (95% CIs) for the effect of time-restricted eating on waist circumference (cm), grouped by health status

eFigure 22. Meta-analysis of difference in mean difference (95% CIs) for the effect of time-restricted eating on waist circumference (cm), grouped by energy prescription

eFigure 23. Meta-analysis of difference in mean difference (95% CIs) for the effect of time-restricted eating on waist circumference (cm), grouped by follow duration

eFigure 24. Meta-analysis of difference in mean difference (95% CIs) for the effect of time-restricted eating on waist circumference (cm), grouped by eating window

eFigure 25. Meta-analysis of difference in mean difference (95% CIs) for the effect of time-restricted eating on waist circumference (cm), grouped by frequency of contact

eFigure 26. Meta-analysis of difference in mean difference (95% CIs) for the effect of time-restricted eating on waist circumference (cm), grouped by delivery personnel

eFigure 27. Meta-analysis of difference in mean difference (95% CIs) for the effect of time-restricted eating on waist circumference (cm), grouped by resource provision

eFigure 28. Meta-analysis of difference in mean difference (95% CIs) for the effect of time-restricted eating on waist circumference (cm), grouped by risk of bias

© 2024 Liu HY et al. *JAMA Network Open.*

eFigure 29. Meta-analysis of difference in mean difference (95% CIs) for the effect of meal timing interventions on HbA1c (%), grouped by the meal timing intervention

eFigure 30. Meta-analysis of difference in mean difference (95% CIs) for the effect of meal timing interventions on fasting plasma glucose (mg/dL), grouped by the meal timing intervention

eFigure 31. Meta-analysis of difference in mean difference (95% CIs) for the effect of time-restricted eating on HbA1c (%), grouped by gender proportion

eFigure 32. Meta-analysis of difference in mean difference (95% CIs) for the effect of time-restricted eating on HbA1c (%), grouped by delivery personnel

eFigure 33. Meta-analysis of difference in mean difference (95% CIs) for the effect of meal timing interventions on LDL (mg/dL), grouped by the meal timing intervention

eFigure 34. Meta-analysis of difference in mean difference (95% CIs) for the effect of time-restricted eating on LDL (mg/dL), grouped by gender proportion

eFigure 35. Meta-analysis of difference in mean difference (95% CIs) for the effect of time-restricted eating on LDL (mg/dL), grouped by baseline BMI status

eFigure 36. Meta-analysis of difference in mean difference (95% CIs) for the effect of time-restricted eating on LDL (mg/dL), grouped by health status

eFigure 37. Meta-analysis of difference in mean difference (95% CIs) for the effect of time-restricted eating on LDL (mg/dL), grouped by energy prescription

eFigure 38. Meta-analysis of difference in mean difference (95% CIs) for the effect of time-restricted eating on LDL (mg/dL), grouped by follow duration

eFigure 39. Meta-analysis of difference in mean difference (95% CIs) for the effect of time-restricted eating on LDL (mg/dL), grouped by eating window

eFigure 40. Meta-analysis of difference in mean difference (95% CIs) for the effect of time-restricted eating on LDL (mg/dL), grouped by frequency of contact

eFigure 41. Meta-analysis of difference in mean difference (95% CIs) for the effect of time-restricted eating on LDL (mg/dL), grouped by delivery personnel

eFigure 42. Meta-analysis of difference in mean difference (95% CIs) for the effect of time-restricted eating on LDL (mg/dL), grouped by resource provision

eFigure 43. Meta-analysis of difference in mean difference (95% CIs) for the effect of time-restricted eating on LDL (mg/dL), grouped by risk of bias

eFigure 44. Meta-analysis of difference in mean difference (95% CIs) for the effect of meal timing interventions on energy intake (kcal/day), grouped by the meal timing intervention

eFigure 45. Meta-analysis of difference in mean difference (95% CIs) for the effect of time-restricted eating on energy intake (kcal/day), grouped by energy prescription

eFigure 46. Meta-analysis of difference in mean difference (95% CIs) for the effect of meal timing interventions on systolic blood pressure (mmHg), grouped by the meal timing intervention

© 2024 Liu HY et al. *JAMA Network Open.*

eFigure 47. Meta-analysis of difference in mean difference (95% CIs) for the effect of meal timing interventions on diastolic blood pressure (mmHg), grouped by the meal timing intervention

eFigure 48. Meta-analysis of difference in mean difference (95% CIs) for the effect of time-restricted eating on systolic blood pressure (mmHg), grouped by gender proportion

eFigure 49. Meta-analysis of difference in mean difference (95% CIs) for the effect of time-restricted eating on systolic blood pressure (mmHg), grouped by baseline BMI status

eFigure 50. Meta-analysis of difference in mean difference (95% CIs) for the effect of time-restricted eating on systolic blood pressure (mmHg), grouped by health status

eFigure 51. Meta-analysis of difference in mean difference (95% CIs) for the effect of time-restricted eating on systolic blood pressure (mmHg), grouped by energy prescription

eFigure 52. Meta-analysis of difference in mean difference (95% CIs) for the effect of time-restricted eating on systolic blood pressure (mmHg), grouped by follow duration

eFigure 53. Meta-analysis of difference in mean difference (95% CIs) for the effect of time-restricted eating on systolic blood pressure (mmHg), grouped by eating window

eFigure 54. Meta-analysis of difference in mean difference (95% CIs) for the effect of time-restricted eating on systolic blood pressure (mmHg), grouped by frequency of contact

eFigure 55. Meta-analysis of difference in mean difference (95% CIs) for the effect of time-restricted eating on systolic blood pressure (mmHg), grouped by delivery personnel

eFigure 56. Meta-analysis of difference in mean difference (95% CIs) for the effect of time-restricted eating on systolic blood pressure (mmHg), grouped by resource provision

eFigure 57. Meta-analysis of difference in mean difference (95% CIs) for the effect of time-restricted eating on systolic blood pressure (mmHg), grouped by risk of bias

eFigure 58. Meta-analysis of difference in mean difference (95% CIs) for the effect of time-restricted eating on diastolic blood pressure (mmHg), grouped by gender proportion

eFigure 59. Meta-analysis of difference in mean difference (95% CIs) for the effect of time-restricted eating on diastolic blood pressure (mmHg), grouped by baseline BMI status

eFigure 60. Meta-analysis of difference in mean difference (95% CIs) for the effect of time-restricted eating on diastolic blood pressure (mmHg), grouped by health status

eFigure 61. Meta-analysis of difference in mean difference (95% CIs) for the effect of time-restricted eating on Diastolic blood pressure (mmHg), grouped by energy prescription

eFigure 62. Meta-analysis of difference in mean difference (95% CIs) for the effect of time-restricted eating on Diastolic blood pressure (mmHg), grouped by follow duration

eFigure 63. Meta-analysis of difference in mean difference (95% CIs) for the effect of time-restricted eating on Diastolic blood pressure (mmHg), grouped by eating window

eFigure 64. Meta-analysis of difference in mean difference (95% CIs) for the effect of time-restricted eating on Diastolic blood pressure (mmHg), grouped by frequency of contact

© 2024 Liu HY et al. *JAMA Network Open.*

eFigure 65. Meta-analysis of difference in mean difference (95% CIs) for the effect of time-restricted eating on Diastolic blood pressure (mmHg), grouped by delivery personnel

eFigure 66. Meta-analysis of difference in mean difference (95% CIs) for the effect of time-restricted eating on Diastolic blood pressure (mmHg), grouped by resource provision

eFigure 67. Meta-analysis of difference in mean difference (95% CIs) for the effect of time-restricted eating on Diastolic blood pressure (mmHg), grouped by risk of bias

eFigure 68. Meta-analysis of difference in mean difference (95% CIs) for the effect of meal frequency on BMI (kg/m2), grouped by health status

eFigure 69. Meta-analysis of difference in mean difference (95% CIs) for the effect of meal frequency on BMI (kg/m2), grouped by intervention intensity

eFigure 70. Meta-analysis of difference in mean difference (95% CIs) for the effect of time restricted eating on weight (kg), grouped by gender proportion

eFigure 71. Meta-analysis of difference in mean difference (95% CIs) for the effect of time restricted eating on weight (kg), grouped by health status

eFigure 72. Meta-analysis of difference in mean difference (95% CIs) for the effect of time restricted eating on weight (kg), grouped by energy prescription

eFigure 73. Meta-analysis of difference in mean difference (95% CIs) for the effect of time restricted eating on weight (kg), grouped by follow duration

eFigure 74. Meta-analysis of difference in mean difference (95% CIs) for the effect of time restricted eating on weight (kg), grouped by frequency of contact

eFigure 75. Meta-analysis of difference in mean difference (95% CIs) for the effect of time restricted eating on weight (kg), grouped by delivery personnel

eFigure 76. Meta-analysis of difference in mean difference (95% CIs) for the effect of time restricted eating on weight (kg), grouped by resource provision

eFigure 77. Meta-analysis of difference in mean difference (95% CIs) for the effect of time restricted eating on weight (kg), grouped by risk of bias

eFigure 78. Meta-analysis of difference in mean difference (95% CIs) for the effect of meal frequency on weight (kg), grouped by gender proportion

eFigure 79. Meta-analysis of difference in mean difference (95% CIs) for the effect of meal frequency on weight (kg), grouped by baseline BMI status

eFigure 80. Meta-analysis of difference in mean difference (95% CIs) for the effect of meal frequency on weight (kg), grouped by health status

eFigure 81. Meta-analysis of difference in mean difference (95% CIs) for the effect of meal frequency on weight (kg), grouped by follow duration

eFigure 82. Meta-analysis of difference in mean difference (95% CIs) for the effect of meal frequency on weight (kg), grouped by intervention intensity

© 2024 Liu HY et al. *JAMA Network Open.*

eFigure 83. Meta-analysis of difference in mean difference (95% CIs) for the effect of meal frequency on weight (kg), grouped by frequency of contact

eFigure 84. Meta-analysis of difference in mean difference (95% CIs) for the effect of meal frequency on weight (kg), grouped by delivery personnel

eFigure 85. Meta-analysis of difference in mean difference (95% CIs) for the effect of meal distribution on weight (kg), grouped by baseline BMI status

eFigure 86. Meta-analysis of difference in mean difference (95% CIs) for the effect of meal distribution on weight (kg), grouped by health status

eFigure 87. Meta-analysis of difference in mean difference (95% CIs) for the effect of meal distribution on weight (kg), grouped by delivery personnel

eFigure 88. Meta-analysis of difference in mean difference (95% CIs) for the effect of meal distribution on weight (kg), grouped by resource provision

eFigure 89. Meta-analysis of difference in mean difference (95% CIs) for the effect of time restricted eating on BMI (kg/m2), grouped by gender proportion

eFigure 90. Meta-analysis of difference in mean difference (95% CIs) for the effect of time restricted eating on BMI (kg/m2), grouped by health status

eFigure 91. Meta-analysis of difference in mean difference (95% CIs) for the effect of time restricted eating on BMI (kg/m2), grouped by energy prescription

eFigure 92. Meta-analysis of difference in mean difference (95% CIs) for the effect of time restricted eating on BMI (kg/m2), grouped by follow duration

eFigure 93. Meta-analysis of difference in mean difference (95% CIs) for the effect of time restricted eating on BMI (kg/m2), grouped by eating window

eFigure 94. Meta-analysis of difference in mean difference (95% CIs) for the effect of time restricted eating on BMI (kg/m2), grouped by frequency of contact

eFigure 95. Meta-analysis of difference in mean difference (95% CIs) for the effect of time restricted eating on BMI (kg/m2), grouped by delivery personnel

eFigure 96. Meta-analysis of difference in mean difference (95% CIs) for the effect of time restricted eating on BMI (kg/m2), grouped by resource provision

eFigure 97. Meta-analysis of difference in mean difference (95% CIs) for the effect of time restricted eating on BMI (kg/m2), grouped by risk of bias

eFigure 98. Meta-analysis of difference in mean difference (95% CIs) for the effect of meal frequency on BMI (kg/m2), grouped by gender proportion

eFigure 99. Meta-analysis of difference in mean difference (95% CIs) for the effect of meal frequency on BMI (kg/m2), grouped by baseline BMI status

eFigure 100. Meta-analysis of difference in mean difference (95% CIs) for the effect of meal frequency on BMI (kg/m2), grouped by follow duration

© 2024 Liu HY et al. *JAMA Network Open.*

eFigure 101. Meta-analysis of difference in mean difference (95% CIs) for the effect of meal frequency on BMI (kg/m2), grouped by frequency of contact

eFigure 102. Meta-analysis of difference in mean difference (95% CIs) for the effect of meal frequency on BMI (kg/m2), grouped by delivery personnel

eFigure 103. Meta-analysis of difference in mean difference (95% CIs) for the effect of meal frequency on BMI (kg/m2), grouped by resource provision

eFigure 104. Meta-analysis of difference in mean difference (95% CIs) for the effect of meal distribution on BMI (kg/m2), grouped by baseline BMI status

eFigure 105. Meta-analysis of difference in mean difference (95% CIs) for the effect of meal distribution on BMI (kg/m2), grouped by health status

eFigure 106. Meta-analysis of difference in mean difference (95% CIs) for the effect of meal distribution on BMI (kg/m2), grouped by delivery personnel

eFigure 107. Meta-analysis of difference in mean difference (95% CIs) for the effect of meal distribution on BMI (kg/m2), grouped by resource provision

eFigure 108. Meta-analysis of difference in mean difference (95% CIs) for the effect of meal frequency on lean mass (kg), grouped by gender proportion

eFigure 109. Meta-analysis of difference in mean difference (95% CIs) for the effect of meal frequency on lean mass (kg), grouped by follow duration

eFigure 110. Meta-analysis of difference in mean difference (95% CIs) for the effect of meal frequency on lean mass (kg), grouped by intervention intensity

eFigure 111. Meta-analysis of difference in mean difference (95% CIs) for the effect of meal frequency on lean mass (kg), grouped by frequency of contact

eFigure 112. Meta-analysis of difference in mean difference (95% CIs) for the effect of meal frequency on lean mass (kg), grouped by resource provision

eFigure 113. Meta-analysis of difference in mean difference (95% CIs) for the effect of meal frequency on waist circumference (cm), grouped by gender proportion

eFigure 114. Meta-analysis of difference in mean difference (95% CIs) for the effect of meal frequency on waist circumference (cm), grouped by baseline BMI status

eFigure 115. Meta-analysis of difference in mean difference (95% CIs) for the effect of meal frequency on waist circumference (cm), grouped by health status

eFigure 116. Meta-analysis of difference in mean difference (95% CIs) for the effect of meal frequency on waist circumference (cm), grouped by intervention intensity

eFigure 117. Meta-analysis of difference in mean difference (95% CIs) for the effect of meal frequency on waist circumference (cm), grouped by delivery personnel

eFigure 118. Meta-analysis of difference in mean difference (95% CIs) for the effect of meal distribution on waist circumference (cm), grouped by baseline BMI status

© 2024 Liu HY et al. *JAMA Network Open.*

eFigure 119. Meta-analysis of difference in mean difference (95% CIs) for the effect of meal distribution on waist circumference (cm), grouped by health status

eFigure 120. Meta-analysis of difference in mean difference (95% CIs) for the effect of meal distribution on waist circumference (cm), grouped by delivery personnel

eFigure 121. Meta-analysis of difference in mean difference (95% CIs) for the effect of meal distribution on waist circumference (cm), grouped by resource provision

eFigure 122. Meta-analysis of difference in mean difference (95% CIs) for the effect of time-restricted eating on energy intake (kcal/day), grouped by gender proportion

eFigure 123. Meta-analysis of difference in mean difference (95% CIs) for the effect of time-restricted eating on energy intake (kcal/day), grouped by baseline BMI status

eFigure 124. Meta-analysis of difference in mean difference (95% CIs) for the effect of time-restricted eating on energy intake (kcal/day), grouped by health status

eFigure 125. Meta-analysis of difference in mean difference (95% CIs) for the effect of time-restricted eating on energy intake (kcal/day), grouped by follow duration

eFigure 126. Meta-analysis of difference in mean difference (95% CIs) for the effect of time-restricted eating on energy intake (kcal/day), grouped by eating window

eFigure 127. Meta-analysis of difference in mean difference (95% CIs) for the effect of time-restricted eating on energy intake (kcal/day), grouped by frequency of contact

eFigure 128. Meta-analysis of difference in mean difference (95% CIs) for the effect of time-restricted eating on energy intake (kcal/day), grouped by delivery personnel

eFigure 129. Meta-analysis of difference in mean difference (95% CIs) for the effect of time-restricted eating on energy intake (kcal/day), grouped by resource provision

eFigure 130. Meta-analysis of difference in mean difference (95% CIs) for the effect of time-restricted eating on energy intake (kcal/day), grouped by risk of bias

eFigure 131. Meta-analysis of difference in mean difference (95% CIs) for the effect of time-restricted eating on HbA1c (%), grouped by baseline BMI status

eFigure 132. Meta-analysis of difference in mean difference (95% CIs) for the effect of time-restricted eating on HbA1c (%), grouped by health status

eFigure 133. Meta-analysis of difference in mean difference (95% CIs) for the effect of time-restricted eating on HbA1c (%), grouped by energy prescription

eFigure 134. Meta-analysis of difference in mean difference (95% CIs) for the effect of time-restricted eating on HbA1c (%), grouped by follow duration

eFigure 135. Meta-analysis of difference in mean difference (95% CIs) for the effect of time-restricted eating on HbA1c (%), grouped by eating window

eFigure 136. Meta-analysis of difference in mean difference (95% CIs) for the effect of time-restricted eating on HbA1c (%), grouped by frequency of contact

© 2024 Liu HY et al. *JAMA Network Open.*

eFigure 137. Meta-analysis of difference in mean difference (95% CIs) for the effect of time-restricted eating on HbA1c (%), grouped by resource provision

eFigure 138. Meta-analysis of difference in mean difference (95% CIs) for the effect of time-restricted eating on HbA1c (%), grouped by risk of bias

eFigure 139. Meta-analysis of difference in mean difference (95% CIs) for the effect of meal frequency on HbA1c (%), grouped by gender proportion

eFigure 140. Meta-analysis of difference in mean difference (95% CIs) for the effect of meal frequency on HbA1c (%), grouped by baseline BMI status

eFigure 141. Meta-analysis of difference in mean difference (95% CIs) for the effect of meal frequency on HbA1c (%), grouped by intervention intensity

eFigure 142. Meta-analysis of difference in mean difference (95% CIs) for the effect of meal frequency on HbA1c (%), grouped by frequency of contact

eFigure 143. Meta-analysis of difference in mean difference (95% CIs) for the effect of meal frequency on HbA1c (%), grouped by delivery personnel

eFigure 144. Meta-analysis of difference in mean difference (95% CIs) for the effect of time-restricted eating on fasting glucose (mg/dL), grouped by gender proportion

eFigure 145. Meta-analysis of difference in mean difference (95% CIs) for the effect of time-restricted eating on fasting glucose (mg/dL), grouped by baseline BMI status

eFigure 146. Meta-analysis of difference in mean difference (95% CIs) for the effect of time-restricted eating on fasting glucose (mg/dL), grouped by health status

eFigure 147. Meta-analysis of difference in mean difference (95% CIs) for the effect of time-restricted eating on fasting glucose (mg/dL), grouped by energy prescription

eFigure 148. Meta-analysis of difference in mean difference (95% CIs) for the effect of time-restricted eating on fasting glucose (mg/dL), grouped by follow duration

eFigure 149. Meta-analysis of difference in mean difference (95% CIs) for the effect of time-restricted eating on fasting glucose (mg/dL), grouped by eating window

eFigure 150. Meta-analysis of difference in mean difference (95% CIs) for the effect of time-restricted eating on fasting glucose (mg/dL), grouped by frequency of contact

eFigure 151. Meta-analysis of difference in mean difference (95% CIs) for the effect of time-restricted eating on fasting glucose (mg/dL), grouped by delivery personnel

eFigure 152. Meta-analysis of difference in mean difference (95% CIs) for the effect of time-restricted eating on fasting glucose (mg/dL), grouped by resource provision

eFigure 153. Meta-analysis of difference in mean difference (95% CIs) for the effect of time-restricted eating on fasting glucose (mg/dL), grouped by risk of bias

eFigure 154. Meta-analysis of difference in mean difference (95% CIs) for the effect of meal frequency on fasting glucose (mg/dL), grouped by gender proportion

© 2024 Liu HY et al. *JAMA Network Open.*

eFigure 155. Meta-analysis of difference in mean difference (95% CIs) for the effect of meal frequency on fasting glucose (mg/dL), grouped by baseline BMI status

eFigure 156. Meta-analysis of difference in mean difference (95% CIs) for the effect of meal frequency on fasting glucose (mg/dL), grouped by health status

eFigure 157. Meta-analysis of difference in mean difference (95% CIs) for the effect of meal frequency on fasting glucose (mg/dL), grouped by follow duration

eFigure 158. Meta-analysis of difference in mean difference (95% CIs) for the effect of meal frequency on fasting glucose (mg/dL), grouped by intervention intensity

eFigure 159. Meta-analysis of difference in mean difference (95% CIs) for the effect of meal frequency on fasting glucose (mg/dL), grouped by frequency of contact

eFigure 160. Meta-analysis of difference in mean difference (95% CIs) for the effect of meal frequency on fasting glucose (mg/dL), grouped by delivery personnel

eFigure 161. Meta-analysis of difference in mean difference (95% CIs) for the effect of meal distribution on fasting glucose (mg/dL), grouped by baseline BMI status

eFigure 162. Meta-analysis of difference in mean difference (95% CIs) for the effect of meal distribution on fasting glucose (mg/dL), grouped by health status

eFigure 163. Meta-analysis of difference in mean difference (95% CIs) for the effect of meal distribution on fasting glucose (mg/dL), grouped by delivery personnel

eFigure 164. Meta-analysis of difference in mean difference (95% CIs) for the effect of meal distribution on fasting glucose (mg/dL), grouped by resource provision

eFigure 165. Meta-analysis of difference in mean difference (95% CIs) for the effect of meal frequency on LDL (mg/dL), grouped by gender proportion

eFigure 166. Meta-analysis of difference in mean difference (95% CIs) for the effect of meal frequency on LDL (mg/dL), grouped by baseline BMI status

eFigure 167. Meta-analysis of difference in mean difference (95% CIs) for the effect of meal frequency on LDL (mg/dL), grouped by health status

eFigure 168. Meta-analysis of difference in mean difference (95% CIs) for the effect of meal frequency on LDL (mg/dL), grouped by follow duration

eFigure 169. Meta-analysis of difference in mean difference (95% CIs) for the effect of meal frequency on LDL (mg/dL), grouped by intervention intensity

eFigure 170. Meta-analysis of difference in mean difference (95% CIs) for the effect of meal frequency on LDL (mg/dL), grouped by frequency of contact

eFigure 171. Meta-analysis of difference in mean difference (95% CIs) for the effect of meal frequency on LDL (mg/dL), grouped by delivery personnel

eFigure 172. Meta-analysis of difference in mean difference (95% CIs) for the effect of meal frequency on LDL (mg/dL), grouped by resource provision

© 2024 Liu HY et al. *JAMA Network Open.*

eFigure 173. Meta-analysis of difference in mean difference (95% CIs) for the effect of meal distribution on LDL (mg/dL), grouped by baseline BMI status

eFigure 174. Meta-analysis of difference in mean difference (95% CIs) for the effect of meal distribution on LDL (mg/dL), grouped by health status

eFigure 175. Meta-analysis of difference in mean difference (95% CIs) for the effect of meal distribution on LDL (mg/dL), grouped by delivery personnel

eFigure 176. Meta-analysis of difference in mean difference (95% CIs) for the effect of meal distribution on LDL (mg/dL), grouped by resource provision

eFigure 177. Funnel plots for weight (TRE vs control)

eFigure 178. Funnel plots for BMI (TRE vs control)

eFigure 179. Funnel plots for lean mass (TRE vs control)

eFigure 180. Funnel plots for energy intake (TRE vs control)

eFigure 181. Funnel plots for HbA1c (TRE vs control)

eFigure 182. Funnel plots for fasting glucose (TRE vs control)

eFigure 183. Funnel plots for LDL (TRE vs control)

eFigure 184. Funnel plots for systolic blood pressure (TRE vs control)

eFigure 185. Funnel plots for diastolic blood pressure (TRE vs control)

This supplemental material has been provided by the authors to give readers additional information about their work.

© 2024 Liu HY et al. *JAMA Network Open.*

eTable 1: Search strategy and key concepts/MeSH

Medline (via Ovid)

(((Meal adj3 Frequency) or (meal adj3 timing) or "time-restricted eating" or "time-restricted feeding").tw. or exp "intermittent fasting"/ or "intermittent fasting".tw. or (breakfast adj3 skipping).tw. or "no breakfast".tw. or (morning adj3 fasting).tw. or (morning adj3 fasted).tw. or (morning adj3 fast).tw. or "time restricted fasting".tw. or "time restricted diet".tw. or "delayed eating".tw. or (meal adj3 regularity).tw. or (breakfast adj3 omission).tw. or (omitting adj3 breakfast).tw. or (skipping adj3 meal).tw. or (skipping adj3 meals).tw. or "three-meal diet".tw. or "six meal".tw. or "six meals".tw. or "chrono nutrition".tw. or chrono-nutrition.tw. or "High-calorie breakfast".tw.) and (exp "body weight"/ or "body weight".tw. or exp "body mass index"/ or overweight.tw. or obesity.tw. or "weight loss".tw.)

Cochrane CENTRAL

(((Meal:ti,ab NEAR/3 Frequency:ti,ab) OR (meal:ti,ab NEAR/3 timing:ti,ab) OR "time-restricted eating":ti,ab OR "time-restricted feeding":ti,ab) OR [mh "intermittent fasting"] OR "intermittent fasting":ti,ab OR (breakfast:ti,ab NEAR/3 skipping:ti,ab) OR "no breakfast":ti,ab OR (morning:ti,ab NEAR/3 fasting:ti,ab) OR (morning:ti,ab NEAR/3 fasted:ti,ab) OR (morning:ti,ab NEAR/3 fast:ti,ab) OR "time restricted fasting":ti,ab OR "time restricted diet":ti,ab OR "delayed eating":ti,ab OR (meal:ti,ab NEAR/3 regularity:ti,ab) OR (breakfast:ti,ab NEAR/3 omission:ti,ab) OR (omitting:ti,ab NEAR/3 breakfast:ti,ab) OR (skipping:ti,ab NEAR/3 meal:ti,ab) OR (skipping:ti,ab NEAR/3 meals:ti,ab) OR "three-meal diet":ti,ab OR "six meal":ti,ab OR "six meals":ti,ab OR "chrono nutrition":ti,ab OR chrono-nutrition:ti,ab OR "High-calorie breakfast":ti,ab) AND ([mh "body weight"] OR "body weight":ti,ab OR [mh "body mass index"] OR overweight:ti,ab OR obesity:ti,ab OR "weight loss":ti,ab)

CINAHL

((((TI Meal OR AB Meal) N3 (TI Frequency OR AB Frequency)) OR ((TI meal OR AB meal) N3 (TI timing OR AB timing)) OR (TI "time-restricted eating" OR AB "time-restricted eating") OR (TI "time-restricted feeding" OR AB "time-restricted feeding")) OR (MH "intermittent fasting+") OR (TI "intermittent fasting" OR AB "intermittent fasting") OR ((TI breakfast OR AB breakfast) N3 (TI skipping OR AB skipping)) OR (TI "no breakfast" OR AB "no breakfast") OR ((TI morning OR AB morning) N3 (TI fasting OR AB fasting)) OR ((TI morning OR AB morning) N3 (TI fasted OR AB fasted)) OR ((TI morning OR AB morning) N3 (TI fast OR AB fast)) OR (TI "time restricted fasting" OR AB "time restricted fasting") OR (TI "time restricted diet" OR AB "time restricted diet") OR (TI "delayed eating" OR AB "delayed eating") OR ((TI meal OR AB meal) N3 (TI regularity OR AB regularity)) OR ((TI breakfast OR AB breakfast) N3 (TI omission OR AB omission)) OR ((TI omitting OR AB omitting) N3 (TI breakfast OR AB breakfast)) OR ((TI skipping OR AB skipping) N3 (TI meal OR AB meal)) OR ((TI skipping OR AB skipping) N3 (TI meals OR AB meals)) OR (TI "three-meal diet" OR AB "three-meal diet") OR (TI "six meal" OR AB "six meal") OR (TI "six meals" OR AB "six meals") OR (TI "chrono nutrition" OR AB "chrono nutrition") OR (TI chrono-nutrition OR AB chrono-nutrition) OR (TI "High-calorie breakfast" OR AB "High-calorie breakfast")) AND ((MH "body weight+") OR (TI "body weight" OR AB "body weight") OR (MH "body mass index+") OR (TI overweight OR AB overweight) OR (TI obesity OR AB obesity) OR (TI "weight loss" OR AB "weight loss"))

Embase

(((meal NEAR/3 frequency):ti,ab) OR ((meal NEAR/3 timing):ti,ab) OR 'time-restricted eating':ti,ab OR 'time-restricted feeding':ti,ab OR 'intermittent fasting'/exp/mj OR 'intermittent fasting' OR 'intermittent fasting':ti,ab OR ((breakfast NEAR/3 skipping):ti,ab) OR 'no breakfast':ti,ab OR ((morning NEAR/3 fasting):ti,ab) OR ((morning NEAR/3 fasted):ti,ab) OR ((morning NEAR/3 fast):ti,ab) OR 'time restricted

fasting':ti,ab OR 'time restricted diet':ti,ab OR 'delayed eating':ti,ab OR ((meal NEAR/3 regularity):ti,ab) OR ((breakfast NEAR/3 omission):ti,ab) OR ((omitting NEAR/3 breakfast):ti,ab) OR ((skipping NEAR/3 meal):ti,ab) OR ((skipping NEAR/3 meals):ti,ab) OR 'three-meal diet':ti,ab OR 'six meal':ti,ab OR 'six meals':ti,ab OR 'chrono nutrition':ti,ab OR 'high-calorie breakfast':ti,ab) AND ('body weight'/exp OR 'body weight' OR 'body weight':ti,ab OR 'body mass'/exp/mj OR 'body mass index' OR overweight:ti,ab OR obesity:ti,ab OR 'weight loss':ti,ab)

Note. eTable 1 shows the exact search strategies used for each of the mentioned databases

eTable 2: Notable studies excluded with reasoning

Author, Date	Reason for Exclusion
**Alencar, 2015**	Wrong intervention duration
**Bantle, 2022**	No anthropometric measurements
**Crimarco, 2019**	Wrong intervention duration
**Erdem, 2022**	Wrong comparator
**Papamichou, 2022**	Study protocol
**White, 2023**	Study protocol
**Isenmann, 2021**	Wrong intervention duration
**Astbury, 2011**	Wrong intervention duration
**Nowak, 2021**	Wrong intervention
**Gabel, 2022**	Study protocol
**Bao, 2022**	Wrong intervention duration
**Pala, 2022**	Study protocol
**Moro, 2021**	Wrong comparator
**Obermayer, 2022**	Wrong intervention
**Obermayer, 2023**	Wrong intervention
**Suthutvoravut, 2022**	Study protocol
**Yang, 2023**	Wrong intervention
**Ferrocino, 2022**	Wrong study design
**Kanaley, 2014**	Wrong study design
**Griffiths, 2016**	Wrong intervention
**Munsters, 2012**	Wrong intervention duration
**Lee, 2023**	Study protocol
**Correia, 2021**	Wrong intervention duration
**Opstad, 2021**	Wrong intervention
**Godlin Jeneta, 2016**	Wrong study design
**Gabel, 2019**	Wrong study design
**Yildiran, 2019**	Wrong study design
**Agyepong, 2021**	Wrong study design
**Moore, 2022**	Wrong study design
**Yoshimura, 2017**	Wrong intervention duration
**Young, 1972**	Wrong intervention duration
**Zimmermann, 2023**	Wrong intervention duration

© 2024 Liu HY et al. *JAMA Network Open.*

**Zhang, 2022**	Wrong intervention duration
**Vondra, 1976**	Wrong study design

Note. eTable 2 details each of the full text articles excluded from the review with the excluded reason.

eTable 3: Included studies

Study	Reports
Che 2021	1. Che T, Yan C, Tian D, Zhang X, Liu X, Wu Z. Time-restricted feeding improves blood glucose and insulin sensitivity in overweight patients with type 2 diabetes: a randomised controlled trial. *Nutrition & Metabolism*. 2021;18(1)doi:10.1186/s12986-021-00613-9
Chow 2020	1. Bantle A, Alvear A, Knights D, Chow L, Johnson A. Weight Loss Associated With Time Restricted Eating Is Not Reflected in Changes in the Human Gut Microbiome. *Current Developments in Nutrition*. 2022;6doi:10.1093/cdn/nzac069.003<br>2. Chow LS, Manoogian E, Alvear AC, Wang Q, Panda S, Mashek DG. Time restricted eating (TRE) promotes weight loss, alters body composition, and improves metabolic parameters in overweight humans. 2019;68doi:10.2337/db19-2076-P<br>3. Chow LS, Manoogian ENC, Alvear A, et al. Time-Restricted Eating Effects on Body Composition and Metabolic Measures in Humans who are Overweight: A Feasibility Study. *Obesity (19307381)*. 2020;28(5)doi:10.1002/oby.22756<br>4. Crose A, Alvear A, Singroy S, et al. Time-Restricted Eating Improves Quality of Life Measures in Overweight Humans. *Nutrients*. 2021;13(5)doi:10.3390/nu13051430<br>5. Hibbing PR, Shook RP, Panda S, Manoogian ENC, Mashek DG, Chow LS. Predicting energy intake with an accelerometer-based intake-balance method. 2022;doi:10.1017/S0007114522003312<br>6. Mehfooz A, Lee YL, Wang Q, Chow LS. Time-Restricted Eating Did Not Alter Glycemic Variability in Humans Who Are Overweight and without Diabetes. *Diabetes*. 2022;71doi:10.2337/db22-699-P<br>7. Simon SL, Fleischer JG, Manoogian EN, Panda S, Mashek DG, Chow L. Objectively-measured sleep following a time restricted eating intervention in adults with obesity. 2020;43(SUPPL 1)doi:10.1093/sleep/zsaa056.1025
de Oliveira Maranhao Pureza 2021	1. de Oliveira Maranhão Pureza IR, da Silva Junior AE, Silva Praxedes DR, et al. Effects of time-restricted feeding on body weight, body composition and vital signs in low-income women with obesity: A 12-month randomized clinical trial. *Clinical Nutrition*. 2021;40(3)doi:10.1016/j.clnu.2020.06.036
Dhurandhar 2014	1. Dhurandhar EJ, Dawson J, Alcorn A, et al. The effectiveness of breakfast recommendations on weight loss: a randomized controlled trial. *American Journal of Clinical Nutrition*. 2014;100(2)doi:10.3945/ajcn.114.089573
Jamshed 2022	1. Hanick C, Jamshed H, Bryan D, et al. Effects of time-restricted eating on appetite, eating behaviors, and physical activity in adults. 2020;28(SUPPL 2)doi:10.1002/oby.23063

© 2024 Liu HY et al. *JAMA Network Open.*

2. Hanick C, Steger F, Jamshed H, et al. Early Time-Restricted Eating for Weight Loss and Metabolic Health: a Secondary Per-Protocol Analysis. 2022;30doi:10.1002/oby.23626

3. Jamshed H, Steger FL, Bryan DR, et al. Effectiveness of Early Time-Restricted Eating for Weight Loss, Fat Loss, and Cardiometabolic Health in Adults With Obesity: A Randomized Clinical Trial. *JAMA Internal Medicine*. 2022;182(9)doi:10.1001/jamainternmed.2022.3050

4. Steger F, Jamshed H, Bryan D, et al. Effect of time-restricted eating on weight, fat loss and cardiometabolic risk in adults with obesity. 2020;28(SUPPL 2)doi:10.1002/oby.23057

5. Steger FL, Jamshed H, Bryan DR, et al. Early time-restricted eating affects weight, metabolic health, mood, and sleep in adherent completers: a secondary analysis. 2023;31 Suppl 1(Suppl 1)doi:10.1002/oby.23614

6. Steger FL, Jamshed H, Martin CK, et al. Impact of early time-restricted eating on diet quality, meal frequency, appetite, and eating behaviors: a randomized trial. 2023;31 Suppl 1(Suppl 1)doi:10.1002/oby.23642

Kunduraci 2020

1. Kunduraci YE, Ozbek H. Does the Energy Restriction Intermittent Fasting Diet Alleviate Metabolic Syndrome Biomarkers? A Randomized Controlled Trial. *Nutrients*. 2020;12(10)doi:10.3390/nu12103213

Lin 2023

1. Lin S, Cienfuegos S, Ezpeleta M, et al. Time-Restricted Eating Without Calorie Counting for Weight Loss in a Racially Diverse Population: A Randomized Controlled Trial. *Annals of Internal Medicine*. 2023;176(7)doi:10.7326/M23-0052

Liu 2022

1. Liu D, Huang Y, Huang C, et al. Calorie Restriction with or without Time-Restricted Eating in Weight Loss. *New England Journal of Medicine*. 2022;386(16)doi:10.1056/NEJMoa2114833

Lowe 2020

1. Lowe DA, Wu N, Rohdin-Bibby L, et al. Effects of Time-Restricted Eating on Weight Loss and Other Metabolic Parameters in Women and Men With Overweight and Obesity: The TREAT Randomized Clinical Trial. *JAMA Internal Medicine*. 2020;180(11)doi:10.1001/jamainternmed.2020.4153

Manoogian 2022

1. Manoogian ENC, Zadourian A, Lo HC, et al. Feasibility of time-restricted eating and impacts on cardiometabolic health in 24-h shift workers: The Healthy Heroes randomized control trial. *Cell Metabolism*. 2022;34(10)doi:10.1016/j.cmet.2022.08.018

Montero 2023

1. Montero MD. *Effects of three 8-hour time-restricted eating schedules on visceral adipose tissue, body composition and cardiometabolic health in men and women with overweight/obesity: A multicenter randomized controlled trial*. University of Granada; 2023. https://digibug.ugr.es/bitstream/handle/10481/84704/89159.pdf?sequence=4&isAllowed=y

© 2024 Liu HY et al. *JAMA Network Open.*

Pavlou 2023	1. Pavlou V, Cienfuegos S, Lin S, et al. Effect of Time-Restricted Eating on Weight Loss in Adults With Type 2 Diabetes: A Randomized Clinical Trial. *JAMA Netw Open*. Oct 2 2023;6(10):e2339337. doi:10.1001/jamanetworkopen.2023.39337
Philips 2021	1. Papageorgiou M, Biver E, Mareschal J, et al. The Effects Of Time-Restricted Eating (Tre) And Weight Loss On Bone Metabolism And Health: an Exploratory Analysis In A 6-Month Randomised Controlled Trial. 2023;54doi:10.1016/j.clnesp.2022.09.296<br>2. Papageorgiou M, Biver E, Mareschal J, et al. The effects of time-restricted eating and weight loss on bone metabolism and health: a 6-month randomized controlled trial. 2023;31 Suppl 1(Suppl 1)doi:10.1002/oby.23577<br>3. Phillips NE, Mareschal J, Schwab N, et al. The Effects of Time-Restricted Eating versus Standard Dietary Advice on Weight, Metabolic Health and the Consumption of Processed Food: A Pragmatic Randomised Controlled Trial in Community-Based Adults. *Nutrients*. 2021;13(3)doi:10.3390/nu13031042
Roman 2020	1. Roman SN, Fitzgerald KC, Beier M, Mowry EM. Safety and feasibility of various fasting-mimicking diets among people with multiple sclerosis. *Multiple Sclerosis and Related Disorders*. 2020;42doi:10.1016/j.msard.2020.102149
Suthutvoravut 2023	1. Suthutvoravut U, Anothaisintawee T, Boonmanunt S, et al. Efficacy of Time-Restricted Eating and Behavioral Economic Intervention in Reducing Fasting Plasma Glucose, HbA1c, and Cardiometabolic Risk Factors in Patients with Impaired Fasting Glucose: A Randomized Controlled Trial. 2023;15(19). doi:10.3390/nu15194233
Thomas 2022	1. Thomas EA, Zaman A, Sloggett KJ, et al. Early time-restricted eating compared with daily caloric restriction: A randomized trial in adults with obesity. *Obesity (19307381)*. 2022;30(5)doi:10.1002/oby.23420<br>2. Zaman A, Grau L, Jeffers R, et al. The effects of early time restricted eating plus daily caloric restriction compared to daily caloric restriction alone on continuous glucose levels. *Obesity Science and Practice*. 2023;doi:10.1002/osp4.702
Wei 2023	1. Wei X, Lin B, Huang Y, et al. Effects of Time-Restricted Eating on Nonalcoholic Fatty Liver Disease: The TREATY-FLD Randomized Clinical Trial. *JAMA Network Open*. 2023;6(3)doi:10.1001/jamanetworkopen.2023.3513
Bachman 2012	1. Bachman JL, Raynor HA. Effects of manipulating eating frequency during a behavioral weight loss intervention: a pilot randomized controlled trial. *Obesity (Silver Spring, Md)*. 2012;20(5)doi:https://dx.doi.org/10.1038/oby.2011.360
Forslund 2008	1. Forslund HB, Klingström S, Hagberg H, Löndahl M, Torgerson JS, Lindroos AK. Should snacks be recommended in obesity treatment? A 1-year randomized clinical trial. *European Journal of Clinical Nutrition*. 2008;62(11)doi:10.1038/sj.ejcn.1602860
Grangeiro 2021	1. Grangeiro É, Trigueiro MS, Siais LO, et al. Hypocaloric diet with lower meal frequency did not affect weight loss, body composition and insulin responsiveness, but improved lipid profile: a randomized clinical trial. 2021;12(24)doi:10.1039/d1fo00484k

© 2024 Liu HY et al. *JAMA Network Open.*

Jakubowicz 2019	1. Jakubowicz D, Froy O, Tsameret S, et al. Three meals diet with high energy breakfast is an effective strategy for weight loss, reduction of glucose variability and of total daily insulin dose in type 2 diabetes. 2018;61doi:10.1007/s00125-018-4693-0<br><br>2. Jakubowicz D, Froy O, Tsameret S, et al. High energy breakfast diet is an effective strategy for weight loss and reduction of the total daily insulin dose in type 2 diabetes. 2018;39(2)<br><br>3. Jakubowicz D, Landau Z, Tsameret S, et al. Reduction in Glycated Hemoglobin and Daily Insulin Dose Alongside Circadian Clock Upregulation in Patients With Type 2 Diabetes Consuming a Three-Meal Diet: A Randomized Clinical Trial. *Diabetes Care*. 2019;42(12)doi:10.2337/dc19-1142
Kahleova 2014	1. Belinova L, Kahleova H, Hajek M, Dezortova M, Hill M, Pelikanova T. The effect of frequency of meals on hepatic fat content in patients with type 2 diabetes. *Diabetologia*. 2012;55doi:10.1007/s00125-012-2688-9<br><br>2. Belinova L, Kahleova H, Malinska H, et al. The effect of meal frequency in a reduced-energy regimen on the gastrointestinal and appetite hormones in patients with type 2 diabetes: a randomised crossover study. 2017;12(4)doi:10.1371/journal.pone.0174820<br><br>3. Kahleova H, Belinova L, Hajek M, Dezortova M, Hill M, Pelikanova T. Prolonged fasting reduces hepatic fat content in patients with type 2 diabetes. *Diabetes*. 2012;61doi:10.2337/db12-2763-2907<br><br>4. Kahleova H, Belinova L, Hill M, Pelikanova T. The effect of frequency of meals on body weight, HbA1c and resting energy expenditure in patients with type 2 diabetes. *Diabetologia*. 2012;55doi:10.1007/s00125-012-2688-9<br><br>5. Kahleova H, Belinova L, Hill M, Pelikanova T. Two meals a day are better than six for patients with type 2 diabetes. *Diabetes*. 2012;61doi:10.2337/db12-656-835<br><br>6. Kahleova H, Belinova L, Malinska H, et al. Meal frequency and type 2 diabetes. 2015;8doi:10.1159/000382140<br><br>7. Kahleova H, Belinova L, Malinska H, et al. Eating two larger meals a day (breakfast and lunch) is more effective than six smaller meals in a reduced-energy regimen for patients with type 2 diabetes: a randomised crossover study. 2014;57(8)doi:10.1007/s00125-014-3253-5<br><br>8. Kahleova H, Belinova L, Tura A, et al. The effect of frequency of meals on beta cell function in subjects with type 2 diabetes. *Diabetologia*. 2013;56doi:10.1007/s00125-013-3012-z<br><br>9. Kahleova H, Malinska H, Kazdova L, Belinova L, Hill M, Pelikanova T. The effect of meal frequency on fatty acid composition in serum phospholipids in patients with type 2 diabetes. *Diabetologia*. 2014;57(1)doi:10.1007/s00125-014-3355-0
Papakonstantinou 2016	1. Papakonstantinou E, Kechribari I, Mitrou P, et al. Effect of meal frequency on glucose levels in women with polycystic ovary syndrome: a randomized trial. 2015;8doi:10.1159/000382140

	2. Papakonstantinou E, Kechribari I, Mitrou P, et al. Effect of meal frequency on glucose and insulin levels in women with polycystic ovary syndrome: a randomised trial. 2016;70(5)doi:10.1038/ejcn.2015.225
Papakonstantinou 2018	1. Papakonstantinou E, Kontogianni MD, Mitrou P, et al. Effects of 6 vs 3 eucaloric meal patterns on glycaemic control and satiety in people with impaired glucose tolerance or overt type 2 diabetes: A randomized trial. *Diabetes Metab*. Jun 2018;44(3):226-234. doi:10.1016/j.diabet.2018.03.008<br><br>2. Papakonstantinou E, Mitrou P, Kontogianni MD, Dimitriadis G. Effect of meal frequency on glucose and insulin responses in obese people with impaired glucose tolerance and with type 2 diabetes: a randomised trial. 2017;60(1)doi:10.1007/s00125-017-4350-z
Zargaran 2014	1. Zargaran ZH, Salehi M, Taghi Heydari S, Babajafari S. The effects of 6 Isocaloric Meals on Body Weight, Lipid Profiles, Leptin, and Adiponectin in Overweight Subjects (BMI > 25). 2014;8(2)
Jakubowicz 2013	1. Jakubowicz D, Bar Dayan Y, Wainstein J. Differential effect of high calorie breakfast vs. high calorie dinner diet on weight loss and glucose metabolism in women with metabolic syndrome. 2013;5doi:10.1111/1753-0407.12032<br><br>2. Jakubowicz D, Barnea M, Wainstein J, Froy O. High caloric intake at breakfast vs. dinner differentially influences weight loss of overweight and obese women. 2013;21(12)doi:10.1002/oby.20460<br><br>3. Jakubowicz D, Boaz M, Bar-Dayan Y, Wainstein J. Comparison of the effect of high calorie breakfast diet vs high calorie dinner diet on weight loss, ghrelin, lipids and appetite scores in obese non diabetic women. 2012;33(3)<br><br>4. Jakubowicz D, Wainstein J, Froy O. High-calorie breakfast improves weight loss and metabolism vs. isocaloric meal at dinner in obese women with metabolic syndrome. 2013;62doi:10.2337/db13-1395-1677
Lombardo 2014	1. Lombardo M, Bellia A, Padua E, et al. Morning meal more efficient for fat loss in a 3-month lifestyle intervention. *J Am Coll Nutr*. 2014;33(3):198-205. doi:10.1080/07315724.2013.863169
Madjd 2016	1. Madjd A, Taylor MA, Delavari A, Malekzadeh R, Macdonald IA, Farshchi HR. Beneficial effect of high energy intake at lunch rather than dinner on weight loss in healthy obese women in a weight-loss program: a randomized clinical trial. *Am J Clin Nutr*. Oct 2016;104(4):982-989. doi:10.3945/ajcn.116.134163
Madjd 2021	1. Madjd A, Taylor MA, Delavari A, Malekzadeh R, Macdonald IA, Farshchi HR. Effects of consuming later evening meal v. earlier evening meal on weight loss during a weight loss diet: a randomised clinical trial. *Br J Nutr*. Aug 28 2021;126(4):632-640. doi:10.1017/s0007114520004456

© 2024 Liu HY et al. *JAMA Network Open.*

eTable 4: Details of Intervention Delivery

Author, year	Instructions given	Energy/macro-nutrient prescription	Personalisation	Intervention maintenance	Frequency of contact	Mode of delivery	Intervention provider
**Time-restricted eating**							
**Che 2021[33]**	C: Usual diet, no time restriction<br><br>I_1: 10h TRE, 8 am - 6 pm<br><br>Guidance on how to estimate portion sizes and keep detailed food records to obtain accurate dietary intake	Ad libitum	None	Supervisor meetings	Weekly	Face-to-face	Research supervisor<br><br>Nutritionist
**Chow 2020[34]**	C: Usual diet, no time restriction<br><br>I1: 8h TRE (self-selected)	Ad libitum	None	Phone call, email or text reminders	Weekly	Online	Not stated
**de Oliveira Maranhao Pureza 2021[35]**	C: Daily energy restriction<br><br>I_1: 12h TRE (self-selected eating window)	Energy restriction	Energy goal: 500-1000 kcal subtracted from total energy expenditure<br><br>Meal plan based on Brazilian	Nutrition counselling	Monthly	Face-to-face	Dietitian

© 2024 Liu HY et al. *JAMA Network Open.*

			guideline on obesity and habitual food consumption				
**Dhurandhar 2014**[36]	C: General good nutrition habits<br>I_1: Ate breakfast before 10 am<br>I_2: No energy intake before 11 am<br>Received a USDA pamphlet “Let’s Eat for the Health of It” and a summary handout	None	None	Phone calls	Weeks 4, 8 and 12	Hybrid	Study coordinators
**Erdem 2022**[29]	I_1: 6h TRE (1-7 pm)<br>I_2: 8h TRE (1-9 pm)<br>Initial visit: portion size training	Ad libitum	None	None	/	Face-to-face	Researcher
**Jamshed 2022**[40]	C: Self-selected ≥12h eating window<br>I_1: 8h TRE (7 am - 3 pm)	Energy restriction = REE – 500kcal/day	None	Nutrition counselling<br>Group classes	Baseline, weeks 2, 6 and 10<br>≥3 times a month	Face-to-face<br>Hybrid	Dietitian

© 2024 Liu HY et al. *JAMA Network Open.*

	Encouraged to increased exercise to 75-150 minutes/week						
**Kunduraci 2020**[42]	C: Daily energy restriction<br>I_1: 8h TRE (Self-selected eating window)<br>Instructions to fill out food diaries and fasting log given	Energy restriction	Energy goal: 25% reduction from habitual energy intake<br>Macro- and micro-nutrient distribution<br>Meal plan based on Turkey National Dietary Guidelines (Mediterranean Diet)	Phone calls and compliance interviews	Weekly	Hybrid	Clinician & Dietitian
**Lin 2023**[15]	C: Maintained weight, physical activity habits & 10h+ baseline eating window<br>I_1: 8h TRE (12-8 pm). At 26 weeks, eating window widened to 10h	Ad libitum	None	Nutrition counselling	Weekly for first 12 weeks<br>Fortnightly for next 12 weeks<br>Monthly for the final 6 months	Online	Dietitian
**Liu 2022**[43]	C: No time restriction	Energy restriction = 1500-1800	None	Phone calls/app messages	Twice a week then monthly in	Hybrid	Trained health coach

© 2024 Liu HY et al. *JAMA Network Open.*

	I$_1$: 8h TRE (8am - 4pm)	kcal/d for men & 1			the last 6 months		
	Encouraged to weigh food to ensure accuracy of intake	200-1500 kcal/d for women		Nutrition counselling	Fortnightly then monthly in the last 6 months		
	1 protein shake per day for first 6 months	Macronutrient distribution (40-55% carbohydrate, 15-20% protein, 20-30% fat)		Health education sessions	Monthly		
	Written dietary information booklets with food portion advice and sample menus						
	Mobile app for daily food log with pictures and mealtimes						
**Lowe 2020**[45]	C: 3 meals daily (7-11 am, 11 am-3 pm, 4-10 pm) Snacking between meals was permitted.	Ad libitum	None	App reminders	Three times a day	Remote	Not stated

© 2024 Liu HY et al. *JAMA Network Open.*

	I_1: 8h TRE (12-8 pm)<br><br>Mobile app with Bluetooth scale connected for weight and blood pressure measurements, surveys and message reminders						
**Manoogian 2022[46]**	C: No time restriction	Ad libitum	None	App reminders	Not stated	Remote	Dietitian
	I_1: 10h TRE (self-selected eating window)<br><br>Mediterranean Diet cookbook<br><br>Mobile app for food log	Mediterranean Diet (60% carbohydrates, 15% protein and 25% fat)		Nutrition counselling	Week 6		
**Montero 2023[18]**	Received an educational program<br><br>C: Usual care	No energy restriction	None	None	N/A	N/A	N/A

© 2024 Liu HY et al. *JAMA Network Open.*

	I1: 8h TRE (starting by 10:00am)<br>I2: 8h TRE (starting by 12:00pm)<br>I3: 8h TRE (self-selected eating window)	Education on Mediterranean diet					
**Pavlou 2023[19]**	Instructed to maintain their weight ad usual eating and exercise habits<br>C: Usual care<br>I1:8h TRE (Ad libitum 12-8pm)	No energy restriction<br>General healthy eating instructions	None	Recorded adherence, weight changes, etc. TRE groups were taught about healthy food choices.	Weekly until 3 months, bi-weekly thereafter.	Telephone/zoom	Dietitian
**Philips 2021[49]**	C: Usual care (10-minute nutritional counselling at randomisation and a leaflet summarising food pyramid and Swiss dietary advice)<br>I_1: 12h TRE (self-selected eating window with no nutritional advice)	Ad libitum	None	Phone calls and email	After 2 weeks of observation, and after 2 and 4 months of intervention	Remote	Not stated

© 2024 Liu HY et al. *JAMA Network Open.*

	Mobile app for food log						
**Roman 2020[50]**	C: Usual diet<br>I_1: 8h TRE (self-selected eating window)	Ad libitum	None	SMS message	Twice a week	Remote	Not stated
**Suthutvoravut 2023[20]**	C: Usual care<br>I_1: 9h TRE (8 am - 5 pm)	Ad libitum	None	None	/	/	Not stated
**Thomas 2022[51]**	C: No time restriction<br>I_1: 10h TRE (starting within 3 h of waking)<br>Encouraged to perform 150min/week of moderate activity	Energy restriction = REE - 10%	Energy goal	Group-based program based on PreventT2 curriculum	Weekly first 12 weeks then monthly during weeks 13-39	Cohort 1 & 2: Face-to-face<br>Cohort 3: Face-to-face then remote from week 6 due to COVID	Dietitian
**Wei 2023[21]**	C: No time restriction<br>I_1: 8h TRE (8 am - 4 pm)<br>Encouraged to weigh food to	Energy restriction = 1500-1800 kcal/d for men & 1200-1500 kcal/d for women<br>Macronutrient composition	Energy goal	Phone calls/app messages<br>Nutrition counselling	Twice a week then monthly in the last 6 months	Hybrid	Trained nutritionist

© 2024 Liu HY et al. *JAMA Network Open.*

	ensure accuracy of intake	(40% to 55% carbohydrate, 15% to 20% protein, and 20% to 30% fat)			Fortnightly then monthly in the last 6 months		
	1 protein shake per day for first 6 months			Health education sessions	Monthly		
	Written dietary information booklets with food portion advice and sample menus						
	Mobile app for daily dietary log with pictures and mealtimes						
**Meal Frequency**							
**Bachman 2012[32]**	I_1: 3 meals per day<br>I_2: Grazing group - ≥100kcal every 2-3 h. Distribution of energy recommended to follow typical diet for Americans (75% energy from	Isocaloric energy and fat restriction	Encouraged to decide how they wanted to divide their energy intake between the 3 meals.<br><br>Guidelines in form of meal plan provided. Asked	Meetings<br><br>At each meeting, participants were weighed, homework was discussed, and a behavioural	Weekly for 4 months then bimonthly for remaining 2 months	Face-to-face	Study principle investigator (Masters level training in nutrition, registered dietitian, and training in exercise physiology and

© 2024 Liu HY et al. *JAMA Network Open.*

	meals). Provided a list of snack ideas.<br><br>Participants with a body weight ≤200 lbs were prescribed 1,200 kcals/day and participants with body weight >200 lbs were prescribed 1,500 kcals/ day. Intake of fat was restricted to <30% energy from fat. Encouraged to build up physical activity.		to create individual eating schedule, schedules reviewed and adjusted if needed.	lesson. Self-monitoring records were turned in at each meeting and individualized feedback on program goals was provided.			behavioural psychology)
**Forslund 2008**[37]	**I_1:** 3 meals per day.<br><br>Recommended daily energy distribution: 30% Breakfast, 35% Lunch, 35% Dinner.<br><br>**I_2:** 3 snacks + 3 meals per day.	Isocaloric energy restriction	Instruction about eating frequency and individualized diet plan provided.<br><br>Energy needs calculated; participants given personal energy restriction 30% less than needs.	Meetings	Every 2 weeks for first 12 weeks then every four weeks until week 52	Face-to-face?	Local dietician, physician and nurse

© 2024 Liu HY et al. *JAMA Network Open.*

	Recommended daily energy distribution: 20% Breakfast, 25% lunch, 25% dinner + 3 snacks 10% each		All diets followed the Swedish Nutrition Recommendation s				
**Grangeiro 2021**[14]	I_1: 6 meal diet<br>I_2: 3 meal diet<br><br>Hypocaloric diet (decreased from TEE by ~700kcal/day) consisting of six daily meals: breakfast (15%TEE), snack (5%–10% TEE), lunch (25%TEE), snack (5%–10% TEE), dinner (25%TEE) and evening snack (5%–10% TEE) OR<br>consisting of three daily	Isocaloric energy restriction	The dietary prescription was devised by trained nutritionists and the diets were calculated a<br>software.	Initial meeting to teach use of dietary records, second meeting for assessments.<br><br>Diet compliance measured through self-reported food record (assessed during 3 individual meetings). Dietary registration forms reviewed for accuracy.	Carried out until week 12	? Face-to-face?	Nutritionists

© 2024 Liu HY et al. *JAMA Network Open.*

	meals: breakfast (20%-25%TEE), lunch (35%–40% TEE), and<br>dinner (35%–40% TEE)						
**Jakubowicz 2019**[39]	I_1: 3 meal diet<br>I_2: 6 meal diet<br>3 meal diet consisted of a large breakfast of 700 kcal, a medium-sized lunch of 600 kcal, and a small dinner of 200 kcal; 6 meal diet consisted of six meals (breakfast, lunch, dinner, and three snacks) with relatively uniform daily caloric distribution in the meals plus 150 kcal in each one of the three snacks.	Isocaloric energy restriction	Isocaloric energy restriction by subtracting 500 kcal from the individual calculated Harris Benedict equation.	Personal dietary counselling by dietitian	Every 2 weeks	Face-to-face?	Dietitian
				10-15 minute conversation	Twice a week	Phone	
				Appointment for insulin titration	Biweekly	Face-to-face?	Physician

© 2024 Liu HY et al. *JAMA Network Open.*

	All participants were asked to eat breakfast before 0930, lunch between 1200-1500, and dinner between 1800-2000. Additional three snacks at 1100, 1700, and 2200.<br><br>For both - same macronutrient composition of fat, protein, and carbohydrates (35:25:40%, respectively)						
**Kahleova 2014**[41]	I_1: 6 meal diet<br>(3 main meals (breakfast, lunch, dinner) + 3 smaller snacks in between)	Isocaloric energy restriction	Restriction of 500 kcal/day from resting energy expenditure (REE) of individuals measured by indirect calorimetry	Four day diet tutorial with follow up meetings (lectures + cooking classes)	Initial tutorial followed by 1 hour weekly for entire study duration	Face-to-face	Unspecified Dietitian assessed dietary records
				Diet records			

© 2024 Liu HY et al. *JAMA Network Open.*

	I_2: 2 meal diet (Breakfast and Lunch)<br><br>Followed Diabetes and Nutrition of the European Association for the Study of Diabetes guidelines.<br><br>Total daily energy breakdown: 50–55% carbohydrates, 20–25% protein and less than 30% fat with 30–40 grams of fibre.<br><br>Meals provided for a randomised ½ of study participants.				Weeks 0, 12 and 24 (3-day record)		
**Papakonstantinou 2016**[47]	I_1: 3 meal diet<br>I_2: 6 meal diet	Energy maintenance	Instructions provided on how to record food	Energy intake records	Daily	Face-to-face??	Participants kept food records

© 2024 Liu HY et al. *JAMA Network Open.*

Macronutrient breakdown: 40% carbohydrates, 25% protein and 35% fat.		Monitored and adjusted food records	Biweekly	Dietitians checked food records
Three-meal pattern carbohydrate distribution pattern was 20% at breakfast, 50% at lunch and 30% at dinner.				
The six-meal carbohydrate breakdown was 20% at breakfast, 10% at morning snack, 30% at lunch, 10% at afternoon snack, 20% at dinner and 10% at before bedtime snack.				

© 2024 Liu HY et al. *JAMA Network Open.*

**Papakonstantinou 2018a**[48]	I_1: 3 meal diet<br><br>I_2: 6 meal diet<br><br>Macronutrient breakdown: 45% carbohydrates, 20% protein and 35% fat.<br><br>Three-meal pattern carbohydrate distribution pattern was 20% at breakfast, 50% at lunch and 30% at dinner.<br><br>The six-meal carbohydrate breakdown was 20% at breakfast, 10% at morning snack, 30% at lunch, 10% at afternoon snack,	Energy maintenance<br><br>Dietary plans provided with guidance foods to eat and meal preparation.<br><br>Caloric requirements calculated using Schofield equation.	For each 12-week intervention, five 7-day food diaries were used to check compliance with the dietary plan. Detailed instructions were given on how to record the quantity of food consumed, using standard household weights and measures.	Food records daily<br><br>Education sessions and structured interviews where changes were proposed	Reviewed every 2-weeks	??	Kept by participants, reviewed by dietitian

© 2024 Liu HY et al. *JAMA Network Open.*

	20% at dinner and 10% at bedtime<br><br>snack.						
**Papakonstantinou 2018b**[48]	I_1: 3 meal dietI_2: 6 meal diet<br><br>Macronutrient breakdown: 45% carbohydrates, 20% protein and<br><br>35% fat.<br><br>Three-meal pattern carbohydrate distribution<br><br>pattern was 20% at breakfast, 50% at lunch and 30% at dinner.<br><br>The six-meal carbohydrate breakdown was 20% at breakfast, 10% at morning	Energy maintenance<br><br>Dietary plans provided with guidance foods to eat and meal preparation.<br><br>Caloric requirements calculated using Schofield equation.	For each 12-week intervention, five 7-day food diaries were used to check compliance with the dietary plan. Detailed instructions were given on how to record the quantity of food consumed, using standard household weights and measures.	Food records daily<br><br>Education sessions and structured interviews where changes were proposed	Reviewed every 2-weeks	??	Kept by participants, reviewed by dietitian

© 2024 Liu HY et al. *JAMA Network Open.*

	snack, 30% at lunch, 10% at afternoon snack, 20% at dinner and 10% at bedtime snack.						
**Zargaran 2014[52]**	C: Normal diet (mostly three meals and two snacks) I$_1$:6 meal diet (iso-caloric meals) The control group was trained only about healthy nutrition in 2 sessions. 6 iso-caloric meals, energy is divided into six equal parts (one meal every three hours), instead of their previous meal pattern. The intervention	Energy restriction	Weight loss diet written for both groups. Energy intake planned for each subject was 400 kcal less energy needed for weight maintenance. Macronutrient percentage was the same in both groups.	Training sessions	2 sessions (control), 4 sessions (intervention)	N/A	N/A

© 2024 Liu HY et al. *JAMA Network Open.*

	group was trained on how to divide daily energy into 6 isocaloric meals in 4 sessions.						
**Meal Distribution**							
**Jakubowicz 2013**[38]	**I1:** High-calorie breakfast with a small dinner<br><br>I2: high-calorie dinner (D) with a small breakfast<br><br>Meal times: Breakfast: 6:00-9:00, lunch: 12:00-15:00, dinner: 18:00-21:00.	Total daily energy of 1400 +/- 25 kcal with identical macronutrient content and composition between groups.<br><br>BF meal plan: a large breakfast (700 kcal, 50%), medium-sized lunch (500 kcal, 36%), and a small dinner (200 kcal, 14%)<br><br>Dinner plan was opposite above	Meal plans provided and proper food replacement choices for each item allowed for variation	Weekly 3-day food record<br><br>Bi-weekly Nutrition consults	Weekly	Face-to-face	Research supervisor, Dietitian

© 2024 Liu HY et al. *JAMA Network Open.*

**Lombardo 2014**[44]	C: Even energy distribution<br>55% energy breakfast to lunch, 45% energy afternoon tea to dinner)<br><br>I1: Front loading energy<br>70% energy breakfast to lunch, 30% energy afternoon tea to dinner	Ad libitum	Mediterranean-style diet tailored to individual	3-day diet diary each week<br><br>Weekly nutrition counselling<br><br>As needed phone support consultation service	Weekly	Face-to-Face, Phone	Dietitian
**Madjd 2016**[16]	I1: Large Lunch group: Energy distribution 15% breakfast 15% snacks, 50% lunch, 20% dinner<br><br>I2: Large Dinner group<br>Energy distribution same as above with lunch (20%) and	Hypoenergetic diet designed for 7-10% weight loss over 12 weeks<br><br>17% of energy from protein, 23% from fat (10% from saturated fat), and 60% from carbohydrate,	Individualised diet programs in line with food record and preferences with gradual modification to NovinDiet protocol	Nutrition counselling<br><br>Nutrition resources (booklets)<br><br>Access to a website, weekly internet magazines	Bi-weekly	Phone, Face-to-Face	Dietitian, physician

© 2024 Liu HY et al. *JAMA Network Open.*

	dinner (50%) swapped	with 400 g fruit and vegetables		Physician support			
**Madjd 2021**[17]	I1: Early dinner group<br><br>Evening meal between 7 pm-7:30 pm<br><br>I2: Late Dinner group<br><br>Evening meal between 10:30 pm – 11 pm	2092–2184 kJ (500–1000 kcal) energy deficit based on EER<br><br>Energy distribution both groups<br><br>15 % breakfast 15% snacks,<br><br>50 % lunch,<br><br>20 % dinner<br><br>17% of energy from protein, 23 % from fat (<10 % from saturated fat) and 60 % from carbohydrate – with at least 400 g/d fruits and vegetables	Individualised diet program based on food diary and preferences	Nutrition counselling	Bi-Weekly	Phone, Face-to-Face	Dietitian

© 2024 Liu HY et al. *JAMA Network Open.*

eTable 5: Adherence outcomes

Study	Definition	Mean (SD)
Time-restricted eating		
**Che 2021**	Daily self-reported eating window adherence (no. of days/week)	I_1: 6.59 (0.95)
**Chow 2020**	(a) Daily self-reported logging adherence<br>(b) Eating window compliance within ±15 minutes, ±30 minutes, and ±60 minutes via myCircadianClock (mCC) app over 12 weeks	C: (a) 90.9% (8.3)<br>I_1: (a) 83.1% (13.4)<br>(b) ±15 minutes: 55.5% (22.4)<br>±30 minutes: 60% (23)<br>±60 minutes: 66.3% (20.7)
**Dhurandhar 2014**	Self-reported percentage of days complying with the breakfast skipping recommendation over 16 weeks	I: 92.40%
**Jamshed 2022**	Daily self-reported eating window adherence (no. of days/week) via REDCap (Research Electronic Data Capture) surveys	C: 6.3 (0.8)<br>I_1: 6.0 (0.8)
**Lin 2023**	Control: Percentage of participants adhering to energy restriction from food record data<br>TRE: Daily self-reported eating window adherence via app (no. of days/week)	C: 61%<br>I_1: 6.1 (0.8)
**Liu 2022**	Mean percentage of days adhering to the assigned diet assessed from daily dietary log, food pictures, and the eating window noted via app over 12 months	C: 83.8% (12.6)<br>I_1: 84.0% (16.1)
**Lowe 2020**	Percentage of days adhering to protocol reported on daily adherence surveys	C: 92.10%<br>I_1: 83.50%
**Manoogian 2022**	Logging adherence (number of days out of 14 days) via myCircadianClock (mCC) app over 12 weeks	C: 10.07 (2.67)<br>I_1: 10.23 (2.71)
**Roman 2020**	Self-reported percentage of study days with a calorie intake interval (CII) <8 hours duration	C: 29.7% (13.87)<br>I_1: 64.61% (19.80)
**Thomas 2022**	Self-reported adherence (no. of days/week) via questionnaires over 12 weeks<br>(a) Energy restriction<br>(b) Eating window	C: (a) 3.9 (1.5)<br>I_1: (a) 4.4 (1.6)<br>(b) 5.5 (1.2)
**Wei 2023**	Percentage of days adhering to the diet program via dietary log, food pictures and mealtimes via app over 12 months	C: 85.0% (10.7)<br>I_1: 85.7% (9.4)

© 2024 Liu HY et al. *JAMA Network Open.*

Meal frequency		
**Bachman 2012**	(a) Percentage of self-monitoring diaries submitted<br>(b) Percentage of attendance	I_1:<br>(a) 78.8% (23.4)<br>(b) 91.2% (14.1)<br>I_2:<br>(a) 69.2% (31.1)<br>(b) 81.2% (23.0)
**Grangeiro 2021**	Average percentage of stipulated meals consumed assessed from the periodic food record assessed three times over 12 weeks	I_1: 76.80% (16.96)<br>I_2: 70.81% (16.78)
Meal distribution		
**Jakubowicz 2013**	Self-reported weekly three-day diet record<br>Noncompliance: a deviation of 10% or more from the recommended energy intake	Higher compliance in BF group than D group
**Madjd 2021**	Percentage of days following dinner time recommendation from self-reported diary log	I_1: 91.80%<br>I_2: 93.20%

Note. eTable 5 details the reported mean (SD) for adherence to the prescribed intervention over the duration of the study based on how each study reported adherence. SD = Standard deviation, C = control group, I_1 = first intervention group, I_2 = second intervention group, BF = Breakfast, D = Dinner

eTable 6: Hunger outcomes

Study	Measuring tool	Timepoints extracted	Mean (SD)
Time-restricted eating			
**Jamshed 2022 (Steger 2023)**	VAS (0-100 mm)	Baseline & 14 weeks	C: -18 (38.34)<br>I_1: -3 (53.85)
**Thomas 2022**	VAS (0-100 mm)	Baseline & 12 weeks	C: 0.3 (4.27)<br>I_1: -2.5 (3.75)
	TFEQ (0-14)	Baseline & 12 weeks	C: -1.1 (0.89)<br>I_1: -1.0 (0.84)
Meal frequency			
**Bachman 2012**	VAS (0-100 mm)	Baseline & 26 weeks	I_1: no significant difference<br>I_2: significant decrease ($p<0.05$)
**Jakubowicz 2019**	VAS (0-100 mm)	Baseline & 12 weeks	I_1: -18 (3)

© 2024 Liu HY et al. *JAMA Network Open.*

			I_2: 2 (1.7)
**Papakonstantinou 2016**	VAS (0-10)	Biweekly average for 12 weeks	I_1: 3.1 (0.35)<br>I_2: 2.3 (0.30)
**Papakonstantinou 2018**	VAS (0-10)	Biweekly average for 12 weeks	I_1: 3.30 (2.12)<br>I_2: 2.54 (1.65)
Meal distribution			
**Jakubowicz 2013**	VAS (0-100 mm)	Week 2	Mean daily hunger levels in BF group were 28% lower than D group (p<0.0001)

Note. eTable 6 details the reported mean (SD) for hunger as measured using the detailed measuring tool. SD = Standard deviation, C = control group, I_1 = first intervention group, I_2 = second intervention group

eTable 7: Satiety outcomes

**Study**	**Measuring tool**	**Timepoints extracted**	**Mean (SD)**
Time-restricted eating			
**Jamshed 2022 (Steger 2023)**	VAS (0-100 mm)	Baseline & 14 weeks	C: -20 (38.34)<br>I_1: -6 (32.31)
**Thomas 2022**	VAS (0-100 mm)	Baseline & 12 weeks	C: -0.3 (5.24)<br>I_1: -2.4 (4.5)
Meal frequency			
**Papakonstantinou 2016**	VAS (0-10)	Biweekly average	I_1: 8.0 (0.22)<br>I_2: 8.3 (0.24)
**Papakonstantinou 2018**	VAS (0-10)	Biweekly average	I_1: 7.84 (1.58)<br>I_2: 7.72 (1.99)
Meal distribution			
**Jakubowicz 2013**	VAS (0-100 mm)	Week 2	Mean daily satiety levels in BF group were 31% higher than D group (p<0.0001)

Note. eTable 7 details the reported mean (SD) for satiety as measured using the detailed measuring tool. SD = Standard deviation, C = control group, I_1 = first intervention group, I_2 = second intervention group

eTable 8: Notable clinical trials excluded

Clinical Trial Number	Intervention	Year	Stage of completion	Notes
**ACTRN12620000453987**	Time-restricted eating	2020	Completed	Full text not found<br>No anthropometric measurements

© 2024 Liu HY et al. *JAMA Network Open.*

**ACTRN12613000935730**	Time-restricted eating	2013	Completed	Full text found<br>Wrong comparator
**ChiCTR2000032048**	Time-restricted eating	2020	Not yet recruiting	Full text not found
**ChiCTR1800016271**	Intermittent fasting	2018	Recruiting	Full text not found
**ChiCTR2200058929**	Breakfast skipping	2022	Not yet recruiting	Full text not found
**ChiCTR2100052582**	Time-restricted eating	2021	Recruiting	Full text not found
**ChiCTR2100052876**	Time-restricted eating	2021	Recruiting	Full text not found
**ChiCTR2100051792**	Time-restricted eating	2021	Recruitment completed	Full text not found
**ChiCTR2000029797**	Time-restricted eating	2020	Completed	Full text found<br>Wrong intervention duration
**ChiCTR1800016104**	Intermittent fasting	2018	Not yet recruiting	Full text not found
**ChiCTR1900025424**	Intermittent fasting	2019	Not approved	Full text not found
**ChiCTR2000039115**	Time-restricted eating	2020	Recruiting	Wrong intervention duration
**ChiCTR2200056363**	Time-restricted eating	2022	Not yet recruiting	Full text not found
**ChiCTR2300067375**	Time-restricted eating	2023	Not yet recruiting	Full text not found
**ChiCTR2300074846**	Time-restricted eating	2023	Not yet recruiting	Full text not found
**CTRI/2022/02/040292**	Time-restricted eating	2022	Open to recruitment	Full text not found
**CTRI/2022/06/043312**	Time-restricted eating	2022	Open to recruitment	Wrong comparator
**CTRI/2022/11/047684**	Time-restricted eating	2022	Not yet recruiting	Full text not found
**CTRI/2022/07/044356**	Time-restricted eating	2022	Closed to recruitment	Wrong comparator

© 2024 Liu HY et al. *JAMA Network Open.*

**DRKS00031928**	Time-restricted eating	2023	Recruiting	Full text not found
**DRKS00028261**	Time-restricted eating	2022	Recruiting	Full text not found
**IRCT2012102911307N1**	Meal frequency	2012	Completed	Full text included (Zargaran 2014)
**KCT0005390**	Time-restricted eating	2020	Recruiting	Full text not found
**NCT02525419**	High-protein intermittent fasting	2015	Completed	Full text found<br>Wrong intervention
**NCT05742165**	Time-restricted eating	2023	Recruiting	Full text not found
**NCT02948517**	Time-restricted eating	2016	Completed	Full text found<br>Wrong study design
**NCT05581862**	Meal frequency	2022	Completed	Full text not found
**NCT03571048**	Time-restricted eating	2018	Completed	Full text included (Thomas 2022)
**NCT03745612**	Time-restricted eating	2018	Completed	Full text included (Liu 2022)
**NCT03792282**	Time-restricted eating	2019	Completed	Full text not found<br>Wrong comparator
**NCT04692532**	Time-restricted eating	2021	Enrolling by invitation	Full text not found
**NCT05548517**	Time-restricted eating	2022	Recruiting	Full text not found
**NCT04916730**	Time-restricted eating	2021	Recruiting	Full text not found
**NCT05870982**	Time-restricted eating	2023	Not yet recruiting	Full text not found
**NCT05880095**	Time-restricted eating	2023	Recruiting	Full text not found
**NCT05629858**	Time-restricted eating	2023	Enrolling by invitation	Full text not found
**NCT04127994**	Meal frequency	2019	Recruiting	Full text not found
**NCT01178723**	Meal distribution	2010	Unknown status	Full text not found

© 2024 Liu HY et al. *JAMA Network Open.*

**NCT03689608**	Intermittent fasting	2018	Completed	Full text found<br>Wrong intervention
**NCT04155619**	Time-restricted eating	2019	Recruiting	No anthropometric measurements
**NCT04997486**	Time-restricted eating	2021	Active, not recruiting	No anthropometric measurements
**NCT04243746**	Time-restricted eating	2020	Completed	Full text not found
**NCT04057339**	Time-restricted eating	2019	Active, not recruiting	Full text not found
**NCT05220956**	Time-restricted eating	2022	Recruiting	No anthropometric measurements
**NCT04502329**	Time-restricted eating	2020	Completed	Full text included (Kunduraci 2020)
**NCT01277471**	Meal frequency	2011	Completed	Full text included (Kahleova 2014)
**NCT01781780**	Breakfast skipping	2013	Completed	Full text included (Dhurandhar 2014)
**NCT03393195**	Time-restricted eating	2018	Completed	Full text included (Lowe 2020)
**NCT03459703**	Time-restricted eating	2018	Completed	Full text included (Jamshed 2022)
**NCT03802253**	Time-restricted eating	2019	Recruiting	Full text not found
**NCT04062773**	Time-restricted eating	2019	Unknown status	Full text not found
**NCT04131647**	Intermittent fasting	2019	Recruiting	Full text not found<br>Wrong intervention
**NCT04534985**	Time-restricted eating	2020	Active, not recruiting	Full text not found
**NCT04557540**	Intermittent fasting	2020	Recruiting	Wrong comparator
**NCT05126199**	Time-restricted eating	2021	Recruiting	Full text not found

© 2024 Liu HY et al. *JAMA Network Open.*

**NCT05225337**	Time-restricted eating	2022	Completed	Full text included (Pavlou 2023)
**NCT05229835**	Time-restricted eating	2022	Active, not recruiting	No anthropometric measurements
**NCT05230160**	Intermittent fasting	2022	Not yet recruiting	Wrong intervention
**NCT05290246**	Time-restricted eating	2022	Recruiting	Wrong comparator
**NCT05310721**	Time-restricted eating	2022	Completed	Full text included (Montero 2023)
**NCT05549362**	Time-restricted eating	2022	Recruiting	No anthropometric measurements
**NCT05730231**	Time-restricted eating	2022	Active, not recruiting	Full text not found
**NCT05860413**	Time-restricted eating	2022	Recruiting	Wrong comparator
**NCT06018415**	Time-restricted eating	2023	Not yet recruiting	Full text not found
**NL9756**	Time-restricted eating	2021	NA	Trial not found
**PACTR202301674821174**	Time-restricted eating	2023	Recruitment completed	Full text not found
**RBR-3p8346g**	Meal frequency	2021	Not yet recruiting	Full text not found
**RBR-45fpgqh**	Time-restricted eating	2023	Recruiting	Full text not found
**SLCTR/2018/001**	Time-restricted eating	2018	Pending	Full text not found
**TCTR20230131002**	Time-restricted eating	2023	Recruitment completed	Not yet published
**TCTR20210520002**	Time-restricted eating	2021	Completed	Full text included (Suthutvoravut 2023)
**UMIN000045481**	Breakfast skipping	2021	Recruitment suspended	Full text not found

Note. eTable 8 details the clinical trials excluded from the review at full text screening with current stage of completion and reason for exclusion.

© 2024 Liu HY et al. *JAMA Network Open.*

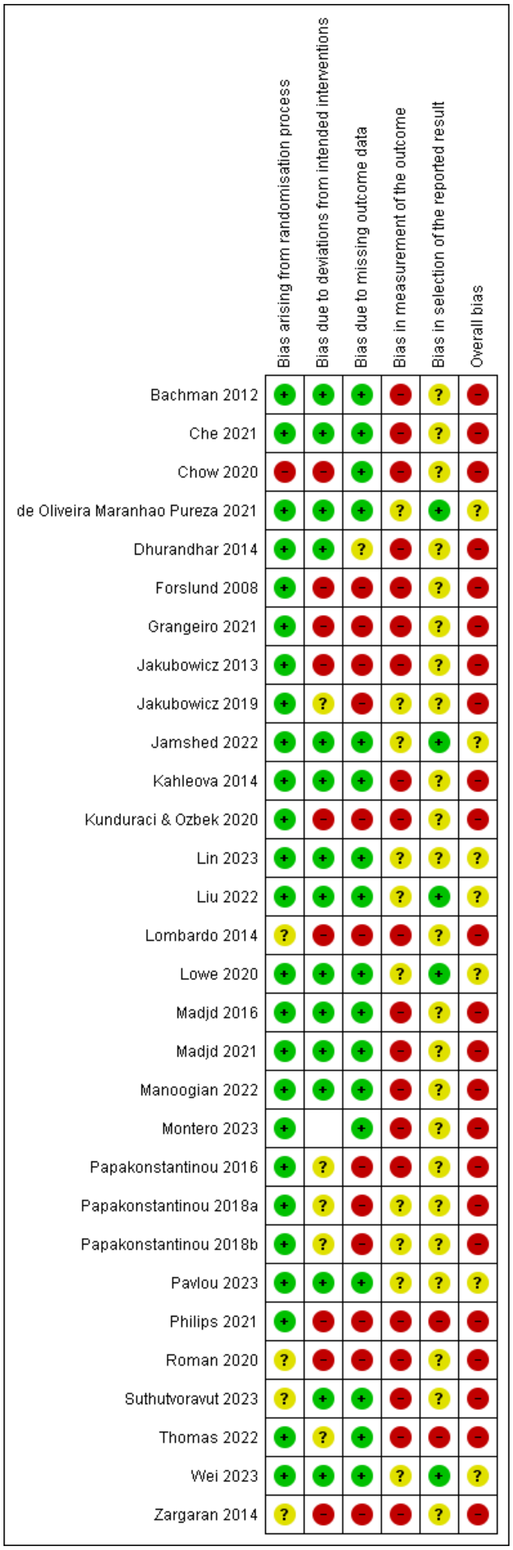

eFigure 1: Risk of summary for included studies.

Note. e Figure 1 details the individual risk of each study included in the review by domain. green = low risk, yellow = some caution, red = high risk

© 2024 Liu HY et al. *JAMA Network Open.*

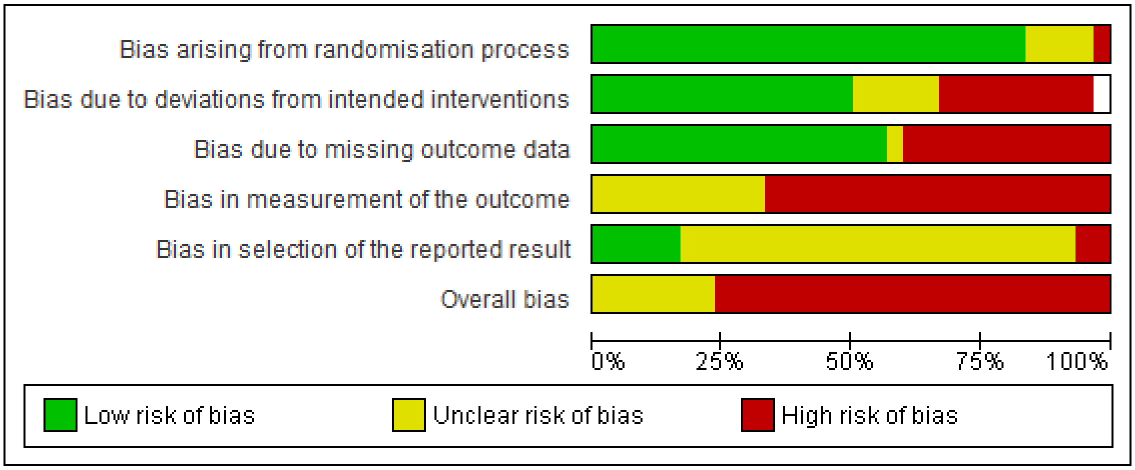

eFigure 2: Risk of bias graph for included studies.

Note. eFigure 2 details the overall risk of bias for all studies included in the review.

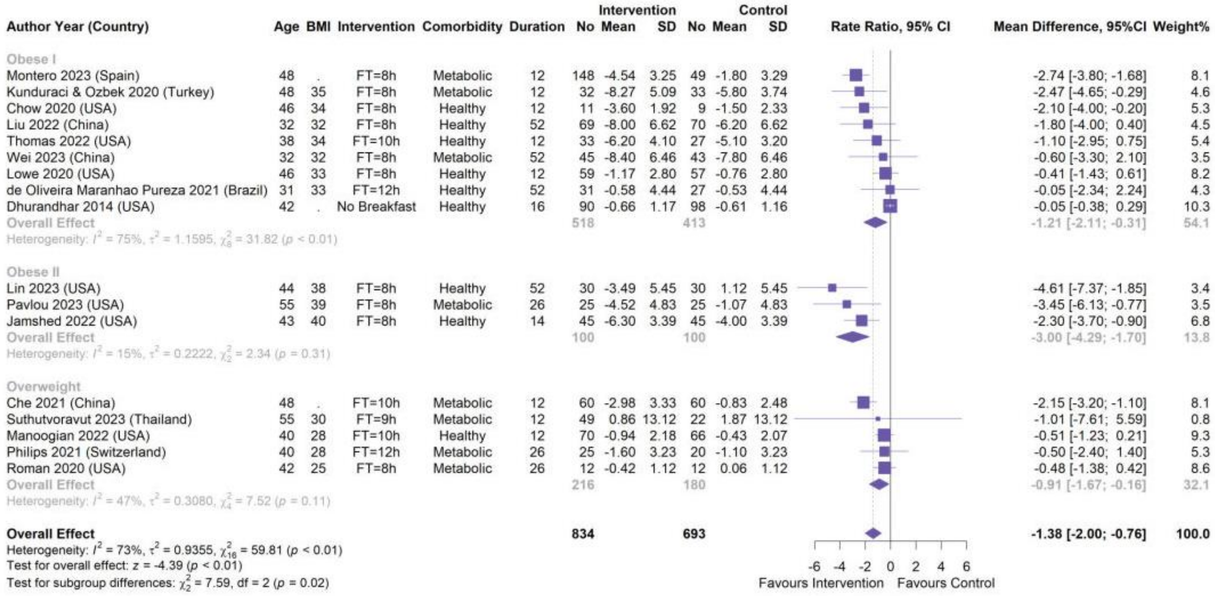

eFigure 3: Meta-analysis of difference in mean difference (95% CIs) for the effect of time-restricted eating on weight (kg), grouped by baseline BMI status.

BMI: Body Mass Index; SD: Standard Deviation; CI: Confidence Interval; USA: United States of America; FT: Feeding Time; Duration: follow-up duration in weeks; 2M/3M/6M: 2, 3 or 6 meals; 3M+2S: 3 meals and 2 snacks; 3M+3S: 3 meals and 3 snacks; 10S: Grazing diet – 100kcal every 2-3 hours; HCB: High Calorie Breakfast; HCD: High Calorie Dinner; ED: Early Dinner; LD: Late Dinner; FL: Front Loading: EED: Equal Energy Distribution; ML: Middle Loading; BL: Back Loading.

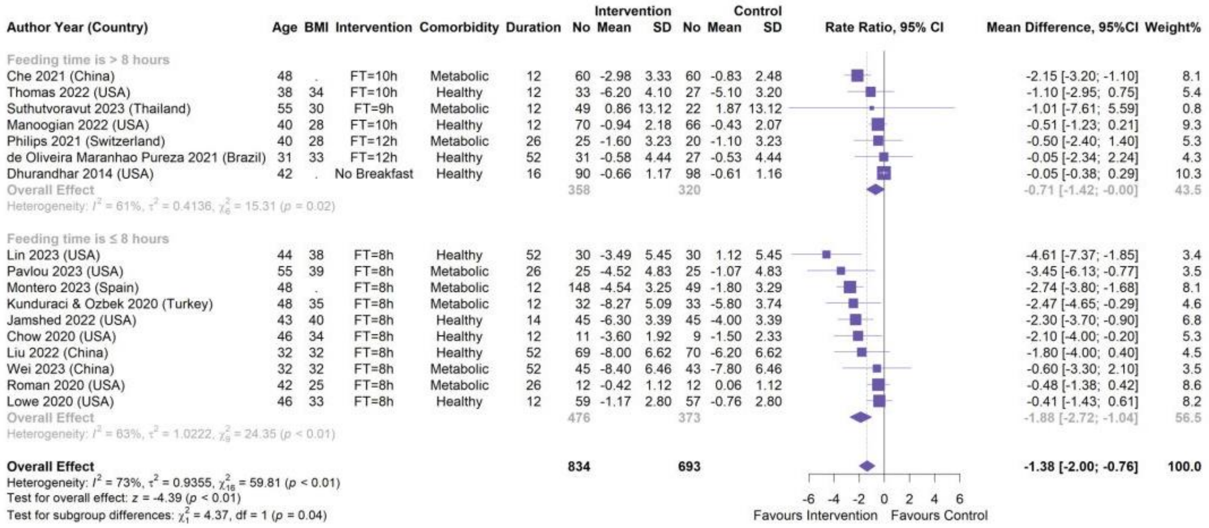

eFigure 4: Meta-analysis of difference in mean difference (95% CIs) for the effect of time-restricted eating interventions on weight (kg), grouped by eating window.

BMI: Body Mass Index; SD: Standard Deviation; CI: Confidence Interval; USA: United States of America; FT: Feeding Time; Duration: follow-up duration in weeks; 2M/3M/6M: 2, 3 or 6 meals; 3M+2S: 3 meals and 2 snacks; 3M+3S: 3 meals and 3 snacks; 10S: Grazing diet – 100kcal every 2-3 hours; HCB: High Calorie Breakfast; HCD: High Calorie Dinner; ED: Early Dinner; LD: Late Dinner; FL: Front Loading: EED: Equal Energy Distribution; ML: Middle Loading; BL: Back Loading.

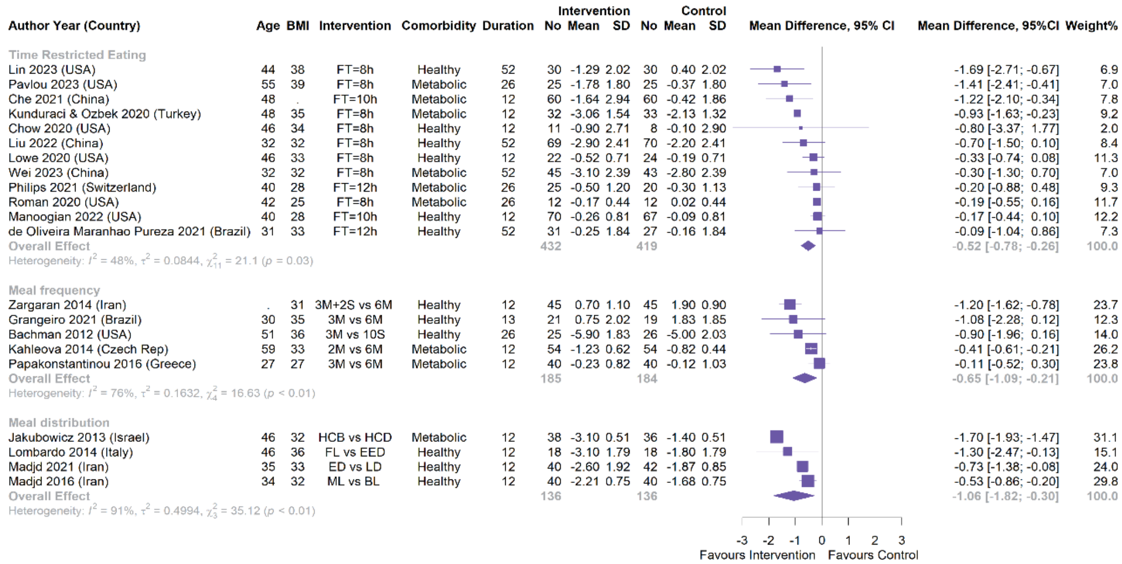

eFigure 5: Meta-analysis of difference in mean difference (95% CIs) for the effect of meal timing interventions on BMI (kg/m^2), grouped by intervention type.

BMI: Body Mass Index; SD: Standard Deviation; CI: Confidence Interval; USA: United States of America; FT: Feeding Time; Duration: follow-up duration in weeks; 2M/3M/6M: 2, 3 or 6 meals; 3M+2S: 3 meals and 2 snacks; 3M+3S: 3 meals and 3 snacks; 10S: Grazing diet – 100kcal every 2-3 hours; HCB: High Calorie Breakfast; HCD: High Calorie Dinner; ED: Early Dinner; LD: Late Dinner; FL: Front Loading: EED: Equal Energy Distribution; ML: Middle Loading; BL: Back Loading.

© 2024 Liu HY et al. *JAMA Network Open.*

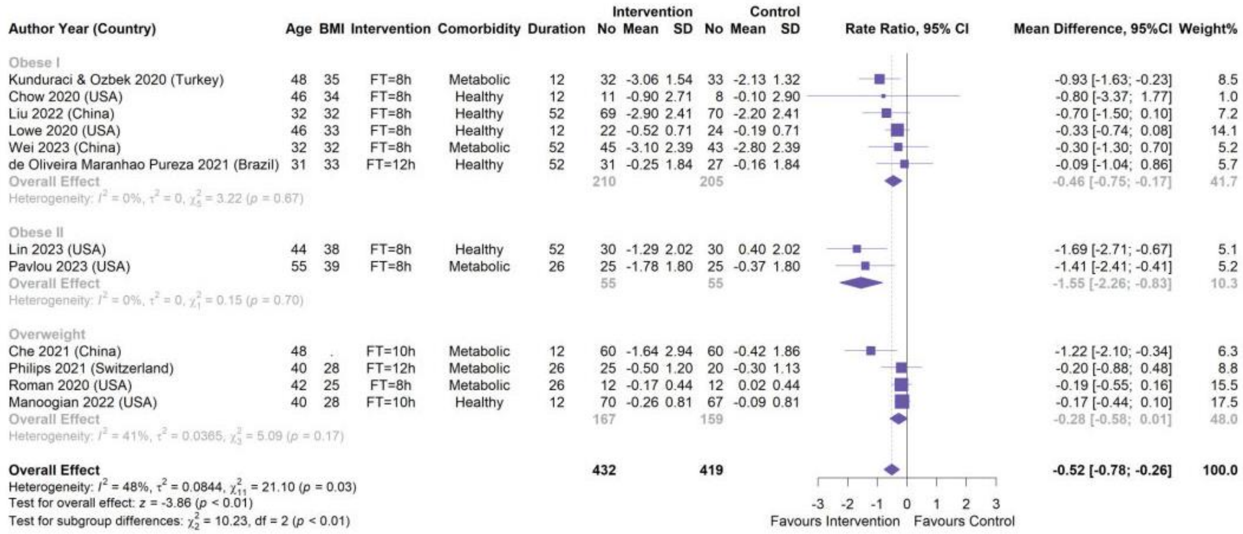

eFigure 6: Meta-analysis of difference in mean difference (95% CIs) for the effect of time-restricted eating on BMI (kg/m²), grouped by baseline BMI status.

BMI: Body Mass Index; SD: Standard Deviation; CI: Confidence Interval; USA: United States of America; FT: Feeding Time; Duration: follow-up duration in weeks; 2M/3M/6M: 2, 3 or 6 meals; 3M+2S: 3 meals and 2 snacks; 3M+3S: 3 meals and 3 snacks; 10S: Grazing diet – 100kcal every 2-3 hours; HCB: High Calorie Breakfast; HCD: High Calorie Dinner; ED: Early Dinner; LD: Late Dinner; FL: Front Loading: EED: Equal Energy Distribution; ML: Middle Loading; BL: Back Loading.

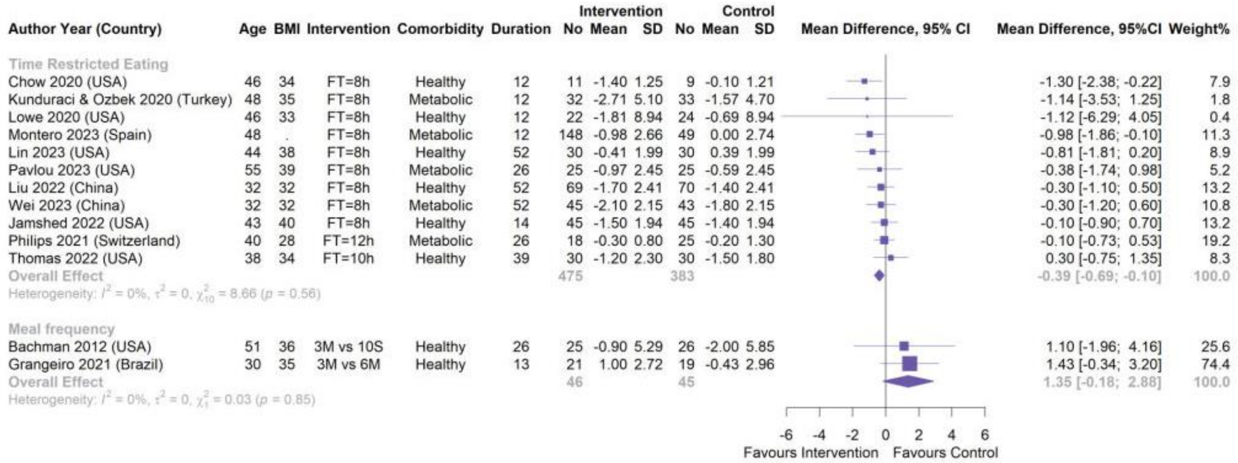

eFigure 7: Meta-analysis of difference in mean difference (95% CIs) for the effect of meal timing interventions on lean mass (kg), grouped by the nature of the meal timing intervention.

BMI: Body Mass Index; SD: Standard Deviation; CI: Confidence Interval; USA: United States of America; FT: Feeding Time; Duration: follow-up duration in weeks; 2M/3M/6M: 2, 3 or 6 meals; 3M+2S: 3 meals and 2 snacks; 3M+3S: 3 meals and 3 snacks; 10S: Grazing diet – 100kcal every 2-3 hours; HCB: High Calorie Breakfast; HCD: High Calorie Dinner; ED: Early Dinner; LD: Late Dinner; FL: Front Loading: EED: Equal Energy Distribution; ML: Middle Loading; BL: Back Loading.

© 2024 Liu HY et al. *JAMA Network Open.*

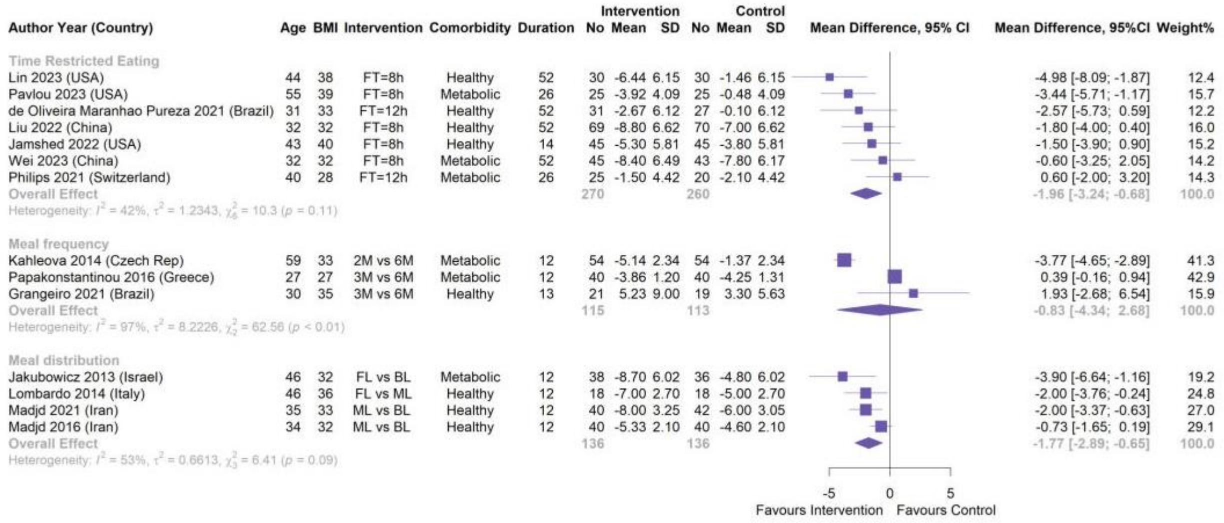

eFigure 8: Meta-analysis of difference in mean difference (95% CIs) for the effect of meal timing interventions on waist circumference (cm), grouped by meal timing intervention.

BMI: Body Mass Index; SD: Standard Deviation; CI: Confidence Interval; USA: United States of America; FT: Feeding Time; Duration: follow-up duration in weeks; 2M/3M/6M: 2, 3 or 6 meals; 3M+2S: 3 meals and 2 snacks; 3M+3S: 3 meals and 3 snacks; 10S: Grazing diet – 100kcal every 2-3 hours; HCB: High Calorie Breakfast; HCD: High Calorie Dinner; ED: Early Dinner; LD: Late Dinner; FL: Front Loading: EED: Equal Energy Distribution; ML: Middle Loading; BL: Back Loading.

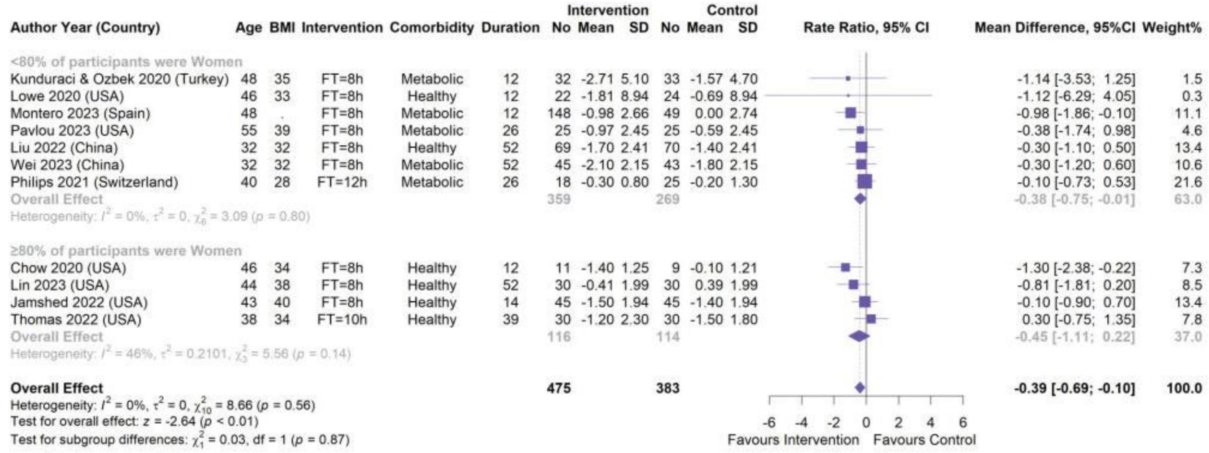

eFigure 9: Meta-analysis of difference in mean difference (95% CIs) for the effect of time-restricted eating on lean mass (kg), grouped by gender proportion.

BMI: Body Mass Index; SD: Standard Deviation; CI: Confidence Interval; USA: United States of America; FT: Feeding Time; Duration: follow-up duration in weeks; 2M/3M/6M: 2, 3 or 6 meals; 3M+2S: 3 meals and 2 snacks; 3M+3S: 3 meals and 3 snacks; 10S: Grazing diet – 100kcal every 2-3 hours; HCB: High Calorie Breakfast; HCD: High Calorie Dinner; ED: Early Dinner; LD: Late Dinner; FL: Front Loading: EED: Equal Energy Distribution; ML: Middle Loading; BL: Back Loading.

© 2024 Liu HY et al. *JAMA Network Open.*

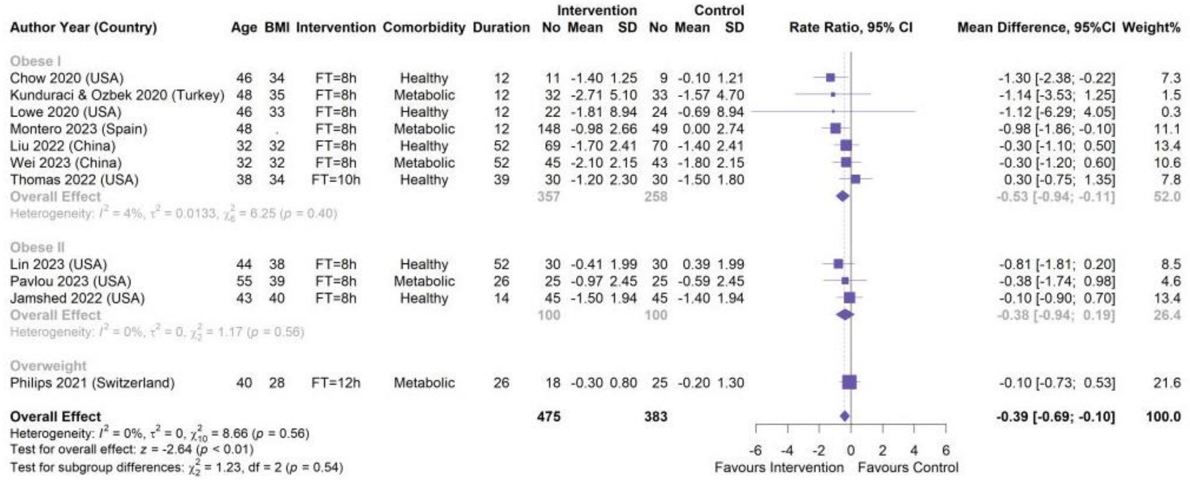

eFigure 10: Meta-analysis of difference in mean difference (95% CIs) for the effect of time-restricted eating on lean mass (kg), grouped by baseline BMI status.

BMI: Body Mass Index; SD: Standard Deviation; CI: Confidence Interval; USA: United States of America; FT: Feeding Time; Duration: follow-up duration in weeks; 2M/3M/6M: 2, 3 or 6 meals; 3M+2S: 3 meals and 2 snacks; 3M+3S: 3 meals and 3 snacks; 10S: Grazing diet – 100kcal every 2-3 hours; HCB: High Calorie Breakfast; HCD: High Calorie Dinner; ED: Early Dinner; LD: Late Dinner; FL: Front Loading: EED: Equal Energy Distribution; ML: Middle Loading; BL: Back Loading.

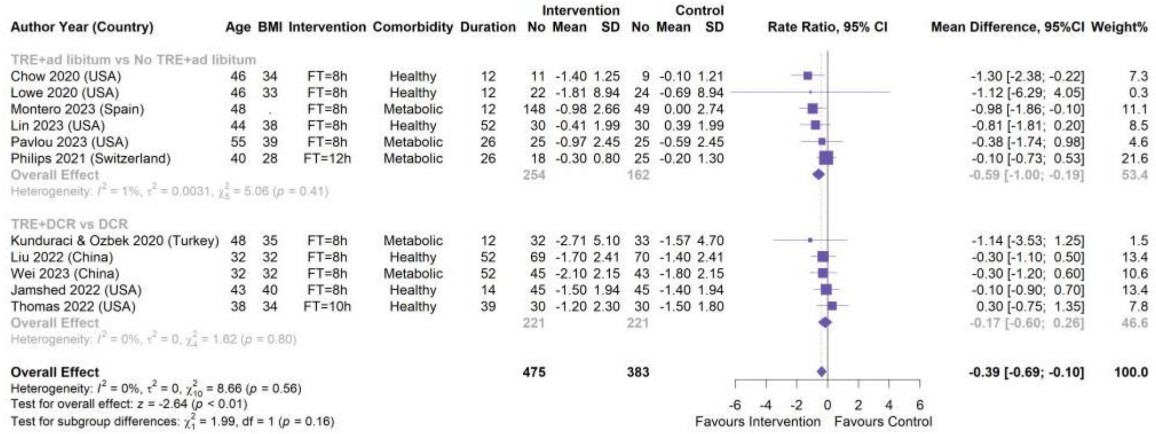

eFigure 11: Meta-analysis of difference in mean difference (95% CIs) for the effect of time-restricted eating on lean mass (kg), grouped by energy prescription.

BMI: Body Mass Index; SD: Standard Deviation; CI: Confidence Interval; USA: United States of America; FT: Feeding Time; Duration: follow-up duration in weeks; 2M/3M/6M: 2, 3 or 6 meals; 3M+2S: 3 meals and 2 snacks; 3M+3S: 3 meals and 3 snacks; 10S: Grazing diet – 100kcal every 2-3 hours; HCB: High Calorie Breakfast; HCD: High Calorie Dinner; ED: Early Dinner; LD: Late Dinner; FL: Front Loading: EED: Equal Energy Distribution; ML: Middle Loading; BL: Back Loading.

© 2024 Liu HY et al. *JAMA Network Open.*

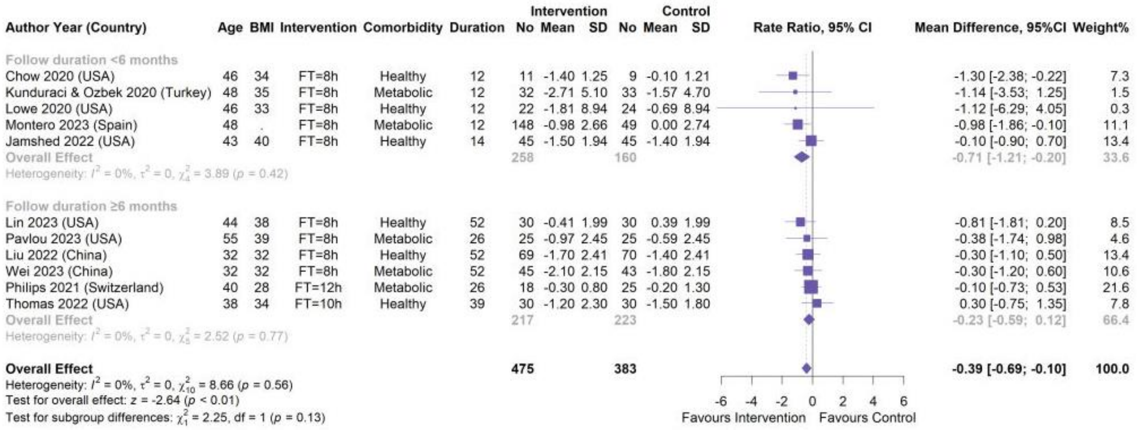

eFigure 12: Meta-analysis of difference in mean difference (95% CIs) for the effect of time-restricted eating on lean mass (kg), grouped by follow duration.

BMI: Body Mass Index; SD: Standard Deviation; CI: Confidence Interval; USA: United States of America; FT: Feeding Time; Duration: follow-up duration in weeks; 2M/3M/6M: 2, 3 or 6 meals; 3M+2S: 3 meals and 2 snacks; 3M+3S: 3 meals and 3 snacks; 10S: Grazing diet – 100kcal every 2-3 hours; HCB: High Calorie Breakfast; HCD: High Calorie Dinner; ED: Early Dinner; LD: Late Dinner; FL: Front Loading: EED: Equal Energy Distribution; ML: Middle Loading; BL: Back Loading.

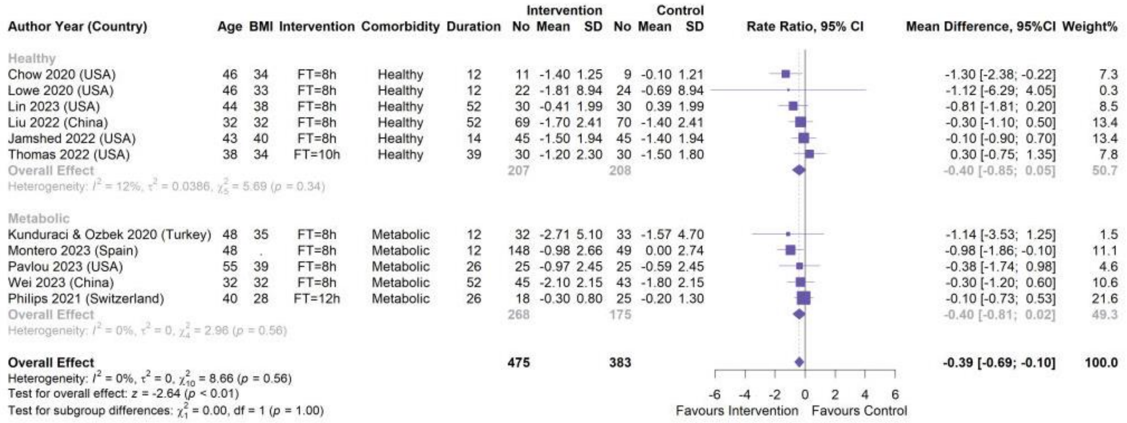

eFigure 13: Meta-analysis of difference in mean difference (95% CIs) for the effect of time-restricted eating on lean mass (kg), grouped by health status.

BMI: Body Mass Index; SD: Standard Deviation; CI: Confidence Interval; USA: United States of America; FT: Feeding Time; Duration: follow-up duration in weeks; 2M/3M/6M: 2, 3 or 6 meals; 3M+2S: 3 meals and 2 snacks; 3M+3S: 3 meals and 3 snacks; 10S: Grazing diet – 100kcal every 2-3 hours; HCB: High Calorie Breakfast; HCD: High Calorie Dinner; ED: Early Dinner; LD: Late Dinner; FL: Front Loading: EED: Equal Energy Distribution; ML: Middle Loading; BL: Back Loading.

© 2024 Liu HY et al. *JAMA Network Open.*

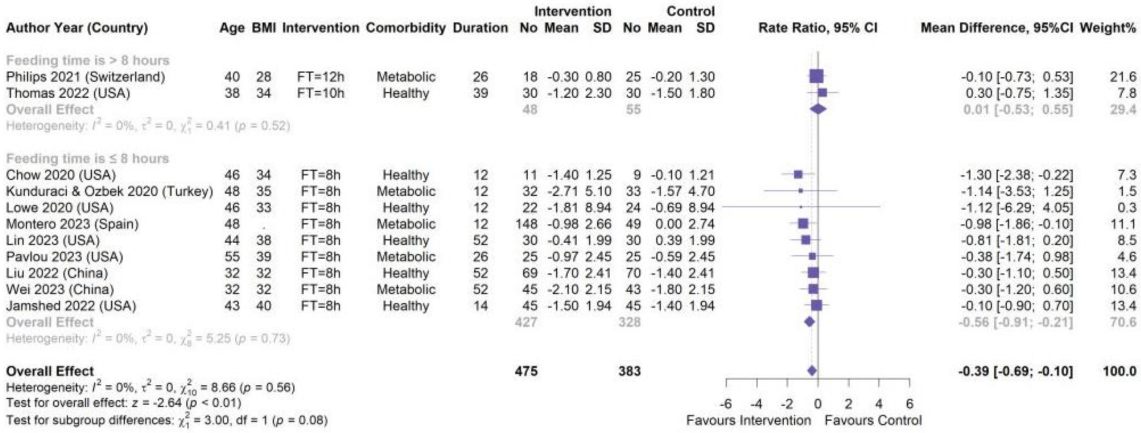

eFigure 14: Meta-analysis of difference in mean difference (95% CIs) for the effect of time-restricted eating on lean mass (kg), grouped by eating window.

BMI: Body Mass Index; SD: Standard Deviation; CI: Confidence Interval; USA: United States of America; FT: Feeding Time; Duration: follow-up duration in weeks; 2M/3M/6M: 2, 3 or 6 meals; 3M+2S: 3 meals and 2 snacks; 3M+3S: 3 meals and 3 snacks; 10S: Grazing diet – 100kcal every 2-3 hours; HCB: High Calorie Breakfast; HCD: High Calorie Dinner; ED: Early Dinner; LD: Late Dinner; FL: Front Loading: EED: Equal Energy Distribution; ML: Middle Loading; BL: Back Loading.

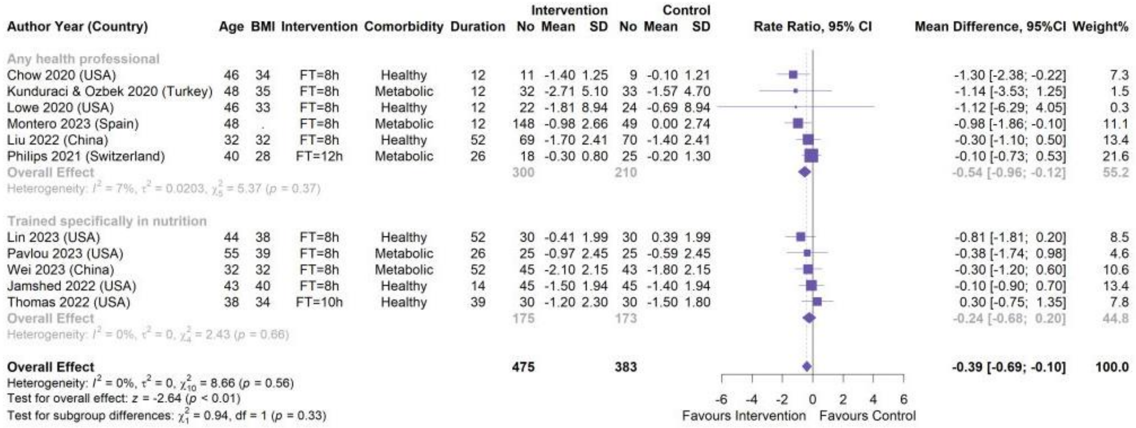

eFigure 15: Meta-analysis of difference in mean difference (95% CIs) for the effect of time-restricted eating on lean mass (kg), grouped by delivery personnel.

BMI: Body Mass Index; SD: Standard Deviation; CI: Confidence Interval; USA: United States of America; FT: Feeding Time; Duration: follow-up duration in weeks; 2M/3M/6M: 2, 3 or 6 meals; 3M+2S: 3 meals and 2 snacks; 3M+3S: 3 meals and 3 snacks; 10S: Grazing diet – 100kcal every 2-3 hours; HCB: High Calorie Breakfast; HCD: High Calorie Dinner; ED: Early Dinner; LD: Late Dinner; FL: Front Loading: EED: Equal Energy Distribution; ML: Middle Loading; BL: Back Loading.

© 2024 Liu HY et al. *JAMA Network Open.*

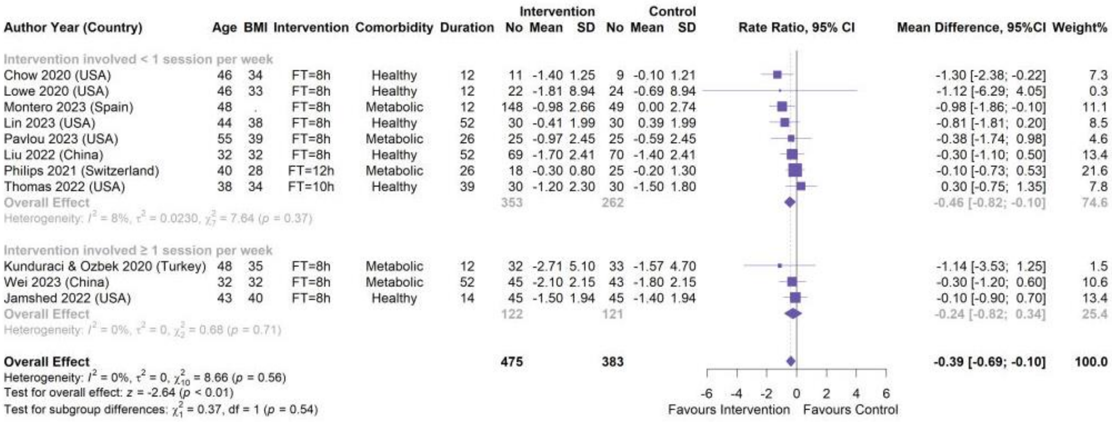

eFigure 16: Meta-analysis of difference in mean difference (95% CIs) for the effect of time-restricted eating on lean mass (kg), grouped by frequency of contact.

BMI: Body Mass Index; SD: Standard Deviation; CI: Confidence Interval; USA: United States of America; FT: Feeding Time; Duration: follow-up duration in weeks; 2M/3M/6M: 2, 3 or 6 meals; 3M+2S: 3 meals and 2 snacks; 3M+3S: 3 meals and 3 snacks; 10S: Grazing diet – 100kcal every 2-3 hours; HCB: High Calorie Breakfast; HCD: High Calorie Dinner; ED: Early Dinner; LD: Late Dinner; FL: Front Loading: EED: Equal Energy Distribution; ML: Middle Loading; BL: Back Loading.

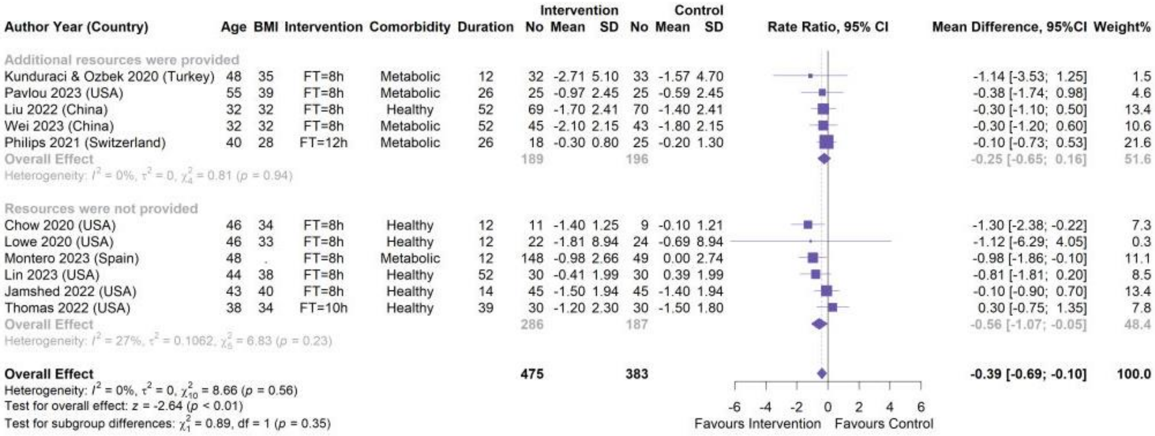

eFigure 17: Meta-analysis of difference in mean difference (95% CIs) for the effect of time-restricted eating on lean mass (kg), grouped by resource provision.

BMI: Body Mass Index; SD: Standard Deviation; CI: Confidence Interval; USA: United States of America; FT: Feeding Time; Duration: follow-up duration in weeks; 2M/3M/6M: 2, 3 or 6 meals; 3M+2S: 3 meals and 2 snacks; 3M+3S: 3 meals and 3 snacks; 10S: Grazing diet – 100kcal every 2-3 hours; HCB: High Calorie Breakfast; HCD: High Calorie Dinner; ED: Early Dinner; LD: Late Dinner; FL: Front Loading: EED: Equal Energy Distribution; ML: Middle Loading; BL: Back Loading.

© 2024 Liu HY et al. *JAMA Network Open.*

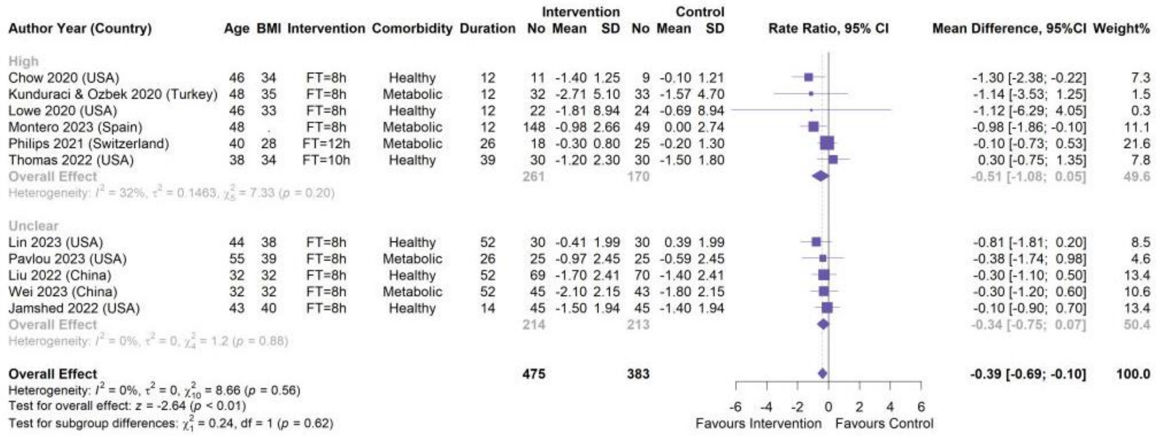

eFigure 18: Meta-analysis of difference in mean difference (95% CIs) for the effect of time-restricted eating on lean mass (kg), grouped by risk of bias.

BMI: Body Mass Index; SD: Standard Deviation; CI: Confidence Interval; USA: United States of America; FT: Feeding Time; Duration: follow-up duration in weeks; 2M/3M/6M: 2, 3 or 6 meals; 3M+2S: 3 meals and 2 snacks; 3M+3S: 3 meals and 3 snacks; 10S: Grazing diet – 100kcal every 2-3 hours; HCB: High Calorie Breakfast; HCD: High Calorie Dinner; ED: Early Dinner; LD: Late Dinner; FL: Front Loading: EED: Equal Energy Distribution; ML: Middle Loading; BL: Back Loading.

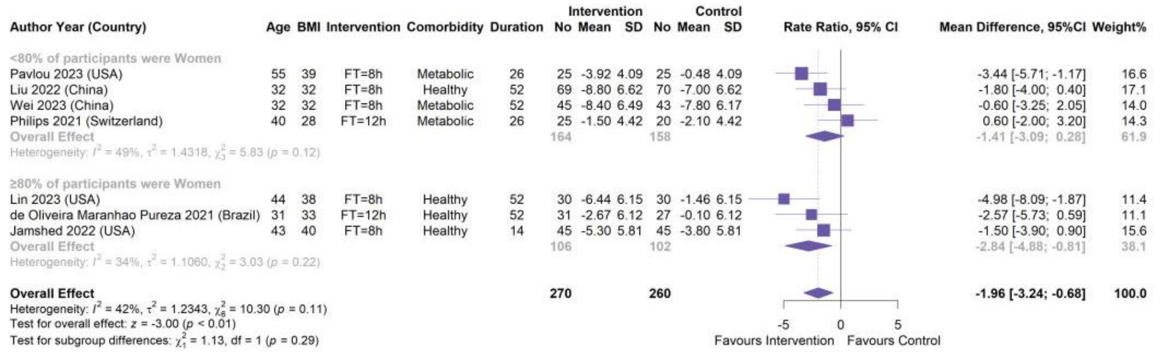

eFigure 19: Meta-analysis of difference in mean difference (95% CIs) for the effect of time-restricted eating on waist circumference (cm), grouped by gender proportion.

BMI: Body Mass Index; SD: Standard Deviation; CI: Confidence Interval; USA: United States of America; FT: Feeding Time; Duration: follow-up duration in weeks; 2M/3M/6M: 2, 3 or 6 meals; 3M+2S: 3 meals and 2 snacks; 3M+3S: 3 meals and 3 snacks; 10S: Grazing diet – 100kcal every 2-3 hours; HCB: High Calorie Breakfast; HCD: High Calorie Dinner; ED: Early Dinner; LD: Late Dinner; FL: Front Loading: EED: Equal Energy Distribution; ML: Middle Loading; BL: Back Loading.

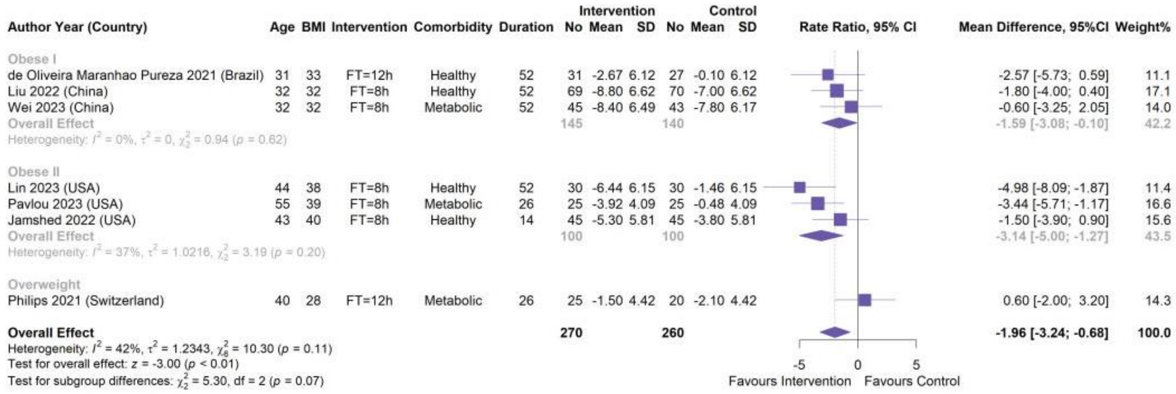

© 2024 Liu HY et al. *JAMA Network Open.*

eFigure 20: Meta-analysis of difference in mean difference (95% CIs) for the effect of time-restricted eating on waist circumference (cm), grouped by baseline BMI status.

BMI: Body Mass Index; SD: Standard Deviation; CI: Confidence Interval; USA: United States of America; FT: Feeding Time; Duration: follow-up duration in weeks; 2M/3M/6M: 2, 3 or 6 meals; 3M+2S: 3 meals and 2 snacks; 3M+3S: 3 meals and 3 snacks; 10S: Grazing diet – 100kcal every 2-3 hours; HCB: High Calorie Breakfast; HCD: High Calorie Dinner; ED: Early Dinner; LD: Late Dinner; FL: Front Loading: EED: Equal Energy Distribution; ML: Middle Loading; BL: Back Loading.

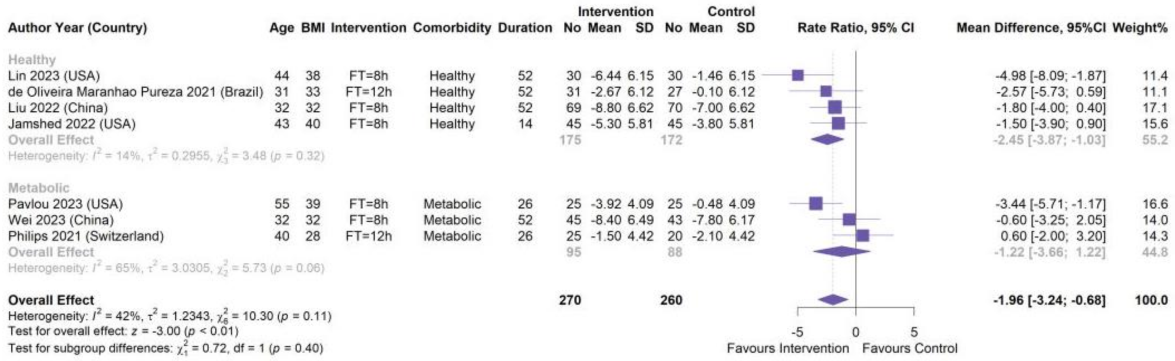

eFigure 21: Meta-analysis of difference in mean difference (95% CIs) for the effect of time-restricted eating on waist circumference (cm), grouped by health status.

BMI: Body Mass Index; SD: Standard Deviation; CI: Confidence Interval; USA: United States of America; FT: Feeding Time; Duration: follow-up duration in weeks; 2M/3M/6M: 2, 3 or 6 meals; 3M+2S: 3 meals and 2 snacks; 3M+3S: 3 meals and 3 snacks; 10S: Grazing diet – 100kcal every 2-3 hours; HCB: High Calorie Breakfast; HCD: High Calorie Dinner; ED: Early Dinner; LD: Late Dinner; FL: Front Loading: EED: Equal Energy Distribution; ML: Middle Loading; BL: Back Loading.

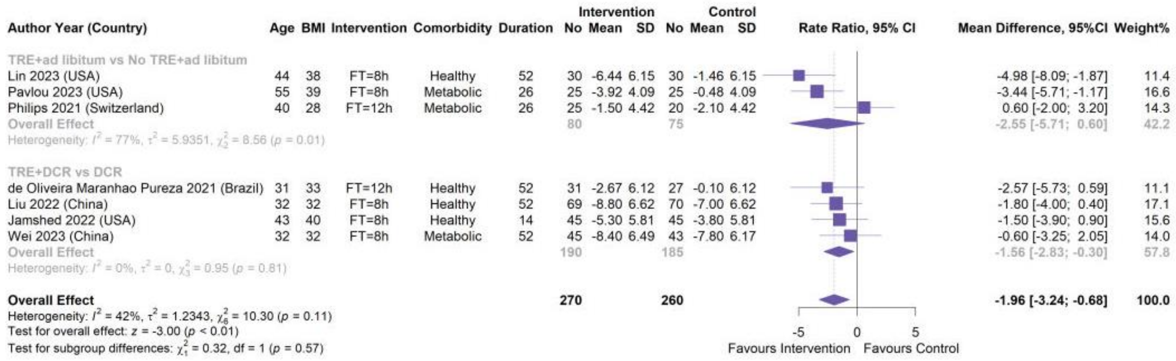

eFigure 22: Meta-analysis of difference in mean difference (95% CIs) for the effect of time-restricted eating on waist circumference (cm), grouped by energy prescription.

BMI: Body Mass Index; SD: Standard Deviation; CI: Confidence Interval; USA: United States of America; FT: Feeding Time; Duration: follow-up duration in weeks; 2M/3M/6M: 2, 3 or 6 meals; 3M+2S: 3 meals and 2 snacks; 3M+3S: 3 meals and 3 snacks; 10S: Grazing diet – 100kcal every 2-3 hours; HCB: High Calorie Breakfast; HCD: High Calorie Dinner; ED: Early Dinner; LD: Late Dinner; FL: Front Loading: EED: Equal Energy Distribution; ML: Middle Loading; BL: Back Loading.

© 2024 Liu HY et al. *JAMA Network Open.*

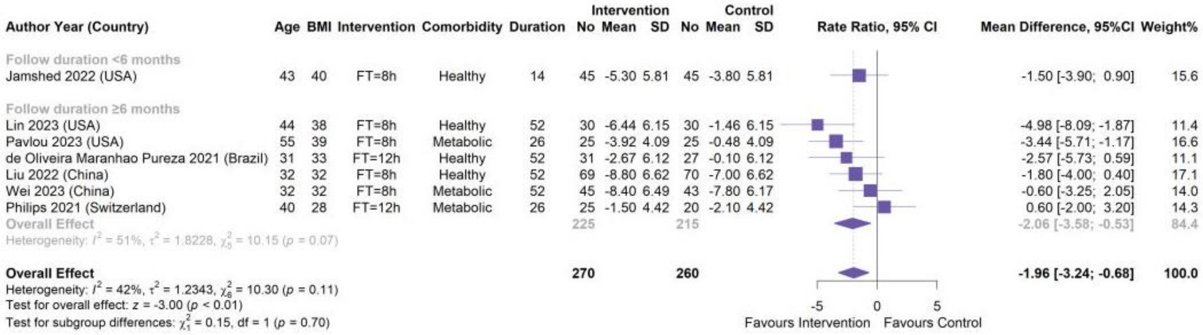

eFigure 23: Meta-analysis of difference in mean difference (95% CIs) for the effect of time-restricted eating on waist circumference (cm), grouped by follow duration.

BMI: Body Mass Index; SD: Standard Deviation; CI: Confidence Interval; USA: United States of America; FT: Feeding Time; Duration: follow-up duration in weeks; 2M/3M/6M: 2, 3 or 6 meals; 3M+2S: 3 meals and 2 snacks; 3M+3S: 3 meals and 3 snacks; 10S: Grazing diet – 100kcal every 2-3 hours; HCB: High Calorie Breakfast; HCD: High Calorie Dinner; ED: Early Dinner; LD: Late Dinner; FL: Front Loading: EED: Equal Energy Distribution; ML: Middle Loading; BL: Back Loading.

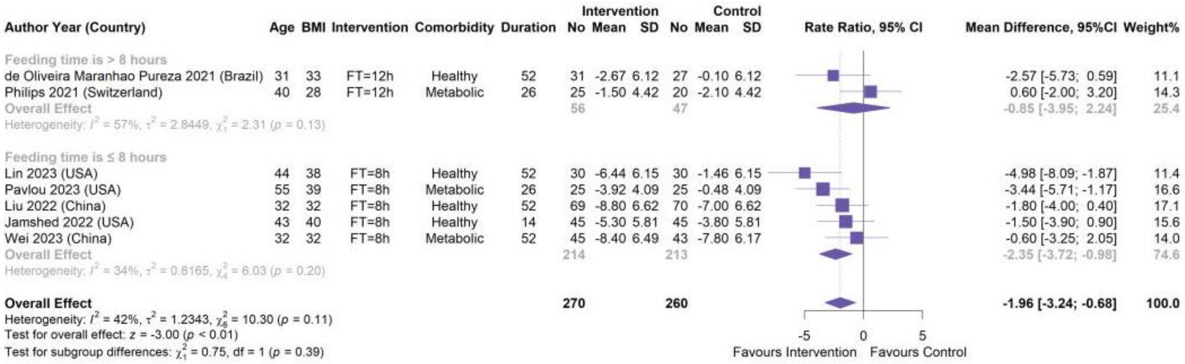

eFigure 24: Meta-analysis of difference in mean difference (95% CIs) for the effect of time-restricted eating on waist circumference (cm), grouped by eating window.

BMI: Body Mass Index; SD: Standard Deviation; CI: Confidence Interval; USA: United States of America; FT: Feeding Time; Duration: follow-up duration in weeks; 2M/3M/6M: 2, 3 or 6 meals; 3M+2S: 3 meals and 2 snacks; 3M+3S: 3 meals and 3 snacks; 10S: Grazing diet – 100kcal every 2-3 hours; HCB: High Calorie Breakfast; HCD: High Calorie Dinner; ED: Early Dinner; LD: Late Dinner; FL: Front Loading: EED: Equal Energy Distribution; ML: Middle Loading; BL: Back Loading.

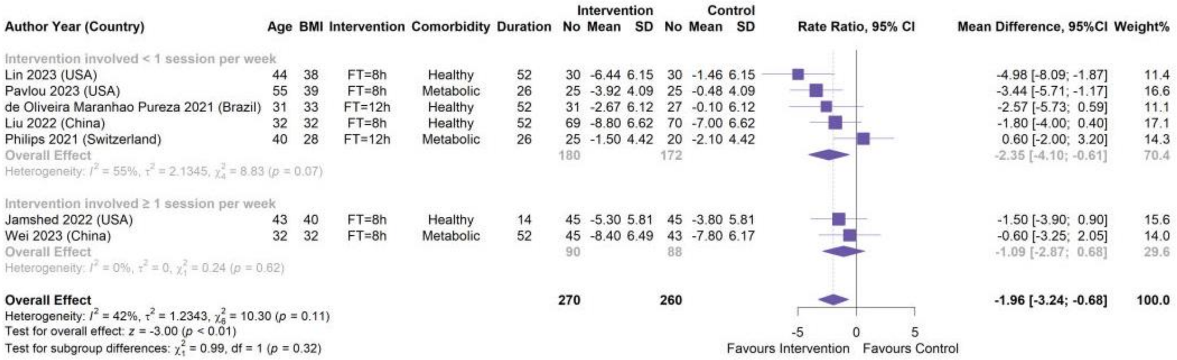

© 2024 Liu HY et al. *JAMA Network Open.*

eFigure 25: Meta-analysis of difference in mean difference (95% CIs) for the effect of time-restricted eating on waist circumference (cm), grouped by frequency of contact.

BMI: Body Mass Index; SD: Standard Deviation; CI: Confidence Interval; USA: United States of America; FT: Feeding Time; Duration: follow-up duration in weeks; 2M/3M/6M: 2, 3 or 6 meals; 3M+2S: 3 meals and 2 snacks; 3M+3S: 3 meals and 3 snacks; 10S: Grazing diet – 100kcal every 2-3 hours; HCB: High Calorie Breakfast; HCD: High Calorie Dinner; ED: Early Dinner; LD: Late Dinner; FL: Front Loading: EED: Equal Energy Distribution; ML: Middle Loading; BL: Back Loading.

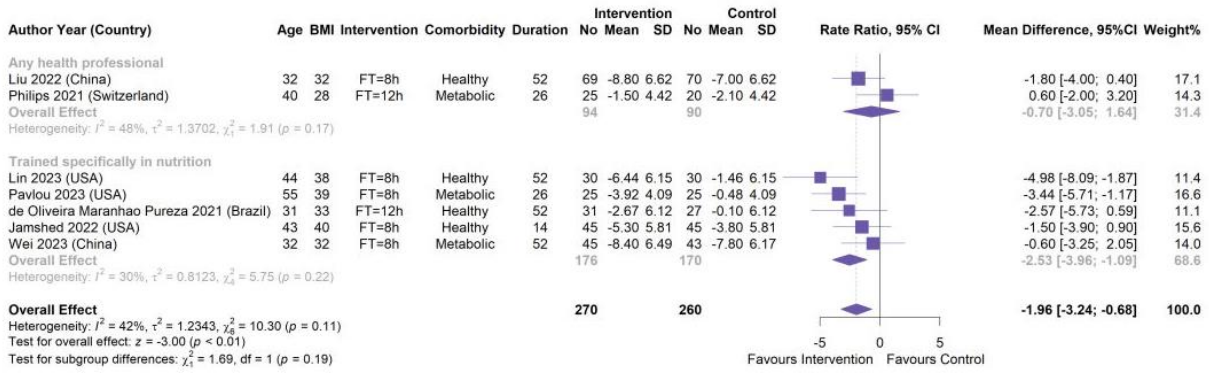

eFigure 26: Meta-analysis of difference in mean difference (95% CIs) for the effect of time-restricted eating on waist circumference (cm), grouped by delivery personnel.

BMI: Body Mass Index; SD: Standard Deviation; CI: Confidence Interval; USA: United States of America; FT: Feeding Time; Duration: follow-up duration in weeks; 2M/3M/6M: 2, 3 or 6 meals; 3M+2S: 3 meals and 2 snacks; 3M+3S: 3 meals and 3 snacks; 10S: Grazing diet – 100kcal every 2-3 hours; HCB: High Calorie Breakfast; HCD: High Calorie Dinner; ED: Early Dinner; LD: Late Dinner; FL: Front Loading: EED: Equal Energy Distribution; ML: Middle Loading; BL: Back Loading.

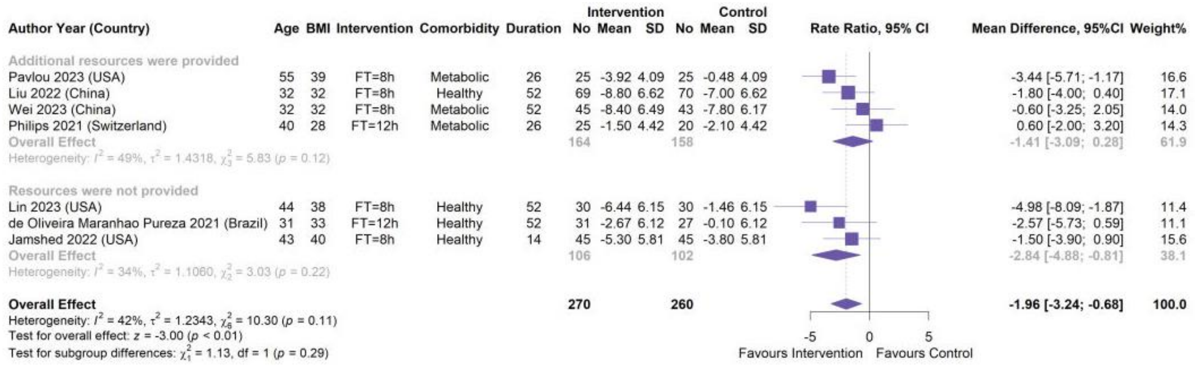

eFigure 27: Meta-analysis of difference in mean difference (95% CIs) for the effect of time-restricted eating on waist circumference (cm), grouped by resource provision.

BMI: Body Mass Index; SD: Standard Deviation; CI: Confidence Interval; USA: United States of America; FT: Feeding Time; Duration: follow-up duration in weeks; 2M/3M/6M: 2, 3 or 6 meals; 3M+2S: 3 meals and 2 snacks; 3M+3S: 3 meals and 3 snacks; 10S: Grazing diet – 100kcal every 2-3 hours; HCB: High Calorie Breakfast; HCD: High Calorie Dinner; ED: Early Dinner; LD: Late Dinner; FL: Front Loading: EED: Equal Energy Distribution; ML: Middle Loading; BL: Back Loading.

© 2024 Liu HY et al. *JAMA Network Open.*

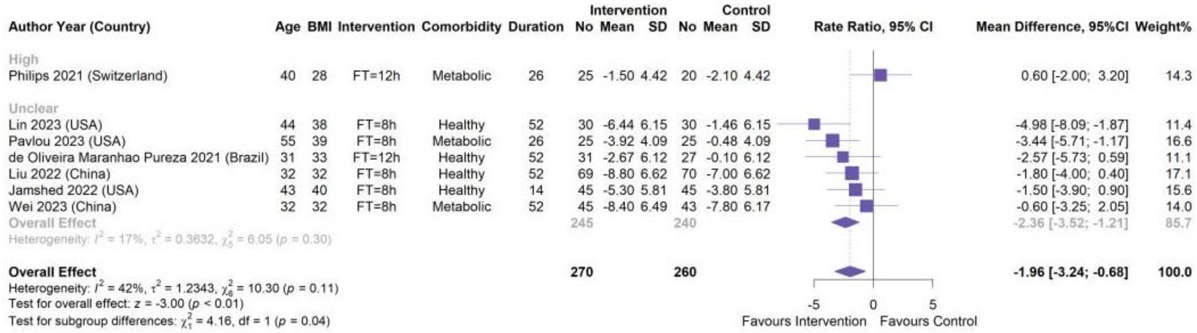

eFigure 28: Meta-analysis of difference in mean difference (95% CIs) for the effect of time-restricted eating on waist circumference (cm), grouped by risk of bias.

BMI: Body Mass Index; SD: Standard Deviation; CI: Confidence Interval; USA: United States of America; FT: Feeding Time; Duration: follow-up duration in weeks; 2M/3M/6M: 2, 3 or 6 meals; 3M+2S: 3 meals and 2 snacks; 3M+3S: 3 meals and 3 snacks; 10S: Grazing diet – 100kcal every 2-3 hours; HCB: High Calorie Breakfast; HCD: High Calorie Dinner; ED: Early Dinner; LD: Late Dinner; FL: Front Loading: EED: Equal Energy Distribution; ML: Middle Loading; BL: Back Loading.

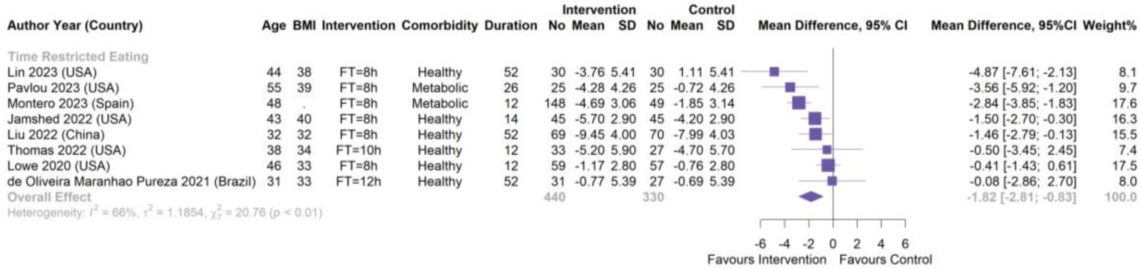

eFigure 29: Meta-analysis of difference in mean difference (95% CIs) for the effect of meal timing interventions on percentage of weight loss (%), grouped by the meal timing intervention.

BMI: Body Mass Index; SD: Standard Deviation; CI: Confidence Interval; USA: United States of America; FT: Feeding Time; Duration: follow-up duration in weeks; 2M/3M/6M: 2, 3 or 6 meals; 3M+2S: 3 meals and 2 snacks; 3M+3S: 3 meals and 3 snacks; 10S: Grazing diet – 100kcal every 2-3 hours; HCB: High Calorie Breakfast; HCD: High Calorie Dinner; ED: Early Dinner; LD: Late Dinner; FL: Front Loading: EED: Equal Energy Distribution; ML: Middle Loading; BL: Back Loading.

© 2024 Liu HY et al. *JAMA Network Open.*

Author Year (Country)	Age	BMI	Intervention	Comorbidity	Duration	Intervention No	Intervention Mean	Intervention SD	Control No	Control Mean	Control SD	Mean Difference, 95%CI	Weight%
**Time Restricted Eating**													
Che 2021 (China)	48	.	FT=10h	Metabolic	12	60	-26.49	34.86	60	-14.05	29.28	-12.44 [-23.96; -0.92]	5.4
Philips 2021 (Switzerland)	40	28	FT=12h	Metabolic	26	23	-3.06	10.22	18	2.16	10.22	-5.23 [-11.53; 1.08]	7.4
Wei 2023 (China)	32	32	FT=8h	Metabolic	52	45	-5.90	17.47	43	-0.80	17.47	-5.10 [-12.40; 2.20]	7.0
Suthutvoravut 2023 (Thailand)	55	30	FT=9h	Metabolic	12	49	-4.54	7.63	22	0.20	7.63	-4.74 [-8.58; -0.90]	8.2
Lin 2023 (USA)	44	38	FT=8h	Healthy	52	30	2.82	11.76	30	6.26	11.76	-3.44 [-9.39; 2.51]	7.5
Kunduraci & Ozbek 2020 (Turkey)	48	35	FT=8h	Metabolic	12	32	-15.47	37.70	33	-13.12	28.40	-2.35 [-18.61; 13.91]	3.9
Montero 2023 (Spain)	48	.	FT=8h	Metabolic	12	148	-1.92	9.40	49	0.34	9.57	-2.26 [-5.34; 0.82]	8.4
Jamshed 2022 (USA)	43	40	FT=8h	Healthy	14	45	-8.00	16.94	45	-6.00	16.94	-2.00 [-9.00; 5.00]	7.1
Lowe 2020 (USA)	46	33	FT=8h	Healthy	12	22	-1.06	6.74	24	0.29	6.74	-1.35 [-5.25; 2.55]	8.2
Chow 2020 (USA)	46	34	FT=8h	Healthy	12	11	-8.00	8.21	9	-7.00	13.93	-1.00 [-11.31; 9.31]	5.8
Thomas 2022 (USA)	38	34	FT=10h	Healthy	39	18	2.20	4.30	20	3.20	19.30	-1.00 [-9.69; 7.69]	6.4
Pavlou 2023 (USA)	55	39	FT=8h	Metabolic	26	25	-0.72	1.26	25	0.19	1.26	-0.91 [-1.61; -0.21]	8.7
Liu 2022 (China)	32	32	FT=8h	Healthy	52	69	-3.50	16.54	70	-2.90	16.54	-0.60 [-6.10; 4.90]	7.6
Manoogian 2022 (USA)	40	28	FT=10h	Healthy	12	70	0.19	6.99	66	0.14	6.99	0.05 [-2.30; 2.40]	8.5
Overall Effect						647			514			-1.15 [-1.77; -0.53]	100.0
Heterogeneity: $I^2 = 0\%$, $\tau^2 = 0$, $\chi^2_{13} = 12.43$ ($p = 0.49$)													
**Meal frequency**													
Jakubowicz 2019 (Israel)	69	32	3M vs 6M	Metabolic	12	14	-55.00	2.09	14	-23.00	2.26	-32.00 [-33.61; -30.39]	15.2
Kahleova 2014 (Czech Rep)	59	33	2M vs 6M	Metabolic	12	54	-14.18	7.07	54	-8.47	7.25	-5.71 [-8.41; -3.01]	14.9
Papakonstantinou 2018a (Greece)	48	32	3M vs 6M	Metabolic	12	35	-3.06	7.21	35	-0.54	3.78	-2.52 [-5.22; 0.18]	14.9
Grangeiro 2021 (Brazil)	30	35	3M vs 6M	Healthy	13	21	2.59	14.98	19	2.82	15.52	-0.23 [-9.70; 9.24]	10.8
Papakonstantinou 2016 (Greece)	27	27	3M vs 6M	Metabolic	12	40	-3.61	0.83	40	-3.61	1.45	0.00 [-0.52; 0.52]	15.4
Papakonstantinou 2018b (Greece)	52	32	3M vs 6M	Metabolic	12	12	-5.59	5.59	12	-5.77	3.06	0.18 [-3.43; 3.79]	14.5
Forslund 2008 (Sweden)	39	38	3M vs 3M+3S	Healthy	52	70	-2.88	8.29	70	-5.95	14.05	3.07 [-0.75; 6.89]	14.4
Overall Effect						246			244			-5.40 [-17.22; 6.42]	100.0
Heterogeneity: $I^2 = 100\%$, $\tau^2 = 249.6048$, $\chi^2_6 = 1388.36$ ($p < 0.01$)													
**Meal distribution**													
Jakubowicz 2013 (Israel)	46	32	HCB vs HCD	Metabolic	12	38	-10.90	2.45	36	-3.90	2.17	-7.00 [-8.05; -5.95]	25.5
Lombardo 2014 (Italy)	46	36	FL vs EED	Healthy	12	18	-2.52	7.00	18	0.72	5.00	-3.24 [-7.21; 0.73]	23.8
Madjd 2021 (Iran)	35	33	ED vs LD	Healthy	12	40	-8.29	3.24	42	-7.21	3.96	-1.08 [-2.64; 0.48]	25.3
Madjd 2016 (Iran)	34	32	ML vs BL	Healthy	12	40	-7.21	2.97	40	-6.31	2.97	-0.90 [-2.20; 0.40]	25.4
Overall Effect						136			136			-3.06 [-6.73; 0.60]	100.0
Heterogeneity: $I^2 = 95\%$, $\tau^2 = 12.7997$, $\chi^2_3 = 66.29$ ($p < 0.01$)													

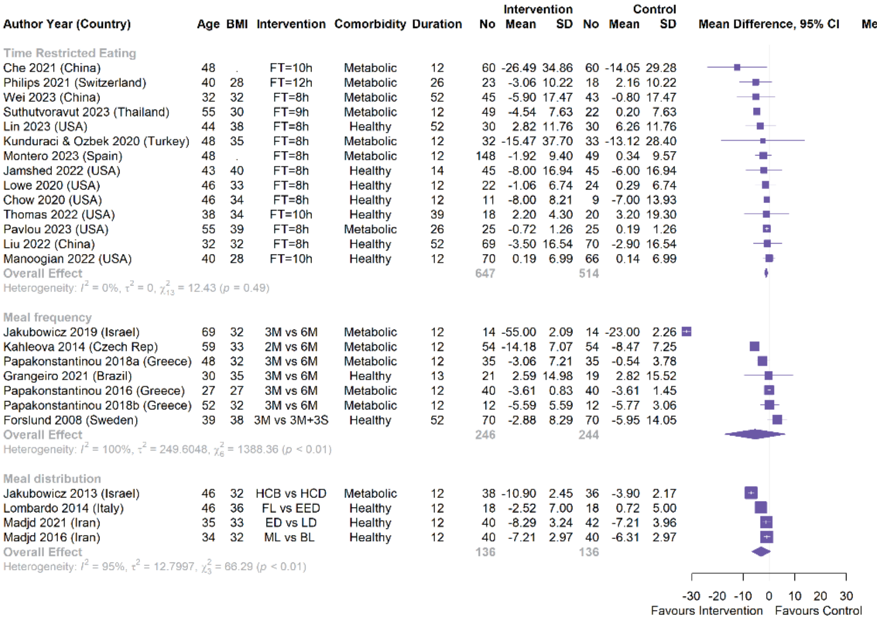

eFigure 30: Meta-analysis of difference in mean difference (95% CIs) for the effect of meal timing interventions on fasting plasma glucose (mg/dL), grouped by the meal timing intervention.

BMI: Body Mass Index; SD: Standard Deviation; CI: Confidence Interval; USA: United States of America; FT: Feeding Time; Duration: follow-up duration in weeks; 2M/3M/6M: 2, 3 or 6 meals; 3M+2S: 3 meals and 2 snacks; 3M+3S: 3 meals and 3 snacks; 10S: Grazing diet – 100kcal every 2-3 hours; HCB: High Calorie Breakfast; HCD: High Calorie Dinner; ED: Early Dinner; LD: Late Dinner; FL: Front Loading: EED: Equal Energy Distribution; ML: Middle Loading; BL: Back Loading.

Author Year (Country)	Age	BMI	Intervention	Comorbidity	Duration	Intervention No	Intervention Mean	Intervention SD	Control No	Control Mean	Control SD	Mean Difference, 95%CI	Weight%
**<80% of participants were Women**													
Pavlou 2023 (USA)	55	39	FT=8h	Metabolic	26	25	-0.72	1.26	25	0.19	1.26	-0.91 [-1.61; -0.21]	1.0
Che 2021 (China)	48	.	FT=10h	Metabolic	12	60	-1.54	1.47	60	-0.66	1.24	-0.88 [-1.37; -0.39]	1.9
Manoogian 2022 (USA)	40	28	FT=10h	Healthy	12	70	-0.04	0.27	66	0.27	0.24	-0.31 [-0.39; -0.22]	10.5
Suthutvoravut 2023 (Thailand)	55	30	FT=9h	Metabolic	12	49	-0.89	0.43	22	-0.65	0.43	-0.24 [-0.46; -0.02]	5.9
Wei 2023 (China)	32	32	FT=8h	Metabolic	52	45	-0.20	0.48	43	-0.10	0.48	-0.10 [-0.30; 0.10]	6.4
Montero 2023 (Spain)	48	.	FT=8h	Metabolic	12	148	0.00	0.25	49	0.05	0.32	-0.05 [-0.15; 0.05]	10.0
Lowe 2020 (USA)	46	33	FT=8h	Healthy	12	22	-0.02	0.18	24	-0.01	0.18	-0.02 [-0.12; 0.08]	9.9
Kunduraci & Ozbek 2020 (Turkey)	48	35	FT=8h	Metabolic	12	32	-0.32	0.07	33	-0.31	0.18	-0.01 [-0.08; 0.06]	11.2
Philips 2021 (Switzerland)	40	28	FT=12h	Metabolic	26	23	0.03	0.18	18	-0.09	0.18	0.12 [0.01; 0.23]	9.6
Overall Effect						474			340			-0.14 [-0.26; -0.02]	66.4
Heterogeneity: $I^2 = 87\%$, $\tau^2 = 0.0259$, $\chi^2_8 = 63.58$ ($p < 0.01$)													
**≥80% of participants were Women**													
Lin 2023 (USA)	44	38	FT=8h	Healthy	52	30	-0.01	0.34	30	0.07	0.34	-0.08 [-0.25; 0.09]	7.3
Chow 2020 (USA)	46	34	FT=8h	Healthy	12	11	-0.00	0.01	9	0.00	0.01	-0.00 [-0.01; 0.00]	12.3
Jamshed 2022 (USA)	43	40	FT=8h	Healthy	14	45	-0.10	0.48	45	-0.10	0.48	0.00 [-0.20; 0.20]	6.4
Thomas 2022 (USA)	38	34	FT=10h	Healthy	39	18	0.00	0.20	20	0.00	0.30	0.00 [-0.16; 0.16]	7.7
Overall Effect						104			104			-0.00 [-0.01; 0.00]	33.6
Heterogeneity: $I^2 = 0\%$, $\tau^2 = 0$, $\chi^2_3 = 0.83$ ($p = 0.84$)													
**Overall Effect**						**578**			**444**			**-0.08 [-0.15; -0.01]**	**100.0**
Heterogeneity: $I^2 = 85\%$, $\tau^2 = 0.0112$, $\chi^2_{12} = 79.12$ ($p < 0.01$)													
Test for overall effect: $z = -2.18$ ($p = 0.03$)													
Test for subgroup differences: $\chi^2_1 = 4.67$, df = 1 ($p = 0.03$)													

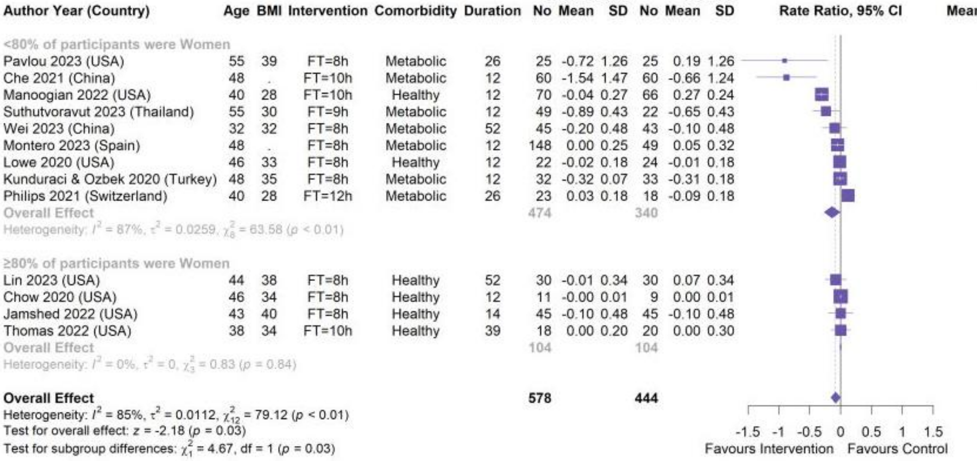

eFigure 31: Meta-analysis of difference in mean difference (95% CIs) for the effect of time-restricted eating on HbA1c (%), grouped by gender proportion.

BMI: Body Mass Index; SD: Standard Deviation; CI: Confidence Interval; USA: United States of America; FT: Feeding Time; Duration: follow-up duration in weeks; 2M/3M/6M: 2, 3 or 6 meals; 3M+2S: 3 meals and 2 snacks; 3M+3S: 3 meals and 3 snacks; 10S: Grazing diet – 100kcal every 2-3 hours; HCB: High Calorie Breakfast; HCD: High Calorie Dinner; ED: Early Dinner; LD: Late Dinner; FL: Front Loading: EED: Equal Energy Distribution; ML: Middle Loading; BL: Back Loading.

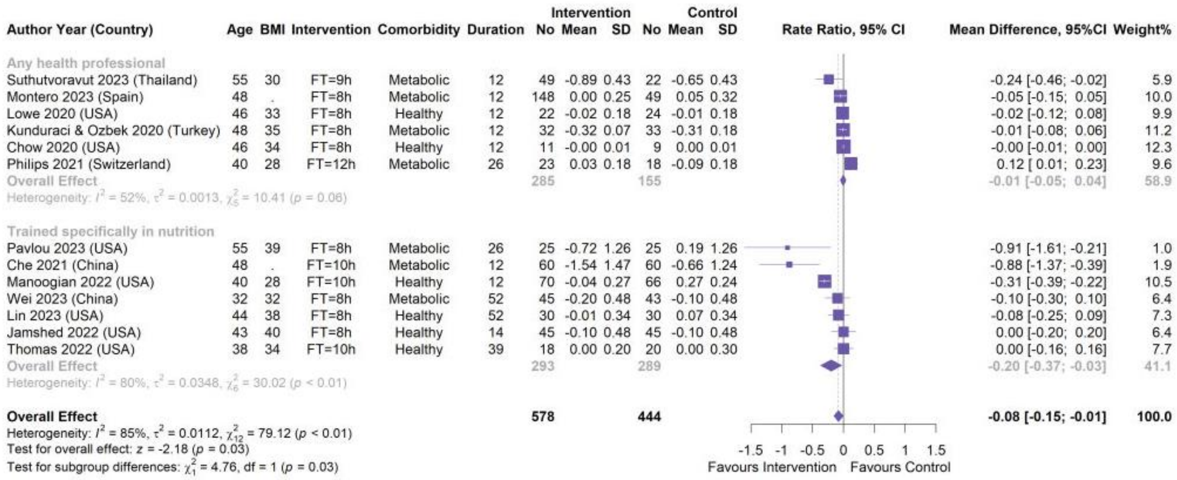

eFigure 32: Meta-analysis of difference in mean difference (95% CIs) for the effect of time-restricted eating on HbA1c (%), grouped by delivery personnel.

BMI: Body Mass Index; SD: Standard Deviation; CI: Confidence Interval; USA: United States of America; FT: Feeding Time; Duration: follow-up duration in weeks; 2M/3M/6M: 2, 3 or 6 meals; 3M+2S: 3 meals and 2 snacks; 3M+3S: 3 meals and 3 snacks; 10S: Grazing diet – 100kcal every 2-3 hours; HCB: High Calorie Breakfast; HCD: High Calorie Dinner; ED: Early Dinner; LD: Late Dinner; FL: Front Loading: EED: Equal Energy Distribution; ML: Middle Loading; BL: Back Loading.

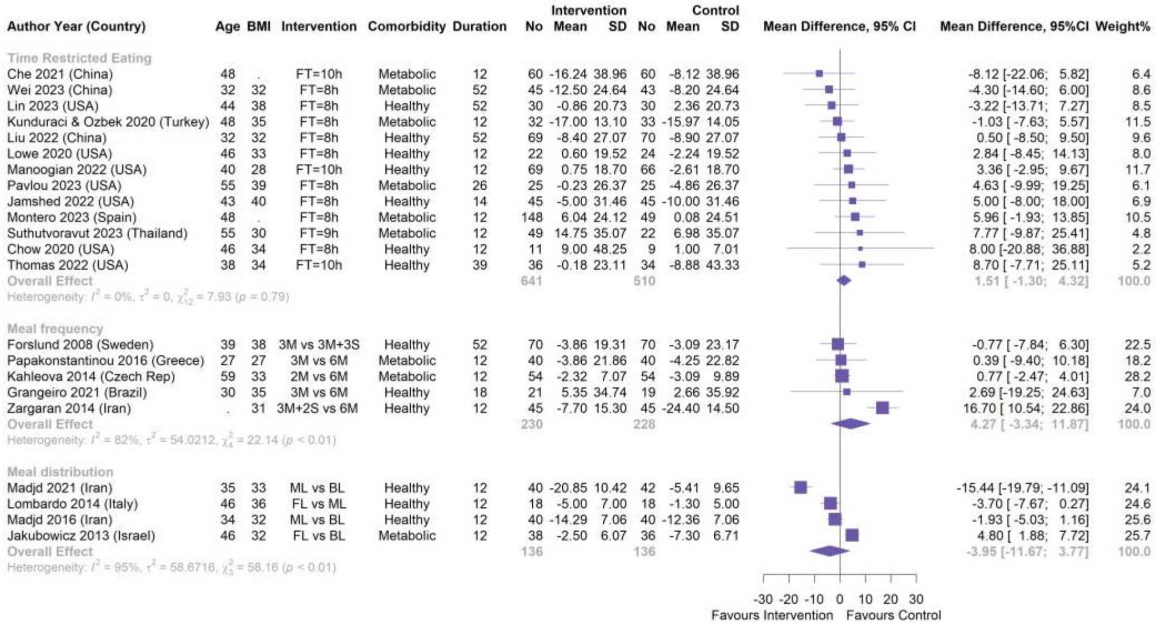

eFigure 33: Meta-analysis of difference in mean difference (95% CIs) for the effect of meal timing interventions on LDL (mg/dL), grouped by the meal timing intervention.

BMI: Body Mass Index; SD: Standard Deviation; CI: Confidence Interval; USA: United States of America; FT: Feeding Time; Duration: follow-up duration in weeks; 2M/3M/6M: 2, 3 or 6 meals; 3M+2S: 3 meals and 2 snacks; 3M+3S: 3 meals and 3 snacks; 10S: Grazing diet – 100kcal every 2-3 hours; HCB: High Calorie Breakfast; HCD: High Calorie Dinner; ED: Early Dinner; LD: Late Dinner; FL: Front Loading: EED: Equal Energy Distribution; ML: Middle Loading; BL: Back Loading.

© 2024 Liu HY et al. *JAMA Network Open.*

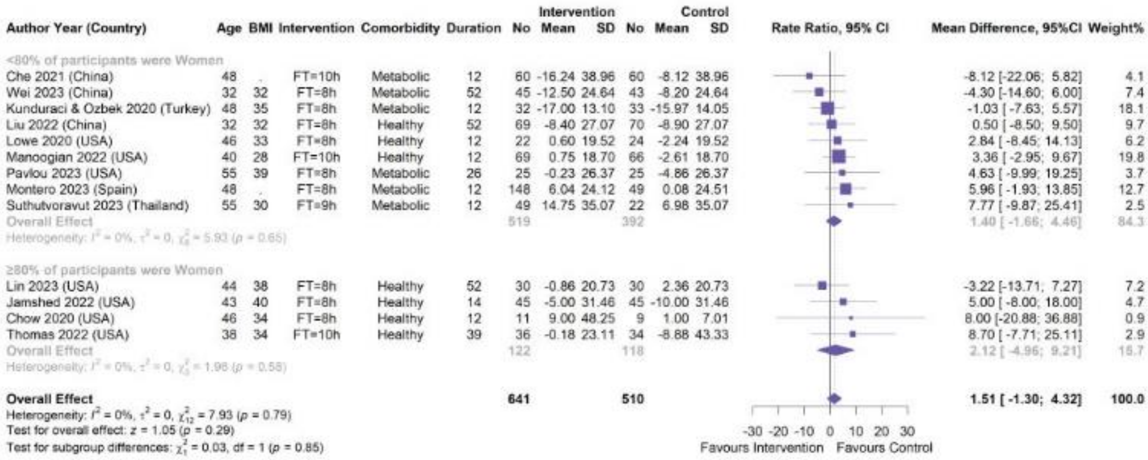

eFigure 34: Meta-analysis of difference in mean difference (95% CIs) for the effect of time-restricted eating on LDL (mg/dL), grouped by gender proportion.

BMI: Body Mass Index; SD: Standard Deviation; CI: Confidence Interval; USA: United States of America; FT: Feeding Time; Duration: follow-up duration in weeks; 2M/3M/6M: 2, 3 or 6 meals; 3M+2S: 3 meals and 2 snacks; 3M+3S: 3 meals and 3 snacks; 10S: Grazing diet – 100kcal every 2-3 hours; HCB: High Calorie Breakfast; HCD: High Calorie Dinner; ED: Early Dinner; LD: Late Dinner; FL: Front Loading: EED: Equal Energy Distribution; ML: Middle Loading; BL: Back Loading.

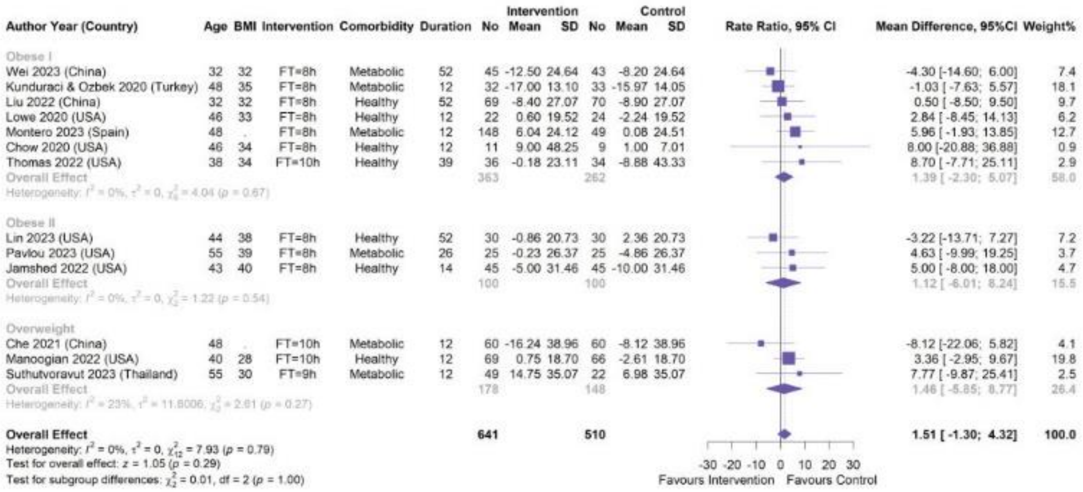

eFigure 35: Meta-analysis of difference in mean difference (95% CIs) for the effect of time-restricted eating on LDL (mg/dL), grouped by baseline BMI status.

BMI: Body Mass Index; SD: Standard Deviation; CI: Confidence Interval; USA: United States of America; FT: Feeding Time; Duration: follow-up duration in weeks; 2M/3M/6M: 2, 3 or 6 meals; 3M+2S: 3 meals and 2 snacks; 3M+3S: 3 meals and 3 snacks; 10S: Grazing diet – 100kcal every 2-3 hours; HCB: High Calorie Breakfast; HCD: High Calorie Dinner; ED: Early Dinner; LD: Late Dinner; FL: Front Loading: EED: Equal Energy Distribution; ML: Middle Loading; BL: Back Loading.

© 2024 Liu HY et al. *JAMA Network Open.*

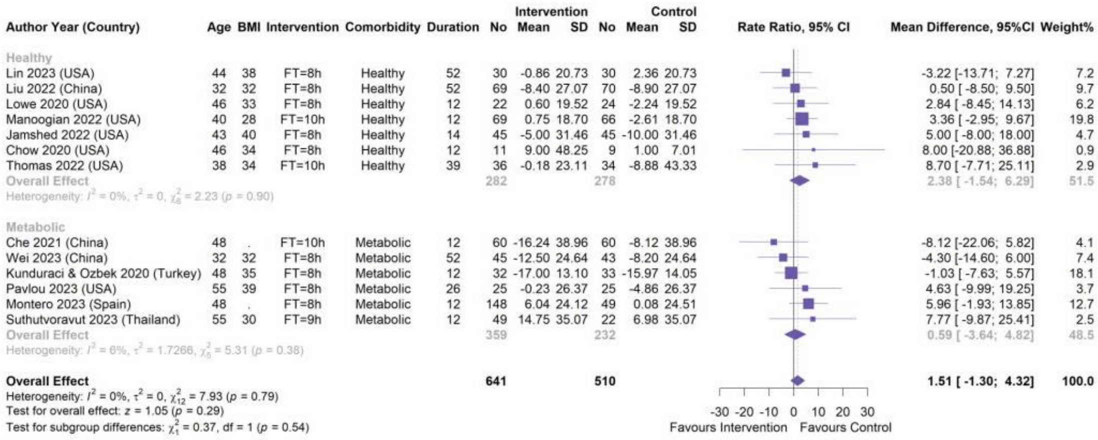

eFigure 36: Meta-analysis of difference in mean difference (95% CIs) for the effect of time-restricted eating on LDL (mg/dL), grouped by health status.

BMI: Body Mass Index; SD: Standard Deviation; CI: Confidence Interval; USA: United States of America; FT: Feeding Time; Duration: follow-up duration in weeks; 2M/3M/6M: 2, 3 or 6 meals; 3M+2S: 3 meals and 2 snacks; 3M+3S: 3 meals and 3 snacks; 10S: Grazing diet – 100kcal every 2-3 hours; HCB: High Calorie Breakfast; HCD: High Calorie Dinner; ED: Early Dinner; LD: Late Dinner; FL: Front Loading: EED: Equal Energy Distribution; ML: Middle Loading; BL: Back Loading.

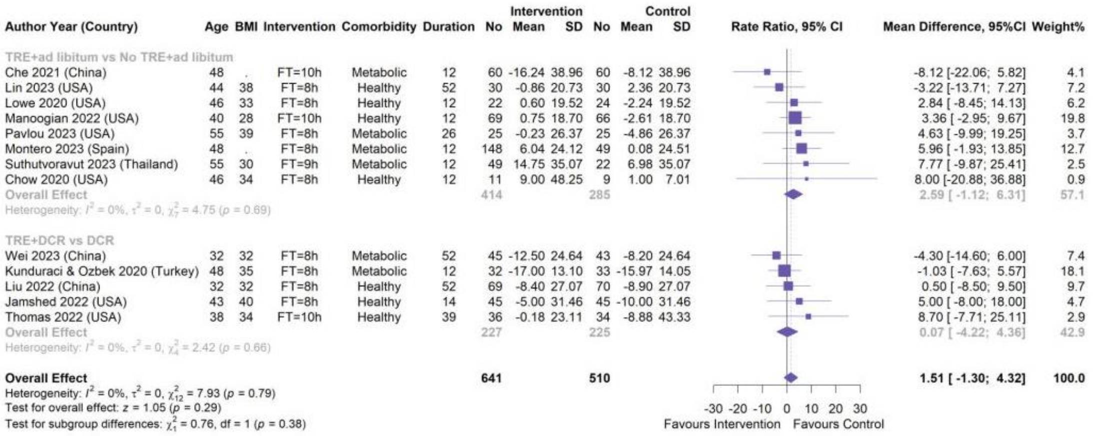

eFigure 37: Meta-analysis of difference in mean difference (95% CIs) for the effect of time-restricted eating on LDL (mg/dL), grouped by energy prescription.

BMI: Body Mass Index; SD: Standard Deviation; CI: Confidence Interval; USA: United States of America; FT: Feeding Time; Duration: follow-up duration in weeks; 2M/3M/6M: 2, 3 or 6 meals; 3M+2S: 3 meals and 2 snacks; 3M+3S: 3 meals and 3 snacks; 10S: Grazing diet – 100kcal every 2-3 hours; HCB: High Calorie Breakfast; HCD: High Calorie Dinner; ED: Early Dinner; LD: Late Dinner; FL: Front Loading: EED: Equal Energy Distribution; ML: Middle Loading; BL: Back Loading.

© 2024 Liu HY et al. *JAMA Network Open.*

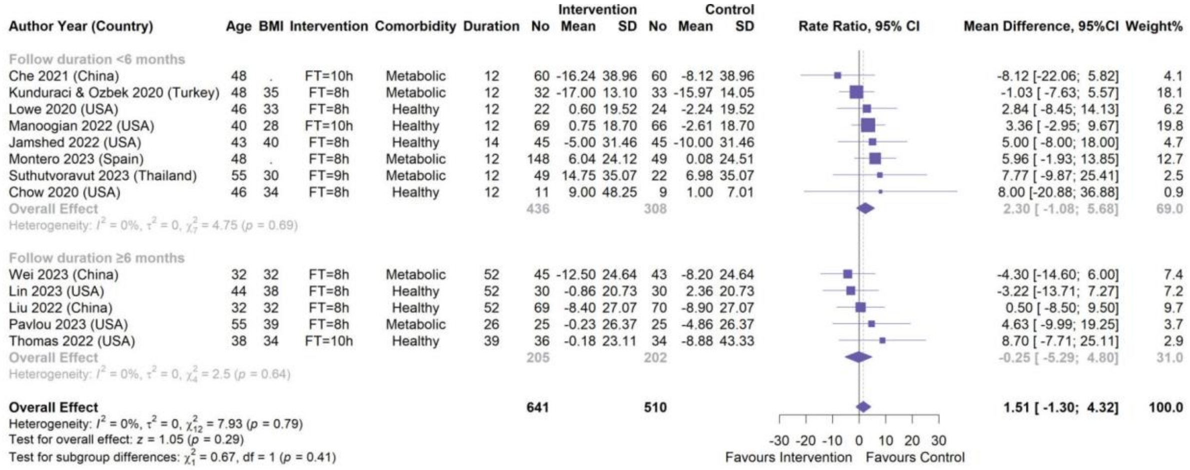

eFigure 38: Meta-analysis of difference in mean difference (95% CIs) for the effect of time-restricted eating on LDL (mg/dL), grouped by follow duration.

BMI: Body Mass Index; SD: Standard Deviation; CI: Confidence Interval; USA: United States of America; FT: Feeding Time; Duration: follow-up duration in weeks; 2M/3M/6M: 2, 3 or 6 meals; 3M+2S: 3 meals and 2 snacks; 3M+3S: 3 meals and 3 snacks; 10S: Grazing diet – 100kcal every 2-3 hours; HCB: High Calorie Breakfast; HCD: High Calorie Dinner; ED: Early Dinner; LD: Late Dinner; FL: Front Loading: EED: Equal Energy Distribution; ML: Middle Loading; BL: Back Loading.

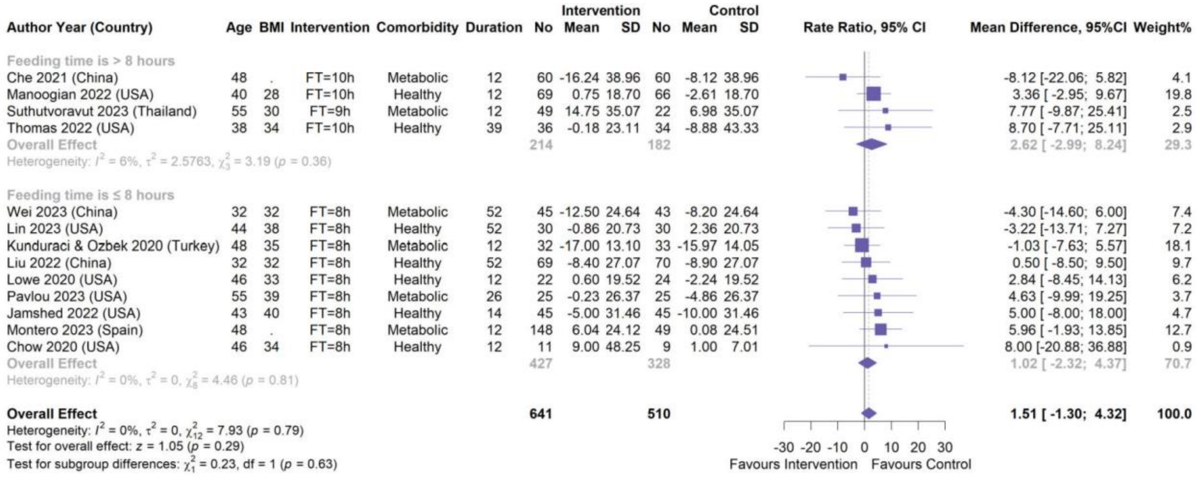

eFigure 39: Meta-analysis of difference in mean difference (95% CIs) for the effect of time-restricted eating on LDL (mg/dL), grouped by eating window.

BMI: Body Mass Index; SD: Standard Deviation; CI: Confidence Interval; USA: United States of America; FT: Feeding Time; Duration: follow-up duration in weeks; 2M/3M/6M: 2, 3 or 6 meals; 3M+2S: 3 meals and 2 snacks; 3M+3S: 3 meals and 3 snacks; 10S: Grazing diet – 100kcal every 2-3 hours; HCB: High Calorie Breakfast; HCD: High Calorie Dinner; ED: Early Dinner; LD: Late Dinner; FL: Front Loading: EED: Equal Energy Distribution; ML: Middle Loading; BL: Back Loading.

© 2024 Liu HY et al. *JAMA Network Open.*

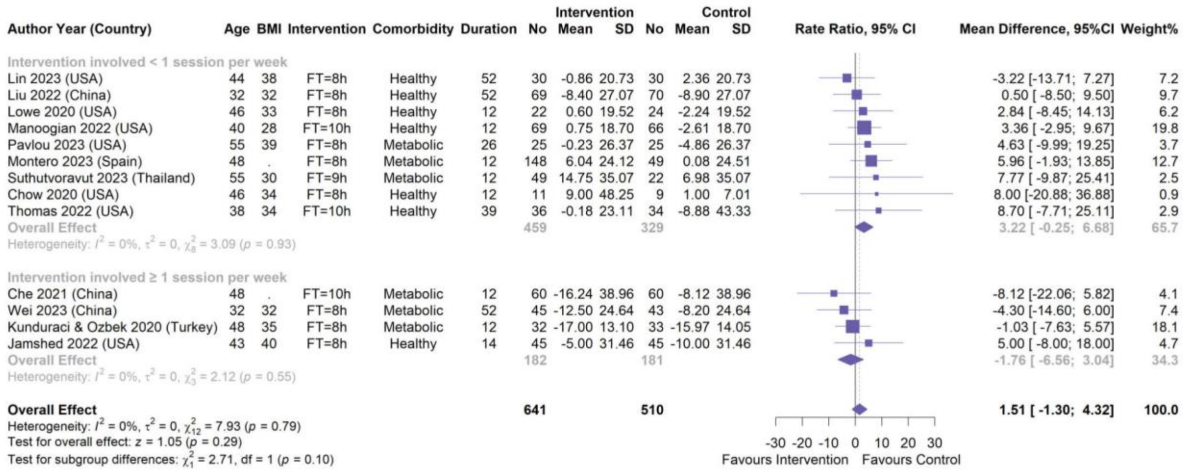

eFigure 40: Meta-analysis of difference in mean difference (95% CIs) for the effect of time-restricted eating on LDL (mg/dL), grouped by frequency of contact.

BMI: Body Mass Index; SD: Standard Deviation; CI: Confidence Interval; USA: United States of America; FT: Feeding Time; Duration: follow-up duration in weeks; 2M/3M/6M: 2, 3 or 6 meals; 3M+2S: 3 meals and 2 snacks; 3M+3S: 3 meals and 3 snacks; 10S: Grazing diet – 100kcal every 2-3 hours; HCB: High Calorie Breakfast; HCD: High Calorie Dinner; ED: Early Dinner; LD: Late Dinner; FL: Front Loading: EED: Equal Energy Distribution; ML: Middle Loading; BL: Back Loading.

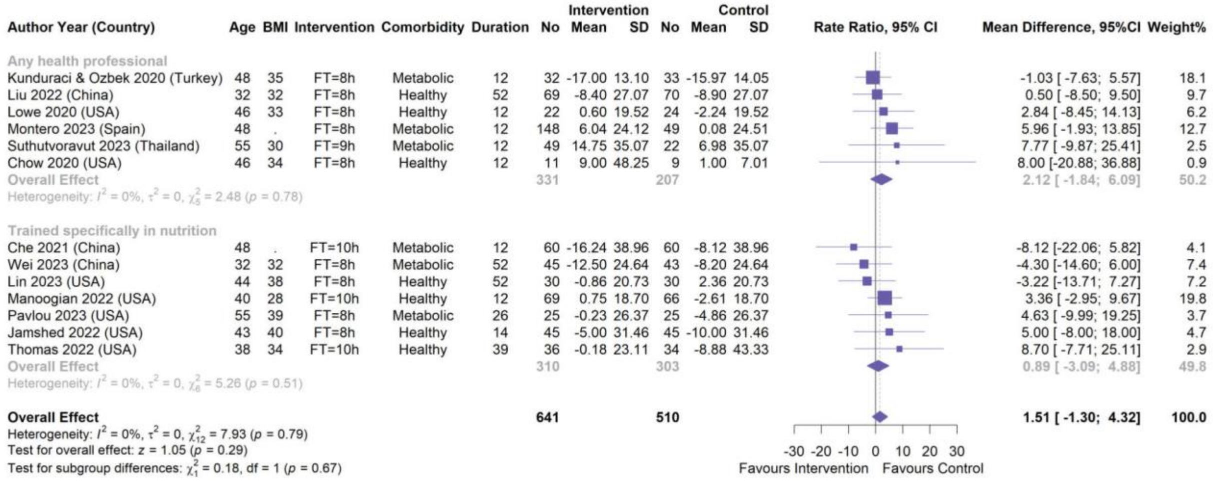

eFigure 41: Meta-analysis of difference in mean difference (95% CIs) for the effect of time-restricted eating on LDL (mg/dL), grouped by delivery personnel.

BMI: Body Mass Index; SD: Standard Deviation; CI: Confidence Interval; USA: United States of America; FT: Feeding Time; Duration: follow-up duration in weeks; 2M/3M/6M: 2, 3 or 6 meals; 3M+2S: 3 meals and 2 snacks; 3M+3S: 3 meals and 3 snacks; 10S: Grazing diet – 100kcal every 2-3 hours; HCB: High Calorie Breakfast; HCD: High Calorie Dinner; ED: Early Dinner; LD: Late Dinner; FL: Front Loading: EED: Equal Energy Distribution; ML: Middle Loading; BL: Back Loading.

© 2024 Liu HY et al. *JAMA Network Open.*

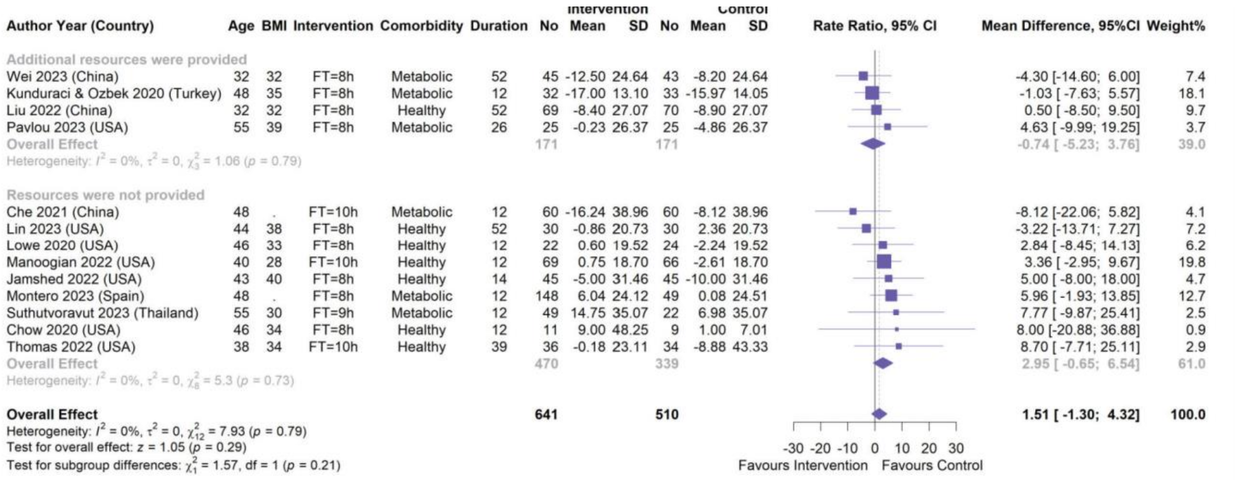

eFigure 42: Meta-analysis of difference in mean difference (95% CIs) for the effect of time-restricted eating on LDL (mg/dL), grouped by resource provision.

BMI: Body Mass Index; SD: Standard Deviation; CI: Confidence Interval; USA: United States of America; FT: Feeding Time; Duration: follow-up duration in weeks; 2M/3M/6M: 2, 3 or 6 meals; 3M+2S: 3 meals and 2 snacks; 3M+3S: 3 meals and 3 snacks; 10S: Grazing diet – 100kcal every 2-3 hours; HCB: High Calorie Breakfast; HCD: High Calorie Dinner; ED: Early Dinner; LD: Late Dinner; FL: Front Loading: EED: Equal Energy Distribution; ML: Middle Loading; BL: Back Loading.

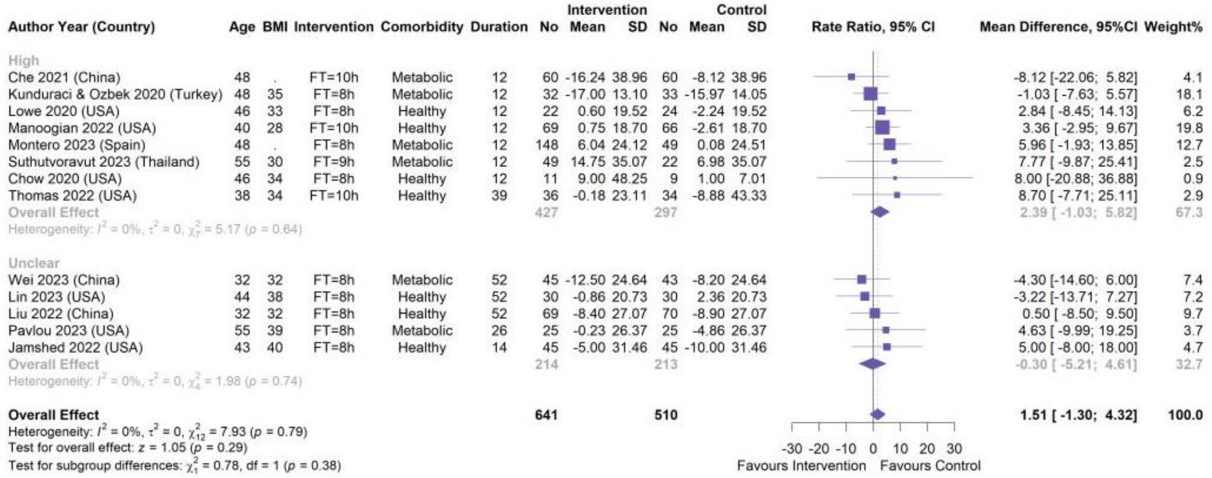

eFigure 43: Meta-analysis of difference in mean difference (95% CIs) for the effect of time-restricted eating on LDL (mg/dL), grouped by risk of bias.

BMI: Body Mass Index; SD: Standard Deviation; CI: Confidence Interval; USA: United States of America; FT: Feeding Time; Duration: follow-up duration in weeks; 2M/3M/6M: 2, 3 or 6 meals; 3M+2S: 3 meals and 2 snacks; 3M+3S: 3 meals and 3 snacks; 10S: Grazing diet – 100kcal every 2-3 hours; HCB: High Calorie Breakfast; HCD: High Calorie Dinner; ED: Early Dinner; LD: Late Dinner; FL: Front Loading: EED: Equal Energy Distribution; ML: Middle Loading; BL: Back Loading.

© 2024 Liu HY et al. *JAMA Network Open.*

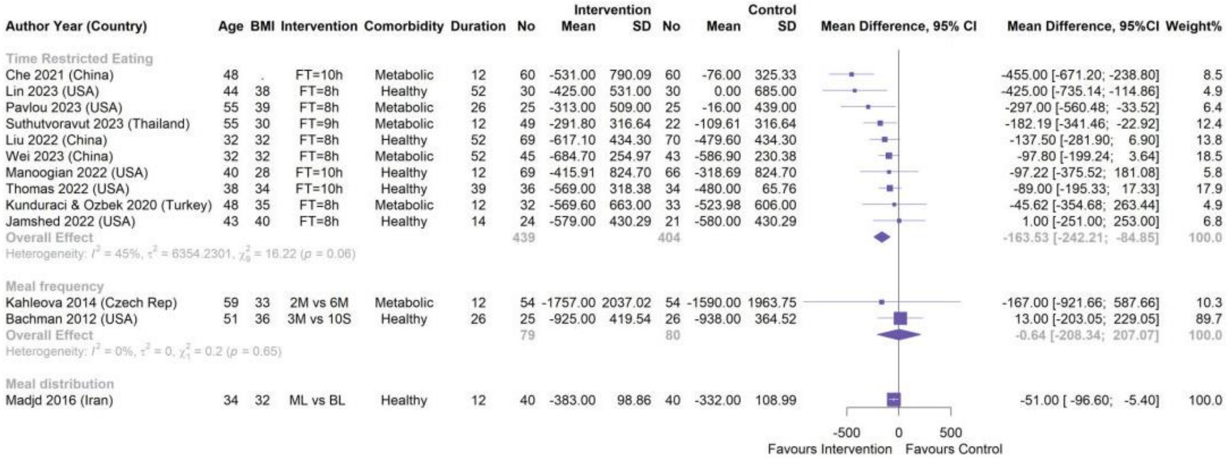

eFigure 44: Meta-analysis of difference in mean difference (95% CIs) for the effect of meal timing interventions on energy intake (kcal/day), grouped by the meal timing intervention.

BMI: Body Mass Index; SD: Standard Deviation; CI: Confidence Interval; USA: United States of America; FT: Feeding Time; Duration: follow-up duration in weeks; 2M/3M/6M: 2, 3 or 6 meals; 3M+2S: 3 meals and 2 snacks; 3M+3S: 3 meals and 3 snacks; 10S: Grazing diet – 100kcal every 2-3 hours; HCB: High Calorie Breakfast; HCD: High Calorie Dinner; ED: Early Dinner; LD: Late Dinner; FL: Front Loading: EED: Equal Energy Distribution; ML: Middle Loading; BL: Back Loading.

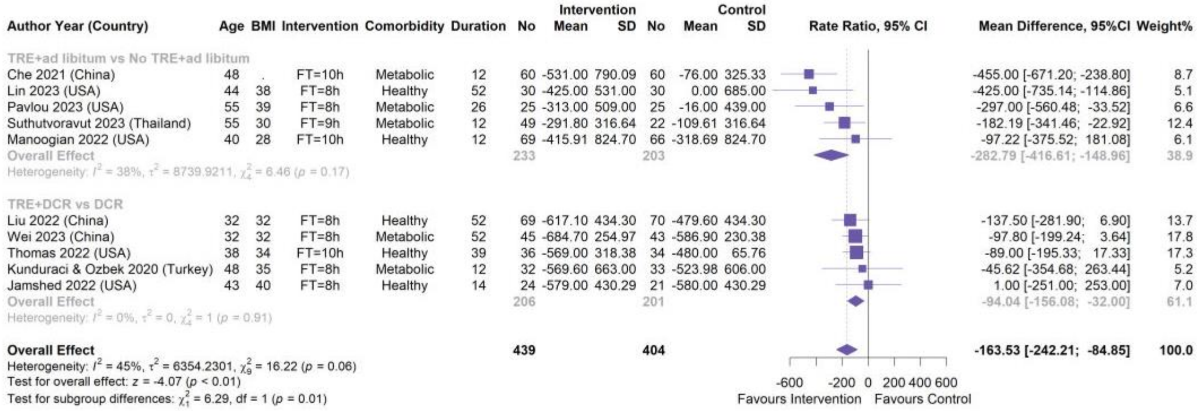

eFigure 45: Meta-analysis of difference in mean difference (95% CIs) for the effect of time-restricted eating on energy intake (kcal/day), grouped by energy prescription.

BMI: Body Mass Index; SD: Standard Deviation; CI: Confidence Interval; USA: United States of America; FT: Feeding Time; Duration: follow-up duration in weeks; 2M/3M/6M: 2, 3 or 6 meals; 3M+2S: 3 meals and 2 snacks; 3M+3S: 3 meals and 3 snacks; 10S: Grazing diet – 100kcal every 2-3 hours; HCB: High Calorie Breakfast; HCD: High Calorie Dinner; ED: Early Dinner; LD: Late Dinner; FL: Front Loading: EED: Equal Energy Distribution; ML: Middle Loading; BL: Back Loading.

© 2024 Liu HY et al. *JAMA Network Open.*

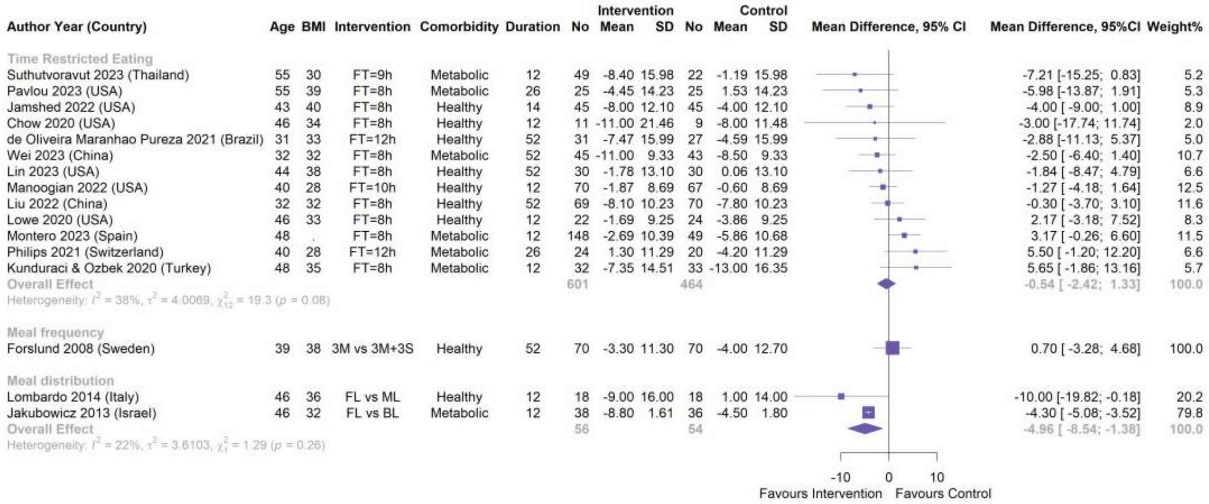

eFigure 46: Meta-analysis of difference in mean difference (95% CIs) for the effect of meal timing interventions on systolic blood pressure (mmHg), grouped by the meal timing intervention.

BMI: Body Mass Index; SD: Standard Deviation; CI: Confidence Interval; USA: United States of America; FT: Feeding Time; Duration: follow-up duration in weeks; 2M/3M/6M: 2, 3 or 6 meals; 3M+2S: 3 meals and 2 snacks; 3M+3S: 3 meals and 3 snacks; 10S: Grazing diet – 100kcal every 2-3 hours; HCB: High Calorie Breakfast; HCD: High Calorie Dinner; ED: Early Dinner; LD: Late Dinner; FL: Front Loading: EED: Equal Energy Distribution; ML: Middle Loading; BL: Back Loading.

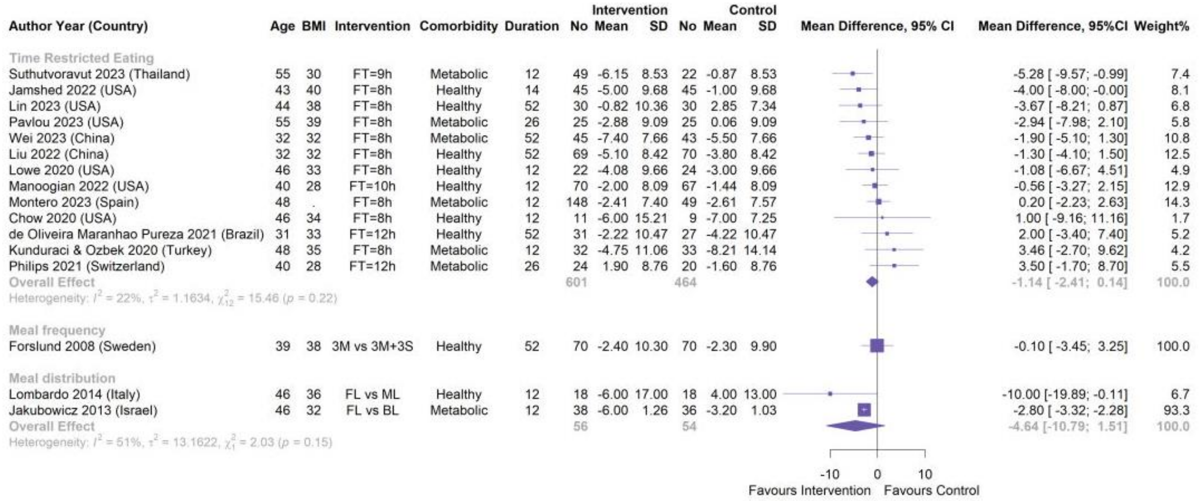

eFigure 47: Meta-analysis of difference in mean difference (95% CIs) for the effect of meal timing interventions on diastolic blood pressure (mmHg), grouped by the meal timing intervention.

BMI: Body Mass Index; SD: Standard Deviation; CI: Confidence Interval; USA: United States of America; FT: Feeding Time; Duration: follow-up duration in weeks; 2M/3M/6M: 2, 3 or 6 meals; 3M+2S: 3 meals and 2 snacks; 3M+3S: 3 meals and 3 snacks; 10S: Grazing diet – 100kcal every 2-3 hours; HCB: High Calorie Breakfast; HCD: High Calorie Dinner; ED: Early Dinner; LD: Late Dinner; FL: Front Loading: EED: Equal Energy Distribution; ML: Middle Loading; BL: Back Loading.

© 2024 Liu HY et al. *JAMA Network Open.*

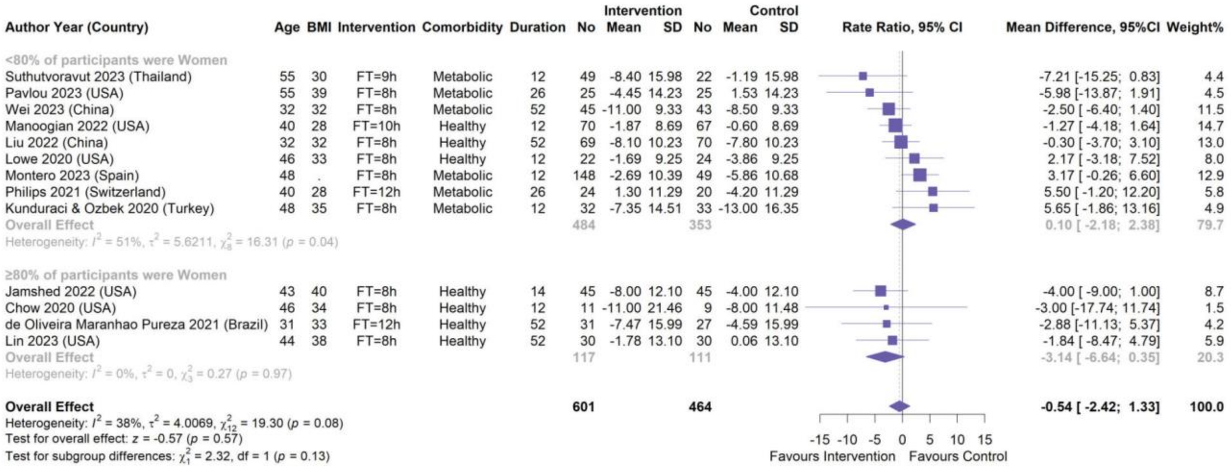

eFigure 48: Meta-analysis of difference in mean difference (95% CIs) for the effect of time-restricted eating on systolic blood pressure (mmHg), grouped by gender proportion.

BMI: Body Mass Index; SD: Standard Deviation; CI: Confidence Interval; USA: United States of America; FT: Feeding Time; Duration: follow-up duration in weeks; 2M/3M/6M: 2, 3 or 6 meals; 3M+2S: 3 meals and 2 snacks; 3M+3S: 3 meals and 3 snacks; 10S: Grazing diet – 100kcal every 2-3 hours; HCB: High Calorie Breakfast; HCD: High Calorie Dinner; ED: Early Dinner; LD: Late Dinner; FL: Front Loading: EED: Equal Energy Distribution; ML: Middle Loading; BL: Back Loading.

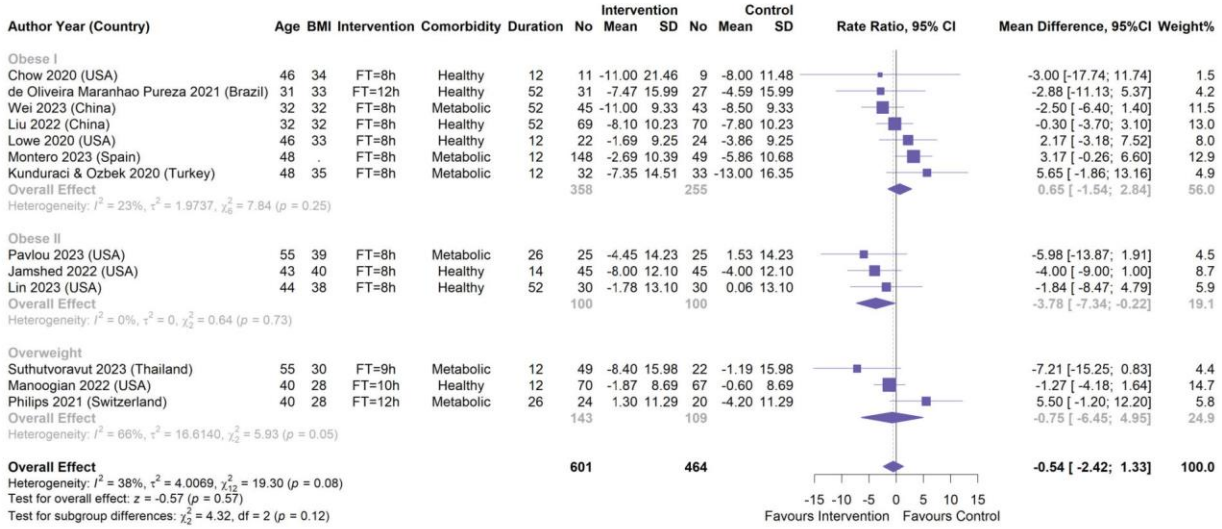

eFigure 49: Meta-analysis of difference in mean difference (95% CIs) for the effect of time-restricted eating on systolic blood pressure (mmHg), grouped by baseline BMI status.

BMI: Body Mass Index; SD: Standard Deviation; CI: Confidence Interval; USA: United States of America; FT: Feeding Time; Duration: follow-up duration in weeks; 2M/3M/6M: 2, 3 or 6 meals; 3M+2S: 3 meals and 2 snacks; 3M+3S: 3 meals and 3 snacks; 10S: Grazing diet – 100kcal every 2-3 hours; HCB: High Calorie Breakfast; HCD: High Calorie Dinner; ED: Early Dinner; LD: Late Dinner; FL: Front Loading: EED: Equal Energy Distribution; ML: Middle Loading; BL: Back Loading.

© 2024 Liu HY et al. *JAMA Network Open.*

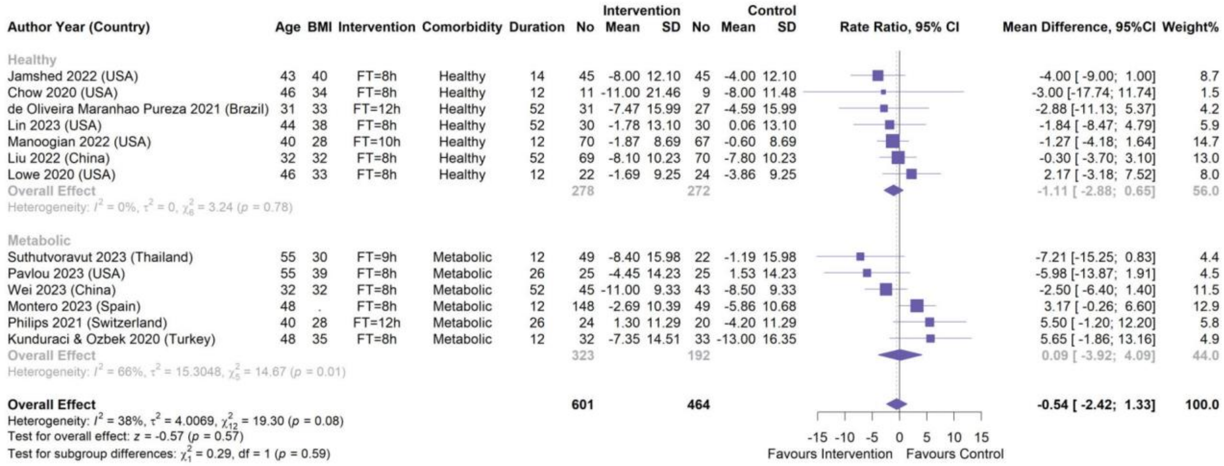

eFigure 50: Meta-analysis of difference in mean difference (95% CIs) for the effect of time-restricted eating on systolic blood pressure (mmHg), grouped by health status.

BMI: Body Mass Index; SD: Standard Deviation; CI: Confidence Interval; USA: United States of America; FT: Feeding Time; Duration: follow-up duration in weeks; 2M/3M/6M: 2, 3 or 6 meals; 3M+2S: 3 meals and 2 snacks; 3M+3S: 3 meals and 3 snacks; 10S: Grazing diet – 100kcal every 2-3 hours; HCB: High Calorie Breakfast; HCD: High Calorie Dinner; ED: Early Dinner; LD: Late Dinner; FL: Front Loading: EED: Equal Energy Distribution; ML: Middle Loading; BL: Back Loading.

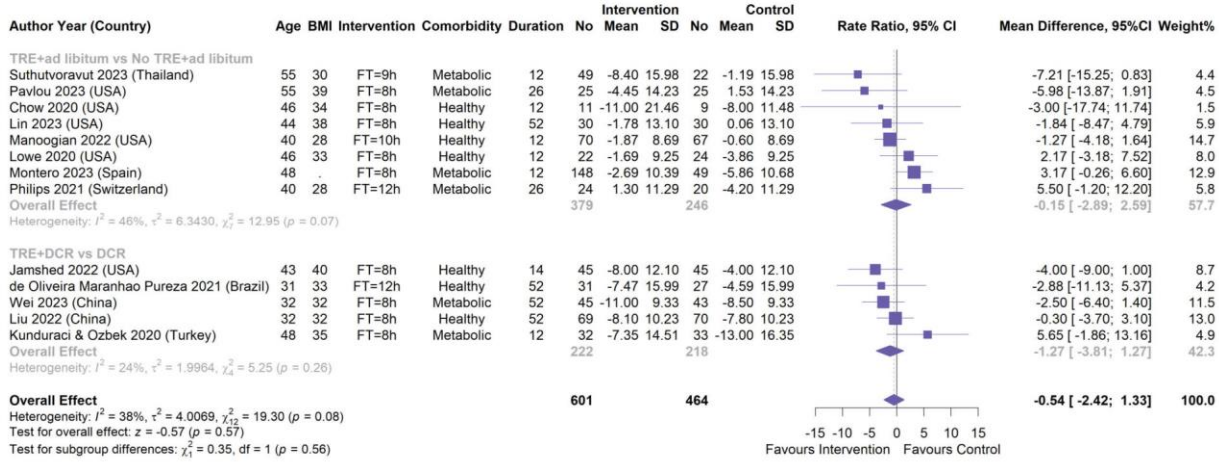

eFigure 51: Meta-analysis of difference in mean difference (95% CIs) for the effect of time-restricted eating on systolic blood pressure (mmHg), grouped by energy prescription.

BMI: Body Mass Index; SD: Standard Deviation; CI: Confidence Interval; USA: United States of America; FT: Feeding Time; Duration: follow-up duration in weeks; 2M/3M/6M: 2, 3 or 6 meals; 3M+2S: 3 meals and 2 snacks; 3M+3S: 3 meals and 3 snacks; 10S: Grazing diet – 100kcal every 2-3 hours; HCB: High Calorie Breakfast; HCD: High Calorie Dinner; ED: Early Dinner; LD: Late Dinner; FL: Front Loading: EED: Equal Energy Distribution; ML: Middle Loading; BL: Back Loading.

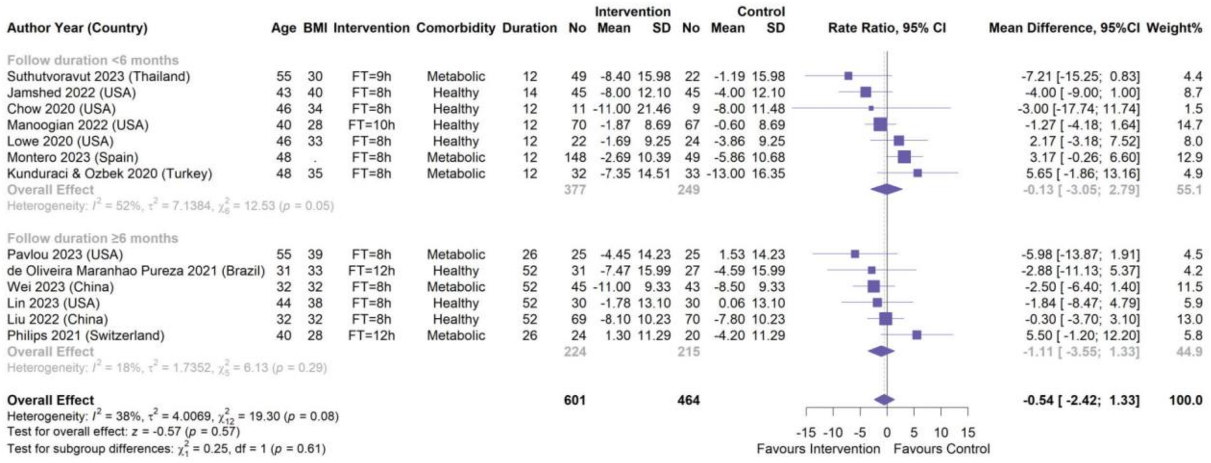

eFigure 52: Meta-analysis of difference in mean difference (95% CIs) for the effect of time-restricted eating on systolic blood pressure (mmHg), grouped by follow duration.

BMI: Body Mass Index; SD: Standard Deviation; CI: Confidence Interval; USA: United States of America; FT: Feeding Time; Duration: follow-up duration in weeks; 2M/3M/6M: 2, 3 or 6 meals; 3M+2S: 3 meals and 2 snacks; 3M+3S: 3 meals and 3 snacks; 10S: Grazing diet – 100kcal every 2-3 hours; HCB: High Calorie Breakfast; HCD: High Calorie Dinner; ED: Early Dinner; LD: Late Dinner; FL: Front Loading: EED: Equal Energy Distribution; ML: Middle Loading; BL: Back Loading.

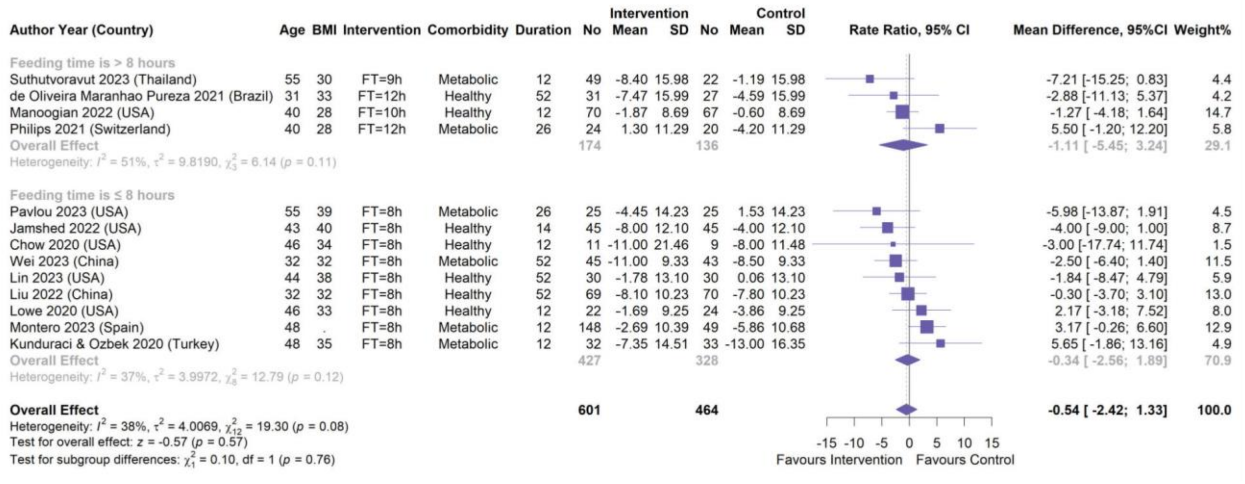

eFigure 53: Meta-analysis of difference in mean difference (95% CIs) for the effect of time-restricted eating on systolic blood pressure (mmHg), grouped by eating window.

BMI: Body Mass Index; SD: Standard Deviation; CI: Confidence Interval; USA: United States of America; FT: Feeding Time; Duration: follow-up duration in weeks; 2M/3M/6M: 2, 3 or 6 meals; 3M+2S: 3 meals and 2 snacks; 3M+3S: 3 meals and 3 snacks; 10S: Grazing diet – 100kcal every 2-3 hours; HCB: High Calorie Breakfast; HCD: High Calorie Dinner; ED: Early Dinner; LD: Late Dinner; FL: Front Loading: EED: Equal Energy Distribution; ML: Middle Loading; BL: Back Loading.

© 2024 Liu HY et al. *JAMA Network Open.*

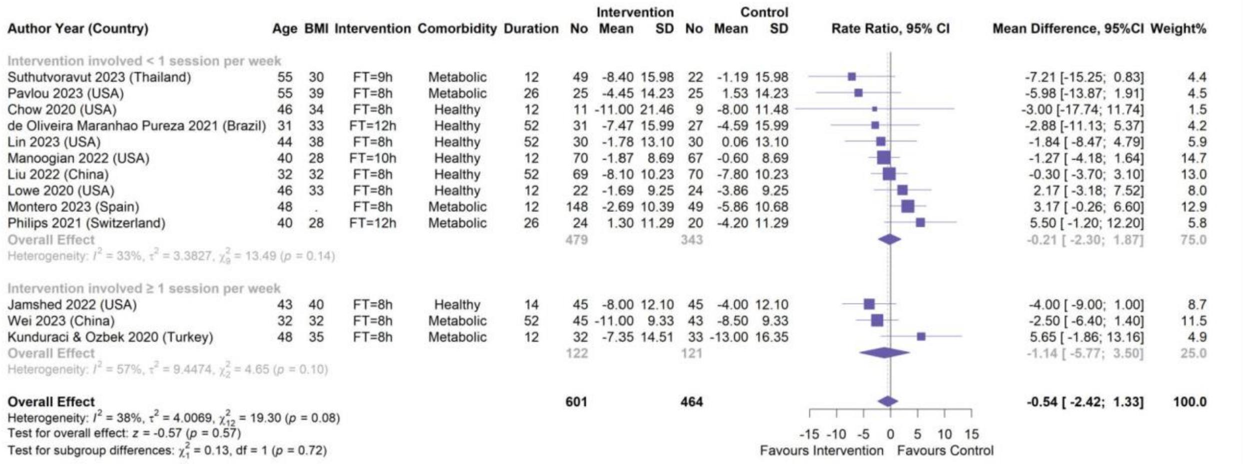

eFigure 54: Meta-analysis of difference in mean difference (95% CIs) for the effect of time-restricted eating on systolic blood pressure (mmHg), grouped by frequency of contact.

BMI: Body Mass Index; SD: Standard Deviation; CI: Confidence Interval; USA: United States of America; FT: Feeding Time; Duration: follow-up duration in weeks; 2M/3M/6M: 2, 3 or 6 meals; 3M+2S: 3 meals and 2 snacks; 3M+3S: 3 meals and 3 snacks; 10S: Grazing diet – 100kcal every 2-3 hours; HCB: High Calorie Breakfast; HCD: High Calorie Dinner; ED: Early Dinner; LD: Late Dinner; FL: Front Loading: EED: Equal Energy Distribution; ML: Middle Loading; BL: Back Loading.

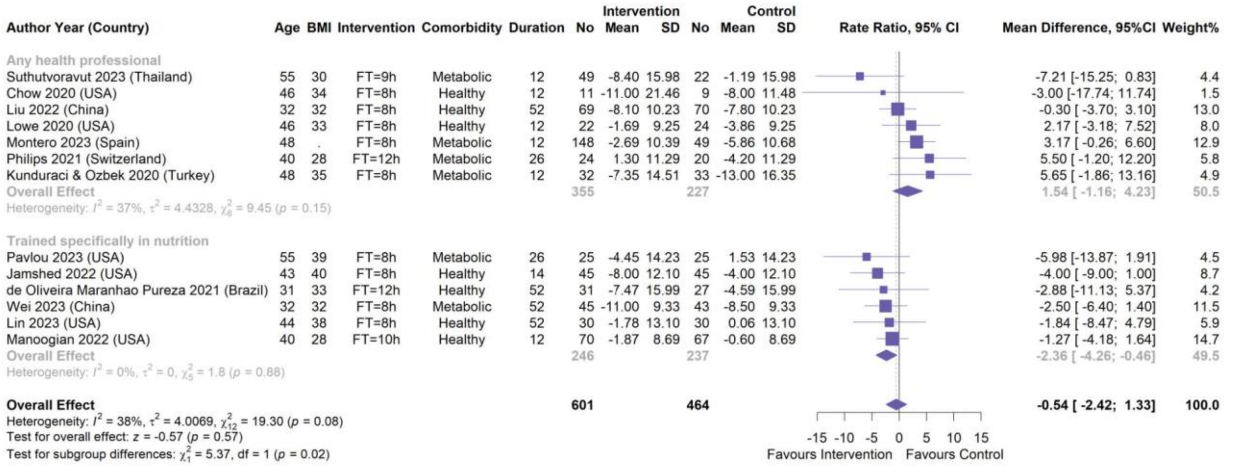

eFigure 55: Meta-analysis of difference in mean difference (95% CIs) for the effect of time-restricted eating on systolic blood pressure (mmHg), grouped by delivery personnel.

BMI: Body Mass Index; SD: Standard Deviation; CI: Confidence Interval; USA: United States of America; FT: Feeding Time; Duration: follow-up duration in weeks; 2M/3M/6M: 2, 3 or 6 meals; 3M+2S: 3 meals and 2 snacks; 3M+3S: 3 meals and 3 snacks; 10S: Grazing diet – 100kcal every 2-3 hours; HCB: High Calorie Breakfast; HCD: High Calorie Dinner; ED: Early Dinner; LD: Late Dinner; FL: Front Loading: EED: Equal Energy Distribution; ML: Middle Loading; BL: Back Loading.

© 2024 Liu HY et al. *JAMA Network Open.*

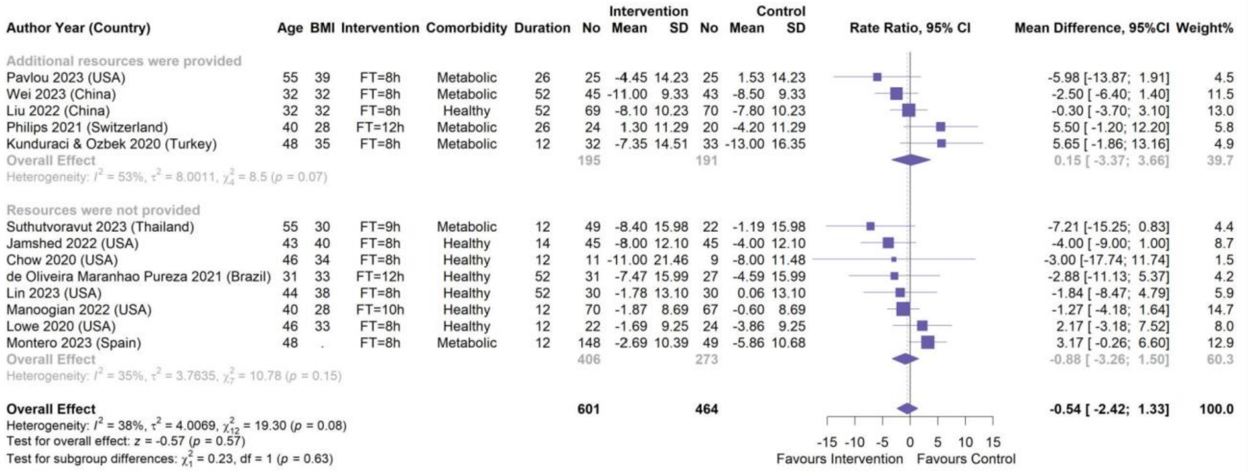

eFigure 56: Meta-analysis of difference in mean difference (95% CIs) for the effect of time-restricted eating on systolic blood pressure (mmHg), grouped by resource provision.

BMI: Body Mass Index; SD: Standard Deviation; CI: Confidence Interval; USA: United States of America; FT: Feeding Time; Duration: follow-up duration in weeks; 2M/3M/6M: 2, 3 or 6 meals; 3M+2S: 3 meals and 2 snacks; 3M+3S: 3 meals and 3 snacks; 10S: Grazing diet – 100kcal every 2-3 hours; HCB: High Calorie Breakfast; HCD: High Calorie Dinner; ED: Early Dinner; LD: Late Dinner; FL: Front Loading: EED: Equal Energy Distribution; ML: Middle Loading; BL: Back Loading.

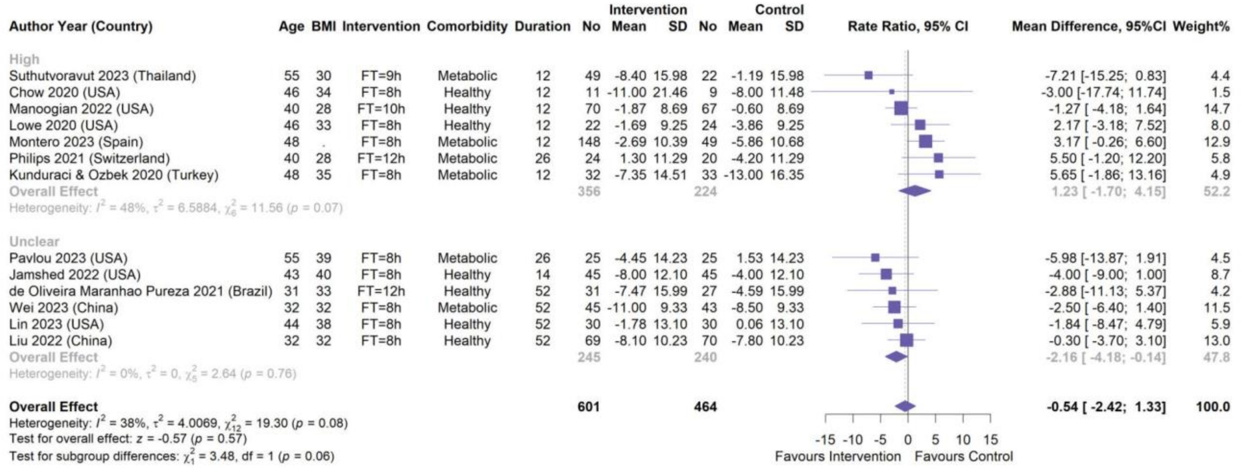

eFigure 57: Meta-analysis of difference in mean difference (95% CIs) for the effect of time-restricted eating on systolic blood pressure (mmHg), grouped by risk of bias.

BMI: Body Mass Index; SD: Standard Deviation; CI: Confidence Interval; USA: United States of America; FT: Feeding Time; Duration: follow-up duration in weeks; 2M/3M/6M: 2, 3 or 6 meals; 3M+2S: 3 meals and 2 snacks; 3M+3S: 3 meals and 3 snacks; 10S: Grazing diet – 100kcal every 2-3 hours; HCB: High Calorie Breakfast; HCD: High Calorie Dinner; ED: Early Dinner; LD: Late Dinner; FL: Front Loading: EED: Equal Energy Distribution; ML: Middle Loading; BL: Back Loading.

© 2024 Liu HY et al. *JAMA Network Open.*

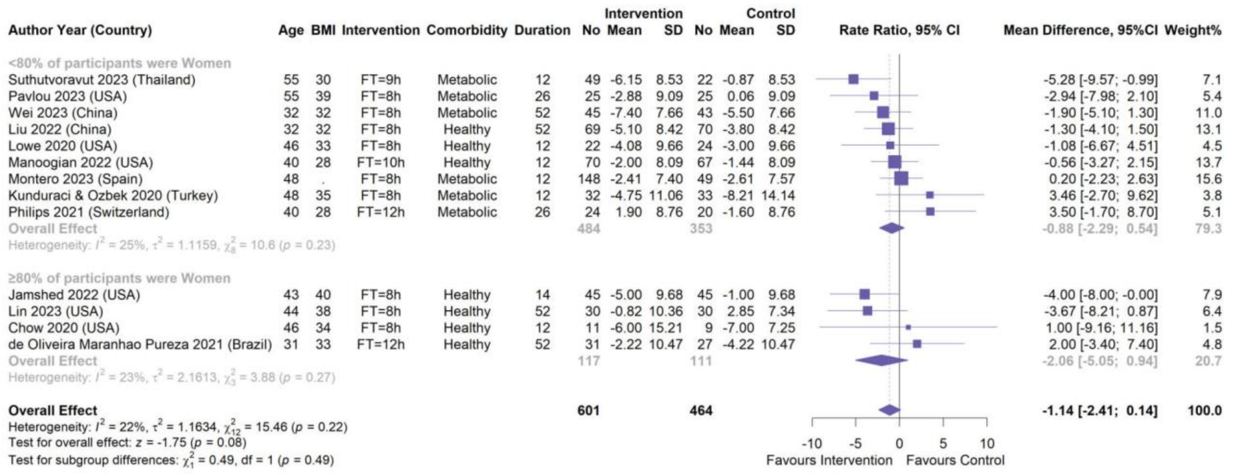

eFigure 58: Meta-analysis of difference in mean difference (95% CIs) for the effect of time-restricted eating on diastolic blood pressure (mmHg), grouped by gender proportion.

BMI: Body Mass Index; SD: Standard Deviation; CI: Confidence Interval; USA: United States of America; FT: Feeding Time; Duration: follow-up duration in weeks; 2M/3M/6M: 2, 3 or 6 meals; 3M+2S: 3 meals and 2 snacks; 3M+3S: 3 meals and 3 snacks; 10S: Grazing diet – 100kcal every 2-3 hours; HCB: High Calorie Breakfast; HCD: High Calorie Dinner; ED: Early Dinner; LD: Late Dinner; FL: Front Loading: EED: Equal Energy Distribution; ML: Middle Loading; BL: Back Loading.

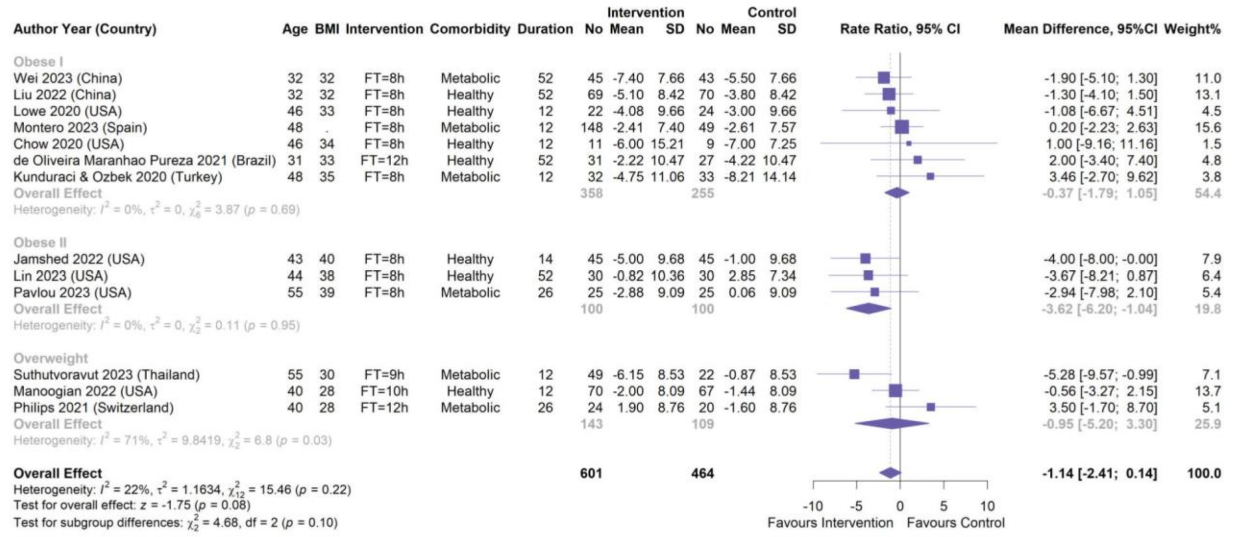

eFigure 59: Meta-analysis of difference in mean difference (95% CIs) for the effect of time-restricted eating on diastolic blood pressure (mmHg), grouped by baseline BMI status.

BMI: Body Mass Index; SD: Standard Deviation; CI: Confidence Interval; USA: United States of America; FT: Feeding Time; Duration: follow-up duration in weeks; 2M/3M/6M: 2, 3 or 6 meals; 3M+2S: 3 meals and 2 snacks; 3M+3S: 3 meals and 3 snacks; 10S: Grazing diet – 100kcal every 2-3 hours; HCB: High Calorie Breakfast; HCD: High Calorie Dinner; ED: Early Dinner; LD: Late Dinner; FL: Front Loading: EED: Equal Energy Distribution; ML: Middle Loading; BL: Back Loading.

© 2024 Liu HY et al. *JAMA Network Open.*

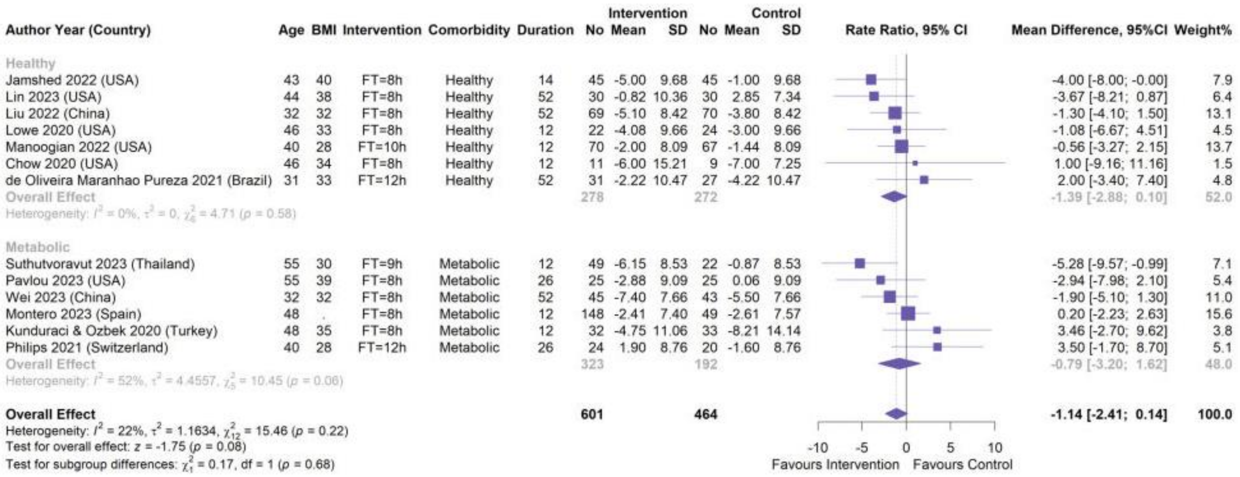

eFigure 60: Meta-analysis of difference in mean difference (95% CIs) for the effect of time-restricted eating on diastolic blood pressure (mmHg), grouped by health status

BMI: Body Mass Index; SD: Standard Deviation; CI: Confidence Interval; USA: United States of America; FT: Feeding Time; Duration: follow-up duration in weeks; 2M/3M/6M: 2, 3 or 6 meals; 3M+2S: 3 meals and 2 snacks; 3M+3S: 3 meals and 3 snacks; 10S: Grazing diet – 100kcal every 2-3 hours; HCB: High Calorie Breakfast; HCD: High Calorie Dinner; ED: Early Dinner; LD: Late Dinner; FL: Front Loading: EED: Equal Energy Distribution; ML: Middle Loading; BL: Back Loading.

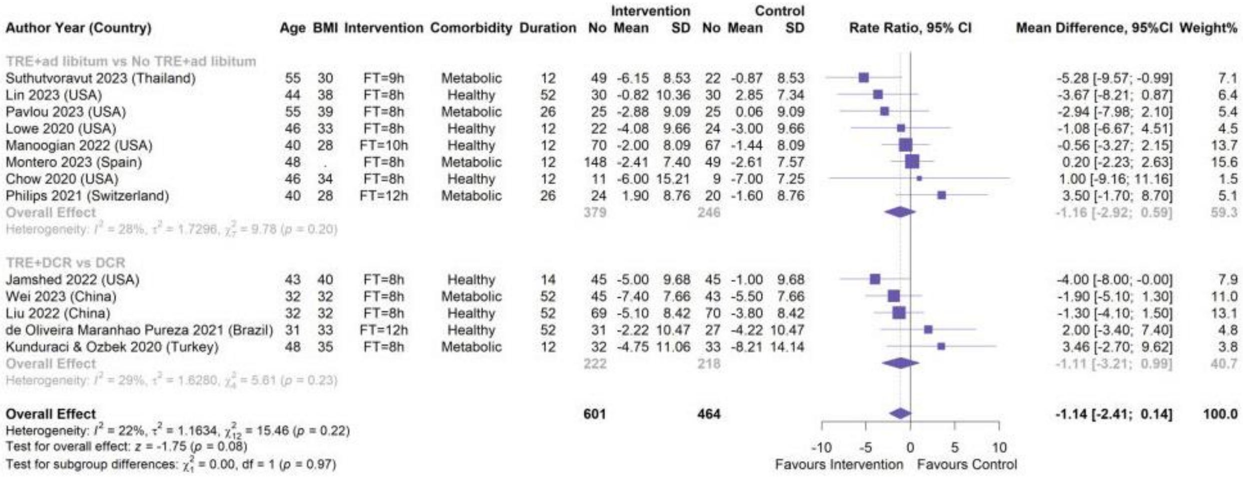

eFigure 61: Meta-analysis of difference in mean difference (95% CIs) for the effect of time-restricted eating on Diastolic blood pressure (mmHg), grouped by energy prescription

BMI: Body Mass Index; SD: Standard Deviation; CI: Confidence Interval; USA: United States of America; FT: Feeding Time; Duration: follow-up duration in weeks; 2M/3M/6M: 2, 3 or 6 meals; 3M+2S: 3 meals and 2 snacks; 3M+3S: 3 meals and 3 snacks; 10S: Grazing diet – 100kcal every 2-3 hours; HCB: High Calorie Breakfast; HCD: High Calorie Dinner; ED: Early Dinner; LD: Late Dinner; FL: Front Loading: EED: Equal Energy Distribution; ML: Middle Loading; BL: Back Loading.

© 2024 Liu HY et al. *JAMA Network Open.*

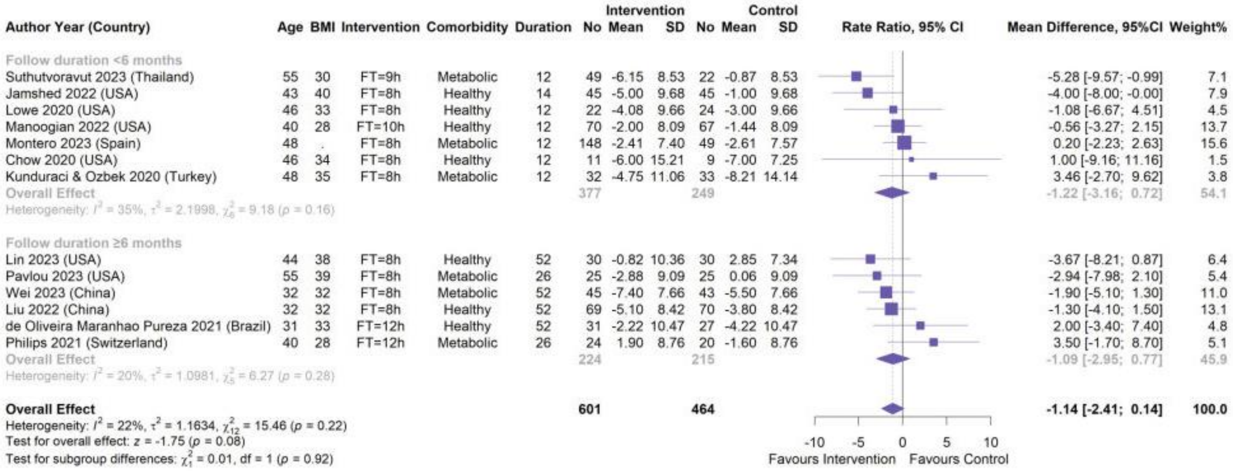

Author Year (Country)	Age	BMI	Intervention	Comorbidity	Duration	Intervention No	Intervention Mean	Intervention SD	Control No	Control Mean	Control SD	Mean Difference, 95%CI	Weight%
Follow duration <6 months													
Suthutvoravut 2023 (Thailand)	55	30	FT=9h	Metabolic	12	49	-6.15	8.53	22	-0.87	8.53	-5.28 [-9.57; -0.99]	7.1
Jamshed 2022 (USA)	43	40	FT=8h	Healthy	14	45	-5.00	9.68	45	-1.00	9.68	-4.00 [-8.00; -0.00]	7.9
Lowe 2020 (USA)	46	33	FT=8h	Healthy	12	22	-4.08	9.66	24	-3.00	9.66	-1.08 [-6.67; 4.51]	4.5
Manoogian 2022 (USA)	40	28	FT=10h	Healthy	12	70	-2.00	8.09	67	-1.44	8.09	-0.56 [-3.27; 2.15]	13.7
Montero 2023 (Spain)	48	.	FT=8h	Metabolic	12	148	-2.41	7.40	49	-2.61	7.57	0.20 [-2.23; 2.63]	15.6
Chow 2020 (USA)	46	34	FT=8h	Healthy	12	11	-6.00	15.21	9	-7.00	7.25	1.00 [-9.16; 11.16]	1.5
Kunduraci & Ozbek 2020 (Turkey)	48	35	FT=8h	Metabolic	12	32	-4.75	11.06	33	-8.21	14.14	3.46 [-2.70; 9.62]	3.8
Overall Effect						377			249			-1.22 [-3.16; 0.72]	54.1
Heterogeneity: I^2 = 35%, τ^2 = 2.1998, χ^2_6 = 9.18 (p = 0.16)													
Follow duration ≥6 months													
Lin 2023 (USA)	44	38	FT=8h	Healthy	52	30	-0.82	10.36	30	2.85	7.34	-3.67 [-8.21; 0.87]	6.4
Pavlou 2023 (USA)	55	39	FT=8h	Metabolic	26	25	-2.88	9.09	25	0.06	9.09	-2.94 [-7.98; 2.10]	5.4
Wei 2023 (China)	32	32	FT=8h	Metabolic	52	45	-7.40	7.66	43	-5.50	7.66	-1.90 [-5.10; 1.30]	11.0
Liu 2022 (China)	32	32	FT=8h	Healthy	52	69	-5.10	8.42	70	-3.80	8.42	-1.30 [-4.10; 1.50]	13.1
de Oliveira Maranhao Pureza 2021 (Brazil)	31	33	FT=12h	Healthy	52	31	-2.22	10.47	27	-4.22	10.47	2.00 [-3.40; 7.40]	4.8
Philips 2021 (Switzerland)	40	28	FT=12h	Metabolic	26	24	1.90	8.76	20	-1.60	8.76	3.50 [-1.70; 8.70]	5.1
Overall Effect						224			215			-1.09 [-2.95; 0.77]	45.9
Heterogeneity: I^2 = 20%, τ^2 = 1.0981, χ^2_5 = 6.27 (p = 0.28)													
**Overall Effect**						**601**			**464**			**-1.14 [-2.41; 0.14]**	**100.0**
Heterogeneity: I^2 = 22%, τ^2 = 1.1634, χ^2_{12} = 15.46 (p = 0.22)													
Test for overall effect: z = -1.75 (p = 0.08)													
Test for subgroup differences: χ^2_1 = 0.01, df = 1 (p = 0.92)													

eFigure 62: Meta-analysis of difference in mean difference (95% CIs) for the effect of time-restricted eating on Diastolic blood pressure (mmHg), grouped by follow duration

BMI: Body Mass Index; SD: Standard Deviation; CI: Confidence Interval; USA: United States of America; FT: Feeding Time; Duration: follow-up duration in weeks; 2M/3M/6M: 2, 3 or 6 meals; 3M+2S: 3 meals and 2 snacks; 3M+3S: 3 meals and 3 snacks; 10S: Grazing diet – 100kcal every 2-3 hours; HCB: High Calorie Breakfast; HCD: High Calorie Dinner; ED: Early Dinner; LD: Late Dinner; FL: Front Loading: EED: Equal Energy Distribution; ML: Middle Loading; BL: Back Loading.

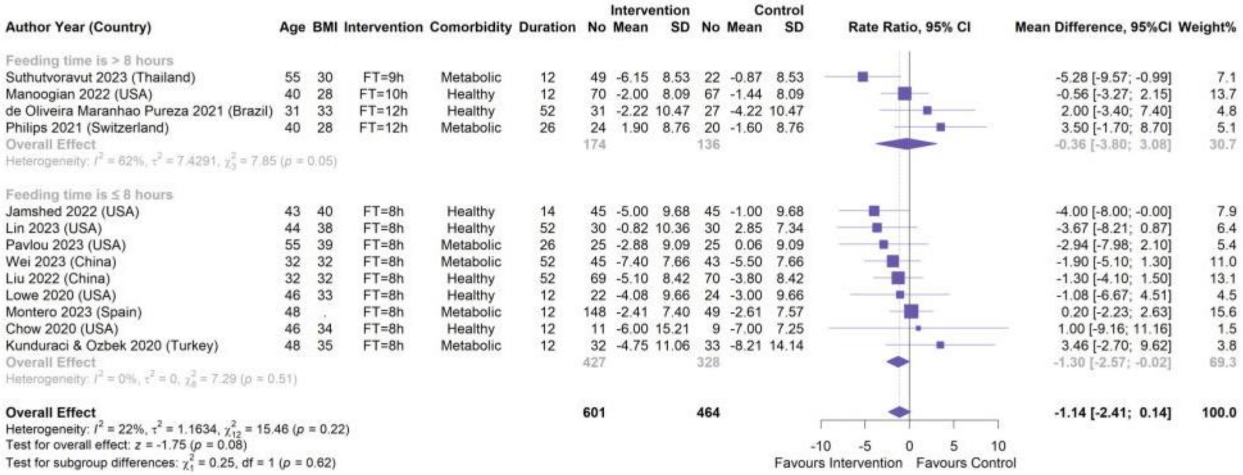

Author Year (Country)	Age	BMI	Intervention	Comorbidity	Duration	Intervention No	Intervention Mean	Intervention SD	Control No	Control Mean	Control SD	Mean Difference, 95%CI	Weight%
Feeding time is > 8 hours													
Suthutvoravut 2023 (Thailand)	55	30	FT=9h	Metabolic	12	49	-6.15	8.53	22	-0.87	8.53	-5.28 [-9.57; -0.99]	7.1
Manoogian 2022 (USA)	40	28	FT=10h	Healthy	12	70	-2.00	8.09	67	-1.44	8.09	-0.56 [-3.27; 2.15]	13.7
de Oliveira Maranhao Pureza 2021 (Brazil)	31	33	FT=12h	Healthy	52	31	-2.22	10.47	27	-4.22	10.47	2.00 [-3.40; 7.40]	4.8
Philips 2021 (Switzerland)	40	28	FT=12h	Metabolic	26	24	1.90	8.76	20	-1.60	8.76	3.50 [-1.70; 8.70]	5.1
Overall Effect						174			136			-0.36 [-3.80; 3.08]	30.7
Heterogeneity: I^2 = 62%, τ^2 = 7.4291, χ^2_3 = 7.85 (p = 0.05)													
Feeding time is ≤ 8 hours													
Jamshed 2022 (USA)	43	40	FT=8h	Healthy	14	45	-5.00	9.68	45	-1.00	9.68	-4.00 [-8.00; -0.00]	7.9
Lin 2023 (USA)	44	38	FT=8h	Healthy	52	30	-0.82	10.36	30	2.85	7.34	-3.67 [-8.21; 0.87]	6.4
Pavlou 2023 (USA)	55	39	FT=8h	Metabolic	26	25	-2.88	9.09	25	0.06	9.09	-2.94 [-7.98; 2.10]	5.4
Wei 2023 (China)	32	32	FT=8h	Metabolic	52	45	-7.40	7.66	43	-5.50	7.66	-1.90 [-5.10; 1.30]	11.0
Liu 2022 (China)	32	32	FT=8h	Healthy	52	69	-5.10	8.42	70	-3.80	8.42	-1.30 [-4.10; 1.50]	13.1
Lowe 2020 (USA)	46	33	FT=8h	Healthy	12	22	-4.08	9.66	24	-3.00	9.66	-1.08 [-6.67; 4.51]	4.5
Montero 2023 (Spain)	48	.	FT=8h	Metabolic	12	148	-2.41	7.40	49	-2.61	7.57	0.20 [-2.23; 2.63]	15.6
Chow 2020 (USA)	46	34	FT=8h	Healthy	12	11	-6.00	15.21	9	-7.00	7.25	1.00 [-9.16; 11.16]	1.5
Kunduraci & Ozbek 2020 (Turkey)	48	35	FT=8h	Metabolic	12	32	-4.75	11.06	33	-8.21	14.14	3.46 [-2.70; 9.62]	3.8
Overall Effect						427			328			-1.30 [-2.57; -0.02]	69.3
Heterogeneity: I^2 = 0%, τ^2 = 0, χ^2_8 = 7.29 (p = 0.51)													
**Overall Effect**						**601**			**464**			**-1.14 [-2.41; 0.14]**	**100.0**
Heterogeneity: I^2 = 22%, τ^2 = 1.1634, χ^2_{12} = 15.46 (p = 0.22)													
Test for overall effect: z = -1.75 (p = 0.08)													
Test for subgroup differences: χ^2_1 = 0.25, df = 1 (p = 0.62)													

eFigure 63: Meta-analysis of difference in mean difference (95% CIs) for the effect of time-restricted eating on Diastolic blood pressure (mmHg), grouped by eating window

BMI: Body Mass Index; SD: Standard Deviation; CI: Confidence Interval; USA: United States of America; FT: Feeding Time; Duration: follow-up duration in weeks; 2M/3M/6M: 2, 3 or 6 meals; 3M+2S: 3 meals and 2 snacks; 3M+3S: 3 meals and 3 snacks; 10S: Grazing diet – 100kcal every 2-3 hours; HCB: High Calorie Breakfast; HCD: High Calorie Dinner; ED: Early Dinner; LD: Late Dinner; FL: Front Loading: EED: Equal Energy Distribution; ML: Middle Loading; BL: Back Loading.

© 2024 Liu HY et al. *JAMA Network Open.*

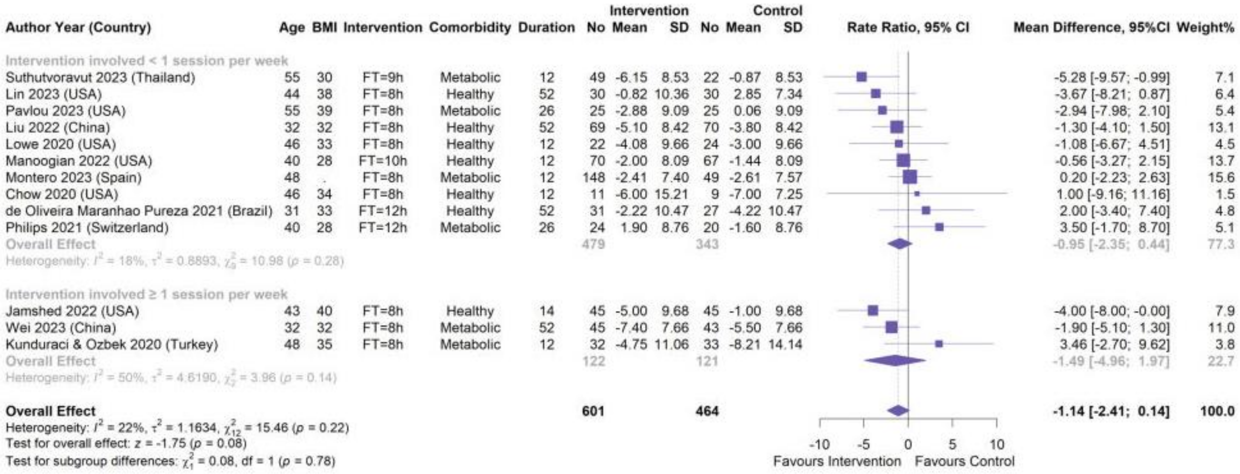

eFigure 64: Meta-analysis of difference in mean difference (95% CIs) for the effect of time-restricted eating on Diastolic blood pressure (mmHg), grouped by frequency of contact

BMI: Body Mass Index; SD: Standard Deviation; CI: Confidence Interval; USA: United States of America; FT: Feeding Time; Duration: follow-up duration in weeks; 2M/3M/6M: 2, 3 or 6 meals; 3M+2S: 3 meals and 2 snacks; 3M+3S: 3 meals and 3 snacks; 10S: Grazing diet – 100kcal every 2-3 hours; HCB: High Calorie Breakfast; HCD: High Calorie Dinner; ED: Early Dinner; LD: Late Dinner; FL: Front Loading: EED: Equal Energy Distribution; ML: Middle Loading; BL: Back Loading.

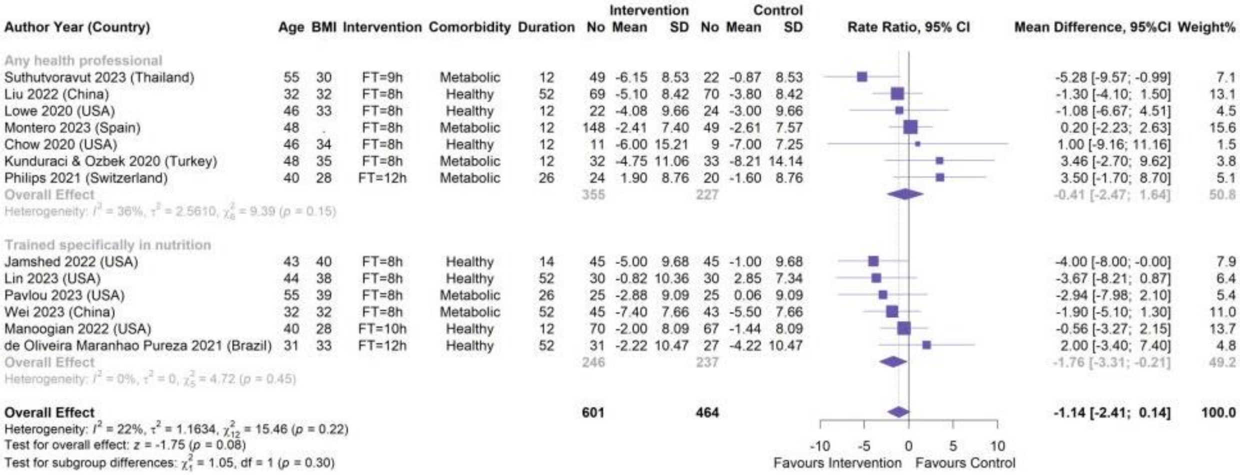

eFigure 65: Meta-analysis of difference in mean difference (95% CIs) for the effect of time-restricted eating on Diastolic blood pressure (mmHg), grouped by delivery personnel

BMI: Body Mass Index; SD: Standard Deviation; CI: Confidence Interval; USA: United States of America; FT: Feeding Time; Duration: follow-up duration in weeks; 2M/3M/6M: 2, 3 or 6 meals; 3M+2S: 3 meals and 2 snacks; 3M+3S: 3 meals and 3 snacks; 10S: Grazing diet – 100kcal every 2-3 hours; HCB: High Calorie Breakfast; HCD: High Calorie Dinner; ED: Early Dinner; LD: Late Dinner; FL: Front Loading: EED: Equal Energy Distribution; ML: Middle Loading; BL: Back Loading.

© 2024 Liu HY et al. *JAMA Network Open.*

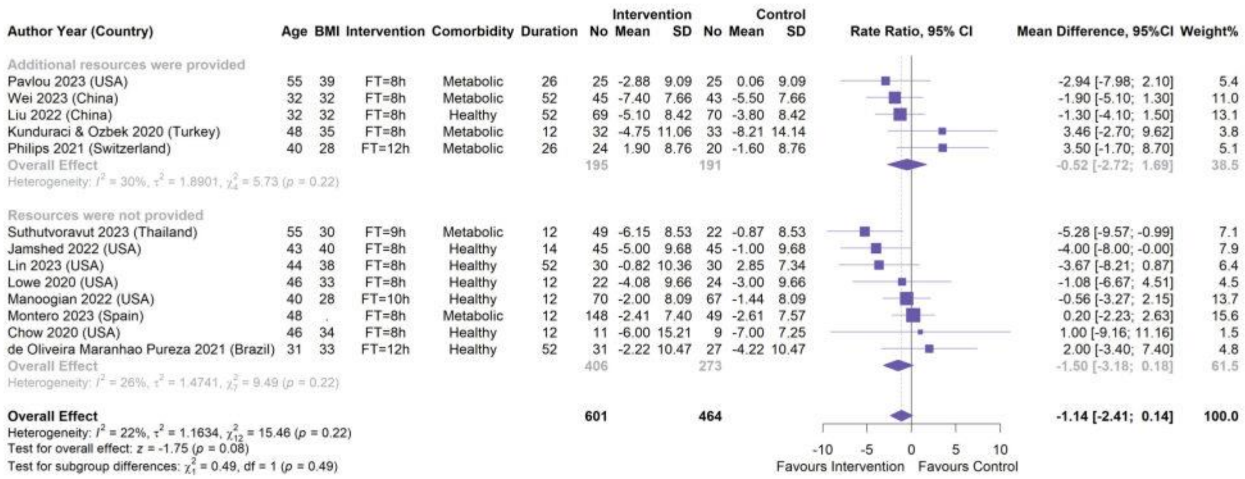

eFigure 66: Meta-analysis of difference in mean difference (95% CIs) for the effect of time-restricted eating on Diastolic blood pressure (mmHg), grouped by resource provision

BMI: Body Mass Index; SD: Standard Deviation; CI: Confidence Interval; USA: United States of America; FT: Feeding Time; Duration: follow-up duration in weeks; 2M/3M/6M: 2, 3 or 6 meals; 3M+2S: 3 meals and 2 snacks; 3M+3S: 3 meals and 3 snacks; 10S: Grazing diet – 100kcal every 2-3 hours; HCB: High Calorie Breakfast; HCD: High Calorie Dinner; ED: Early Dinner; LD: Late Dinner; FL: Front Loading: EED: Equal Energy Distribution; ML: Middle Loading; BL: Back Loading.

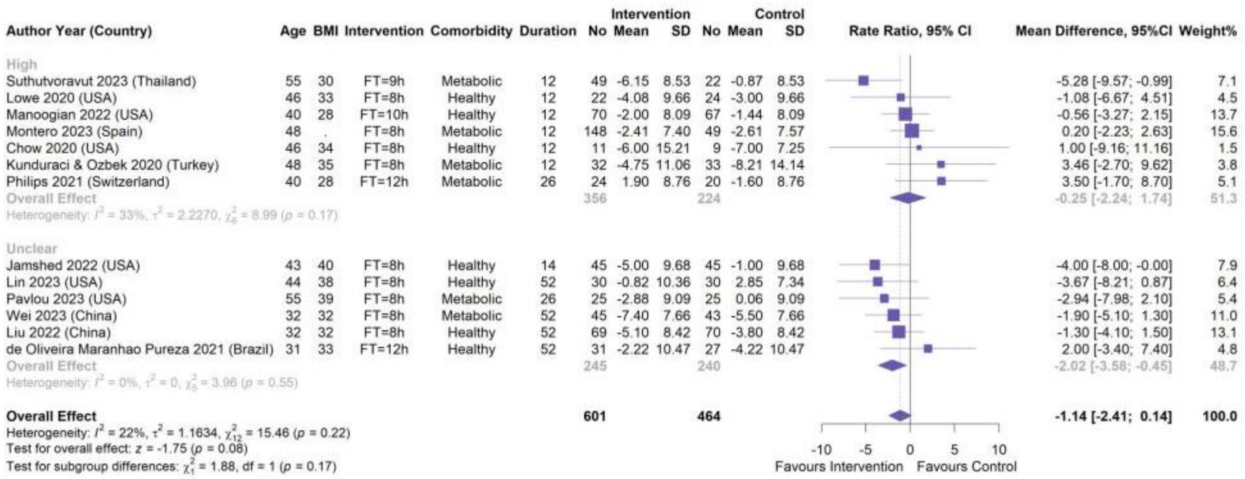

eFigure 67: Meta-analysis of difference in mean difference (95% CIs) for the effect of time-restricted eating on Diastolic blood pressure (mmHg), grouped by risk of bias

BMI: Body Mass Index; SD: Standard Deviation; CI: Confidence Interval; USA: United States of America; FT: Feeding Time; Duration: follow-up duration in weeks; 2M/3M/6M: 2, 3 or 6 meals; 3M+2S: 3 meals and 2 snacks; 3M+3S: 3 meals and 3 snacks; 10S: Grazing diet – 100kcal every 2-3 hours; HCB: High Calorie Breakfast; HCD: High Calorie Dinner; ED: Early Dinner; LD: Late Dinner; FL: Front Loading: EED: Equal Energy Distribution; ML: Middle Loading; BL: Back Loading.

© 2024 Liu HY et al. *JAMA Network Open.*

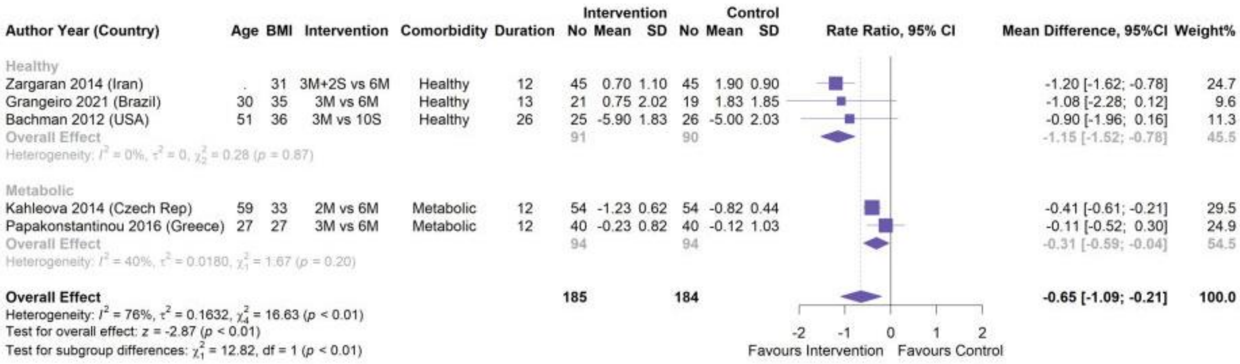

eFigure 68: Meta-analysis of difference in mean difference (95% CIs) for the effect of meal frequency on BMI (kg/m²), grouped by health status

BMI: Body Mass Index; SD: Standard Deviation; CI: Confidence Interval; USA: United States of America; FT: Feeding Time; Duration: follow-up duration in weeks; 2M/3M/6M: 2, 3 or 6 meals; 3M+2S: 3 meals and 2 snacks; 3M+3S: 3 meals and 3 snacks; 10S: Grazing diet – 100kcal every 2-3 hours; HCB: High Calorie Breakfast; HCD: High Calorie Dinner; ED: Early Dinner; LD: Late Dinner; FL: Front Loading: EED: Equal Energy Distribution; ML: Middle Loading; BL: Back Loading.

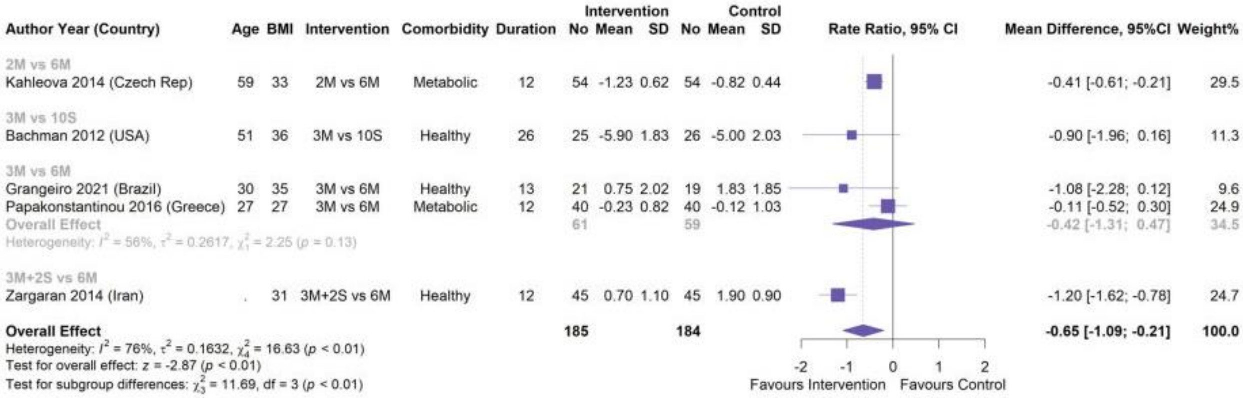

eFigure 69: Meta-analysis of difference in mean difference (95% CIs) for the effect of meal frequency on BMI (kg/m²), grouped by intervention intensity

BMI: Body Mass Index; SD: Standard Deviation; CI: Confidence Interval; USA: United States of America; FT: Feeding Time; Duration: follow-up duration in weeks; 2M/3M/6M: 2, 3 or 6 meals; 3M+2S: 3 meals and 2 snacks; 3M+3S: 3 meals and 3 snacks; 10S: Grazing diet – 100kcal every 2-3 hours; HCB: High Calorie Breakfast; HCD: High Calorie Dinner; ED: Early Dinner; LD: Late Dinner; FL: Front Loading: EED: Equal Energy Distribution; ML: Middle Loading; BL: Back Loading.

© 2024 Liu HY et al. *JAMA Network Open.*

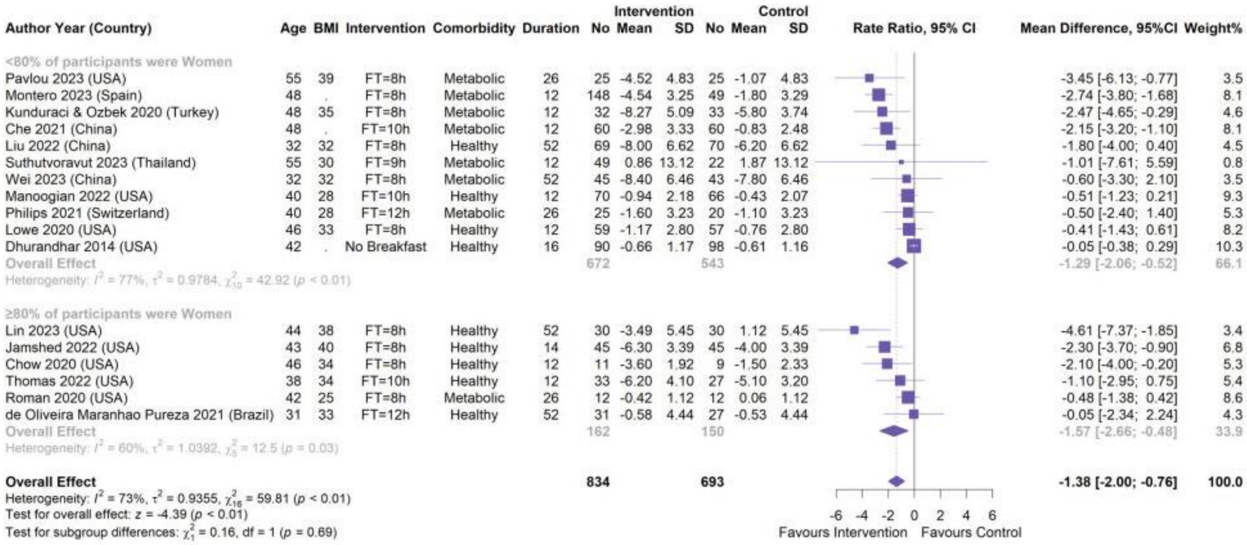

Author Year (Country)	Age	BMI	Intervention	Comorbidity	Duration	Intervention No	Intervention Mean	Intervention SD	Control No	Control Mean	Control SD	Mean Difference, 95%CI	Weight%
<80% of participants were Women													
Pavlou 2023 (USA)	55	39	FT=8h	Metabolic	26	25	-4.52	4.83	25	-1.07	4.83	-3.45 [-6.13; -0.77]	3.5
Montero 2023 (Spain)	48	.	FT=8h	Metabolic	12	148	-4.54	3.25	49	-1.80	3.29	-2.74 [-3.80; -1.68]	8.1
Kunduraci & Ozbek 2020 (Turkey)	48	35	FT=8h	Metabolic	12	32	-8.27	5.09	33	-5.80	3.74	-2.47 [-4.65; -0.29]	4.6
Che 2021 (China)	48	.	FT=10h	Metabolic	12	60	-2.98	3.33	60	-0.83	2.48	-2.15 [-3.20; -1.10]	8.1
Liu 2022 (China)	32	32	FT=8h	Healthy	52	69	-8.00	6.62	70	-6.20	6.62	-1.80 [-4.00; 0.40]	4.5
Suthutvoravut 2023 (Thailand)	55	30	FT=9h	Metabolic	12	49	0.86	13.12	22	1.87	13.12	-1.01 [-7.61; 5.59]	0.8
Wei 2023 (China)	32	32	FT=8h	Metabolic	52	45	-8.40	6.46	43	-7.80	6.46	-0.60 [-3.30; 2.10]	3.5
Manoogian 2022 (USA)	40	28	FT=10h	Healthy	12	70	-0.94	2.18	66	-0.43	2.07	-0.51 [-1.23; 0.21]	9.3
Philips 2021 (Switzerland)	40	28	FT=12h	Metabolic	26	25	-1.60	3.23	20	-1.10	3.23	-0.50 [-2.40; 1.40]	5.3
Lowe 2020 (USA)	46	33	FT=8h	Healthy	12	59	-1.17	2.80	57	-0.76	2.80	-0.41 [-1.43; 0.61]	8.2
Dhurandhar 2014 (USA)	42	.	No Breakfast	Healthy	16	90	-0.66	1.17	98	-0.61	1.16	-0.05 [-0.38; 0.29]	10.3
Overall Effect						672			543			-1.29 [-2.06; -0.52]	66.1
Heterogeneity: $I^2 = 77\%$, $\tau^2 = 0.9784$, $\chi^2_{10} = 42.92$ ($p < 0.01$)													
≥80% of participants were Women													
Lin 2023 (USA)	44	38	FT=8h	Healthy	52	30	-3.49	5.45	30	1.12	5.45	-4.61 [-7.37; -1.85]	3.4
Jamshed 2022 (USA)	43	40	FT=8h	Healthy	14	45	-6.30	3.39	45	-4.00	3.39	-2.30 [-3.70; -0.90]	6.8
Chow 2020 (USA)	46	34	FT=8h	Healthy	12	11	-3.60	1.92	9	-1.50	2.33	-2.10 [-4.00; -0.20]	5.3
Thomas 2022 (USA)	38	34	FT=10h	Healthy	12	33	-6.20	4.10	27	-5.10	3.20	-1.10 [-2.95; 0.75]	5.4
Roman 2020 (USA)	42	25	FT=8h	Metabolic	26	12	-0.42	1.12	12	0.06	1.12	-0.48 [-1.38; 0.42]	8.6
de Oliveira Maranhao Pureza 2021 (Brazil)	31	33	FT=12h	Healthy	52	31	-0.58	4.44	27	-0.53	4.44	-0.05 [-2.34; 2.24]	4.3
Overall Effect						162			150			-1.57 [-2.66; -0.48]	33.9
Heterogeneity: $I^2 = 60\%$, $\tau^2 = 1.0392$, $\chi^2_5 = 12.5$ ($p = 0.03$)													
**Overall Effect**						**834**			**693**			**-1.38 [-2.00; -0.76]**	**100.0**

Heterogeneity: $I^2 = 73\%$, $\tau^2 = 0.9355$, $\chi^2_{16} = 59.81$ ($p < 0.01$)
Test for overall effect: $z = -4.39$ ($p < 0.01$)
Test for subgroup differences: $\chi^2_1 = 0.16$, df = 1 ($p = 0.69$)

eFigure 70: Meta-analysis of difference in mean difference (95% CIs) for the effect of time restricted eating on weight (kg), grouped by gender proportion

BMI: Body Mass Index; SD: Standard Deviation; CI: Confidence Interval; USA: United States of America; FT: Feeding Time; Duration: follow-up duration in weeks; 2M/3M/6M: 2, 3 or 6 meals; 3M+2S: 3 meals and 2 snacks; 3M+3S: 3 meals and 3 snacks; 10S: Grazing diet – 100kcal every 2-3 hours; HCB: High Calorie Breakfast; HCD: High Calorie Dinner; ED: Early Dinner; LD: Late Dinner; FL: Front Loading: EED: Equal Energy Distribution; ML: Middle Loading; BL: Back Loading.

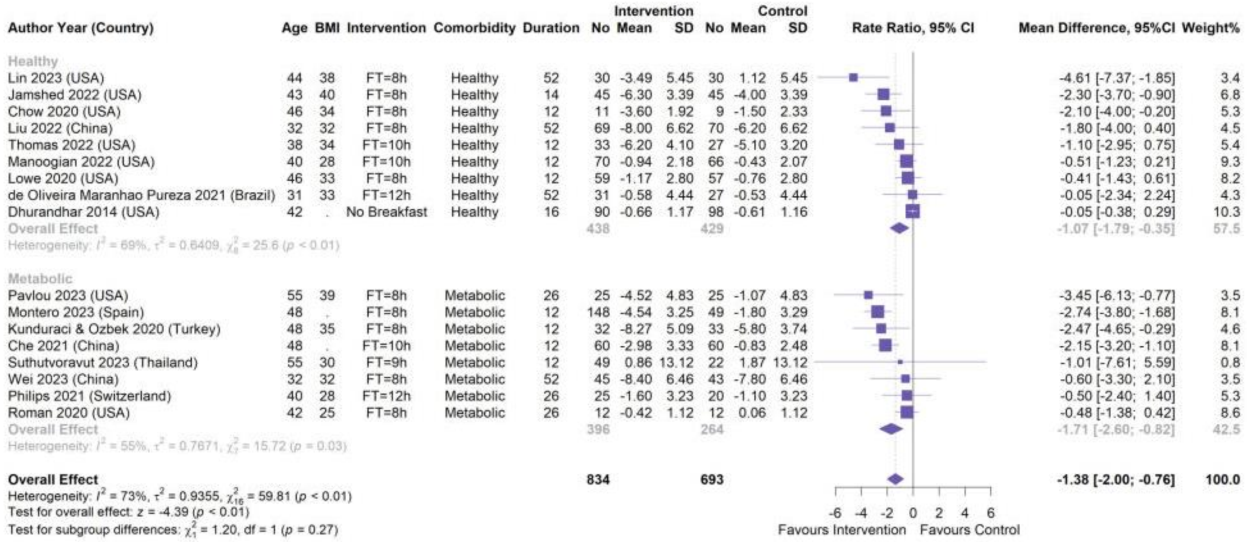

Author Year (Country)	Age	BMI	Intervention	Comorbidity	Duration	Intervention No	Intervention Mean	Intervention SD	Control No	Control Mean	Control SD	Mean Difference, 95%CI	Weight%
Healthy													
Lin 2023 (USA)	44	38	FT=8h	Healthy	52	30	-3.49	5.45	30	1.12	5.45	-4.61 [-7.37; -1.85]	3.4
Jamshed 2022 (USA)	43	40	FT=8h	Healthy	14	45	-6.30	3.39	45	-4.00	3.39	-2.30 [-3.70; -0.90]	6.8
Chow 2020 (USA)	46	34	FT=8h	Healthy	12	11	-3.60	1.92	9	-1.50	2.33	-2.10 [-4.00; -0.20]	5.3
Liu 2022 (China)	32	32	FT=8h	Healthy	52	69	-8.00	6.62	70	-6.20	6.62	-1.80 [-4.00; 0.40]	4.5
Thomas 2022 (USA)	38	34	FT=10h	Healthy	12	33	-6.20	4.10	27	-5.10	3.20	-1.10 [-2.95; 0.75]	5.4
Manoogian 2022 (USA)	40	28	FT=10h	Healthy	12	70	-0.94	2.18	66	-0.43	2.07	-0.51 [-1.23; 0.21]	9.3
Lowe 2020 (USA)	46	33	FT=8h	Healthy	12	59	-1.17	2.80	57	-0.76	2.80	-0.41 [-1.43; 0.61]	8.2
de Oliveira Maranhao Pureza 2021 (Brazil)	31	33	FT=12h	Healthy	52	31	-0.58	4.44	27	-0.53	4.44	-0.05 [-2.34; 2.24]	4.3
Dhurandhar 2014 (USA)	42	.	No Breakfast	Healthy	16	90	-0.66	1.17	98	-0.61	1.16	-0.05 [-0.38; 0.29]	10.3
Overall Effect						438			429			-1.07 [-1.79; -0.35]	57.5
Heterogeneity: $I^2 = 69\%$, $\tau^2 = 0.6409$, $\chi^2_8 = 25.6$ ($p < 0.01$)													
Metabolic													
Pavlou 2023 (USA)	55	39	FT=8h	Metabolic	26	25	-4.52	4.83	25	-1.07	4.83	-3.45 [-6.13; -0.77]	3.5
Montero 2023 (Spain)	48	.	FT=8h	Metabolic	12	148	-4.54	3.25	49	-1.80	3.29	-2.74 [-3.80; -1.68]	8.1
Kunduraci & Ozbek 2020 (Turkey)	48	35	FT=8h	Metabolic	12	32	-8.27	5.09	33	-5.80	3.74	-2.47 [-4.65; -0.29]	4.6
Che 2021 (China)	48	.	FT=10h	Metabolic	12	60	-2.98	3.33	60	-0.83	2.48	-2.15 [-3.20; -1.10]	8.1
Suthutvoravut 2023 (Thailand)	55	30	FT=9h	Metabolic	12	49	0.86	13.12	22	1.87	13.12	-1.01 [-7.61; 5.59]	0.8
Wei 2023 (China)	32	32	FT=8h	Metabolic	52	45	-8.40	6.46	43	-7.80	6.46	-0.60 [-3.30; 2.10]	3.5
Philips 2021 (Switzerland)	40	28	FT=12h	Metabolic	26	25	-1.60	3.23	20	-1.10	3.23	-0.50 [-2.40; 1.40]	5.3
Roman 2020 (USA)	42	25	FT=8h	Metabolic	26	12	-0.42	1.12	12	0.06	1.12	-0.48 [-1.38; 0.42]	8.6
Overall Effect						396			264			-1.71 [-2.60; -0.82]	42.5
Heterogeneity: $I^2 = 55\%$, $\tau^2 = 0.7671$, $\chi^2_7 = 15.72$ ($p = 0.03$)													
**Overall Effect**						**834**			**693**			**-1.38 [-2.00; -0.76]**	**100.0**

Heterogeneity: $I^2 = 73\%$, $\tau^2 = 0.9355$, $\chi^2_{16} = 59.81$ ($p < 0.01$)
Test for overall effect: $z = -4.39$ ($p < 0.01$)
Test for subgroup differences: $\chi^2_1 = 1.20$, df = 1 ($p = 0.27$)

eFigure 71: Meta-analysis of difference in mean difference (95% CIs) for the effect of time restricted eating on weight (kg), grouped by health status

BMI: Body Mass Index; SD: Standard Deviation; CI: Confidence Interval; USA: United States of America; FT: Feeding Time; Duration: follow-up duration in weeks; 2M/3M/6M: 2, 3 or 6 meals; 3M+2S: 3 meals and 2 snacks; 3M+3S: 3 meals and 3 snacks; 10S: Grazing diet – 100kcal every 2-3 hours; HCB: High Calorie Breakfast; HCD: High Calorie Dinner; ED: Early Dinner; LD: Late Dinner; FL: Front Loading: EED: Equal Energy Distribution; ML: Middle Loading; BL: Back Loading.

© 2024 Liu HY et al. *JAMA Network Open.*

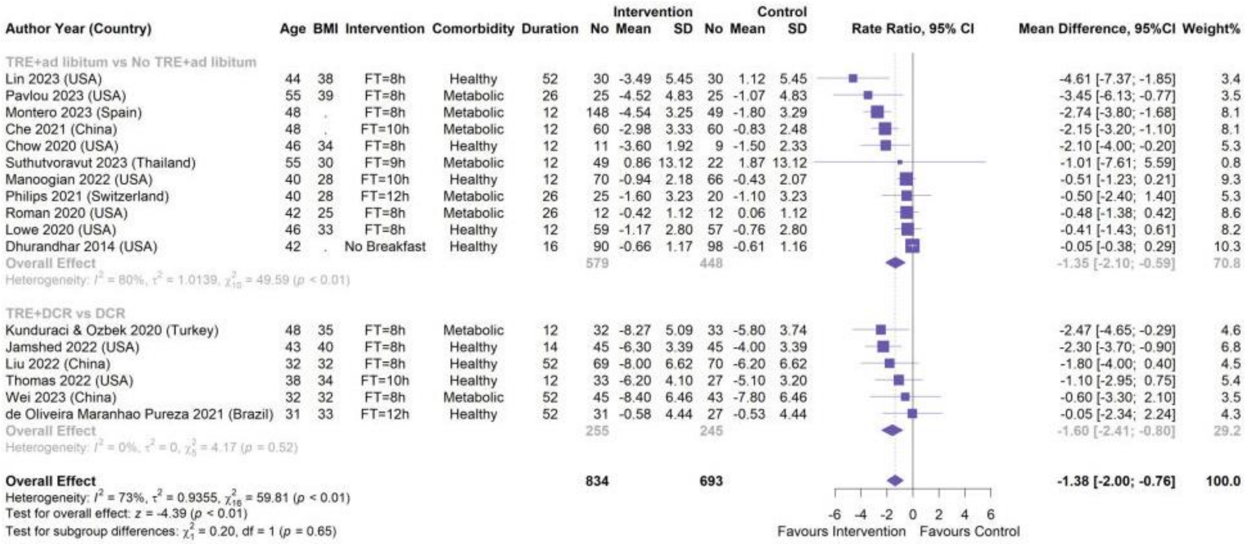

eFigure 72: Meta-analysis of difference in mean difference (95% CIs) for the effect of time restricted eating on weight (kg), grouped by energy prescription

BMI: Body Mass Index; SD: Standard Deviation; CI: Confidence Interval; USA: United States of America; FT: Feeding Time; Duration: follow-up duration in weeks; 2M/3M/6M: 2, 3 or 6 meals; 3M+2S: 3 meals and 2 snacks; 3M+3S: 3 meals and 3 snacks; 10S: Grazing diet – 100kcal every 2-3 hours; HCB: High Calorie Breakfast; HCD: High Calorie Dinner; ED: Early Dinner; LD: Late Dinner; FL: Front Loading: EED: Equal Energy Distribution; ML: Middle Loading; BL: Back Loading.

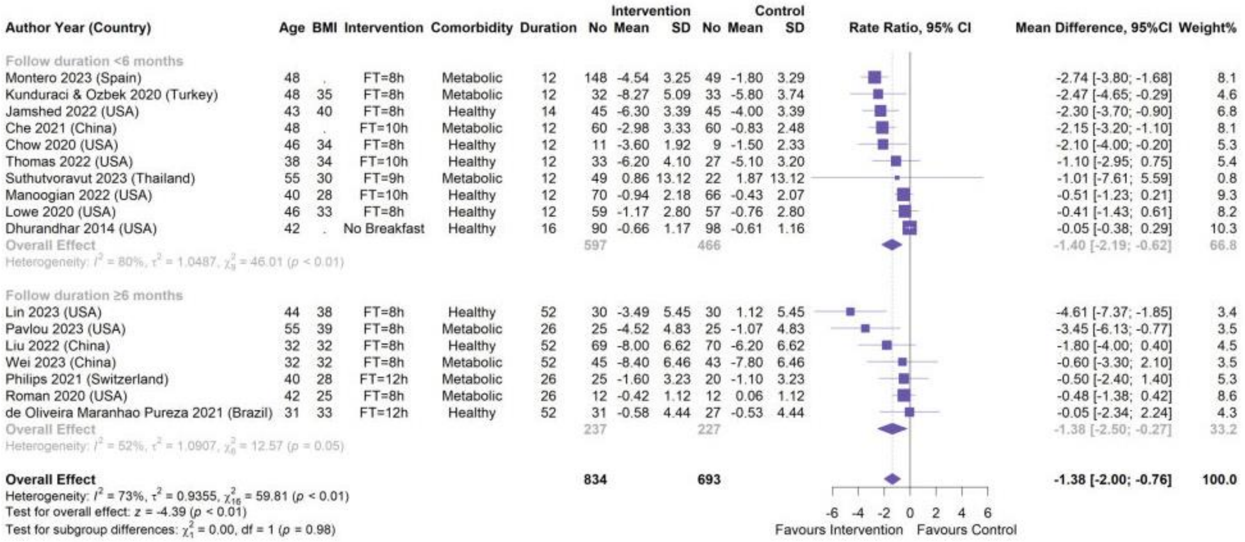

eFigure 73: Meta-analysis of difference in mean difference (95% CIs) for the effect of time restricted eating on weight (kg), grouped by follow duration

BMI: Body Mass Index; SD: Standard Deviation; CI: Confidence Interval; USA: United States of America; FT: Feeding Time; Duration: follow-up duration in weeks; 2M/3M/6M: 2, 3 or 6 meals; 3M+2S: 3 meals and 2 snacks; 3M+3S: 3 meals and 3 snacks; 10S: Grazing diet – 100kcal every 2-3 hours; HCB: High Calorie Breakfast; HCD: High Calorie Dinner; ED: Early Dinner; LD: Late Dinner; FL: Front Loading: EED: Equal Energy Distribution; ML: Middle Loading; BL: Back Loading.

© 2024 Liu HY et al. *JAMA Network Open.*

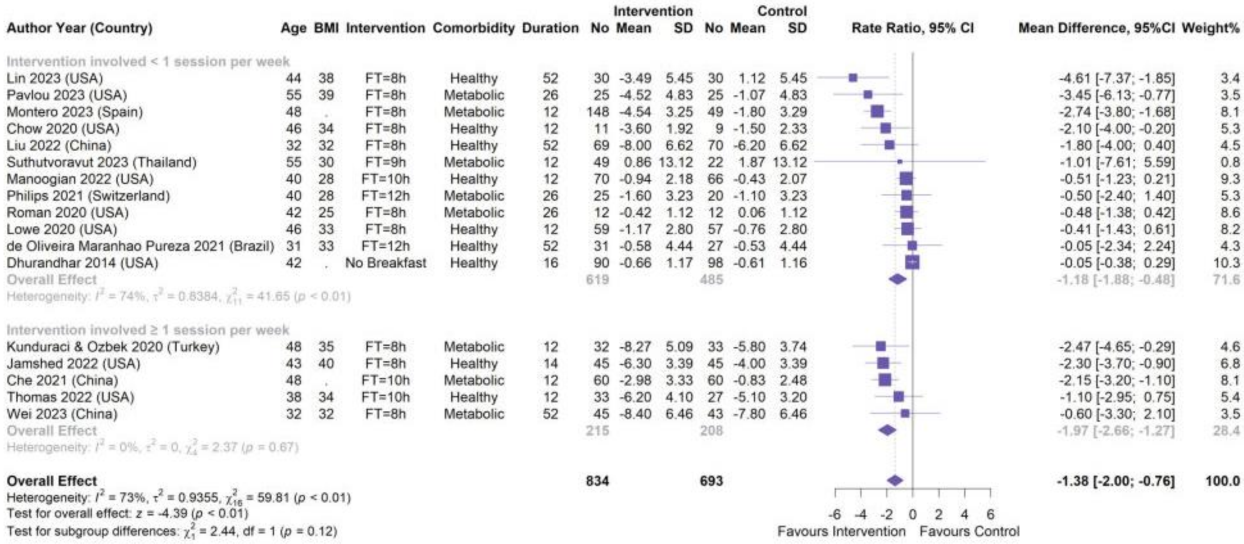

eFigure 74: Meta-analysis of difference in mean difference (95% CIs) for the effect of time restricted eating on weight (kg), grouped by frequency of contact

BMI: Body Mass Index; SD: Standard Deviation; CI: Confidence Interval; USA: United States of America; FT: Feeding Time; Duration: follow-up duration in weeks; 2M/3M/6M: 2, 3 or 6 meals; 3M+2S: 3 meals and 2 snacks; 3M+3S: 3 meals and 3 snacks; 10S: Grazing diet – 100kcal every 2-3 hours; HCB: High Calorie Breakfast; HCD: High Calorie Dinner; ED: Early Dinner; LD: Late Dinner; FL: Front Loading: EED: Equal Energy Distribution; ML: Middle Loading; BL: Back Loading.

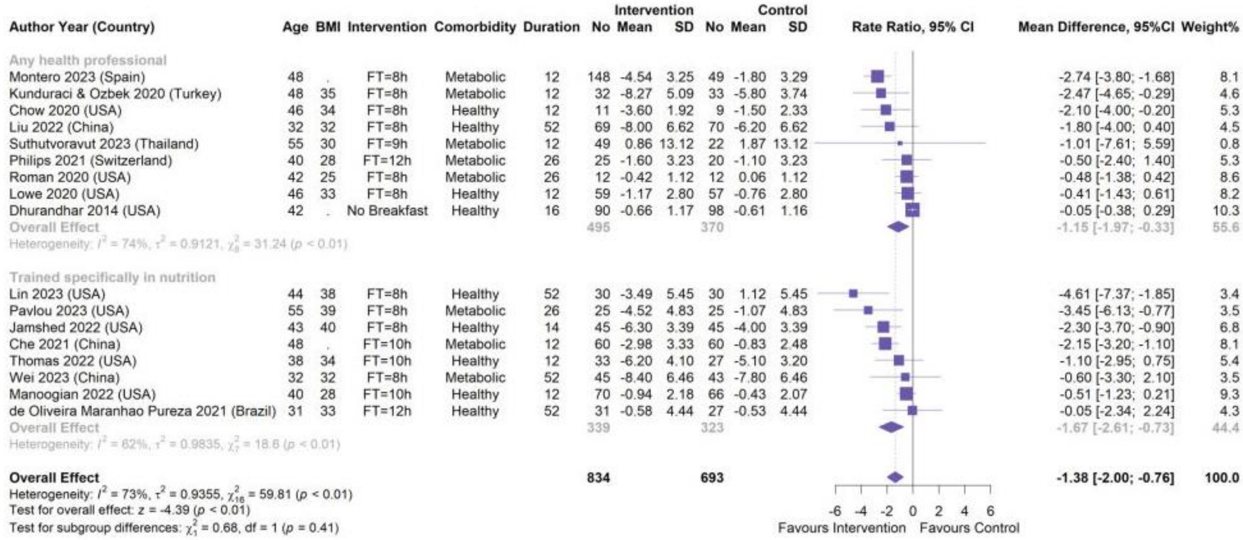

eFigure 75: Meta-analysis of difference in mean difference (95% CIs) for the effect of time restricted eating on weight (kg), grouped by delivery personnel

BMI: Body Mass Index; SD: Standard Deviation; CI: Confidence Interval; USA: United States of America; FT: Feeding Time; Duration: follow-up duration in weeks; 2M/3M/6M: 2, 3 or 6 meals; 3M+2S: 3 meals and 2 snacks; 3M+3S: 3 meals and 3 snacks; 10S: Grazing diet – 100kcal every 2-3 hours; HCB: High Calorie Breakfast; HCD: High Calorie Dinner; ED: Early Dinner; LD: Late Dinner; FL: Front Loading: EED: Equal Energy Distribution; ML: Middle Loading; BL: Back Loading.

© 2024 Liu HY et al. *JAMA Network Open.*

Author Year (Country)	Age	BMI	Intervention	Comorbidity	Duration	Intervention No	Intervention Mean	Intervention SD	Control No	Control Mean	Control SD	Mean Difference, 95%CI	Weight%
Additional resources were provided													
Pavlou 2023 (USA)	55	39	FT=8h	Metabolic	26	25	-4.52	4.83	25	-1.07	4.83	-3.45 [-6.13; -0.77]	3.5
Kunduraci & Ozbek 2020 (Turkey)	48	35	FT=8h	Metabolic	12	32	-8.27	5.09	33	-5.80	3.74	-2.47 [-4.65; -0.29]	4.6
Liu 2022 (China)	32	32	FT=8h	Healthy	52	69	-8.00	6.62	70	-6.20	6.62	-1.80 [-4.00; 0.40]	4.5
Wei 2023 (China)	32	32	FT=8h	Metabolic	52	45	-8.40	6.46	43	-7.80	6.46	-0.60 [-3.30; 2.10]	3.5
Philips 2021 (Switzerland)	40	28	FT=12h	Metabolic	26	25	-1.60	3.23	20	-1.10	3.23	-0.50 [-2.40; 1.40]	5.3
Dhurandhar 2014 (USA)	42	.	No Breakfast	Healthy	16	90	-0.66	1.17	98	-0.61	1.16	-0.05 [-0.38; 0.29]	10.3
Overall Effect						286			289			-1.20 [-2.36; -0.04]	31.7
Heterogeneity: I^2 = 61%, τ^2 = 1.1177, χ^2_5 = 12.85 (p = 0.02)													
Resources were not provided													
Lin 2023 (USA)	44	38	FT=8h	Healthy	52	30	-3.49	5.45	30	1.12	5.45	-4.61 [-7.37; -1.85]	3.4
Montero 2023 (Spain)	48	.	FT=8h	Metabolic	12	148	-4.54	3.25	49	-1.80	3.29	-2.74 [-3.80; -1.68]	8.1
Jamshed 2022 (USA)	43	40	FT=8h	Healthy	14	45	-6.30	3.39	45	-4.00	3.39	-2.30 [-3.70; -0.90]	6.8
Che 2021 (China)	48	.	FT=10h	Metabolic	12	60	-2.98	3.33	60	-0.83	2.48	-2.15 [-3.20; -1.10]	8.1
Chow 2020 (USA)	46	34	FT=8h	Healthy	12	11	-3.60	1.92	9	-1.50	2.33	-2.10 [-4.00; -0.20]	5.3
Thomas 2022 (USA)	38	34	FT=10h	Healthy	12	33	-6.20	4.10	27	-5.10	3.20	-1.10 [-2.95; 0.75]	5.4
Suthutvoravut 2023 (Thailand)	55	30	FT=9h	Metabolic	12	49	0.86	13.12	22	1.87	13.12	-1.01 [-7.61; 5.59]	0.8
Manoogian 2022 (USA)	40	28	FT=10h	Healthy	12	70	-0.94	2.18	66	-0.43	2.07	-0.51 [-1.23; 0.21]	9.3
Roman 2020 (USA)	42	25	FT=8h	Metabolic	26	12	-0.42	1.12	12	0.06	1.12	-0.48 [-1.38; 0.42]	8.6
Lowe 2020 (USA)	46	33	FT=8h	Healthy	12	59	-1.17	2.80	57	-0.76	2.80	-0.41 [-1.43; 0.61]	8.2
de Oliveira Maranhao Pureza 2021 (Brazil)	31	33	FT=12h	Healthy	52	31	-0.58	4.44	27	-0.53	4.44	-0.05 [-2.34; 2.24]	4.3
Overall Effect						548			404			-1.47 [-2.19; -0.76]	68.3
Heterogeneity: I^2 = 66%, τ^2 = 0.8192, χ^2_{10} = 29.64 (p < 0.01)													
**Overall Effect**						**834**			**693**			**-1.38 [-2.00; -0.76]**	**100.0**

Heterogeneity: I^2 = 73%, τ^2 = 0.9355, χ^2_{16} = 59.81 (p < 0.01)
Test for overall effect: z = -4.39 (p < 0.01)
Test for subgroup differences: χ^2_1 = 0.15, df = 1 (p = 0.70)

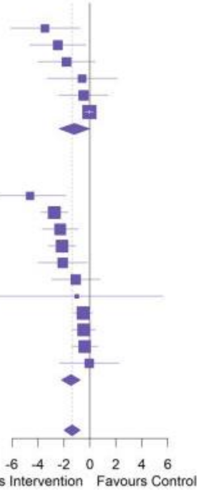

eFigure 76: Meta-analysis of difference in mean difference (95% CIs) for the effect of time restricted eating on weight (kg), grouped by resource provision

BMI: Body Mass Index; SD: Standard Deviation; CI: Confidence Interval; USA: United States of America; FT: Feeding Time; Duration: follow-up duration in weeks; 2M/3M/6M: 2, 3 or 6 meals; 3M+2S: 3 meals and 2 snacks; 3M+3S: 3 meals and 3 snacks; 10S: Grazing diet – 100kcal every 2-3 hours; HCB: High Calorie Breakfast; HCD: High Calorie Dinner; ED: Early Dinner; LD: Late Dinner; FL: Front Loading: EED: Equal Energy Distribution; ML: Middle Loading; BL: Back Loading.

Author Year (Country)	Age	BMI	Intervention	Comorbidity	Duration	Intervention No	Intervention Mean	Intervention SD	Control No	Control Mean	Control SD	Mean Difference, 95%CI	Weight%
High													
Montero 2023 (Spain)	48	.	FT=8h	Metabolic	12	148	-4.54	3.25	49	-1.80	3.29	-2.74 [-3.80; -1.68]	8.1
Kunduraci & Ozbek 2020 (Turkey)	48	35	FT=8h	Metabolic	12	32	-8.27	5.09	33	-5.80	3.74	-2.47 [-4.65; -0.29]	4.6
Che 2021 (China)	48	.	FT=10h	Metabolic	12	60	-2.98	3.33	60	-0.83	2.48	-2.15 [-3.20; -1.10]	8.1
Chow 2020 (USA)	46	34	FT=8h	Healthy	12	11	-3.60	1.92	9	-1.50	2.33	-2.10 [-4.00; -0.20]	5.3
Thomas 2022 (USA)	38	34	FT=10h	Healthy	12	33	-6.20	4.10	27	-5.10	3.20	-1.10 [-2.95; 0.75]	5.4
Suthutvoravut 2023 (Thailand)	55	30	FT=9h	Metabolic	12	49	0.86	13.12	22	1.87	13.12	-1.01 [-7.61; 5.59]	0.8
Manoogian 2022 (USA)	40	28	FT=10h	Healthy	12	70	-0.94	2.18	66	-0.43	2.07	-0.51 [-1.23; 0.21]	9.3
Philips 2021 (Switzerland)	40	28	FT=12h	Metabolic	26	25	-1.60	3.23	20	-1.10	3.23	-0.50 [-2.40; 1.40]	5.3
Roman 2020 (USA)	42	25	FT=8h	Metabolic	26	12	-0.42	1.12	12	0.06	1.12	-0.48 [-1.38; 0.42]	8.6
Lowe 2020 (USA)	46	33	FT=8h	Healthy	12	59	-1.17	2.80	57	-0.76	2.80	-0.41 [-1.43; 0.61]	8.2
Dhurandhar 2014 (USA)	42	.	No Breakfast	Healthy	16	90	-0.66	1.17	98	-0.61	1.16	-0.05 [-0.38; 0.29]	10.3
Overall Effect						589			453			-1.12 [-1.78; -0.46]	73.9
Heterogeneity: I^2 = 75%, τ^2 = 0.7472, χ^2_{10} = 40.07 (p < 0.01)													
Unclear													
Lin 2023 (USA)	44	38	FT=8h	Healthy	52	30	-3.49	5.45	30	1.12	5.45	-4.61 [-7.37; -1.85]	3.4
Pavlou 2023 (USA)	55	39	FT=8h	Metabolic	26	25	-4.52	4.83	25	-1.07	4.83	-3.45 [-6.13; -0.77]	3.5
Jamshed 2022 (USA)	43	40	FT=8h	Healthy	14	45	-6.30	3.39	45	-4.00	3.39	-2.30 [-3.70; -0.90]	6.8
Liu 2022 (China)	32	32	FT=8h	Healthy	52	69	-8.00	6.62	70	-6.20	6.62	-1.80 [-4.00; 0.40]	4.5
Wei 2023 (China)	32	32	FT=8h	Metabolic	52	45	-8.40	6.46	43	-7.80	6.46	-0.60 [-3.30; 2.10]	3.5
de Oliveira Maranhao Pureza 2021 (Brazil)	31	33	FT=12h	Healthy	52	31	-0.58	4.44	27	-0.53	4.44	-0.05 [-2.34; 2.24]	4.3
Overall Effect						245			240			-2.07 [-3.27; -0.87]	26.1
Heterogeneity: I^2 = 42%, τ^2 = 0.9134, χ^2_5 = 8.56 (p = 0.13)													
**Overall Effect**						**834**			**693**			**-1.38 [-2.00; -0.76]**	**100.0**

Heterogeneity: I^2 = 73%, τ^2 = 0.9355, χ^2_{16} = 59.81 (p < 0.01)
Test for overall effect: z = -4.39 (p < 0.01)
Test for subgroup differences: χ^2_1 = 1.85, df = 1 (p = 0.17)

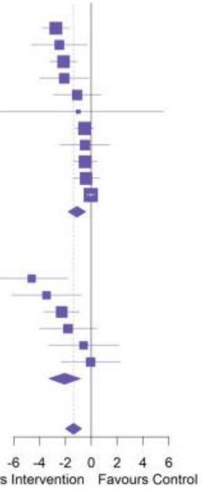

eFigure 77: Meta-analysis of difference in mean difference (95% CIs) for the effect of time restricted eating on weight (kg), grouped by risk of bias

BMI: Body Mass Index; SD: Standard Deviation; CI: Confidence Interval; USA: United States of America; FT: Feeding Time; Duration: follow-up duration in weeks; 2M/3M/6M: 2, 3 or 6 meals; 3M+2S: 3 meals and 2 snacks; 3M+3S: 3 meals and 3 snacks; 10S: Grazing diet – 100kcal every 2-3 hours; HCB: High Calorie Breakfast; HCD: High Calorie Dinner; ED: Early Dinner; LD: Late Dinner; FL: Front Loading: EED: Equal Energy Distribution; ML: Middle Loading; BL: Back Loading.

© 2024 Liu HY et al. *JAMA Network Open.*

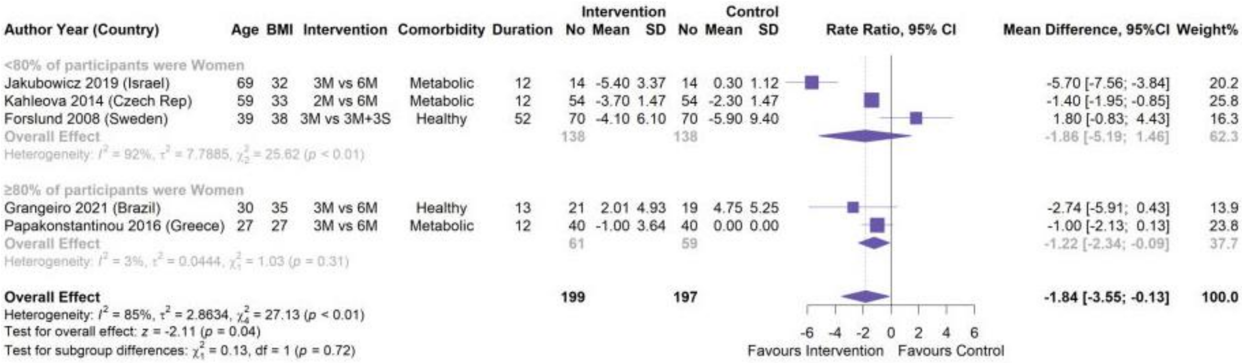

eFigure 78: Meta-analysis of difference in mean difference (95% CIs) for the effect of meal frequency on weight (kg), grouped by gender proportion

BMI: Body Mass Index; SD: Standard Deviation; CI: Confidence Interval; USA: United States of America; FT: Feeding Time; Duration: follow-up duration in weeks; 2M/3M/6M: 2, 3 or 6 meals; 3M+2S: 3 meals and 2 snacks; 3M+3S: 3 meals and 3 snacks; 10S: Grazing diet – 100kcal every 2-3 hours; HCB: High Calorie Breakfast; HCD: High Calorie Dinner; ED: Early Dinner; LD: Late Dinner; FL: Front Loading: EED: Equal Energy Distribution; ML: Middle Loading; BL: Back Loading.

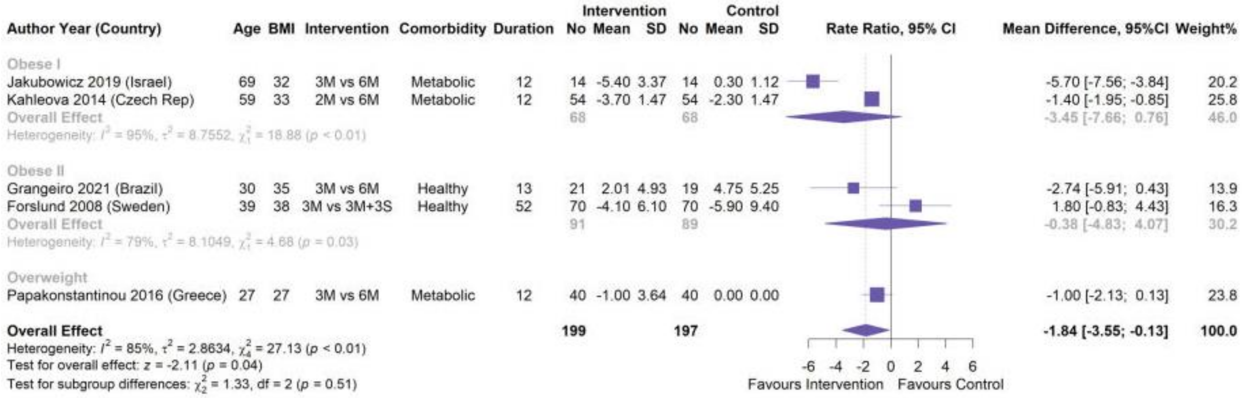

eFigure 79: Meta-analysis of difference in mean difference (95% CIs) for the effect of meal frequency on weight (kg), grouped by baseline BMI status

BMI: Body Mass Index; SD: Standard Deviation; CI: Confidence Interval; USA: United States of America; FT: Feeding Time; Duration: follow-up duration in weeks; 2M/3M/6M: 2, 3 or 6 meals; 3M+2S: 3 meals and 2 snacks; 3M+3S: 3 meals and 3 snacks; 10S: Grazing diet – 100kcal every 2-3 hours; HCB: High Calorie Breakfast; HCD: High Calorie Dinner; ED: Early Dinner; LD: Late Dinner; FL: Front Loading: EED: Equal Energy Distribution; ML: Middle Loading; BL: Back Loading.

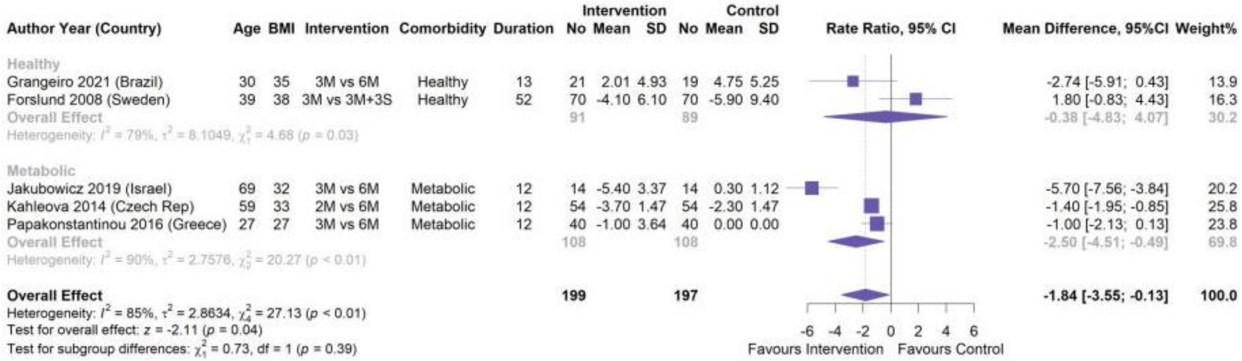

eFigure 80: Meta-analysis of difference in mean difference (95% CIs) for the effect of meal frequency on weight (kg), grouped by health status

© 2024 Liu HY et al. *JAMA Network Open.*

BMI: Body Mass Index; SD: Standard Deviation; CI: Confidence Interval; USA: United States of America; FT: Feeding Time; Duration: follow-up duration in weeks; 2M/3M/6M: 2, 3 or 6 meals; 3M+2S: 3 meals and 2 snacks; 3M+3S: 3 meals and 3 snacks; 10S: Grazing diet – 100kcal every 2-3 hours; HCB: High Calorie Breakfast; HCD: High Calorie Dinner; ED: Early Dinner; LD: Late Dinner; FL: Front Loading: EED: Equal Energy Distribution; ML: Middle Loading; BL: Back Loading.

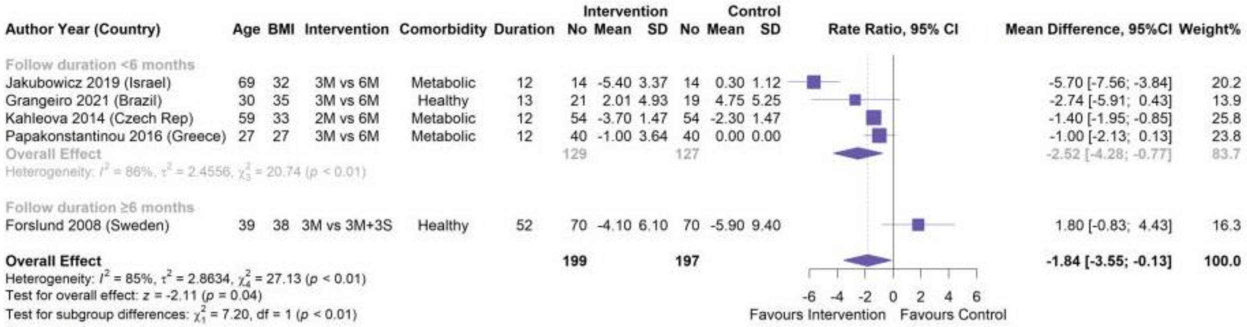

eFigure 81: Meta-analysis of difference in mean difference (95% CIs) for the effect of meal frequency on weight (kg), grouped by follow duration

BMI: Body Mass Index; SD: Standard Deviation; CI: Confidence Interval; USA: United States of America; FT: Feeding Time; Duration: follow-up duration in weeks; 2M/3M/6M: 2, 3 or 6 meals; 3M+2S: 3 meals and 2 snacks; 3M+3S: 3 meals and 3 snacks; 10S: Grazing diet – 100kcal every 2-3 hours; HCB: High Calorie Breakfast; HCD: High Calorie Dinner; ED: Early Dinner; LD: Late Dinner; FL: Front Loading: EED: Equal Energy Distribution; ML: Middle Loading; BL: Back Loading.

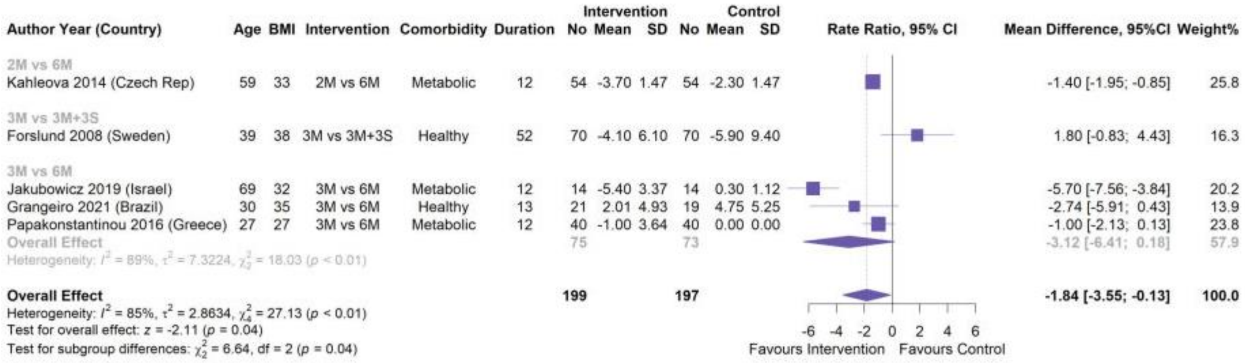

eFigure 82: Meta-analysis of difference in mean difference (95% CIs) for the effect of meal frequency on weight (kg), grouped by intervention intensity

BMI: Body Mass Index; SD: Standard Deviation; CI: Confidence Interval; USA: United States of America; FT: Feeding Time; Duration: follow-up duration in weeks; 2M/3M/6M: 2, 3 or 6 meals; 3M+2S: 3 meals and 2 snacks; 3M+3S: 3 meals and 3 snacks; 10S: Grazing diet – 100kcal every 2-3 hours; HCB: High Calorie Breakfast; HCD: High Calorie Dinner; ED: Early Dinner; LD: Late Dinner; FL: Front Loading: EED: Equal Energy Distribution; ML: Middle Loading; BL: Back Loading.

© 2024 Liu HY et al. *JAMA Network Open.*

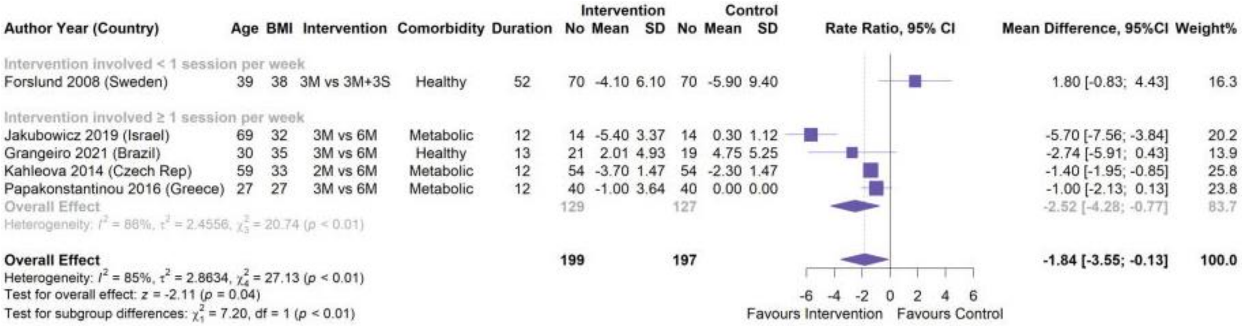

eFigure 83: Meta-analysis of difference in mean difference (95% CIs) for the effect of meal frequency on weight (kg), grouped by frequency of contact

BMI: Body Mass Index; SD: Standard Deviation; CI: Confidence Interval; USA: United States of America; FT: Feeding Time; Duration: follow-up duration in weeks; 2M/3M/6M: 2, 3 or 6 meals; 3M+2S: 3 meals and 2 snacks; 3M+3S: 3 meals and 3 snacks; 10S: Grazing diet – 100kcal every 2-3 hours; HCB: High Calorie Breakfast; HCD: High Calorie Dinner; ED: Early Dinner; LD: Late Dinner; FL: Front Loading: EED: Equal Energy Distribution; ML: Middle Loading; BL: Back Loading.

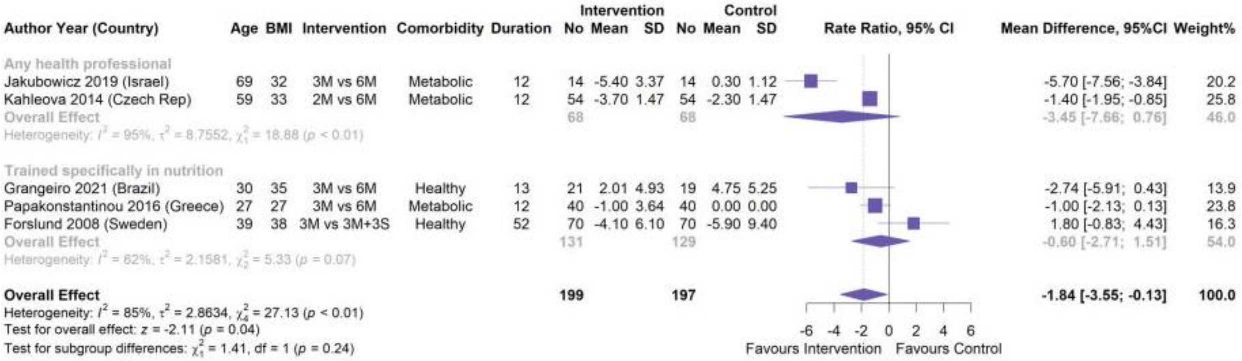

eFigure 84: Meta-analysis of difference in mean difference (95% CIs) for the effect of meal frequency on weight (kg), grouped by delivery personnel

BMI: Body Mass Index; SD: Standard Deviation; CI: Confidence Interval; USA: United States of America; FT: Feeding Time; Duration: follow-up duration in weeks; 2M/3M/6M: 2, 3 or 6 meals; 3M+2S: 3 meals and 2 snacks; 3M+3S: 3 meals and 3 snacks; 10S: Grazing diet – 100kcal every 2-3 hours; HCB: High Calorie Breakfast; HCD: High Calorie Dinner; ED: Early Dinner; LD: Late Dinner; FL: Front Loading: EED: Equal Energy Distribution; ML: Middle Loading; BL: Back Loading.

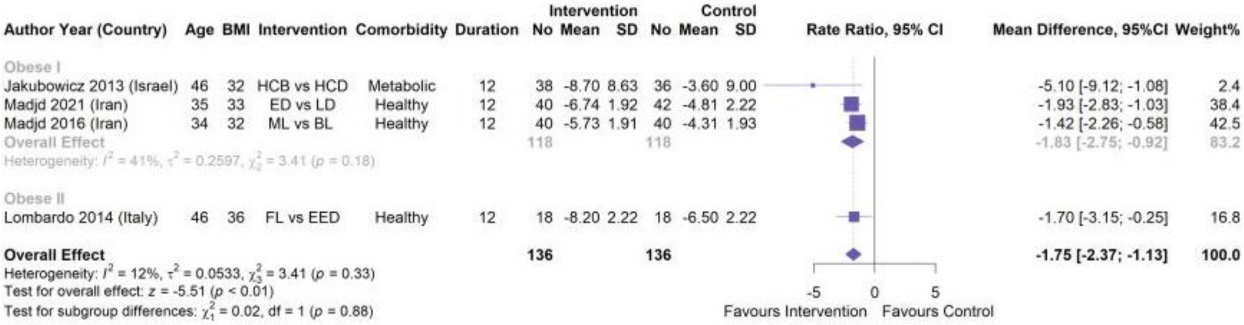

eFigure 85: Meta-analysis of difference in mean difference (95% CIs) for the effect of meal distribution on weight (kg), grouped by baseline BMI status

BMI: Body Mass Index; SD: Standard Deviation; CI: Confidence Interval; USA: United States of America; FT: Feeding Time; Duration: follow-up duration in weeks; 2M/3M/6M: 2, 3 or 6 meals; 3M+2S: 3 meals and 2 snacks; 3M+3S: 3 meals and 3 snacks; 10S: Grazing diet – 100kcal every 2-3 hours; HCB: High Calorie Breakfast; HCD: High Calorie Dinner; ED: Early Dinner; LD: Late Dinner; FL: Front Loading: EED: Equal Energy Distribution; ML: Middle Loading; BL: Back Loading.

© 2024 Liu HY et al. *JAMA Network Open.*

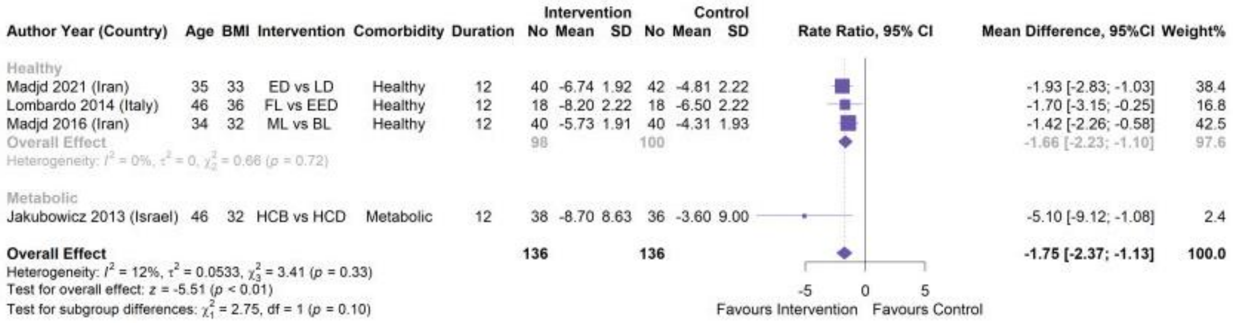

eFigure 86: Meta-analysis of difference in mean difference (95% CIs) for the effect of meal distribution on weight (kg), grouped by health status

BMI: Body Mass Index; SD: Standard Deviation; CI: Confidence Interval; USA: United States of America; FT: Feeding Time; Duration: follow-up duration in weeks; 2M/3M/6M: 2, 3 or 6 meals; 3M+2S: 3 meals and 2 snacks; 3M+3S: 3 meals and 3 snacks; 10S: Grazing diet – 100kcal every 2-3 hours; HCB: High Calorie Breakfast; HCD: High Calorie Dinner; ED: Early Dinner; LD: Late Dinner; FL: Front Loading: EED: Equal Energy Distribution; ML: Middle Loading; BL: Back Loading.

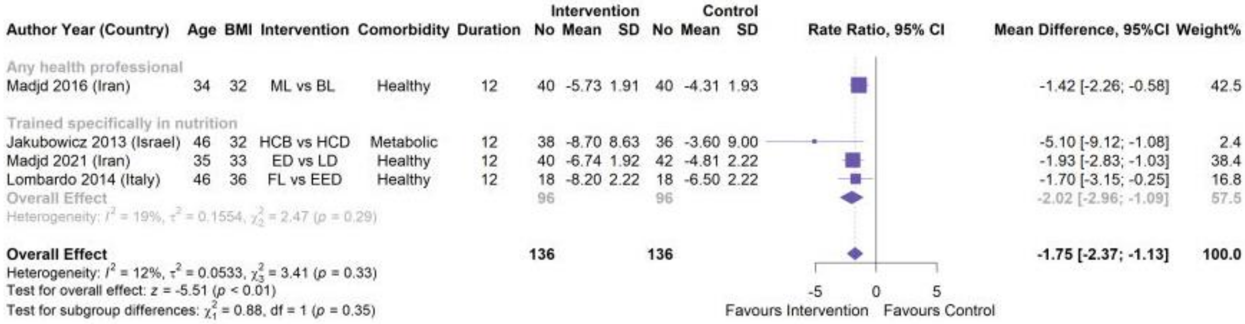

eFigure 87: Meta-analysis of difference in mean difference (95% CIs) for the effect of meal distribution on weight (kg), grouped by delivery personnel

BMI: Body Mass Index; SD: Standard Deviation; CI: Confidence Interval; USA: United States of America; FT: Feeding Time; Duration: follow-up duration in weeks; 2M/3M/6M: 2, 3 or 6 meals; 3M+2S: 3 meals and 2 snacks; 3M+3S: 3 meals and 3 snacks; 10S: Grazing diet – 100kcal every 2-3 hours; HCB: High Calorie Breakfast; HCD: High Calorie Dinner; ED: Early Dinner; LD: Late Dinner; FL: Front Loading: EED: Equal Energy Distribution; ML: Middle Loading; BL: Back Loading.

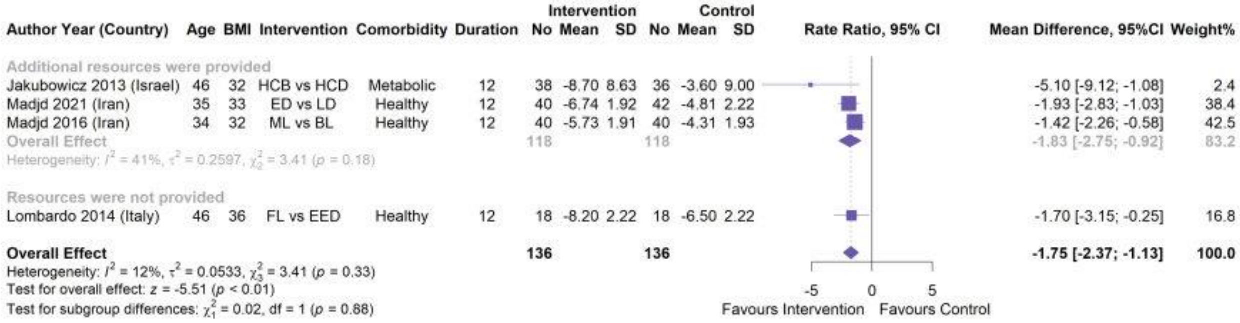

eFigure 88: Meta-analysis of difference in mean difference (95% CIs) for the effect of meal distribution on weight (kg), grouped by resource provision

BMI: Body Mass Index; SD: Standard Deviation; CI: Confidence Interval; USA: United States of America; FT: Feeding Time; Duration: follow-up duration in weeks; 2M/3M/6M: 2, 3 or 6 meals; 3M+2S: 3 meals and 2 snacks; 3M+3S: 3 meals and 3 snacks; 10S: Grazing diet – 100kcal every 2-3 hours; HCB: High Calorie Breakfast; HCD: High Calorie Dinner; ED: Early Dinner; LD: Late Dinner; FL: Front Loading: EED: Equal Energy Distribution; ML: Middle Loading; BL: Back Loading.

© 2024 Liu HY et al. *JAMA Network Open.*

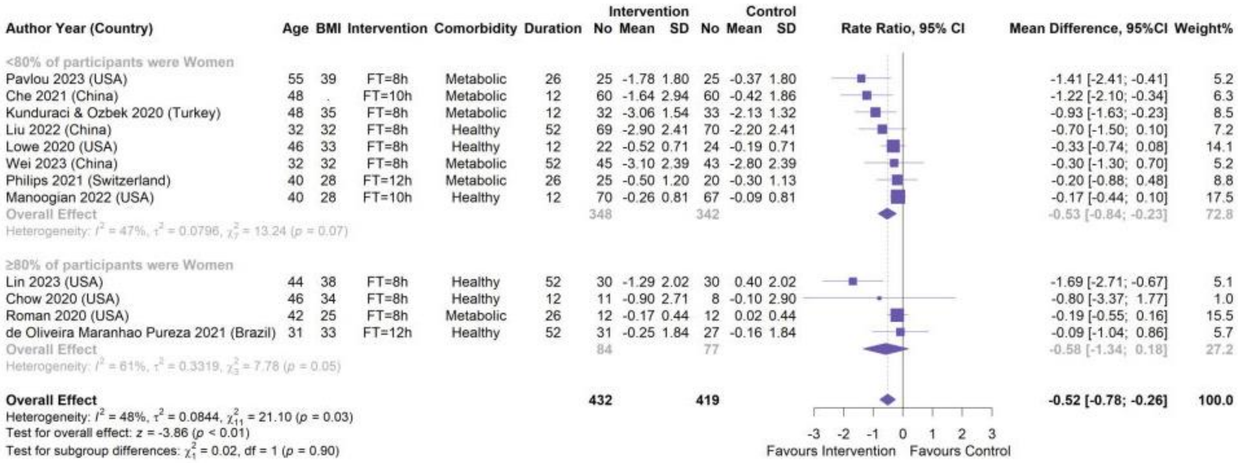

eFigure 89: Meta-analysis of difference in mean difference (95% CIs) for the effect of time restricted eating on BMI (kg/m²), grouped by gender proportion

BMI: Body Mass Index; SD: Standard Deviation; CI: Confidence Interval; USA: United States of America; FT: Feeding Time; Duration: follow-up duration in weeks; 2M/3M/6M: 2, 3 or 6 meals; 3M+2S: 3 meals and 2 snacks; 3M+3S: 3 meals and 3 snacks; 10S: Grazing diet – 100kcal every 2-3 hours; HCB: High Calorie Breakfast; HCD: High Calorie Dinner; ED: Early Dinner; LD: Late Dinner; FL: Front Loading: EED: Equal Energy Distribution; ML: Middle Loading; BL: Back Loading.

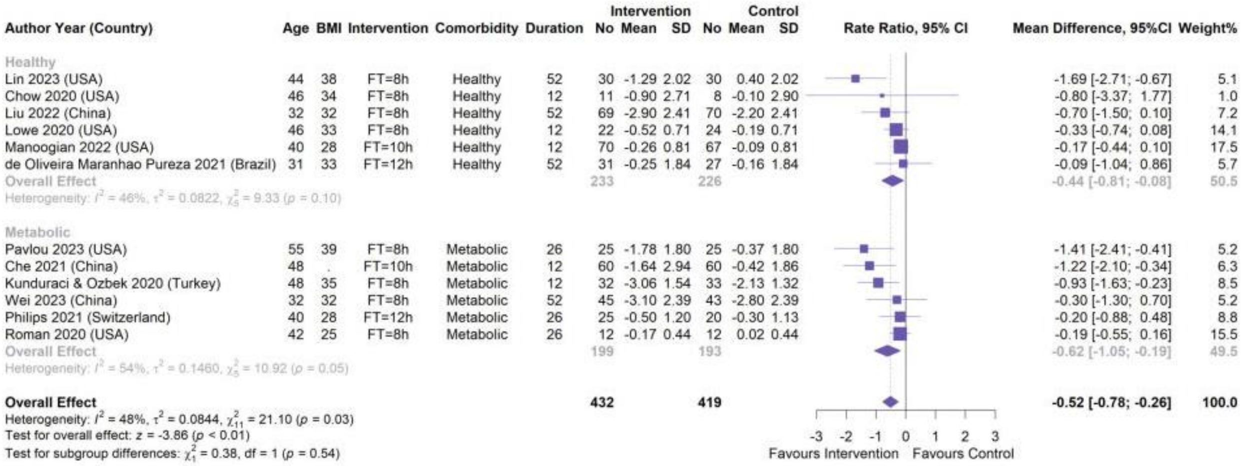

eFigure 90: Meta-analysis of difference in mean difference (95% CIs) for the effect of time restricted eating on BMI (kg/m²), grouped by health status

BMI: Body Mass Index; SD: Standard Deviation; CI: Confidence Interval; USA: United States of America; FT: Feeding Time; Duration: follow-up duration in weeks; 2M/3M/6M: 2, 3 or 6 meals; 3M+2S: 3 meals and 2 snacks; 3M+3S: 3 meals and 3 snacks; 10S: Grazing diet – 100kcal every 2-3 hours; HCB: High Calorie Breakfast; HCD: High Calorie Dinner; ED: Early Dinner; LD: Late Dinner; FL: Front Loading: EED: Equal Energy Distribution; ML: Middle Loading; BL: Back Loading.

© 2024 Liu HY et al. *JAMA Network Open.*

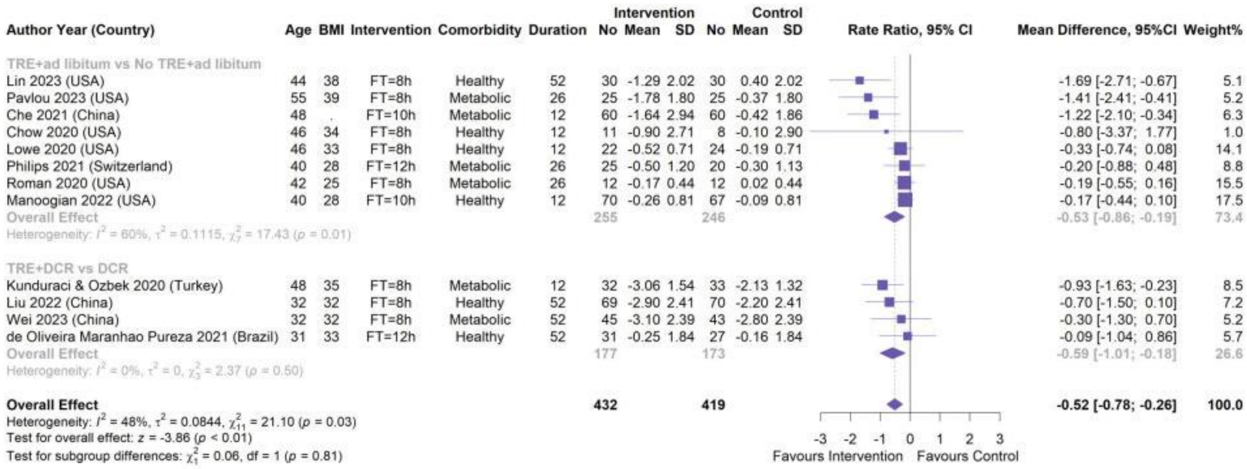

eFigure 91: Meta-analysis of difference in mean difference (95% CIs) for the effect of time restricted eating on BMI (kg/m^2), grouped by energy prescription

BMI: Body Mass Index; SD: Standard Deviation; CI: Confidence Interval; USA: United States of America; FT: Feeding Time; Duration: follow-up duration in weeks; 2M/3M/6M: 2, 3 or 6 meals; 3M+2S: 3 meals and 2 snacks; 3M+3S: 3 meals and 3 snacks; 10S: Grazing diet – 100kcal every 2-3 hours; HCB: High Calorie Breakfast; HCD: High Calorie Dinner; ED: Early Dinner; LD: Late Dinner; FL: Front Loading: EED: Equal Energy Distribution; ML: Middle Loading; BL: Back Loading.

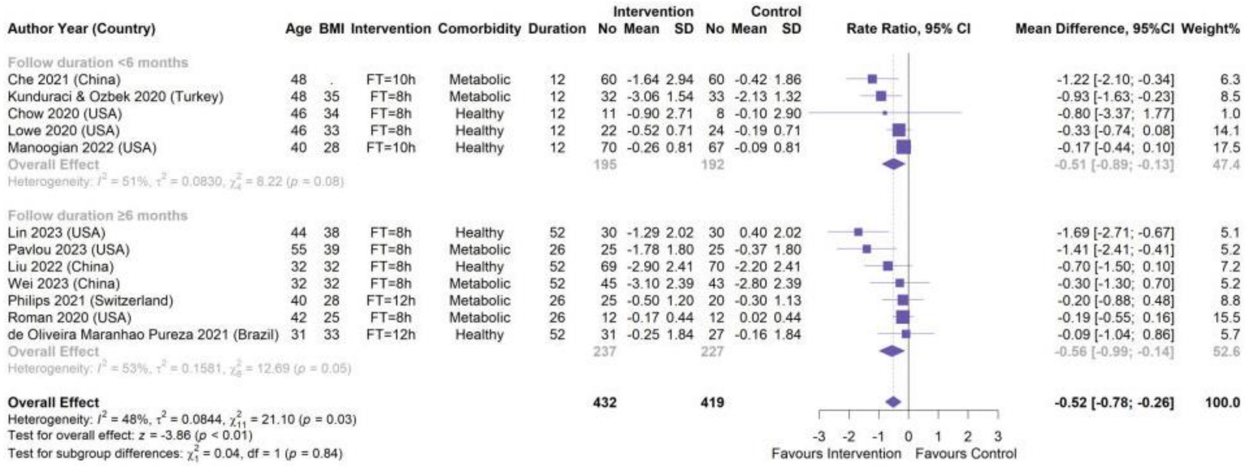

eFigure 92: Meta-analysis of difference in mean difference (95% CIs) for the effect of time restricted eating on BMI (kg/m^2), grouped by follow duration

BMI: Body Mass Index; SD: Standard Deviation; CI: Confidence Interval; USA: United States of America; FT: Feeding Time; Duration: follow-up duration in weeks; 2M/3M/6M: 2, 3 or 6 meals; 3M+2S: 3 meals and 2 snacks; 3M+3S: 3 meals and 3 snacks; 10S: Grazing diet – 100kcal every 2-3 hours; HCB: High Calorie Breakfast; HCD: High Calorie Dinner; ED: Early Dinner; LD: Late Dinner; FL: Front Loading: EED: Equal Energy Distribution; ML: Middle Loading; BL: Back Loading.

© 2024 Liu HY et al. *JAMA Network Open.*

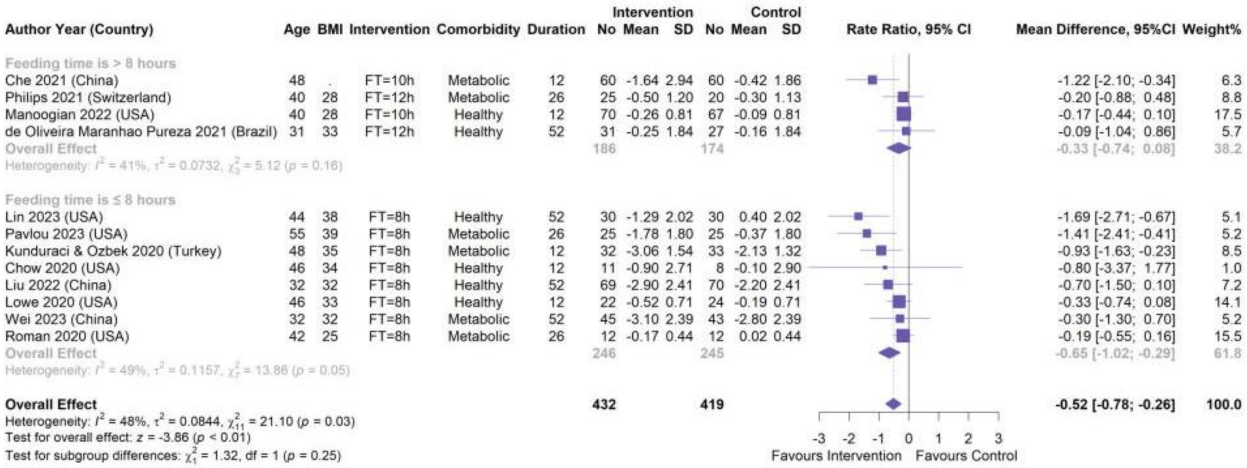

eFigure 93: Meta-analysis of difference in mean difference (95% CIs) for the effect of time restricted eating on BMI (kg/m²), grouped by eating window

BMI: Body Mass Index; SD: Standard Deviation; CI: Confidence Interval; USA: United States of America; FT: Feeding Time; Duration: follow-up duration in weeks; 2M/3M/6M: 2, 3 or 6 meals; 3M+2S: 3 meals and 2 snacks; 3M+3S: 3 meals and 3 snacks; 10S: Grazing diet – 100kcal every 2-3 hours; HCB: High Calorie Breakfast; HCD: High Calorie Dinner; ED: Early Dinner; LD: Late Dinner; FL: Front Loading: EED: Equal Energy Distribution; ML: Middle Loading; BL: Back Loading.

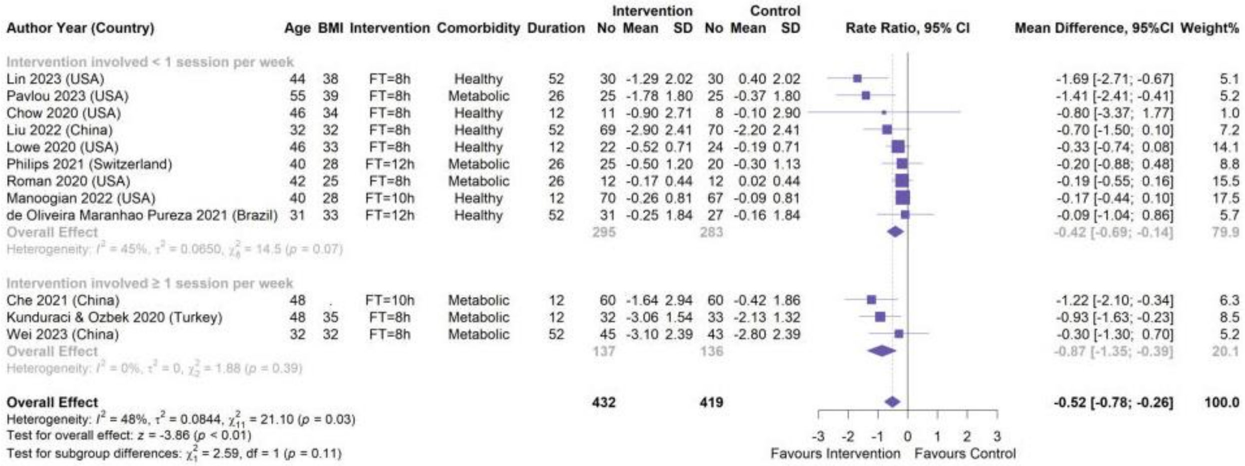

eFigure 94: Meta-analysis of difference in mean difference (95% CIs) for the effect of time restricted eating on BMI (kg/m²), grouped by frequency of contact

BMI: Body Mass Index; SD: Standard Deviation; CI: Confidence Interval; USA: United States of America; FT: Feeding Time; Duration: follow-up duration in weeks; 2M/3M/6M: 2, 3 or 6 meals; 3M+2S: 3 meals and 2 snacks; 3M+3S: 3 meals and 3 snacks; 10S: Grazing diet – 100kcal every 2-3 hours; HCB: High Calorie Breakfast; HCD: High Calorie Dinner; ED: Early Dinner; LD: Late Dinner; FL: Front Loading: EED: Equal Energy Distribution; ML: Middle Loading; BL: Back Loading.

© 2024 Liu HY et al. *JAMA Network Open.*

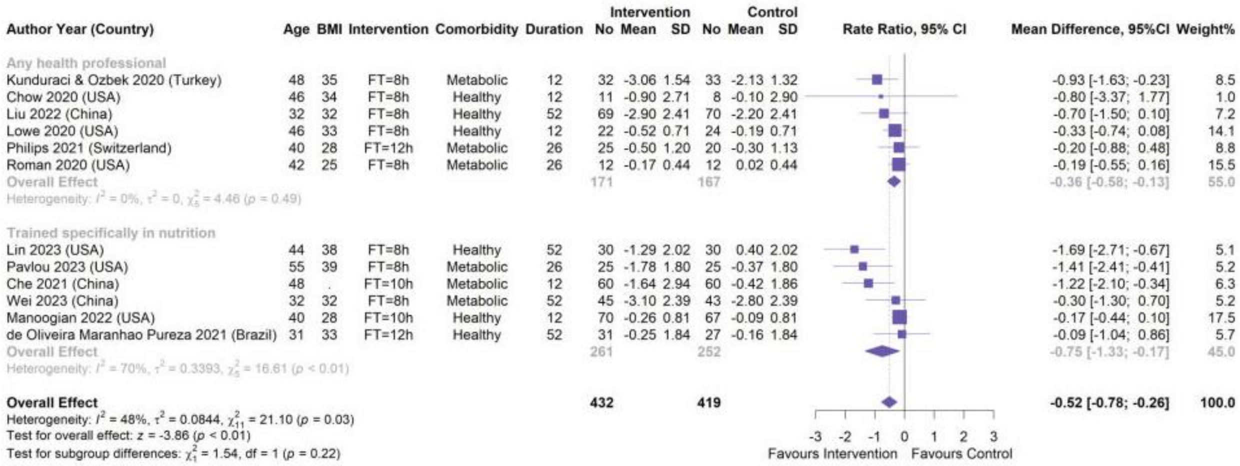

eFigure 95: Meta-analysis of difference in mean difference (95% CIs) for the effect of time restricted eating on BMI (kg/m²), grouped by delivery personnel

BMI: Body Mass Index; SD: Standard Deviation; CI: Confidence Interval; USA: United States of America; FT: Feeding Time; Duration: follow-up duration in weeks; 2M/3M/6M: 2, 3 or 6 meals; 3M+2S: 3 meals and 2 snacks; 3M+3S: 3 meals and 3 snacks; 10S: Grazing diet – 100kcal every 2-3 hours; HCB: High Calorie Breakfast; HCD: High Calorie Dinner; ED: Early Dinner; LD: Late Dinner; FL: Front Loading: EED: Equal Energy Distribution; ML: Middle Loading; BL: Back Loading.

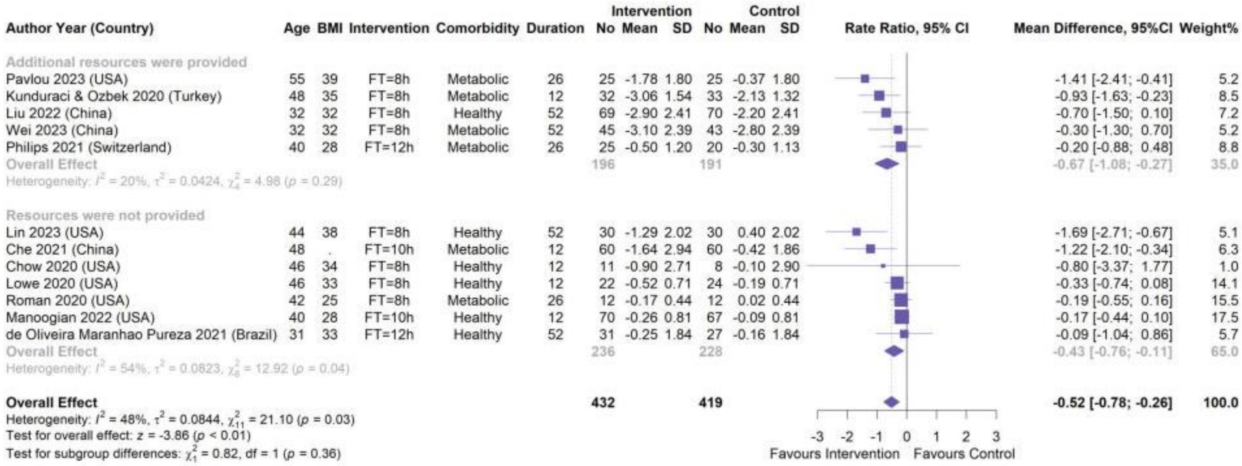

eFigure 96: Meta-analysis of difference in mean difference (95% CIs) for the effect of time restricted eating on BMI (kg/m²), grouped by resource provision

BMI: Body Mass Index; SD: Standard Deviation; CI: Confidence Interval; USA: United States of America; FT: Feeding Time; Duration: follow-up duration in weeks; 2M/3M/6M: 2, 3 or 6 meals; 3M+2S: 3 meals and 2 snacks; 3M+3S: 3 meals and 3 snacks; 10S: Grazing diet – 100kcal every 2-3 hours; HCB: High Calorie Breakfast; HCD: High Calorie Dinner; ED: Early Dinner; LD: Late Dinner; FL: Front Loading: EED: Equal Energy Distribution; ML: Middle Loading; BL: Back Loading.

© 2024 Liu HY et al. *JAMA Network Open.*

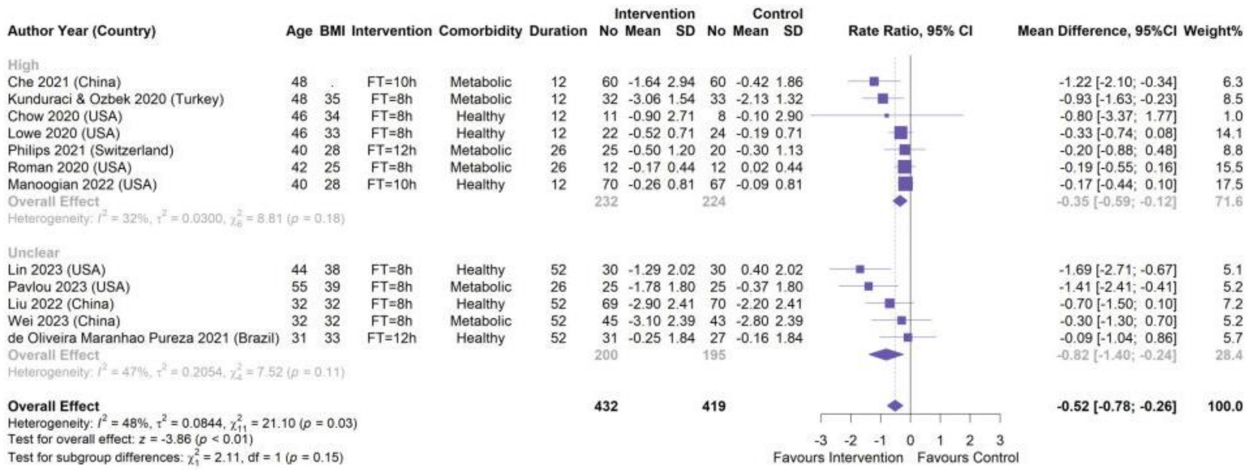

eFigure 97: Meta-analysis of difference in mean difference (95% CIs) for the effect of time restricted eating on BMI (kg/m^2), grouped by risk of bias

BMI: Body Mass Index; SD: Standard Deviation; CI: Confidence Interval; USA: United States of America; FT: Feeding Time; Duration: follow-up duration in weeks; 2M/3M/6M: 2, 3 or 6 meals; 3M+2S: 3 meals and 2 snacks; 3M+3S: 3 meals and 3 snacks; 10S: Grazing diet – 100kcal every 2-3 hours; HCB: High Calorie Breakfast; HCD: High Calorie Dinner; ED: Early Dinner; LD: Late Dinner; FL: Front Loading: EED: Equal Energy Distribution; ML: Middle Loading; BL: Back Loading.

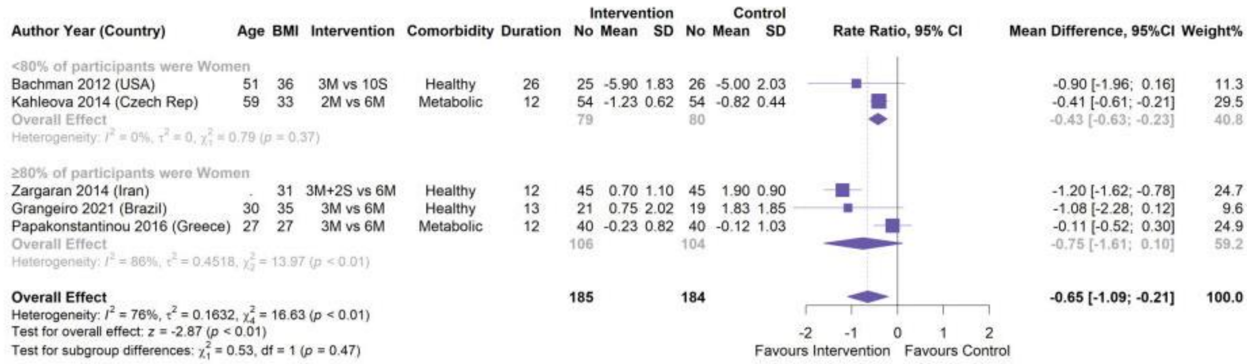

eFigure 98: Meta-analysis of difference in mean difference (95% CIs) for the effect of meal frequency on BMI (kg/m^2), grouped by gender proportion

BMI: Body Mass Index; SD: Standard Deviation; CI: Confidence Interval; USA: United States of America; FT: Feeding Time; Duration: follow-up duration in weeks; 2M/3M/6M: 2, 3 or 6 meals; 3M+2S: 3 meals and 2 snacks; 3M+3S: 3 meals and 3 snacks; 10S: Grazing diet – 100kcal every 2-3 hours; HCB: High Calorie Breakfast; HCD: High Calorie Dinner; ED: Early Dinner; LD: Late Dinner; FL: Front Loading: EED: Equal Energy Distribution; ML: Middle Loading; BL: Back Loading.

© 2024 Liu HY et al. *JAMA Network Open.*

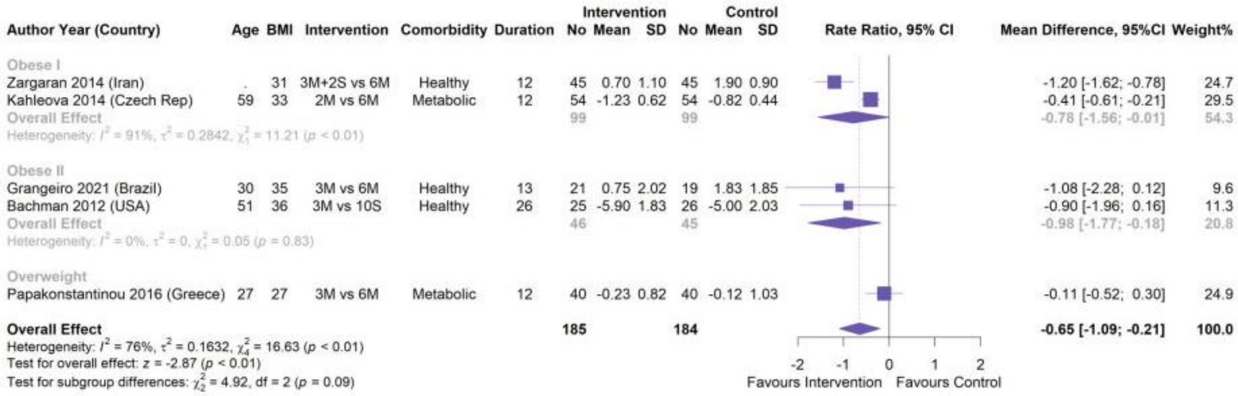

eFigure 99: Meta-analysis of difference in mean difference (95% CIs) for the effect of meal frequency on BMI (kg/m²), grouped by baseline BMI status

BMI: Body Mass Index; SD: Standard Deviation; CI: Confidence Interval; USA: United States of America; FT: Feeding Time; Duration: follow-up duration in weeks; 2M/3M/6M: 2, 3 or 6 meals; 3M+2S: 3 meals and 2 snacks; 3M+3S: 3 meals and 3 snacks; 10S: Grazing diet – 100kcal every 2-3 hours; HCB: High Calorie Breakfast; HCD: High Calorie Dinner; ED: Early Dinner; LD: Late Dinner; FL: Front Loading: EED: Equal Energy Distribution; ML: Middle Loading; BL: Back Loading.

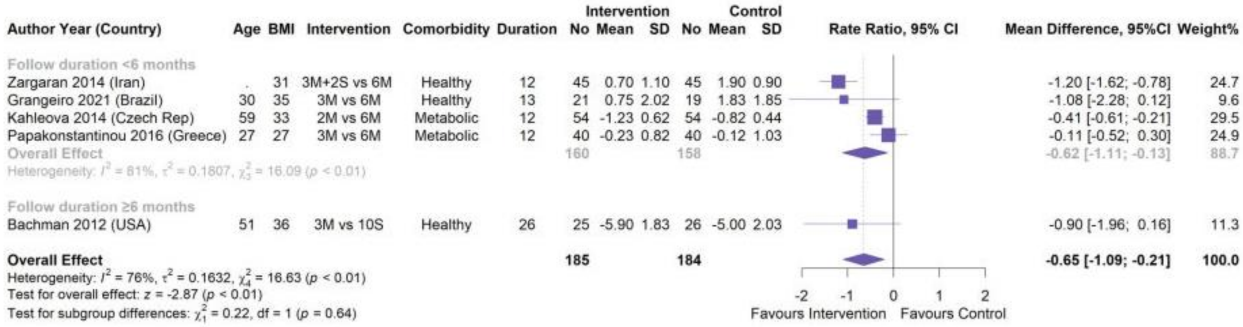

eFigure 100: Meta-analysis of difference in mean difference (95% CIs) for the effect of meal frequency on BMI (kg/m²), grouped by follow duration

BMI: Body Mass Index; SD: Standard Deviation; CI: Confidence Interval; USA: United States of America; FT: Feeding Time; Duration: follow-up duration in weeks; 2M/3M/6M: 2, 3 or 6 meals; 3M+2S: 3 meals and 2 snacks; 3M+3S: 3 meals and 3 snacks; 10S: Grazing diet – 100kcal every 2-3 hours; HCB: High Calorie Breakfast; HCD: High Calorie Dinner; ED: Early Dinner; LD: Late Dinner; FL: Front Loading: EED: Equal Energy Distribution; ML: Middle Loading; BL: Back Loading.

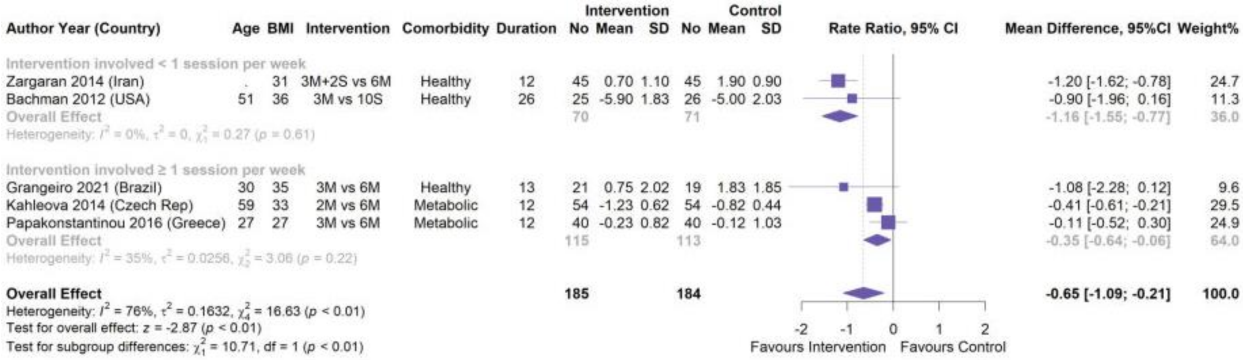

eFigure 101: Meta-analysis of difference in mean difference (95% CIs) for the effect of meal frequency on BMI (kg/m²), grouped by frequency of contact

BMI: Body Mass Index; SD: Standard Deviation; CI: Confidence Interval; USA: United States of America; FT: Feeding Time; Duration: follow-up duration in weeks; 2M/3M/6M: 2, 3 or 6 meals; 3M+2S: 3 meals and 2 snacks; 3M+3S: 3 meals and 3 snacks; 10S: Grazing diet

© 2024 Liu HY et al. *JAMA Network Open.*

– 100kcal every 2-3 hours; HCB: High Calorie Breakfast; HCD: High Calorie Dinner; ED: Early Dinner; LD: Late Dinner; FL: Front Loading: EED: Equal Energy Distribution; ML: Middle Loading; BL: Back Loading.

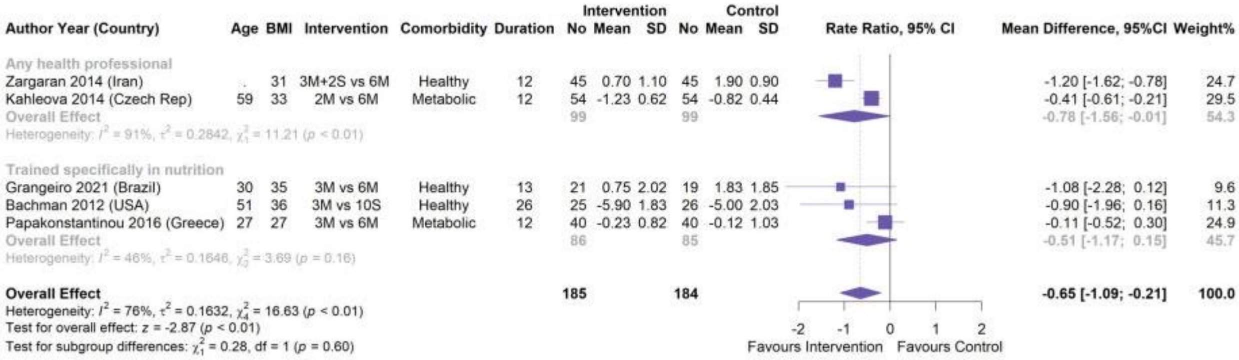

eFigure 102: Meta-analysis of difference in mean difference (95% CIs) for the effect of meal frequency on BMI (kg/m²), grouped by delivery personnel

BMI: Body Mass Index; SD: Standard Deviation; CI: Confidence Interval; USA: United States of America; FT: Feeding Time; Duration: follow-up duration in weeks; 2M/3M/6M: 2, 3 or 6 meals; 3M+2S: 3 meals and 2 snacks; 3M+3S: 3 meals and 3 snacks; 10S: Grazing diet – 100kcal every 2-3 hours; HCB: High Calorie Breakfast; HCD: High Calorie Dinner; ED: Early Dinner; LD: Late Dinner; FL: Front Loading: EED: Equal Energy Distribution; ML: Middle Loading; BL: Back Loading.

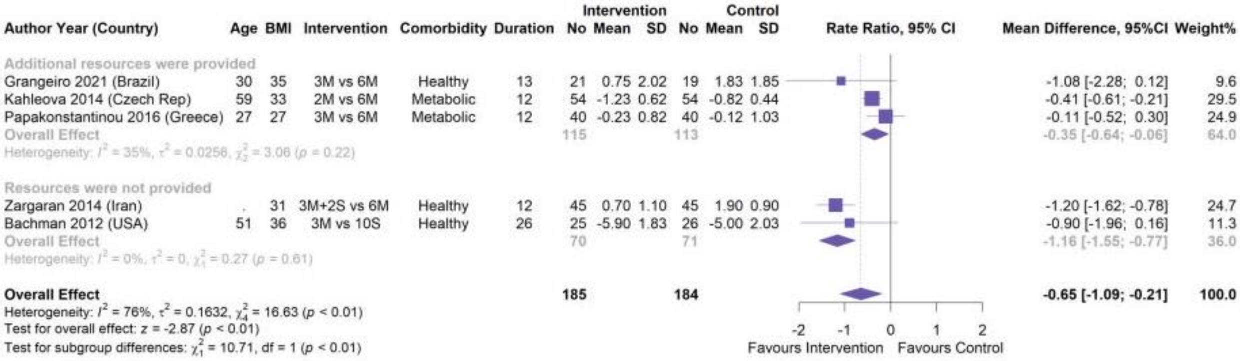

eFigure 103: Meta-analysis of difference in mean difference (95% CIs) for the effect of meal frequency on BMI (kg/m²), grouped by resource provision

BMI: Body Mass Index; SD: Standard Deviation; CI: Confidence Interval; USA: United States of America; FT: Feeding Time; Duration: follow-up duration in weeks; 2M/3M/6M: 2, 3 or 6 meals; 3M+2S: 3 meals and 2 snacks; 3M+3S: 3 meals and 3 snacks; 10S: Grazing diet – 100kcal every 2-3 hours; HCB: High Calorie Breakfast; HCD: High Calorie Dinner; ED: Early Dinner; LD: Late Dinner; FL: Front Loading: EED: Equal Energy Distribution; ML: Middle Loading; BL: Back Loading.

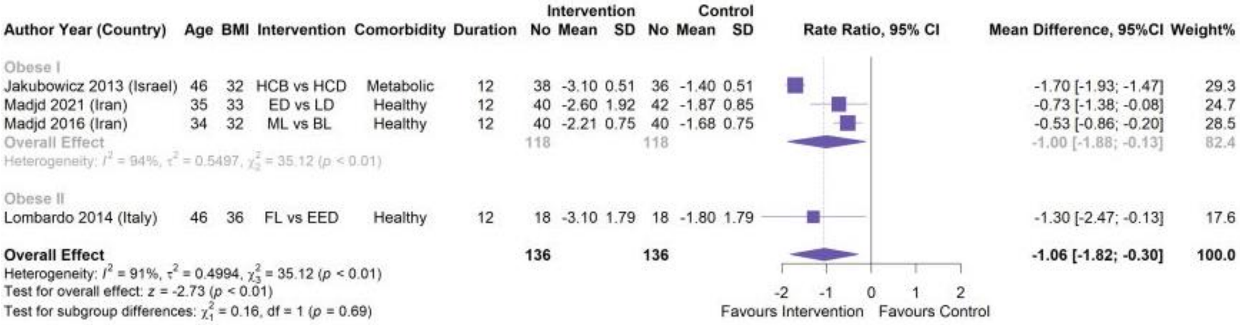

eFigure 104: Meta-analysis of difference in mean difference (95% CIs) for the effect of meal distribution on BMI (kg/m²), grouped by baseline BMI status

© 2024 Liu HY et al. *JAMA Network Open.*

BMI: Body Mass Index; SD: Standard Deviation; CI: Confidence Interval; USA: United States of America; FT: Feeding Time; Duration: follow-up duration in weeks; 2M/3M/6M: 2, 3 or 6 meals; 3M+2S: 3 meals and 2 snacks; 3M+3S: 3 meals and 3 snacks; 10S: Grazing diet – 100kcal every 2-3 hours; HCB: High Calorie Breakfast; HCD: High Calorie Dinner; ED: Early Dinner; LD: Late Dinner; FL: Front Loading: EED: Equal Energy Distribution; ML: Middle Loading; BL: Back Loading.

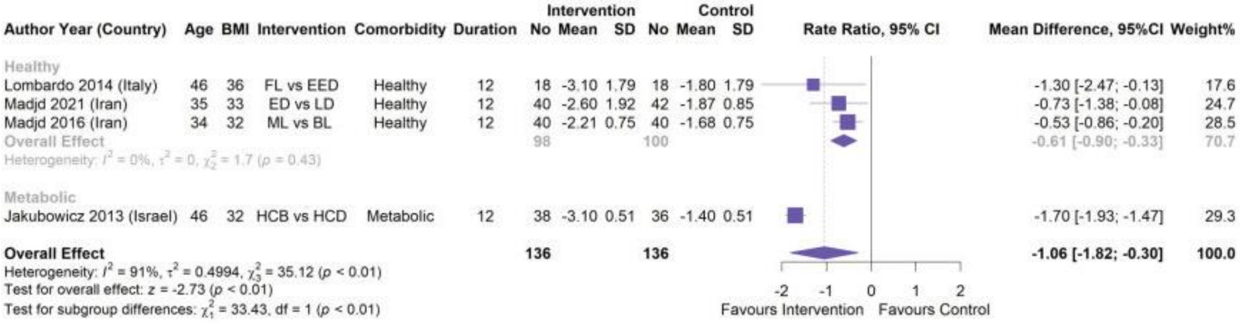

eFigure 105: Meta-analysis of difference in mean difference (95% CIs) for the effect of meal distribution on BMI (kg/m²), grouped by health status

BMI: Body Mass Index; SD: Standard Deviation; CI: Confidence Interval; USA: United States of America; FT: Feeding Time; Duration: follow-up duration in weeks; 2M/3M/6M: 2, 3 or 6 meals; 3M+2S: 3 meals and 2 snacks; 3M+3S: 3 meals and 3 snacks; 10S: Grazing diet – 100kcal every 2-3 hours; HCB: High Calorie Breakfast; HCD: High Calorie Dinner; ED: Early Dinner; LD: Late Dinner; FL: Front Loading: EED: Equal Energy Distribution; ML: Middle Loading; BL: Back Loading.

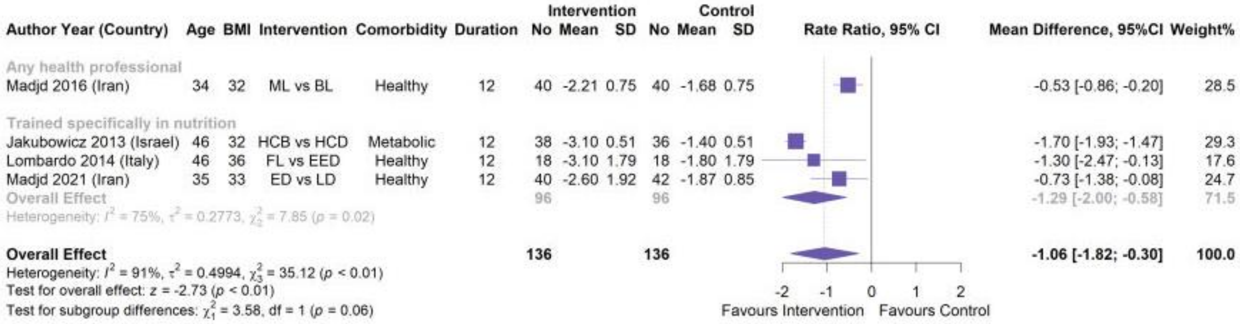

eFigure 106: Meta-analysis of difference in mean difference (95% CIs) for the effect of meal distribution on BMI (kg/m²), grouped by delivery personnel

BMI: Body Mass Index; SD: Standard Deviation; CI: Confidence Interval; USA: United States of America; FT: Feeding Time; Duration: follow-up duration in weeks; 2M/3M/6M: 2, 3 or 6 meals; 3M+2S: 3 meals and 2 snacks; 3M+3S: 3 meals and 3 snacks; 10S: Grazing diet – 100kcal every 2-3 hours; HCB: High Calorie Breakfast; HCD: High Calorie Dinner; ED: Early Dinner; LD: Late Dinner; FL: Front Loading: EED: Equal Energy Distribution; ML: Middle Loading; BL: Back Loading.

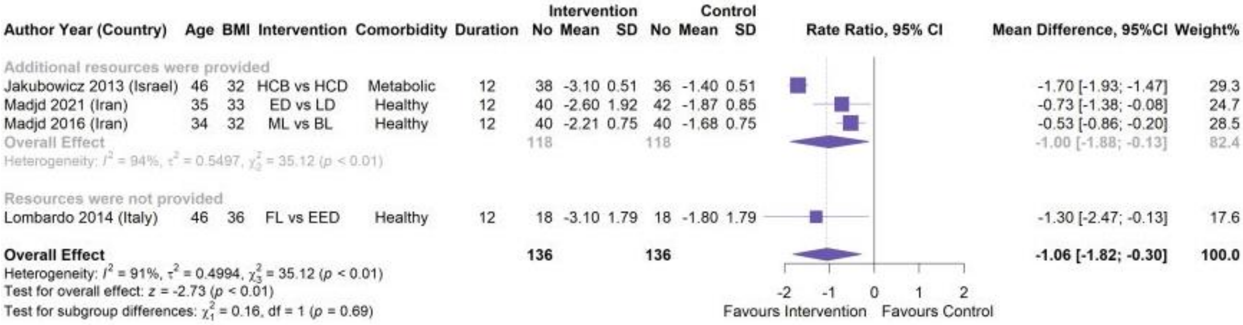

eFigure 107: Meta-analysis of difference in mean difference (95% CIs) for the effect of meal distribution on BMI (kg/m²), grouped by resource provision

© 2024 Liu HY et al. *JAMA Network Open.*

BMI: Body Mass Index; SD: Standard Deviation; CI: Confidence Interval; USA: United States of America; FT: Feeding Time; Duration: follow-up duration in weeks; 2M/3M/6M: 2, 3 or 6 meals; 3M+2S: 3 meals and 2 snacks; 3M+3S: 3 meals and 3 snacks; 10S: Grazing diet – 100kcal every 2-3 hours; HCB: High Calorie Breakfast; HCD: High Calorie Dinner; ED: Early Dinner; LD: Late Dinner; FL: Front Loading: EED: Equal Energy Distribution; ML: Middle Loading; BL: Back Loading.

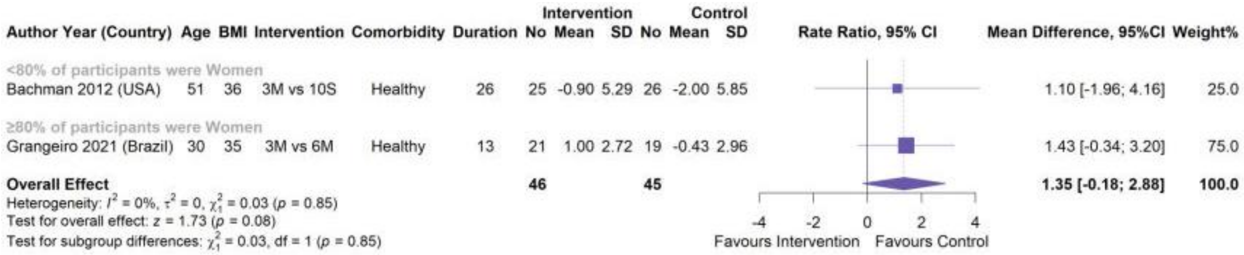

eFigure 108: Meta-analysis of difference in mean difference (95% CIs) for the effect of meal frequency on lean mass (kg), grouped by gender proportion

BMI: Body Mass Index; SD: Standard Deviation; CI: Confidence Interval; USA: United States of America; FT: Feeding Time; Duration: follow-up duration in weeks; 2M/3M/6M: 2, 3 or 6 meals; 3M+2S: 3 meals and 2 snacks; 3M+3S: 3 meals and 3 snacks; 10S: Grazing diet – 100kcal every 2-3 hours; HCB: High Calorie Breakfast; HCD: High Calorie Dinner; ED: Early Dinner; LD: Late Dinner; FL: Front Loading: EED: Equal Energy Distribution; ML: Middle Loading; BL: Back Loading.

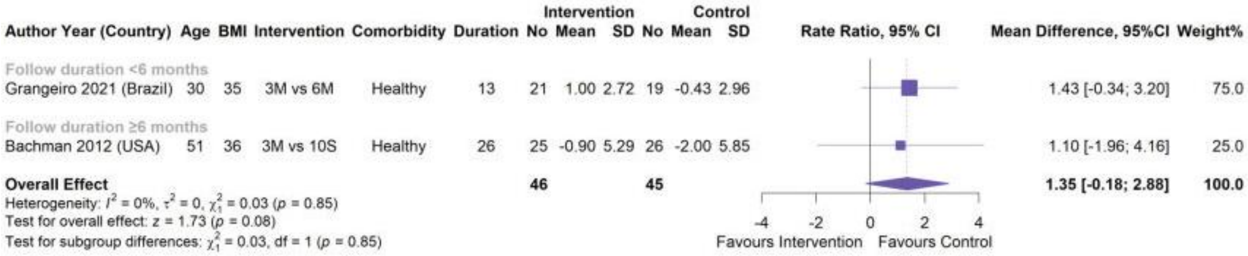

eFigure 109: Meta-analysis of difference in mean difference (95% CIs) for the effect of meal frequency on lean mass (kg), grouped by follow duration

BMI: Body Mass Index; SD: Standard Deviation; CI: Confidence Interval; USA: United States of America; FT: Feeding Time; Duration: follow-up duration in weeks; 2M/3M/6M: 2, 3 or 6 meals; 3M+2S: 3 meals and 2 snacks; 3M+3S: 3 meals and 3 snacks; 10S: Grazing diet – 100kcal every 2-3 hours; HCB: High Calorie Breakfast; HCD: High Calorie Dinner; ED: Early Dinner; LD: Late Dinner; FL: Front Loading: EED: Equal Energy Distribution; ML: Middle Loading; BL: Back Loading.

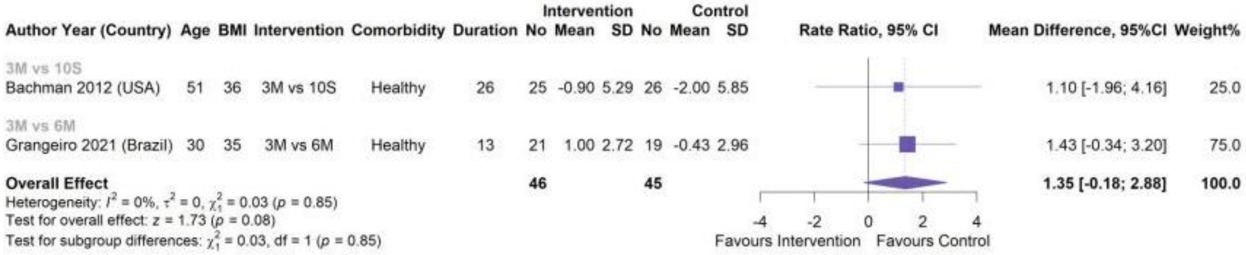

eFigure 110: Meta-analysis of difference in mean difference (95% CIs) for the effect of meal frequency on lean mass (kg), grouped by intervention intensity

BMI: Body Mass Index; SD: Standard Deviation; CI: Confidence Interval; USA: United States of America; FT: Feeding Time; Duration: follow-up duration in weeks; 2M/3M/6M: 2, 3 or 6 meals; 3M+2S: 3 meals and 2 snacks; 3M+3S: 3 meals and 3 snacks; 10S: Grazing diet – 100kcal every 2-3 hours; HCB: High Calorie Breakfast; HCD: High Calorie Dinner; ED: Early Dinner; LD: Late Dinner; FL: Front Loading: EED: Equal Energy Distribution; ML: Middle Loading; BL: Back Loading.

© 2024 Liu HY et al. *JAMA Network Open.*

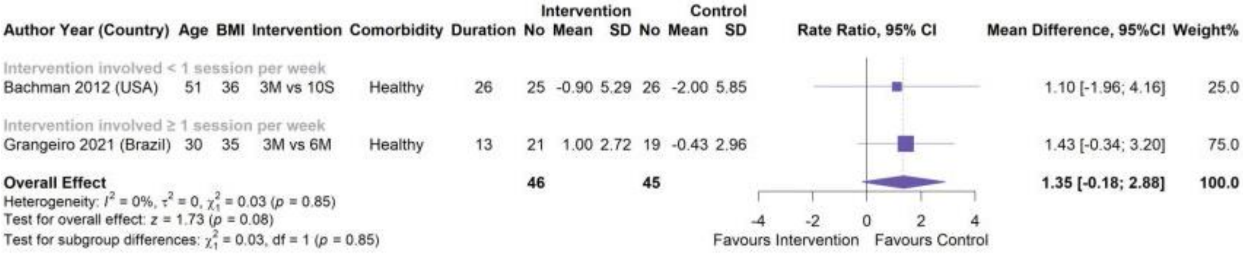

eFigure 111: Meta-analysis of difference in mean difference (95% CIs) for the effect of meal frequency on lean mass (kg), grouped by frequency of contact

BMI: Body Mass Index; SD: Standard Deviation; CI: Confidence Interval; USA: United States of America; FT: Feeding Time; Duration: follow-up duration in weeks; 2M/3M/6M: 2, 3 or 6 meals; 3M+2S: 3 meals and 2 snacks; 3M+3S: 3 meals and 3 snacks; 10S: Grazing diet – 100kcal every 2-3 hours; HCB: High Calorie Breakfast; HCD: High Calorie Dinner; ED: Early Dinner; LD: Late Dinner; FL: Front Loading: EED: Equal Energy Distribution; ML: Middle Loading; BL: Back Loading.

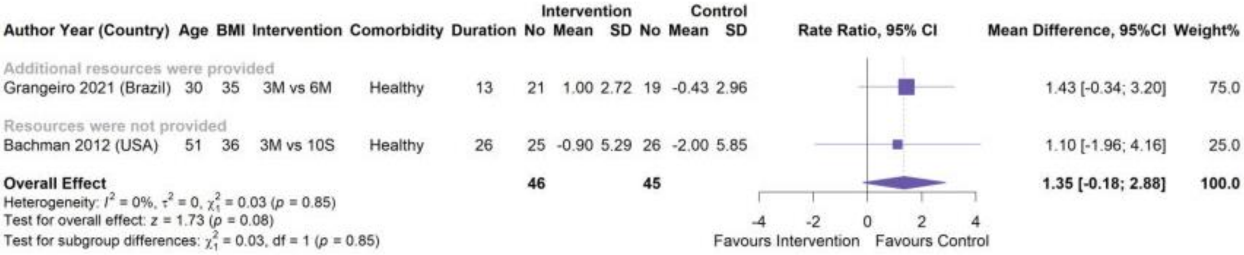

eFigure 112: Meta-analysis of difference in mean difference (95% CIs) for the effect of meal frequency on lean mass (kg), grouped by resource provision

BMI: Body Mass Index; SD: Standard Deviation; CI: Confidence Interval; USA: United States of America; FT: Feeding Time; Duration: follow-up duration in weeks; 2M/3M/6M: 2, 3 or 6 meals; 3M+2S: 3 meals and 2 snacks; 3M+3S: 3 meals and 3 snacks; 10S: Grazing diet – 100kcal every 2-3 hours; HCB: High Calorie Breakfast; HCD: High Calorie Dinner; ED: Early Dinner; LD: Late Dinner; FL: Front Loading: EED: Equal Energy Distribution; ML: Middle Loading; BL: Back Loading.

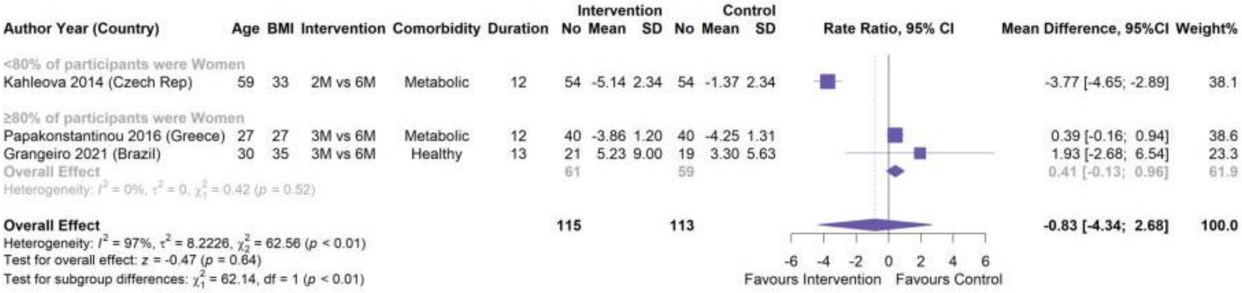

eFigure 113: Meta-analysis of difference in mean difference (95% CIs) for the effect of meal frequency on waist circumference (cm), grouped by gender proportion

BMI: Body Mass Index; SD: Standard Deviation; CI: Confidence Interval; USA: United States of America; FT: Feeding Time; Duration: follow-up duration in weeks; 2M/3M/6M: 2, 3 or 6 meals; 3M+2S: 3 meals and 2 snacks; 3M+3S: 3 meals and 3 snacks; 10S: Grazing diet – 100kcal every 2-3 hours; HCB: High Calorie Breakfast; HCD: High Calorie Dinner; ED: Early Dinner; LD: Late Dinner; FL: Front Loading: EED: Equal Energy Distribution; ML: Middle Loading; BL: Back Loading.

© 2024 Liu HY et al. *JAMA Network Open.*

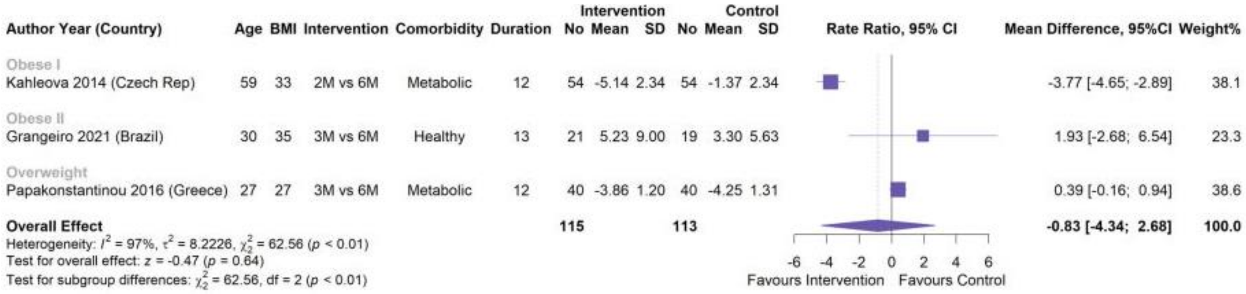

eFigure 114: Meta-analysis of difference in mean difference (95% CIs) for the effect of meal frequency on waist circumference (cm), grouped by baseline BMI status

BMI: Body Mass Index; SD: Standard Deviation; CI: Confidence Interval; USA: United States of America; FT: Feeding Time; Duration: follow-up duration in weeks; 2M/3M/6M: 2, 3 or 6 meals; 3M+2S: 3 meals and 2 snacks; 3M+3S: 3 meals and 3 snacks; 10S: Grazing diet – 100kcal every 2-3 hours; HCB: High Calorie Breakfast; HCD: High Calorie Dinner; ED: Early Dinner; LD: Late Dinner; FL: Front Loading: EED: Equal Energy Distribution; ML: Middle Loading; BL: Back Loading.

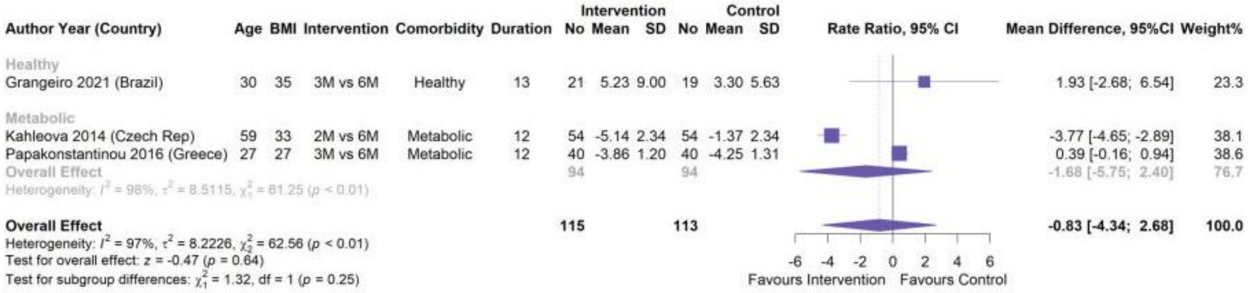

eFigure 115: Meta-analysis of difference in mean difference (95% CIs) for the effect of meal frequency on waist circumference (cm), grouped by health status

BMI: Body Mass Index; SD: Standard Deviation; CI: Confidence Interval; USA: United States of America; FT: Feeding Time; Duration: follow-up duration in weeks; 2M/3M/6M: 2, 3 or 6 meals; 3M+2S: 3 meals and 2 snacks; 3M+3S: 3 meals and 3 snacks; 10S: Grazing diet – 100kcal every 2-3 hours; HCB: High Calorie Breakfast; HCD: High Calorie Dinner; ED: Early Dinner; LD: Late Dinner; FL: Front Loading: EED: Equal Energy Distribution; ML: Middle Loading; BL: Back Loading.

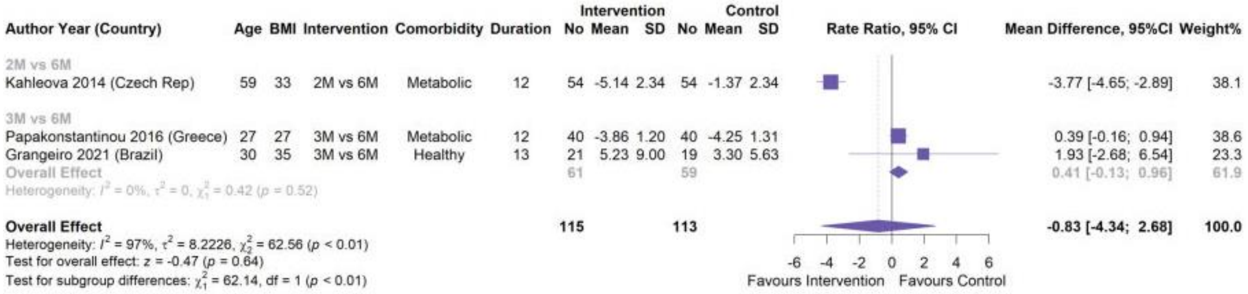

eFigure 116: Meta-analysis of difference in mean difference (95% CIs) for the effect of meal frequency on waist circumference (cm), grouped by intervention intensity

BMI: Body Mass Index; SD: Standard Deviation; CI: Confidence Interval; USA: United States of America; FT: Feeding Time; Duration: follow-up duration in weeks; 2M/3M/6M: 2, 3 or 6 meals; 3M+2S: 3 meals and 2 snacks; 3M+3S: 3 meals and 3 snacks; 10S: Grazing diet – 100kcal every 2-3 hours; HCB: High Calorie Breakfast; HCD: High Calorie Dinner; ED: Early Dinner; LD: Late Dinner; FL: Front Loading: EED: Equal Energy Distribution; ML: Middle Loading; BL: Back Loading.

© 2024 Liu HY et al. *JAMA Network Open.*

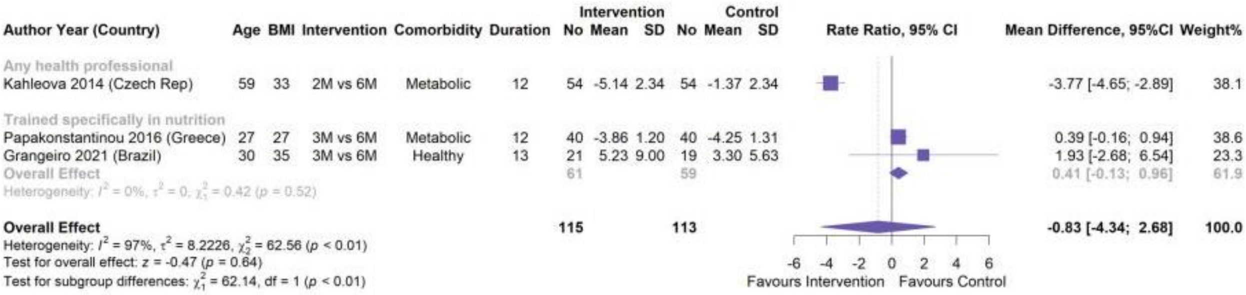

eFigure 117: Meta-analysis of difference in mean difference (95% CIs) for the effect of meal frequency on waist circumference (cm), grouped by delivery personnel

BMI: Body Mass Index; SD: Standard Deviation; CI: Confidence Interval; USA: United States of America; FT: Feeding Time; Duration: follow-up duration in weeks; 2M/3M/6M: 2, 3 or 6 meals; 3M+2S: 3 meals and 2 snacks; 3M+3S: 3 meals and 3 snacks; 10S: Grazing diet – 100kcal every 2-3 hours; HCB: High Calorie Breakfast; HCD: High Calorie Dinner; ED: Early Dinner; LD: Late Dinner; FL: Front Loading: EED: Equal Energy Distribution; ML: Middle Loading; BL: Back Loading.

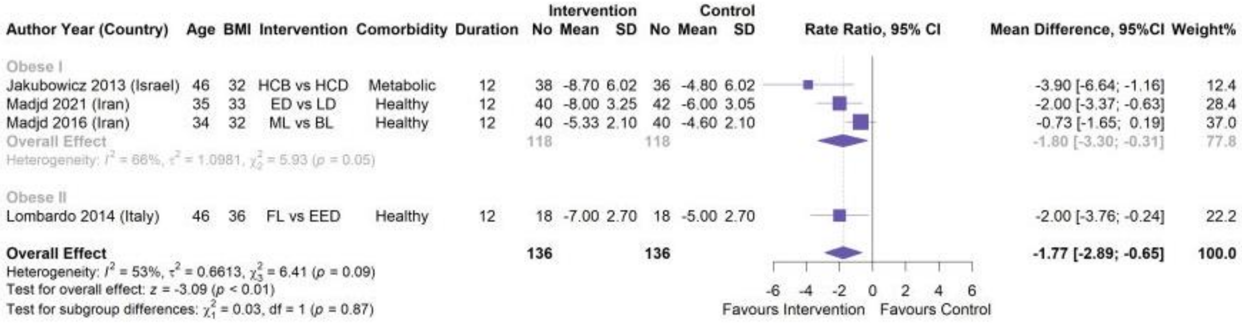

eFigure 118: Meta-analysis of difference in mean difference (95% CIs) for the effect of meal distribution on waist circumference (cm), grouped by baseline BMI status

BMI: Body Mass Index; SD: Standard Deviation; CI: Confidence Interval; USA: United States of America; FT: Feeding Time; Duration: follow-up duration in weeks; 2M/3M/6M: 2, 3 or 6 meals; 3M+2S: 3 meals and 2 snacks; 3M+3S: 3 meals and 3 snacks; 10S: Grazing diet – 100kcal every 2-3 hours; HCB: High Calorie Breakfast; HCD: High Calorie Dinner; ED: Early Dinner; LD: Late Dinner; FL: Front Loading: EED: Equal Energy Distribution; ML: Middle Loading; BL: Back Loading.

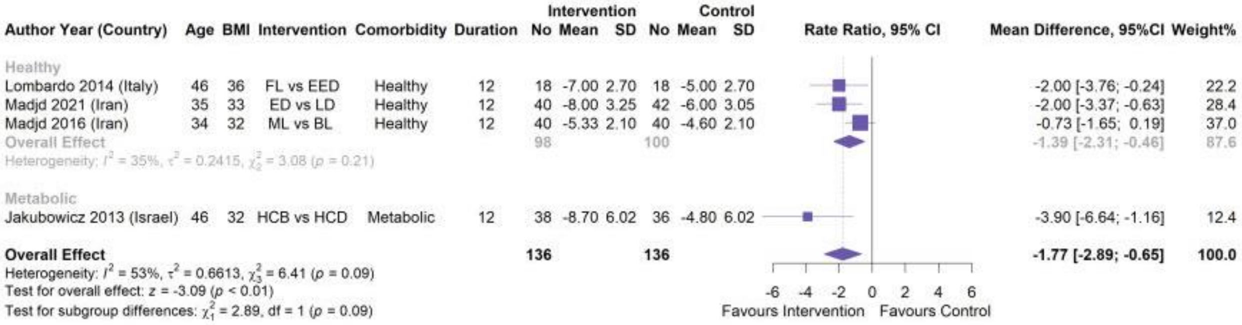

eFigure 119: Meta-analysis of difference in mean difference (95% CIs) for the effect of meal distribution on waist circumference (cm), grouped by health status

BMI: Body Mass Index; SD: Standard Deviation; CI: Confidence Interval; USA: United States of America; FT: Feeding Time; Duration: follow-up duration in weeks; 2M/3M/6M: 2, 3 or 6 meals; 3M+2S: 3 meals and 2 snacks; 3M+3S: 3 meals and 3 snacks; 10S: Grazing diet – 100kcal every 2-3 hours; HCB: High Calorie Breakfast; HCD: High Calorie Dinner; ED: Early Dinner; LD: Late Dinner; FL: Front Loading: EED: Equal Energy Distribution; ML: Middle Loading; BL: Back Loading.

© 2024 Liu HY et al. *JAMA Network Open.*

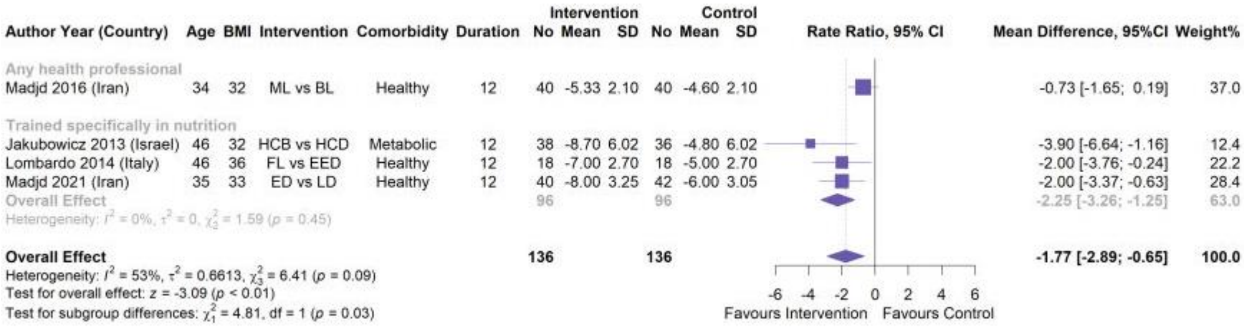

eFigure 120: Meta-analysis of difference in mean difference (95% CIs) for the effect of meal distribution on waist circumference (cm), grouped by delivery personnel

BMI: Body Mass Index; SD: Standard Deviation; CI: Confidence Interval; USA: United States of America; FT: Feeding Time; Duration: follow-up duration in weeks; 2M/3M/6M: 2, 3 or 6 meals; 3M+2S: 3 meals and 2 snacks; 3M+3S: 3 meals and 3 snacks; 10S: Grazing diet – 100kcal every 2-3 hours; HCB: High Calorie Breakfast; HCD: High Calorie Dinner; ED: Early Dinner; LD: Late Dinner; FL: Front Loading: EED: Equal Energy Distribution; ML: Middle Loading; BL: Back Loading.

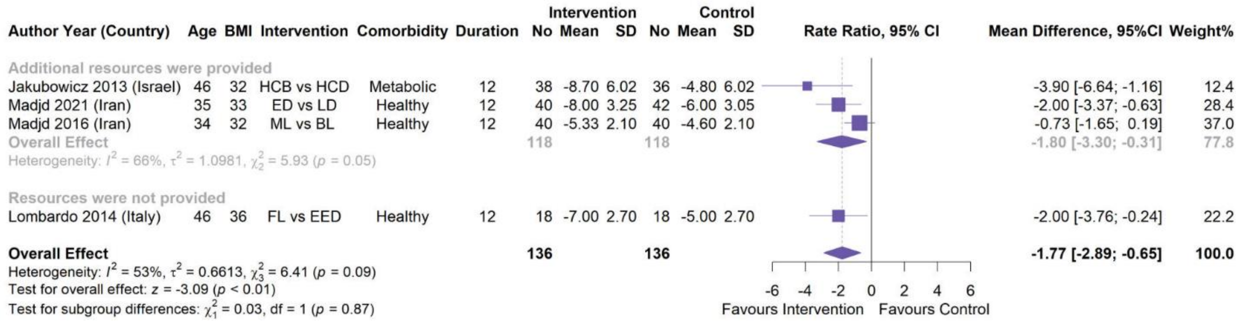

eFigure 121: Meta-analysis of difference in mean difference (95% CIs) for the effect of meal distribution on waist circumference (cm), grouped by resource provision

BMI: Body Mass Index; SD: Standard Deviation; CI: Confidence Interval; USA: United States of America; FT: Feeding Time; Duration: follow-up duration in weeks; 2M/3M/6M: 2, 3 or 6 meals; 3M+2S: 3 meals and 2 snacks; 3M+3S: 3 meals and 3 snacks; 10S: Grazing diet – 100kcal every 2-3 hours; HCB: High Calorie Breakfast; HCD: High Calorie Dinner; ED: Early Dinner; LD: Late Dinner; FL: Front Loading: EED: Equal Energy Distribution; ML: Middle Loading; BL: Back Loading.

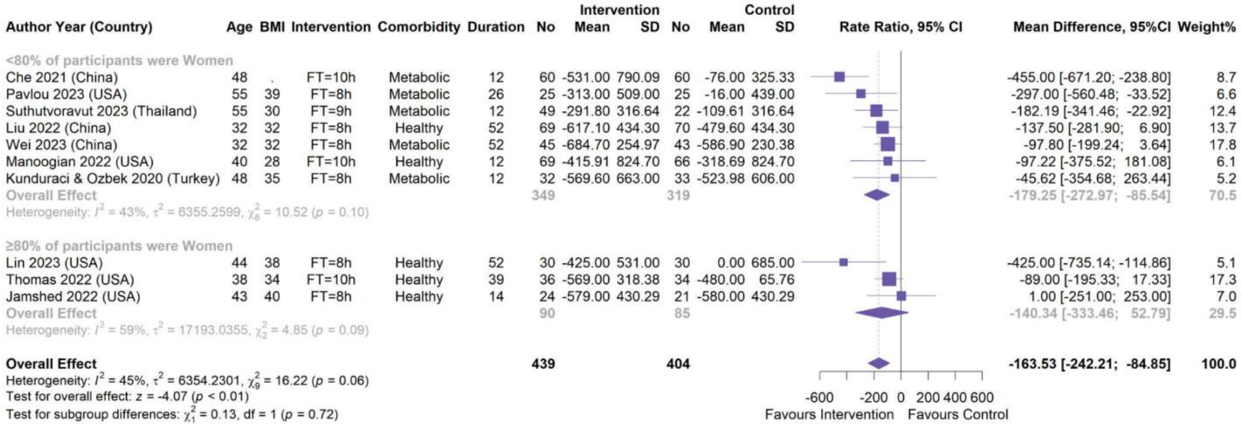

eFigure 122: Meta-analysis of difference in mean difference (95% CIs) for the effect of time-restricted eating on energy intake (kcal/day), grouped by gender proportion

BMI: Body Mass Index; SD: Standard Deviation; CI: Confidence Interval; USA: United States of America; FT: Feeding Time; Duration: follow-up duration in weeks; 2M/3M/6M: 2, 3 or 6 meals; 3M+2S: 3 meals and 2 snacks; 3M+3S: 3 meals and 3 snacks; 10S: Grazing diet

© 2024 Liu HY et al. *JAMA Network Open.*

– 100kcal every 2-3 hours; HCB: High Calorie Breakfast; HCD: High Calorie Dinner; ED: Early Dinner; LD: Late Dinner; FL: Front Loading: EED: Equal Energy Distribution; ML: Middle Loading; BL: Back Loading.

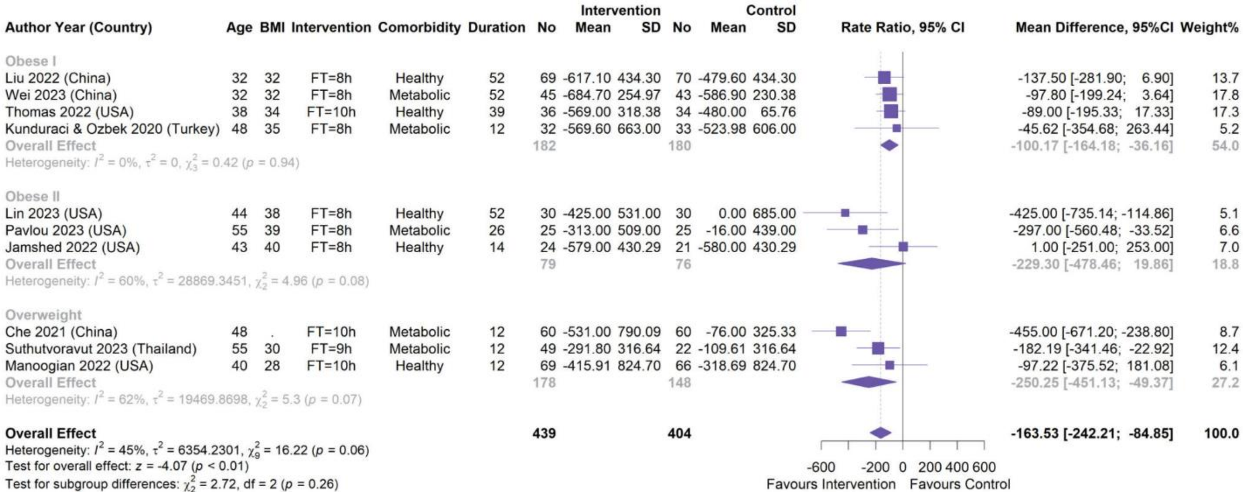

eFigure 123: Meta-analysis of difference in mean difference (95% CIs) for the effect of time-restricted eating on energy intake (kcal/day), grouped by baseline BMI status

BMI: Body Mass Index; SD: Standard Deviation; CI: Confidence Interval; USA: United States of America; FT: Feeding Time; Duration: follow-up duration in weeks; 2M/3M/6M: 2, 3 or 6 meals; 3M+2S: 3 meals and 2 snacks; 3M+3S: 3 meals and 3 snacks; 10S: Grazing diet – 100kcal every 2-3 hours; HCB: High Calorie Breakfast; HCD: High Calorie Dinner; ED: Early Dinner; LD: Late Dinner; FL: Front Loading: EED: Equal Energy Distribution; ML: Middle Loading; BL: Back Loading.

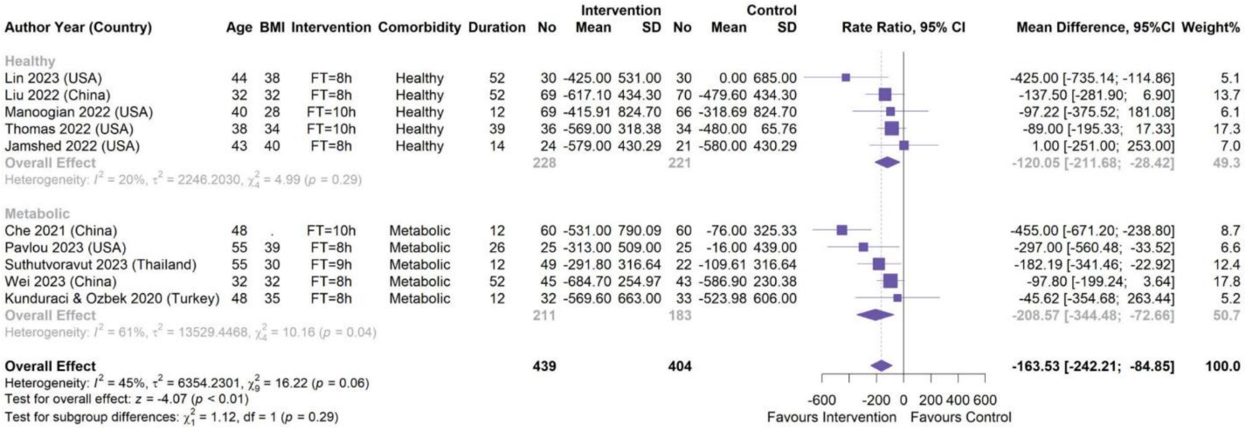

eFigure 124: Meta-analysis of difference in mean difference (95% CIs) for the effect of time-restricted eating on energy intake (kcal/day), grouped by health status

BMI: Body Mass Index; SD: Standard Deviation; CI: Confidence Interval; USA: United States of America; FT: Feeding Time; Duration: follow-up duration in weeks; 2M/3M/6M: 2, 3 or 6 meals; 3M+2S: 3 meals and 2 snacks; 3M+3S: 3 meals and 3 snacks; 10S: Grazing diet – 100kcal every 2-3 hours; HCB: High Calorie Breakfast; HCD: High Calorie Dinner; ED: Early Dinner; LD: Late Dinner; FL: Front Loading: EED: Equal Energy Distribution; ML: Middle Loading; BL: Back Loading.

© 2024 Liu HY et al. *JAMA Network Open.*

Author Year (Country)	Age	BMI	Intervention	Comorbidity	Duration	Intervention No	Intervention Mean	Intervention SD	Control No	Control Mean	Control SD	Mean Difference, 95%CI	Weight%
Follow duration <6 months													
Che 2021 (China)	48	.	FT=10h	Metabolic	12	60	-531.00	790.09	60	-76.00	325.33	-455.00 [-671.20; -238.80]	8.7
Suthutvoravut 2023 (Thailand)	55	30	FT=9h	Metabolic	12	49	-291.80	316.64	22	-109.61	316.64	-182.19 [-341.46; -22.92]	12.4
Manoogian 2022 (USA)	40	28	FT=10h	Healthy	12	69	-415.91	824.70	66	-318.69	824.70	-97.22 [-375.52; 181.08]	6.1
Kunduraci & Ozbek 2020 (Turkey)	48	35	FT=8h	Metabolic	12	32	-569.60	663.00	33	-523.98	606.00	-45.62 [-354.68; 263.44]	5.2
Jamshed 2022 (USA)	43	40	FT=8h	Healthy	14	24	-579.00	430.29	21	-580.00	430.29	1.00 [-251.00; 253.00]	7.0
Overall Effect						234			202			-171.05 [-330.68; -11.43]	39.4
Heterogeneity: I^2 = 57%, τ^2 = 18319.9581, χ^2_4 = 9.25 (p = 0.06)													
Follow duration ≥6 months													
Lin 2023 (USA)	44	38	FT=8h	Healthy	52	30	-425.00	531.00	30	0.00	685.00	-425.00 [-735.14; -114.86]	5.1
Pavlou 2023 (USA)	55	39	FT=8h	Metabolic	26	25	-313.00	509.00	25	-16.00	439.00	-297.00 [-560.48; -33.52]	6.6
Liu 2022 (China)	32	32	FT=8h	Healthy	52	69	-617.10	434.30	70	-479.60	434.30	-137.50 [-281.90; 6.90]	13.7
Wei 2023 (China)	32	32	FT=8h	Metabolic	52	45	-684.70	254.97	43	-586.90	230.38	-97.80 [-199.24; 3.64]	17.8
Thomas 2022 (USA)	38	34	FT=10h	Healthy	39	36	-569.00	318.38	34	-480.00	65.76	-89.00 [-195.33; 17.33]	17.3
Overall Effect						205			202			-141.69 [-224.57; -58.82]	60.6
Heterogeneity: I^2 = 33%, τ^2 = 2833.0808, χ^2_4 = 5.97 (p = 0.20)													
**Overall Effect**						**439**			**404**			**-163.53 [-242.21; -84.85]**	**100.0**
Heterogeneity: I^2 = 45%, τ^2 = 6354.2301, χ^2_9 = 16.22 (p = 0.06)													
Test for overall effect: z = -4.07 (p < 0.01)													
Test for subgroup differences: χ^2_1 = 0.10, df = 1 (p = 0.75)													

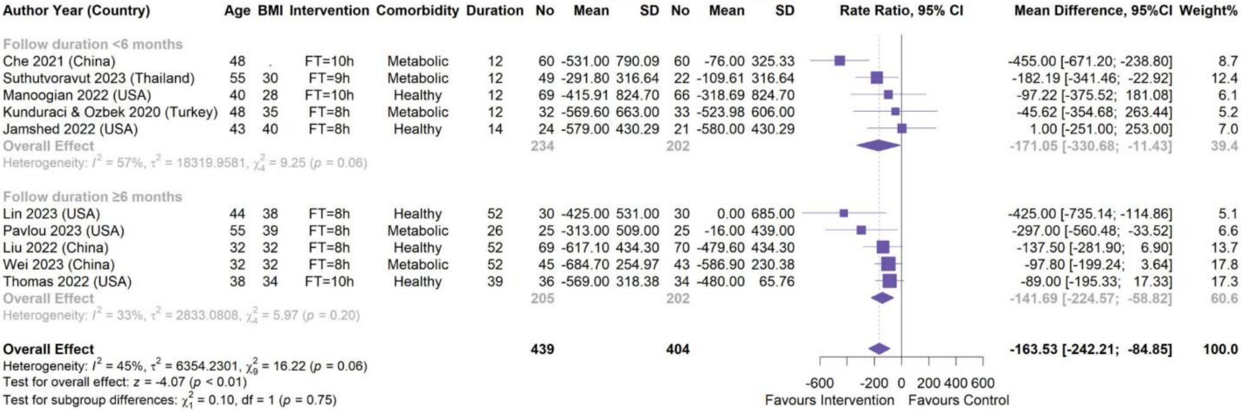

eFigure 125: Meta-analysis of difference in mean difference (95% CIs) for the effect of time-restricted eating on energy intake (kcal/day), grouped by follow duration

BMI: Body Mass Index; SD: Standard Deviation; CI: Confidence Interval; USA: United States of America; FT: Feeding Time; Duration: follow-up duration in weeks; 2M/3M/6M: 2, 3 or 6 meals; 3M+2S: 3 meals and 2 snacks; 3M+3S: 3 meals and 3 snacks; 10S: Grazing diet – 100kcal every 2-3 hours; HCB: High Calorie Breakfast; HCD: High Calorie Dinner; ED: Early Dinner; LD: Late Dinner; FL: Front Loading: EED: Equal Energy Distribution; ML: Middle Loading; BL: Back Loading.

Author Year (Country)	Age	BMI	Intervention	Comorbidity	Duration	Intervention No	Intervention Mean	Intervention SD	Control No	Control Mean	Control SD	Mean Difference, 95%CI	Weight%
Feeding time is > 8 hours													
Che 2021 (China)	48	.	FT=10h	Metabolic	12	60	-531.00	790.09	60	-76.00	325.33	-455.00 [-671.20; -238.80]	8.7
Suthutvoravut 2023 (Thailand)	55	30	FT=9h	Metabolic	12	49	-291.80	316.64	22	-109.61	316.64	-182.19 [-341.46; -22.92]	12.4
Manoogian 2022 (USA)	40	28	FT=10h	Healthy	12	69	-415.91	824.70	66	-318.69	824.70	-97.22 [-375.52; 181.08]	6.1
Thomas 2022 (USA)	38	34	FT=10h	Healthy	39	36	-569.00	318.38	34	-480.00	65.76	-89.00 [-195.33; 17.33]	17.3
Overall Effect						214			182			-197.21 [-351.71; -42.72]	44.5
Heterogeneity: I^2 = 67%, τ^2 = 15889.4446, χ^2_3 = 9.13 (p = 0.03)													
Feeding time is ≤ 8 hours													
Lin 2023 (USA)	44	38	FT=8h	Healthy	52	30	-425.00	531.00	30	0.00	685.00	-425.00 [-735.14; -114.86]	5.1
Pavlou 2023 (USA)	55	39	FT=8h	Metabolic	26	25	-313.00	509.00	25	-16.00	439.00	-297.00 [-560.48; -33.52]	6.6
Liu 2022 (China)	32	32	FT=8h	Healthy	52	69	-617.10	434.30	70	-479.60	434.30	-137.50 [-281.90; 6.90]	13.7
Wei 2023 (China)	32	32	FT=8h	Metabolic	52	45	-684.70	254.97	43	-586.90	230.38	-97.80 [-199.24; 3.64]	17.8
Kunduraci & Ozbek 2020 (Turkey)	48	35	FT=8h	Metabolic	12	32	-569.60	663.00	33	-523.98	606.00	-45.62 [-354.68; 263.44]	5.2
Jamshed 2022 (USA)	43	40	FT=8h	Healthy	14	24	-579.00	430.29	21	-580.00	430.29	1.00 [-251.00; 253.00]	7.0
Overall Effect						225			222			-139.33 [-232.28; -46.39]	55.5
Heterogeneity: I^2 = 26%, τ^2 = 3408.3091, χ^2_5 = 6.74 (p = 0.24)													
**Overall Effect**						**439**			**404**			**-163.53 [-242.21; -84.85]**	**100.0**
Heterogeneity: I^2 = 45%, τ^2 = 6354.2301, χ^2_9 = 16.22 (p = 0.06)													
Test for overall effect: z = -4.07 (p < 0.01)													
Test for subgroup differences: χ^2_1 = 0.40, df = 1 (p = 0.53)													

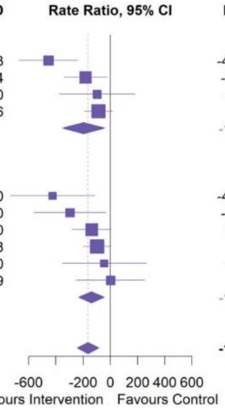

eFigure 126: Meta-analysis of difference in mean difference (95% CIs) for the effect of time-restricted eating on energy intake (kcal/day), grouped by eating window

BMI: Body Mass Index; SD: Standard Deviation; CI: Confidence Interval; USA: United States of America; FT: Feeding Time; Duration: follow-up duration in weeks; 2M/3M/6M: 2, 3 or 6 meals; 3M+2S: 3 meals and 2 snacks; 3M+3S: 3 meals and 3 snacks; 10S: Grazing diet – 100kcal every 2-3 hours; HCB: High Calorie Breakfast; HCD: High Calorie Dinner; ED: Early Dinner; LD: Late Dinner; FL: Front Loading: EED: Equal Energy Distribution; ML: Middle Loading; BL: Back Loading.

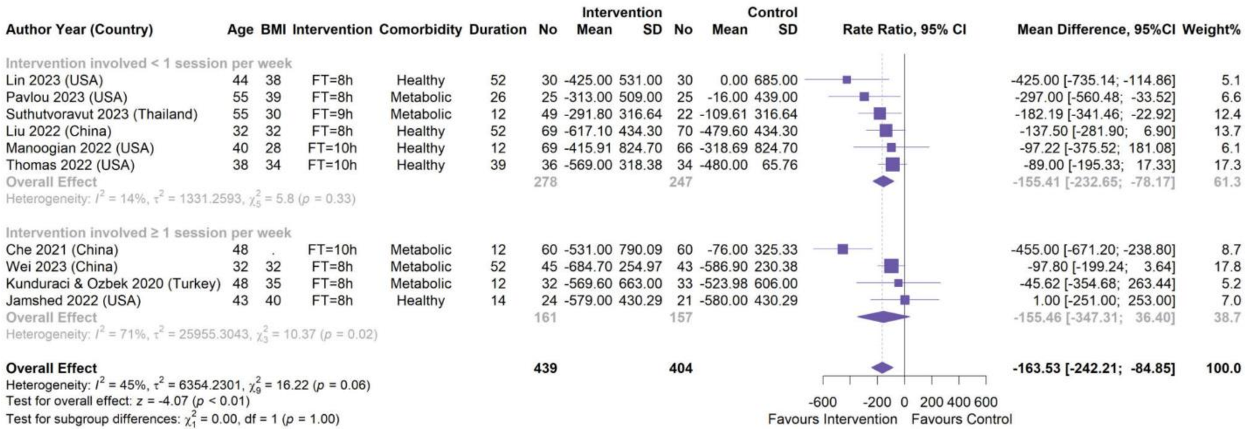

eFigure 127: Meta-analysis of difference in mean difference (95% CIs) for the effect of time-restricted eating on energy intake (kcal/day), grouped by frequency of contact

BMI: Body Mass Index; SD: Standard Deviation; CI: Confidence Interval; USA: United States of America; FT: Feeding Time; Duration: follow-up duration in weeks; 2M/3M/6M: 2, 3 or 6 meals; 3M+2S: 3 meals and 2 snacks; 3M+3S: 3 meals and 3 snacks; 10S: Grazing diet – 100kcal every 2-3 hours; HCB: High Calorie Breakfast; HCD: High Calorie Dinner; ED: Early Dinner; LD: Late Dinner; FL: Front Loading: EED: Equal Energy Distribution; ML: Middle Loading; BL: Back Loading.

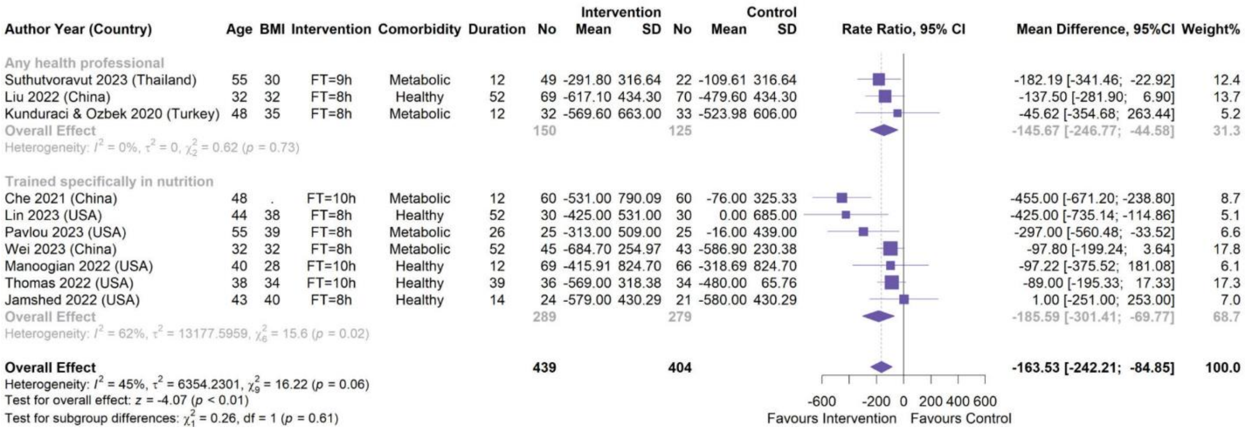

eFigure 128: Meta-analysis of difference in mean difference (95% CIs) for the effect of time-restricted eating on energy intake (kcal/day), grouped by delivery personnel

BMI: Body Mass Index; SD: Standard Deviation; CI: Confidence Interval; USA: United States of America; FT: Feeding Time; Duration: follow-up duration in weeks; 2M/3M/6M: 2, 3 or 6 meals; 3M+2S: 3 meals and 2 snacks; 3M+3S: 3 meals and 3 snacks; 10S: Grazing diet – 100kcal every 2-3 hours; HCB: High Calorie Breakfast; HCD: High Calorie Dinner; ED: Early Dinner; LD: Late Dinner; FL: Front Loading: EED: Equal Energy Distribution; ML: Middle Loading; BL: Back Loading.

© 2024 Liu HY et al. *JAMA Network Open.*

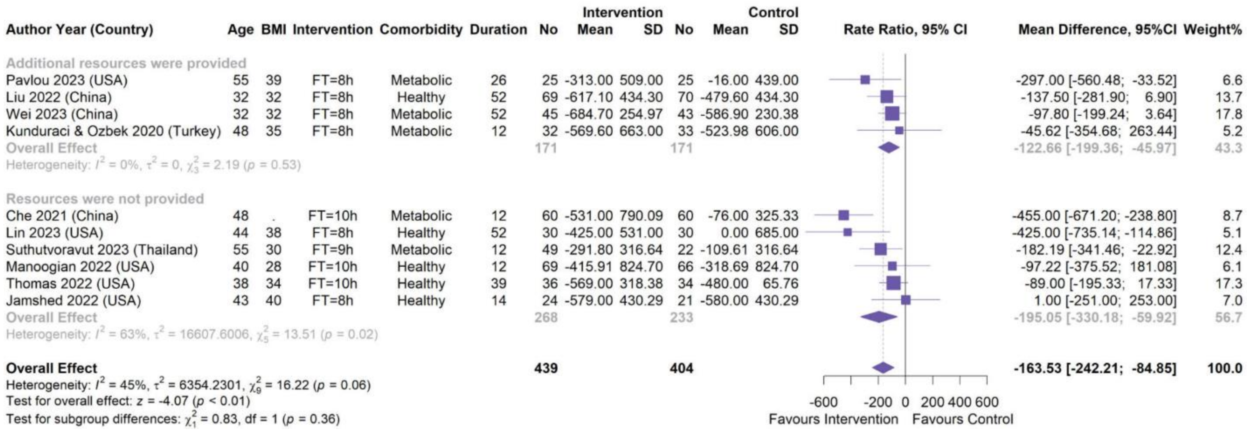

eFigure 129: Meta-analysis of difference in mean difference (95% CIs) for the effect of time-restricted eating on energy intake (kcal/day), grouped by resource provision

BMI: Body Mass Index; SD: Standard Deviation; CI: Confidence Interval; USA: United States of America; FT: Feeding Time; Duration: follow-up duration in weeks; 2M/3M/6M: 2, 3 or 6 meals; 3M+2S: 3 meals and 2 snacks; 3M+3S: 3 meals and 3 snacks; 10S: Grazing diet – 100kcal every 2-3 hours; HCB: High Calorie Breakfast; HCD: High Calorie Dinner; ED: Early Dinner; LD: Late Dinner; FL: Front Loading: EED: Equal Energy Distribution; ML: Middle Loading; BL: Back Loading.

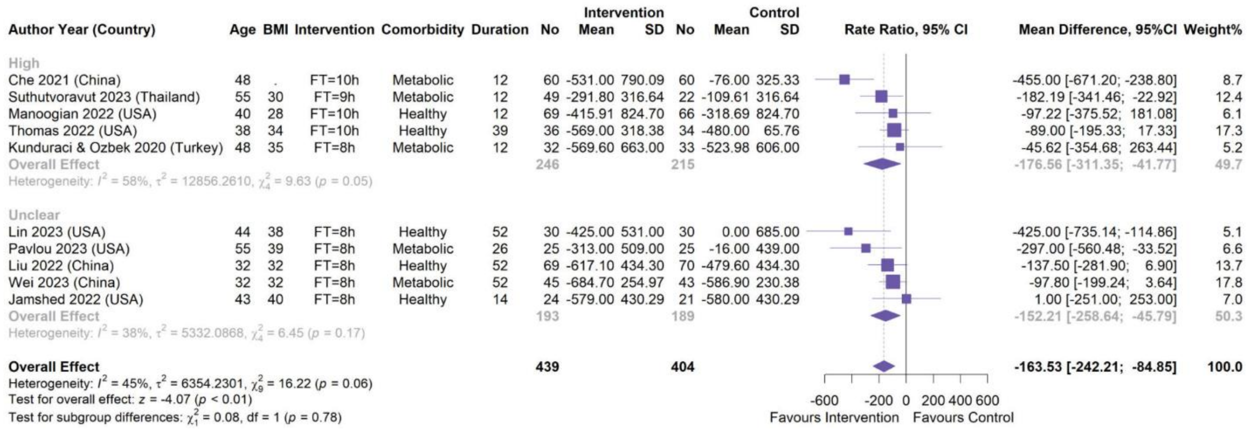

eFigure 130: Meta-analysis of difference in mean difference (95% CIs) for the effect of time-restricted eating on energy intake (kcal/day), grouped by risk of bias

BMI: Body Mass Index; SD: Standard Deviation; CI: Confidence Interval; USA: United States of America; FT: Feeding Time; Duration: follow-up duration in weeks; 2M/3M/6M: 2, 3 or 6 meals; 3M+2S: 3 meals and 2 snacks; 3M+3S: 3 meals and 3 snacks; 10S: Grazing diet – 100kcal every 2-3 hours; HCB: High Calorie Breakfast; HCD: High Calorie Dinner; ED: Early Dinner; LD: Late Dinner; FL: Front Loading: EED: Equal Energy Distribution; ML: Middle Loading; BL: Back Loading.

© 2024 Liu HY et al. *JAMA Network Open.*

Author Year (Country)	Age	BMI	Intervention	Comorbidity	Duration	Intervention No	Intervention Mean	Intervention SD	Control No	Control Mean	Control SD	Mean Difference, 95%CI	Weight%
Obese I													
Wei 2023 (China)	32	32	FT=8h	Metabolic	52	45	-0.20	0.48	43	-0.10	0.48	-0.10 [-0.30; 0.10]	6.4
Montero 2023 (Spain)	48	.	FT=8h	Metabolic	12	148	0.00	0.25	49	0.05	0.32	-0.05 [-0.15; 0.05]	10.0
Lowe 2020 (USA)	46	33	FT=8h	Healthy	12	22	-0.02	0.18	24	-0.01	0.18	-0.02 [-0.12; 0.08]	9.9
Kunduraci & Ozbek 2020 (Turkey)	48	35	FT=8h	Metabolic	12	32	-0.32	0.07	33	-0.31	0.18	-0.01 [-0.08; 0.06]	11.2
Chow 2020 (USA)	46	34	FT=8h	Healthy	12	11	-0.00	0.01	9	0.00	0.01	-0.00 [-0.01; 0.00]	12.3
Thomas 2022 (USA)	38	34	FT=10h	Healthy	39	18	0.00	0.20	20	0.00	0.30	0.00 [-0.16; 0.16]	7.7
Overall Effect						276			178			-0.00 [-0.01; 0.00]	57.5
Heterogeneity: $I^2 = 0\%$, $\tau^2 = 0$, $\chi^2_5 = 1.97$ ($p = 0.85$)													
Obese II													
Pavlou 2023 (USA)	55	39	FT=8h	Metabolic	26	25	-0.72	1.26	25	0.19	1.26	-0.91 [-1.61; -0.21]	1.0
Lin 2023 (USA)	44	38	FT=8h	Healthy	52	30	-0.01	0.34	30	0.07	0.34	-0.08 [-0.25; 0.09]	7.3
Jamshed 2022 (USA)	43	40	FT=8h	Healthy	14	45	-0.10	0.48	45	-0.10	0.48	0.00 [-0.20; 0.20]	6.4
Overall Effect						100			100			-0.14 [-0.41; 0.12]	14.7
Heterogeneity: $I^2 = 67\%$, $\tau^2 = 0.0329$, $\chi^2_2 = 6.01$ ($p = 0.05$)													
Overweight													
Che 2021 (China)	48	.	FT=10h	Metabolic	12	60	-1.54	1.47	60	-0.66	1.24	-0.88 [-1.37; -0.39]	1.9
Manoogian 2022 (USA)	40	28	FT=10h	Healthy	12	70	-0.04	0.27	66	0.27	0.24	-0.31 [-0.39; -0.22]	10.5
Suthutvoravut 2023 (Thailand)	55	30	FT=9h	Metabolic	12	49	-0.89	0.43	22	-0.65	0.43	-0.24 [-0.46; -0.02]	5.9
Philips 2021 (Switzerland)	40	28	FT=12h	Metabolic	26	23	0.03	0.18	18	-0.09	0.18	0.12 [0.01; 0.23]	9.6
Overall Effect						202			166			-0.26 [-0.56; 0.04]	27.9
Heterogeneity: $I^2 = 93\%$, $\tau^2 = 0.0793$, $\chi^2_3 = 45.1$ ($p < 0.01$)													
**Overall Effect**						**578**			**444**			**-0.08 [-0.15; -0.01]**	**100.0**

Heterogeneity: $I^2 = 85\%$, $\tau^2 = 0.0112$, $\chi^2_{12} = 79.12$ ($p < 0.01$)
Test for overall effect: $z = -2.18$ ($p = 0.03$)
Test for subgroup differences: $\chi^2_2 = 3.95$, df = 2 ($p = 0.14$)

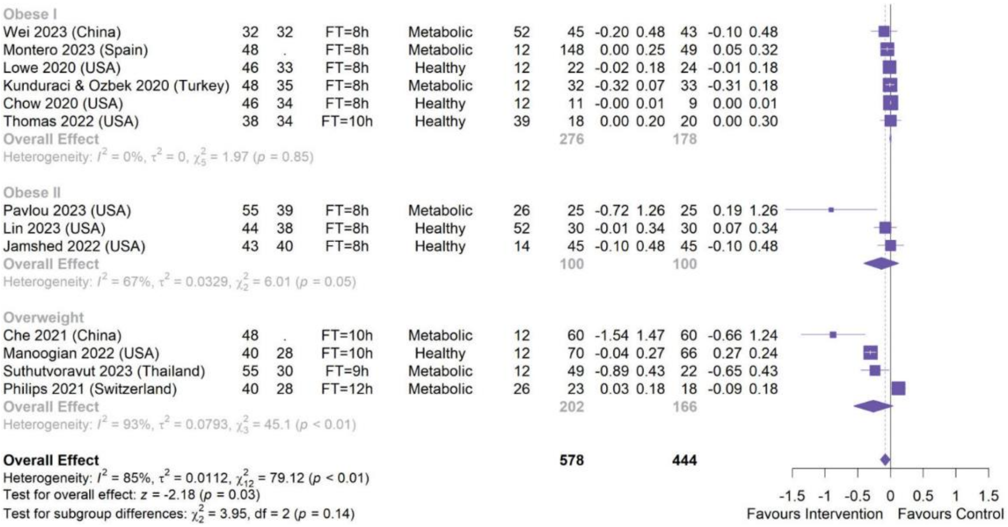

eFigure 131: Meta-analysis of difference in mean difference (95% CIs) for the effect of time-restricted eating on HbA1c (%), grouped by baseline BMI status

BMI: Body Mass Index; SD: Standard Deviation; CI: Confidence Interval; USA: United States of America; FT: Feeding Time; Duration: follow-up duration in weeks; 2M/3M/6M: 2, 3 or 6 meals; 3M+2S: 3 meals and 2 snacks; 3M+3S: 3 meals and 3 snacks; 10S: Grazing diet – 100kcal every 2-3 hours; HCB: High Calorie Breakfast; HCD: High Calorie Dinner; ED: Early Dinner; LD: Late Dinner; FL: Front Loading: EED: Equal Energy Distribution; ML: Middle Loading; BL: Back Loading.

Author Year (Country)	Age	BMI	Intervention	Comorbidity	Duration	Intervention No	Intervention Mean	Intervention SD	Control No	Control Mean	Control SD	Mean Difference, 95%CI	Weight%
Healthy													
Manoogian 2022 (USA)	40	28	FT=10h	Healthy	12	70	-0.04	0.27	66	0.27	0.24	-0.31 [-0.39; -0.22]	10.5
Lin 2023 (USA)	44	38	FT=8h	Healthy	52	30	-0.01	0.34	30	0.07	0.34	-0.08 [-0.25; 0.09]	7.3
Lowe 2020 (USA)	46	33	FT=8h	Healthy	12	22	-0.02	0.18	24	-0.01	0.18	-0.02 [-0.12; 0.08]	9.9
Chow 2020 (USA)	46	34	FT=8h	Healthy	12	11	-0.00	0.01	9	0.00	0.01	-0.00 [-0.01; 0.00]	12.3
Jamshed 2022 (USA)	43	40	FT=8h	Healthy	14	45	-0.10	0.48	45	-0.10	0.48	0.00 [-0.20; 0.20]	6.4
Thomas 2022 (USA)	38	34	FT=10h	Healthy	39	18	0.00	0.20	20	0.00	0.30	0.00 [-0.16; 0.16]	7.7
Overall Effect						196			194			-0.07 [-0.19; 0.05]	54.0
Heterogeneity: $I^2 = 90\%$, $\tau^2 = 0.0176$, $\chi^2_5 = 49.07$ ($p < 0.01$)													
Metabolic													
Pavlou 2023 (USA)	55	39	FT=8h	Metabolic	26	25	-0.72	1.26	25	0.19	1.26	-0.91 [-1.61; -0.21]	1.0
Che 2021 (China)	48	.	FT=10h	Metabolic	12	60	-1.54	1.47	60	-0.66	1.24	-0.88 [-1.37; -0.39]	1.9
Suthutvoravut 2023 (Thailand)	55	30	FT=9h	Metabolic	12	49	-0.89	0.43	22	-0.65	0.43	-0.24 [-0.46; -0.02]	5.9
Wei 2023 (China)	32	32	FT=8h	Metabolic	52	45	-0.20	0.48	43	-0.10	0.48	-0.10 [-0.30; 0.10]	6.4
Montero 2023 (Spain)	48	.	FT=8h	Metabolic	12	148	0.00	0.25	49	0.05	0.32	-0.05 [-0.15; 0.05]	10.0
Kunduraci & Ozbek 2020 (Turkey)	48	35	FT=8h	Metabolic	12	32	-0.32	0.07	33	-0.31	0.18	-0.01 [-0.08; 0.06]	11.2
Philips 2021 (Switzerland)	40	28	FT=12h	Metabolic	26	23	0.03	0.18	18	-0.09	0.18	0.12 [0.01; 0.23]	9.6
Overall Effect						382			250			-0.11 [-0.24; 0.02]	46.0
Heterogeneity: $I^2 = 80\%$, $\tau^2 = 0.0193$, $\chi^2_6 = 29.42$ ($p < 0.01$)													
**Overall Effect**						**578**			**444**			**-0.08 [-0.15; -0.01]**	**100.0**

Heterogeneity: $I^2 = 85\%$, $\tau^2 = 0.0112$, $\chi^2_{12} = 79.12$ ($p < 0.01$)
Test for overall effect: $z = -2.18$ ($p = 0.03$)
Test for subgroup differences: $\chi^2_1 = 0.20$, df = 1 ($p = 0.65$)

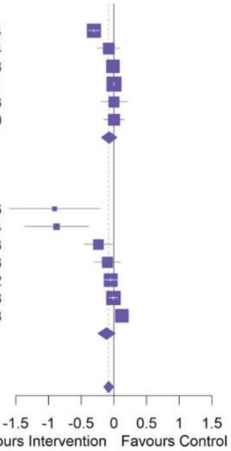

eFigure 132: Meta-analysis of difference in mean difference (95% CIs) for the effect of time-restricted eating on HbA1c (%), grouped by health status

BMI: Body Mass Index; SD: Standard Deviation; CI: Confidence Interval; USA: United States of America; FT: Feeding Time; Duration: follow-up duration in weeks; 2M/3M/6M: 2, 3 or 6 meals; 3M+2S: 3 meals and 2 snacks; 3M+3S: 3 meals and 3 snacks; 10S: Grazing diet – 100kcal every 2-3 hours; HCB: High Calorie Breakfast; HCD: High Calorie Dinner; ED: Early Dinner; LD: Late Dinner; FL: Front Loading: EED: Equal Energy Distribution; ML: Middle Loading; BL: Back Loading.

© 2024 Liu HY et al. *JAMA Network Open.*

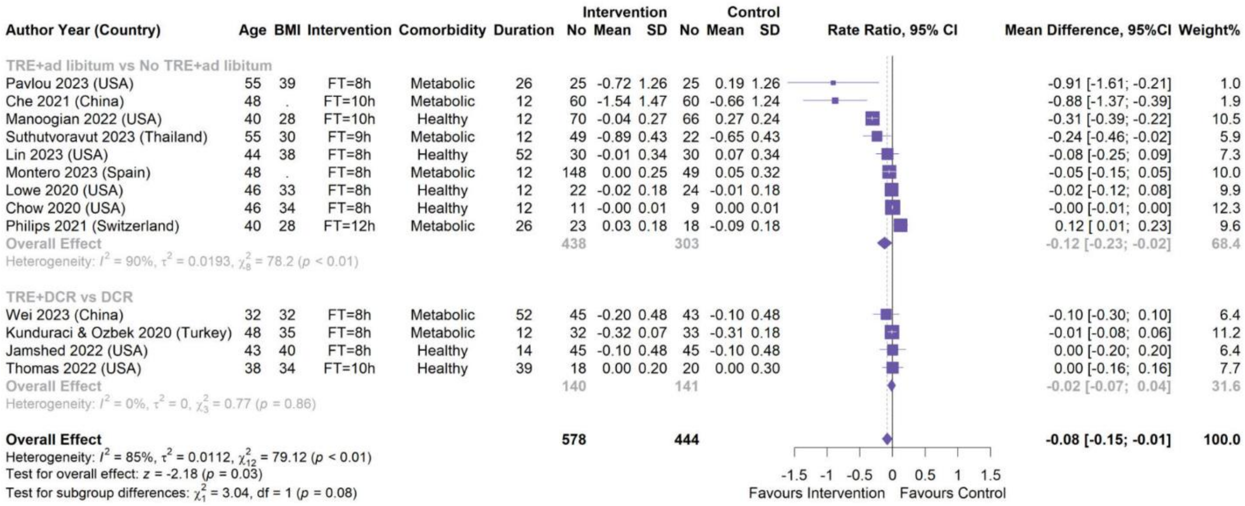

Author Year (Country)	Age	BMI	Intervention	Comorbidity	Duration	Intervention No	Intervention Mean	Intervention SD	Control No	Control Mean	Control SD	Mean Difference, 95%CI	Weight%
**TRE+ad libitum vs No TRE+ad libitum**													
Pavlou 2023 (USA)	55	39	FT=8h	Metabolic	26	25	-0.72	1.26	25	0.19	1.26	-0.91 [-1.61; -0.21]	1.0
Che 2021 (China)	48	.	FT=10h	Metabolic	12	60	-1.54	1.47	60	-0.66	1.24	-0.88 [-1.37; -0.39]	1.9
Manoogian 2022 (USA)	40	28	FT=10h	Healthy	12	70	-0.04	0.27	66	0.27	0.24	-0.31 [-0.39; -0.22]	10.5
Suthutvoravut 2023 (Thailand)	55	30	FT=9h	Metabolic	12	49	-0.89	0.43	22	-0.65	0.43	-0.24 [-0.46; -0.02]	5.9
Lin 2023 (USA)	44	38	FT=8h	Healthy	52	30	-0.01	0.34	30	0.07	0.34	-0.08 [-0.25; 0.09]	7.3
Montero 2023 (Spain)	48	.	FT=8h	Metabolic	12	148	0.00	0.25	49	0.05	0.32	-0.05 [-0.15; 0.05]	10.0
Lowe 2020 (USA)	46	33	FT=8h	Healthy	12	22	-0.02	0.18	24	-0.01	0.18	-0.02 [-0.12; 0.08]	9.9
Chow 2020 (USA)	46	34	FT=8h	Healthy	12	11	-0.00	0.01	9	0.00	0.01	-0.00 [-0.01; 0.00]	12.3
Philips 2021 (Switzerland)	40	28	FT=12h	Metabolic	26	23	0.03	0.18	18	-0.09	0.18	0.12 [0.01; 0.23]	9.6
Overall Effect						438			303			-0.12 [-0.23; -0.02]	68.4
Heterogeneity: $I^2 = 90\%$, $\tau^2 = 0.0193$, $\chi^2_8 = 78.2$ ($p < 0.01$)													
**TRE+DCR vs DCR**													
Wei 2023 (China)	32	32	FT=8h	Metabolic	52	45	-0.20	0.48	43	-0.10	0.48	-0.10 [-0.30; 0.10]	6.4
Kunduraci & Ozbek 2020 (Turkey)	48	35	FT=8h	Metabolic	12	32	-0.32	0.07	33	-0.31	0.18	-0.01 [-0.08; 0.06]	11.2
Jamshed 2022 (USA)	43	40	FT=8h	Healthy	14	45	-0.10	0.48	45	-0.10	0.48	0.00 [-0.20; 0.20]	6.4
Thomas 2022 (USA)	38	34	FT=10h	Healthy	39	18	0.00	0.20	20	0.00	0.30	0.00 [-0.16; 0.16]	7.7
Overall Effect						140			141			-0.02 [-0.07; 0.04]	31.6
Heterogeneity: $I^2 = 0\%$, $\tau^2 = 0$, $\chi^2_3 = 0.77$ ($p = 0.86$)													
**Overall Effect**						**578**			**444**			**-0.08 [-0.15; -0.01]**	**100.0**

Heterogeneity: $I^2 = 85\%$, $\tau^2 = 0.0112$, $\chi^2_{12} = 79.12$ ($p < 0.01$)
Test for overall effect: $z = -2.18$ ($p = 0.03$)
Test for subgroup differences: $\chi^2_1 = 3.04$, df = 1 ($p = 0.08$)

eFigure 133: Meta-analysis of difference in mean difference (95% CIs) for the effect of time-restricted eating on HbA1c (%), grouped by energy prescription

BMI: Body Mass Index; SD: Standard Deviation; CI: Confidence Interval; USA: United States of America; FT: Feeding Time; Duration: follow-up duration in weeks; 2M/3M/6M: 2, 3 or 6 meals; 3M+2S: 3 meals and 2 snacks; 3M+3S: 3 meals and 3 snacks; 10S: Grazing diet – 100kcal every 2-3 hours; HCB: High Calorie Breakfast; HCD: High Calorie Dinner; ED: Early Dinner; LD: Late Dinner; FL: Front Loading: EED: Equal Energy Distribution; ML: Middle Loading; BL: Back Loading.

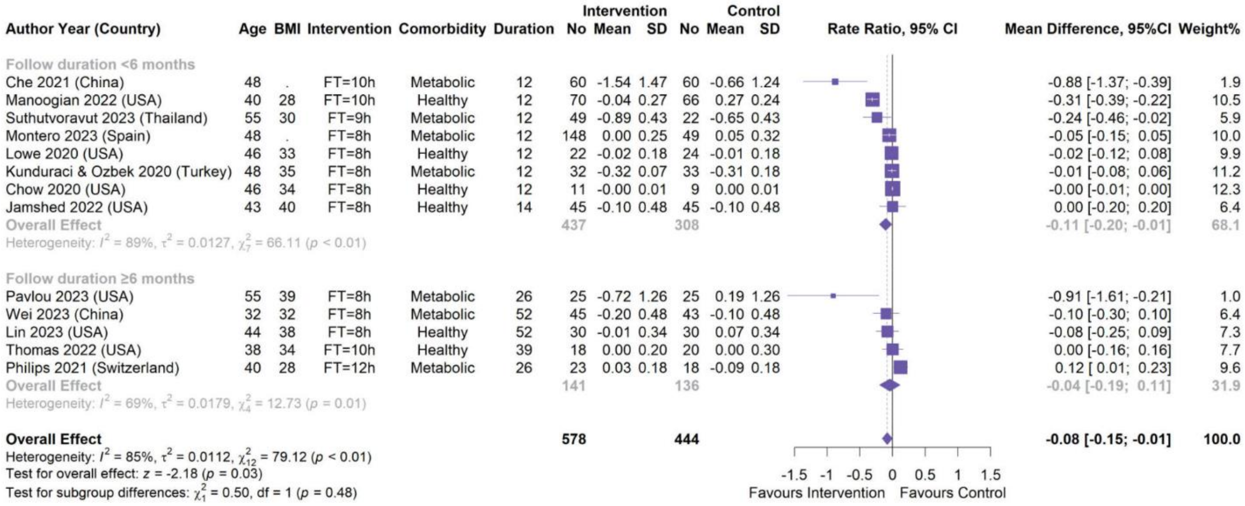

Author Year (Country)	Age	BMI	Intervention	Comorbidity	Duration	Intervention No	Intervention Mean	Intervention SD	Control No	Control Mean	Control SD	Mean Difference, 95%CI	Weight%
**Follow duration <6 months**													
Che 2021 (China)	48	.	FT=10h	Metabolic	12	60	-1.54	1.47	60	-0.66	1.24	-0.88 [-1.37; -0.39]	1.9
Manoogian 2022 (USA)	40	28	FT=10h	Healthy	12	70	-0.04	0.27	66	0.27	0.24	-0.31 [-0.39; -0.22]	10.5
Suthutvoravut 2023 (Thailand)	55	30	FT=9h	Metabolic	12	49	-0.89	0.43	22	-0.65	0.43	-0.24 [-0.46; -0.02]	5.9
Montero 2023 (Spain)	48	.	FT=8h	Metabolic	12	148	0.00	0.25	49	0.05	0.32	-0.05 [-0.15; 0.05]	10.0
Lowe 2020 (USA)	46	33	FT=8h	Healthy	12	22	-0.02	0.18	24	-0.01	0.18	-0.02 [-0.12; 0.08]	9.9
Kunduraci & Ozbek 2020 (Turkey)	48	35	FT=8h	Metabolic	12	32	-0.32	0.07	33	-0.31	0.18	-0.01 [-0.08; 0.06]	11.2
Chow 2020 (USA)	46	34	FT=8h	Healthy	12	11	-0.00	0.01	9	0.00	0.01	-0.00 [-0.01; 0.00]	12.3
Jamshed 2022 (USA)	43	40	FT=8h	Healthy	14	45	-0.10	0.48	45	-0.10	0.48	0.00 [-0.20; 0.20]	6.4
Overall Effect						437			308			-0.11 [-0.20; -0.01]	68.1
Heterogeneity: $I^2 = 89\%$, $\tau^2 = 0.0127$, $\chi^2_7 = 66.11$ ($p < 0.01$)													
**Follow duration ≥6 months**													
Pavlou 2023 (USA)	55	39	FT=8h	Metabolic	26	25	-0.72	1.26	25	0.19	1.26	-0.91 [-1.61; -0.21]	1.0
Wei 2023 (China)	32	32	FT=8h	Metabolic	52	45	-0.20	0.48	43	-0.10	0.48	-0.10 [-0.30; 0.10]	6.4
Lin 2023 (USA)	44	38	FT=8h	Healthy	52	30	-0.01	0.34	30	0.07	0.34	-0.08 [-0.25; 0.09]	7.3
Thomas 2022 (USA)	38	34	FT=10h	Healthy	39	18	0.00	0.20	20	0.00	0.30	0.00 [-0.16; 0.16]	7.7
Philips 2021 (Switzerland)	40	28	FT=12h	Metabolic	26	23	0.03	0.18	18	-0.09	0.18	0.12 [0.01; 0.23]	9.6
Overall Effect						141			136			-0.04 [-0.19; 0.11]	31.9
Heterogeneity: $I^2 = 69\%$, $\tau^2 = 0.0179$, $\chi^2_4 = 12.73$ ($p = 0.01$)													
**Overall Effect**						**578**			**444**			**-0.08 [-0.15; -0.01]**	**100.0**

Heterogeneity: $I^2 = 85\%$, $\tau^2 = 0.0112$, $\chi^2_{12} = 79.12$ ($p < 0.01$)
Test for overall effect: $z = -2.18$ ($p = 0.03$)
Test for subgroup differences: $\chi^2_1 = 0.50$, df = 1 ($p = 0.48$)

eFigure 134: Meta-analysis of difference in mean difference (95% CIs) for the effect of time-restricted eating on HbA1c (%), grouped by follow duration

BMI: Body Mass Index; SD: Standard Deviation; CI: Confidence Interval; USA: United States of America; FT: Feeding Time; Duration: follow-up duration in weeks; 2M/3M/6M: 2, 3 or 6 meals; 3M+2S: 3 meals and 2 snacks; 3M+3S: 3 meals and 3 snacks; 10S: Grazing diet – 100kcal every 2-3 hours; HCB: High Calorie Breakfast; HCD: High Calorie Dinner; ED: Early Dinner; LD: Late Dinner; FL: Front Loading: EED: Equal Energy Distribution; ML: Middle Loading; BL: Back Loading.

© 2024 Liu HY et al. *JAMA Network Open.*

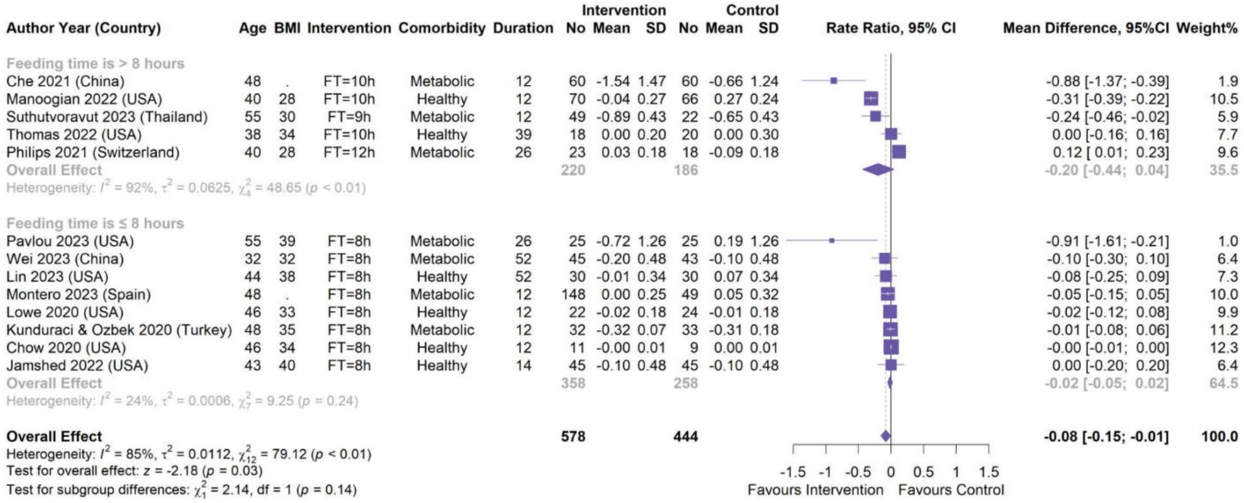

eFigure 135: Meta-analysis of difference in mean difference (95% CIs) for the effect of time-restricted eating on HbA1c (%), grouped by eating window

BMI: Body Mass Index; SD: Standard Deviation; CI: Confidence Interval; USA: United States of America; FT: Feeding Time; Duration: follow-up duration in weeks; 2M/3M/6M: 2, 3 or 6 meals; 3M+2S: 3 meals and 2 snacks; 3M+3S: 3 meals and 3 snacks; 10S: Grazing diet – 100kcal every 2-3 hours; HCB: High Calorie Breakfast; HCD: High Calorie Dinner; ED: Early Dinner; LD: Late Dinner; FL: Front Loading: EED: Equal Energy Distribution; ML: Middle Loading; BL: Back Loading.

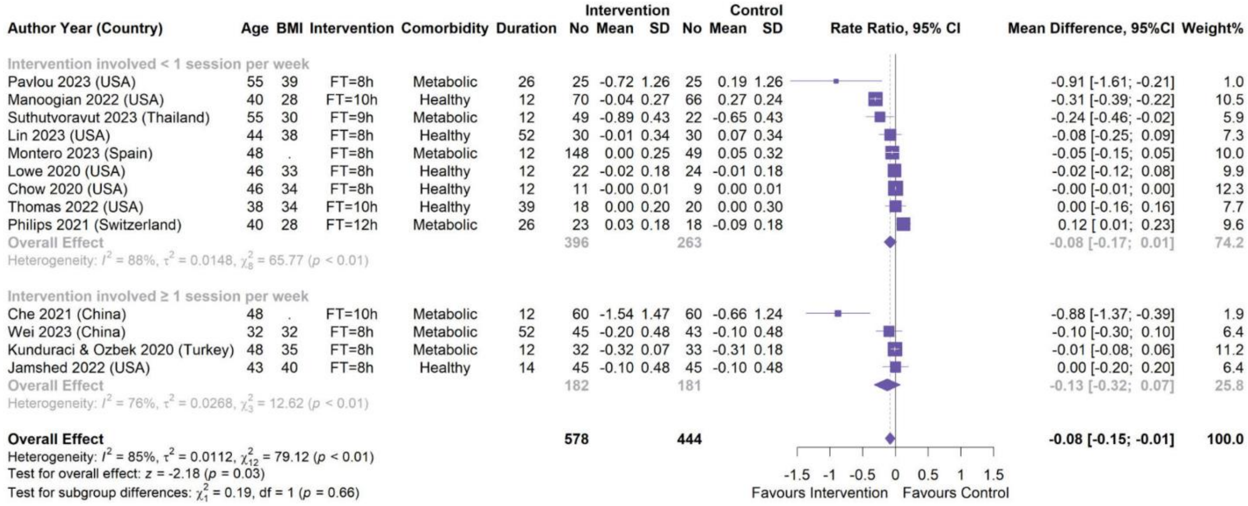

eFigure 136: Meta-analysis of difference in mean difference (95% CIs) for the effect of time-restricted eating on HbA1c (%), grouped by frequency of contact

BMI: Body Mass Index; SD: Standard Deviation; CI: Confidence Interval; USA: United States of America; FT: Feeding Time; Duration: follow-up duration in weeks; 2M/3M/6M: 2, 3 or 6 meals; 3M+2S: 3 meals and 2 snacks; 3M+3S: 3 meals and 3 snacks; 10S: Grazing diet – 100kcal every 2-3 hours; HCB: High Calorie Breakfast; HCD: High Calorie Dinner; ED: Early Dinner; LD: Late Dinner; FL: Front Loading: EED: Equal Energy Distribution; ML: Middle Loading; BL: Back Loading.

© 2024 Liu HY et al. *JAMA Network Open.*

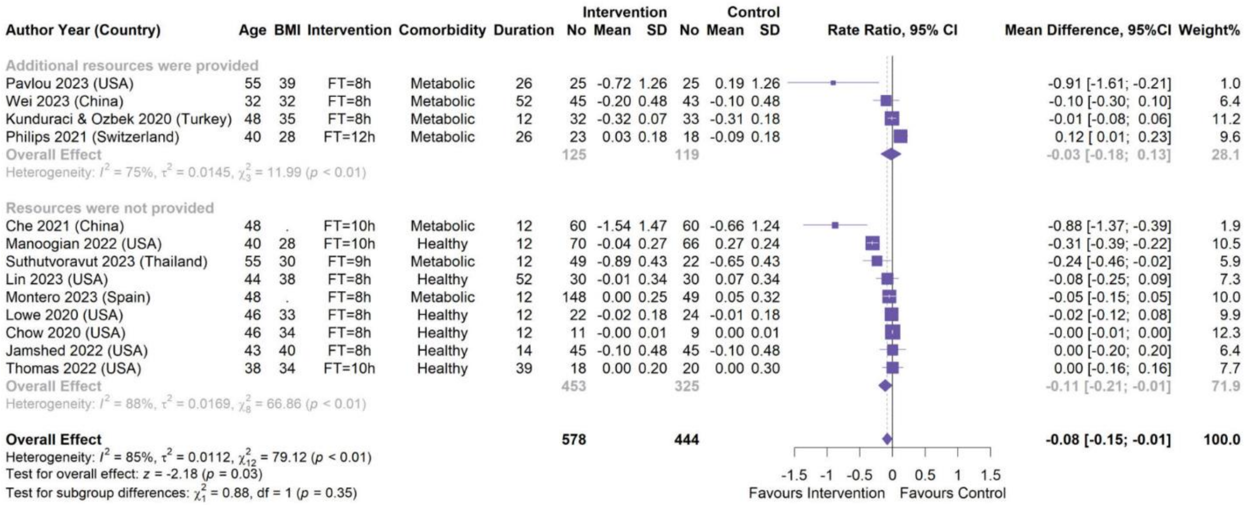

eFigure 137: Meta-analysis of difference in mean difference (95% CIs) for the effect of time-restricted eating on HbA1c (%), grouped by resource provision

BMI: Body Mass Index; SD: Standard Deviation; CI: Confidence Interval; USA: United States of America; FT: Feeding Time; Duration: follow-up duration in weeks; 2M/3M/6M: 2, 3 or 6 meals; 3M+2S: 3 meals and 2 snacks; 3M+3S: 3 meals and 3 snacks; 10S: Grazing diet – 100kcal every 2-3 hours; HCB: High Calorie Breakfast; HCD: High Calorie Dinner; ED: Early Dinner; LD: Late Dinner; FL: Front Loading: EED: Equal Energy Distribution; ML: Middle Loading; BL: Back Loading.

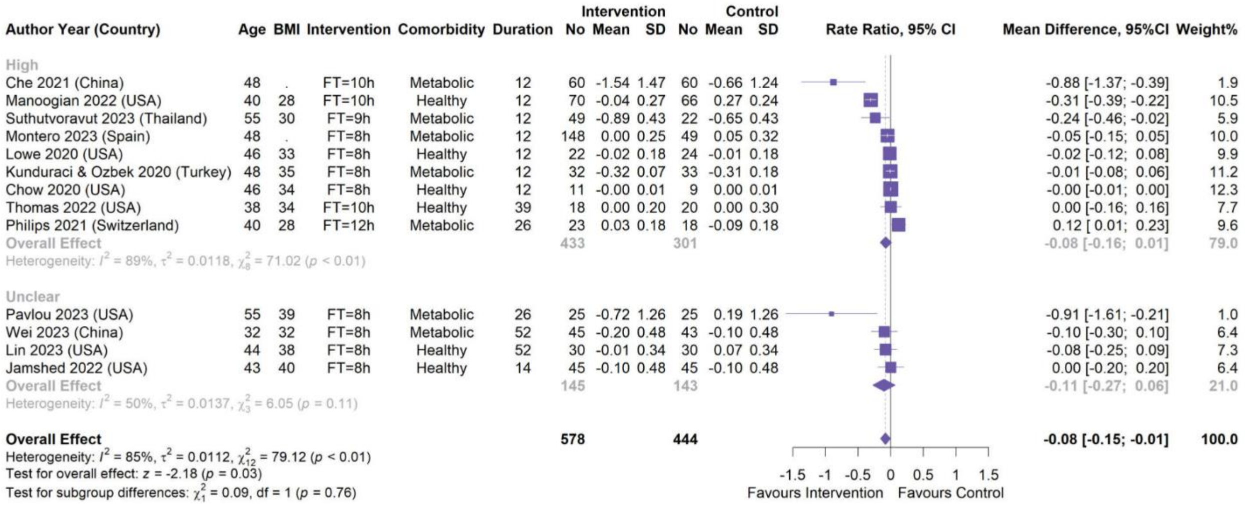

eFigure 138: Meta-analysis of difference in mean difference (95% CIs) for the effect of time-restricted eating on HbA1c (%), grouped by risk of bias

BMI: Body Mass Index; SD: Standard Deviation; CI: Confidence Interval; USA: United States of America; FT: Feeding Time; Duration: follow-up duration in weeks; 2M/3M/6M: 2, 3 or 6 meals; 3M+2S: 3 meals and 2 snacks; 3M+3S: 3 meals and 3 snacks; 10S: Grazing diet – 100kcal every 2-3 hours; HCB: High Calorie Breakfast; HCD: High Calorie Dinner; ED: Early Dinner; LD: Late Dinner; FL: Front Loading: EED: Equal Energy Distribution; ML: Middle Loading; BL: Back Loading.

© 2024 Liu HY et al. *JAMA Network Open.*

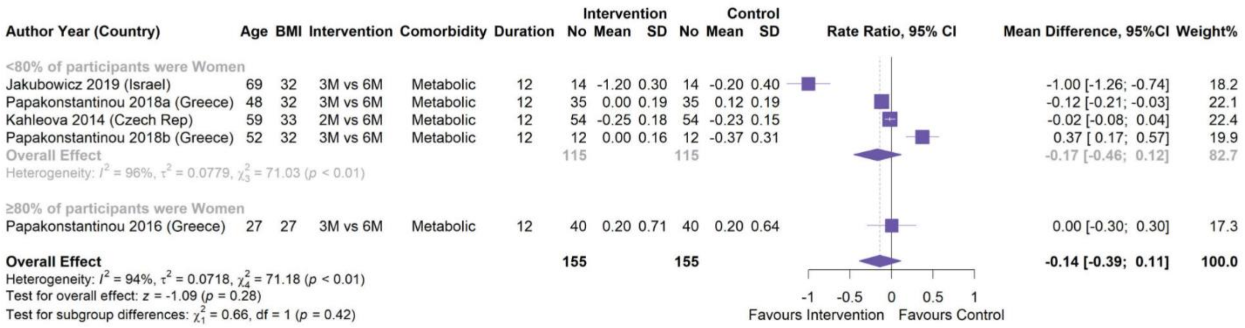

eFigure 139: Meta-analysis of difference in mean difference (95% CIs) for the effect of meal frequency on HbA1c (%), grouped by gender proportion

BMI: Body Mass Index; SD: Standard Deviation; CI: Confidence Interval; USA: United States of America; FT: Feeding Time; Duration: follow-up duration in weeks; 2M/3M/6M: 2, 3 or 6 meals; 3M+2S: 3 meals and 2 snacks; 3M+3S: 3 meals and 3 snacks; 10S: Grazing diet – 100kcal every 2-3 hours; HCB: High Calorie Breakfast; HCD: High Calorie Dinner; ED: Early Dinner; LD: Late Dinner; FL: Front Loading: EED: Equal Energy Distribution; ML: Middle Loading; BL: Back Loading.

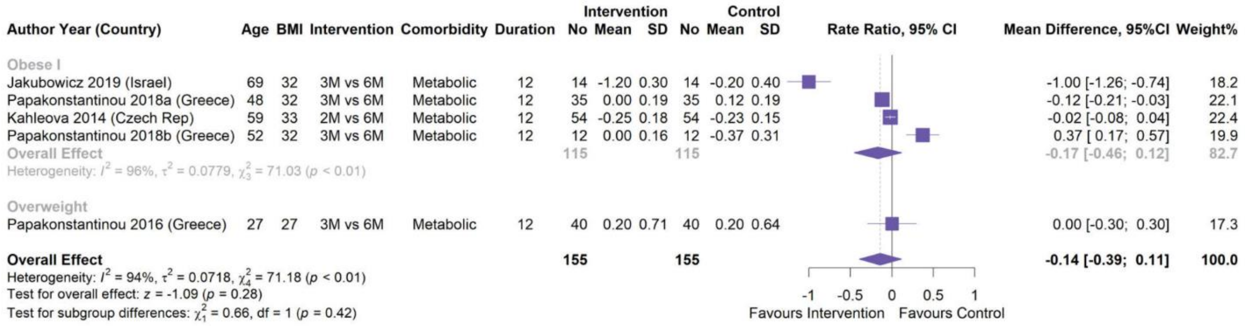

eFigure 140: Meta-analysis of difference in mean difference (95% CIs) for the effect of meal frequency on HbA1c (%), grouped by baseline BMI status

BMI: Body Mass Index; SD: Standard Deviation; CI: Confidence Interval; USA: United States of America; FT: Feeding Time; Duration: follow-up duration in weeks; 2M/3M/6M: 2, 3 or 6 meals; 3M+2S: 3 meals and 2 snacks; 3M+3S: 3 meals and 3 snacks; 10S: Grazing diet – 100kcal every 2-3 hours; HCB: High Calorie Breakfast; HCD: High Calorie Dinner; ED: Early Dinner; LD: Late Dinner; FL: Front Loading: EED: Equal Energy Distribution; ML: Middle Loading; BL: Back Loading.

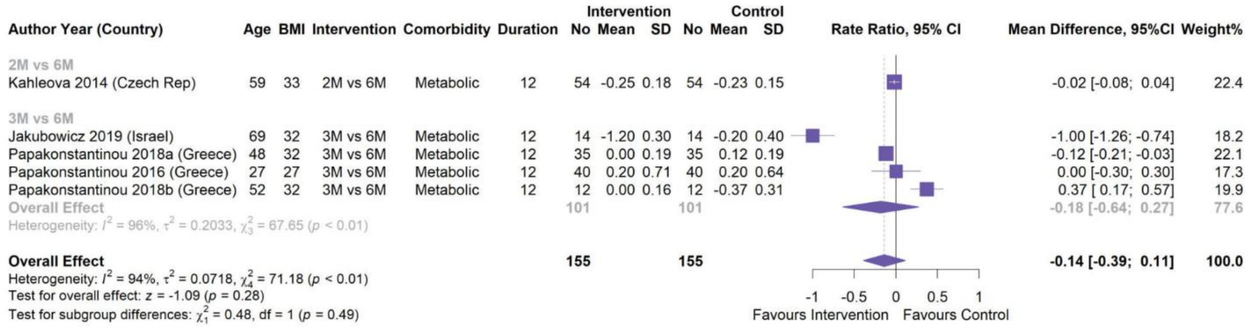

eFigure 141: Meta-analysis of difference in mean difference (95% CIs) for the effect of meal frequency on HbA1c (%), grouped by intervention intensity

BMI: Body Mass Index; SD: Standard Deviation; CI: Confidence Interval; USA: United States of America; FT: Feeding Time; Duration: follow-up duration in weeks; 2M/3M/6M: 2, 3 or 6 meals; 3M+2S: 3 meals and 2 snacks; 3M+3S: 3 meals and 3 snacks; 10S: Grazing diet – 100kcal every 2-3 hours; HCB: High Calorie Breakfast; HCD: High Calorie Dinner; ED: Early Dinner; LD: Late Dinner; FL: Front Loading: EED: Equal Energy Distribution; ML: Middle Loading; BL: Back Loading.

© 2024 Liu HY et al. *JAMA Network Open.*

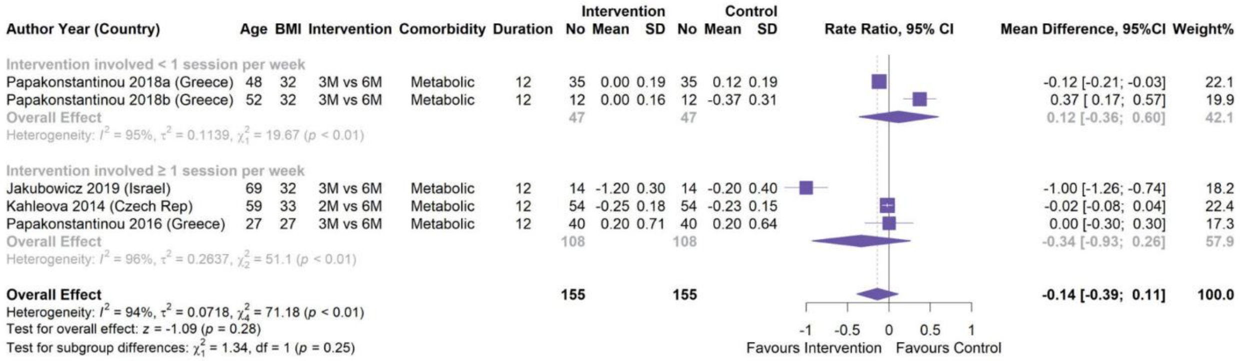

eFigure 142: Meta-analysis of difference in mean difference (95% CIs) for the effect of meal frequency on HbA1c (%), grouped by frequency of contact

BMI: Body Mass Index; SD: Standard Deviation; CI: Confidence Interval; USA: United States of America; FT: Feeding Time; Duration: follow-up duration in weeks; 2M/3M/6M: 2, 3 or 6 meals; 3M+2S: 3 meals and 2 snacks; 3M+3S: 3 meals and 3 snacks; 10S: Grazing diet – 100kcal every 2-3 hours; HCB: High Calorie Breakfast; HCD: High Calorie Dinner; ED: Early Dinner; LD: Late Dinner; FL: Front Loading: EED: Equal Energy Distribution; ML: Middle Loading; BL: Back Loading.

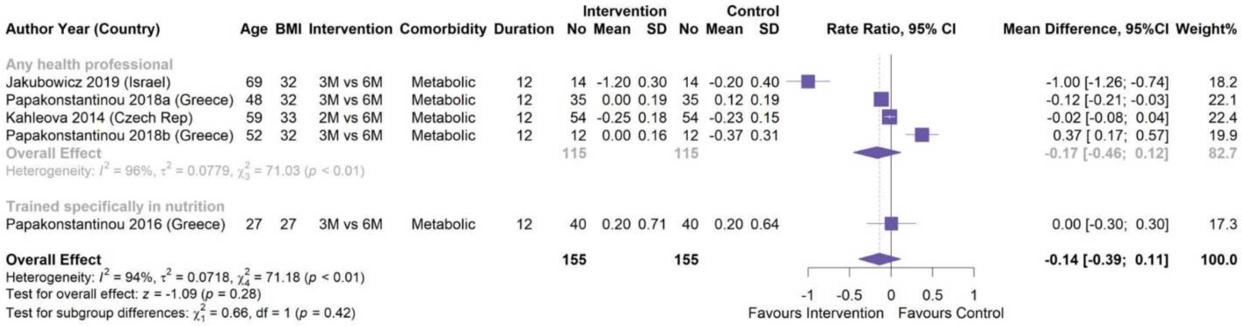

eFigure 143: Meta-analysis of difference in mean difference (95% CIs) for the effect of meal frequency on HbA1c (%), grouped by delivery personnel

BMI: Body Mass Index; SD: Standard Deviation; CI: Confidence Interval; USA: United States of America; FT: Feeding Time; Duration: follow-up duration in weeks; 2M/3M/6M: 2, 3 or 6 meals; 3M+2S: 3 meals and 2 snacks; 3M+3S: 3 meals and 3 snacks; 10S: Grazing diet – 100kcal every 2-3 hours; HCB: High Calorie Breakfast; HCD: High Calorie Dinner; ED: Early Dinner; LD: Late Dinner; FL: Front Loading: EED: Equal Energy Distribution; ML: Middle Loading; BL: Back Loading.

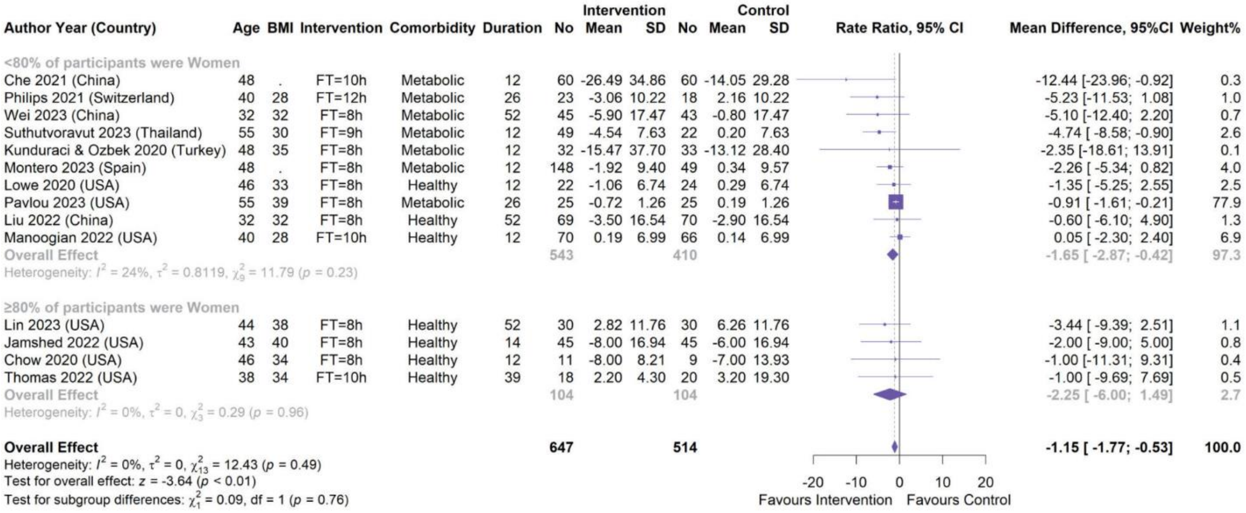

eFigure 144: Meta-analysis of difference in mean difference (95% CIs) for the effect of time-restricted eating on fasting glucose (mg/dL), grouped by gender proportion

© 2024 Liu HY et al. *JAMA Network Open.*

BMI: Body Mass Index; SD: Standard Deviation; CI: Confidence Interval; USA: United States of America; FT: Feeding Time; Duration: follow-up duration in weeks; 2M/3M/6M: 2, 3 or 6 meals; 3M+2S: 3 meals and 2 snacks; 3M+3S: 3 meals and 3 snacks; 10S: Grazing diet – 100kcal every 2-3 hours; HCB: High Calorie Breakfast; HCD: High Calorie Dinner; ED: Early Dinner; LD: Late Dinner; FL: Front Loading: EED: Equal Energy Distribution; ML: Middle Loading; BL: Back Loading.

Author Year (Country)	Age	BMI	Intervention	Comorbidity	Duration	Intervention No	Intervention Mean	Intervention SD	Control No	Control Mean	Control SD	Mean Difference, 95%CI	Weight%
Obese I													
Wei 2023 (China)	32	32	FT=8h	Metabolic	52	45	-5.90	17.47	43	-0.80	17.47	-5.10 [-12.40; 2.20]	0.7
Kunduraci & Ozbek 2020 (Turkey)	48	35	FT=8h	Metabolic	12	32	-15.47	37.70	33	-13.12	28.40	-2.35 [-18.61; 13.91]	0.1
Montero 2023 (Spain)	48	.	FT=8h	Metabolic	12	148	-1.92	9.40	49	0.34	9.57	-2.26 [-5.34; 0.82]	4.0
Lowe 2020 (USA)	46	33	FT=8h	Healthy	12	22	-1.06	6.74	24	0.29	6.74	-1.35 [-5.25; 2.55]	2.5
Chow 2020 (USA)	46	34	FT=8h	Healthy	12	11	-8.00	8.21	9	-7.00	13.93	-1.00 [-11.31; 9.31]	0.4
Thomas 2022 (USA)	38	34	FT=10h	Healthy	39	18	2.20	4.30	20	3.20	19.30	-1.00 [-9.69; 7.69]	0.5
Liu 2022 (China)	32	32	FT=8h	Healthy	52	69	-3.50	16.54	70	-2.90	16.54	-0.60 [-6.10; 4.90]	1.3
Overall Effect						345			248			-1.90 [-3.90; 0.10]	9.5
Heterogeneity: $I^2 = 0\%$, $\tau^2 = 0$, $\chi^2_6 = 1.16$ ($p = 0.98$)													
Obese II													
Lin 2023 (USA)	44	38	FT=8h	Healthy	52	30	2.82	11.76	30	6.26	11.76	-3.44 [-9.39; 2.51]	1.1
Jamshed 2022 (USA)	43	40	FT=8h	Healthy	14	45	-8.00	16.94	45	-6.00	16.94	-2.00 [-9.00; 5.00]	0.8
Pavlou 2023 (USA)	55	39	FT=8h	Metabolic	26	25	-0.72	1.26	25	0.19	1.26	-0.91 [-1.61; -0.21]	77.9
Overall Effect						100			100			-0.95 [-1.65; -0.26]	79.7
Heterogeneity: $I^2 = 0\%$, $\tau^2 = 0$, $\chi^2_2 = 0.77$ ($p = 0.68$)													
Overweight													
Che 2021 (China)	48	.	FT=10h	Metabolic	12	60	-26.49	34.86	60	-14.05	29.28	-12.44 [-23.96; -0.92]	0.3
Philips 2021 (Switzerland)	40	28	FT=12h	Metabolic	26	23	-3.06	10.22	18	2.16	10.22	-5.23 [-11.53; 1.08]	1.0
Suthutvoravut 2023 (Thailand)	55	30	FT=9h	Metabolic	12	49	-4.54	7.63	22	0.20	7.63	-4.74 [-8.58; -0.90]	2.6
Manoogian 2022 (USA)	40	28	FT=10h	Healthy	12	70	0.19	6.99	66	0.14	6.99	0.05 [-2.30; 2.40]	6.9
Overall Effect						202			166			-3.83 [-8.00; 0.35]	10.7
Heterogeneity: $I^2 = 67\%$, $\tau^2 = 10.7272$, $\chi^2_3 = 9.03$ ($p = 0.03$)													
**Overall Effect**						**647**			**514**			**-1.15 [-1.77; -0.53]**	**100.0**

Heterogeneity: $I^2 = 0\%$, $\tau^2 = 0$, $\chi^2_{13} = 12.43$ ($p = 0.49$)
Test for overall effect: $z = -3.64$ ($p < 0.01$)
Test for subgroup differences: $\chi^2_2 = 2.42$, df = 2 ($p = 0.30$)

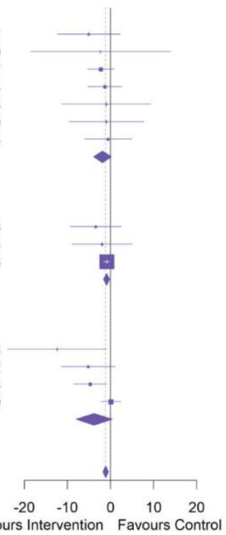

eFigure 145: Meta-analysis of difference in mean difference (95% CIs) for the effect of time-restricted eating on fasting glucose (mg/dL), grouped by baseline BMI status

BMI: Body Mass Index; SD: Standard Deviation; CI: Confidence Interval; USA: United States of America; FT: Feeding Time; Duration: follow-up duration in weeks; 2M/3M/6M: 2, 3 or 6 meals; 3M+2S: 3 meals and 2 snacks; 3M+3S: 3 meals and 3 snacks; 10S: Grazing diet – 100kcal every 2-3 hours; HCB: High Calorie Breakfast; HCD: High Calorie Dinner; ED: Early Dinner; LD: Late Dinner; FL: Front Loading: EED: Equal Energy Distribution; ML: Middle Loading; BL: Back Loading.

Author Year (Country)	Age	BMI	Intervention	Comorbidity	Duration	Intervention No	Intervention Mean	Intervention SD	Control No	Control Mean	Control SD	Mean Difference, 95%CI	Weight%
Healthy													
Lin 2023 (USA)	44	38	FT=8h	Healthy	52	30	2.82	11.76	30	6.26	11.76	-3.44 [-9.39; 2.51]	1.1
Jamshed 2022 (USA)	43	40	FT=8h	Healthy	14	45	-8.00	16.94	45	-6.00	16.94	-2.00 [-9.00; 5.00]	0.8
Lowe 2020 (USA)	46	33	FT=8h	Healthy	12	22	-1.06	6.74	24	0.29	6.74	-1.35 [-5.25; 2.55]	2.5
Chow 2020 (USA)	46	34	FT=8h	Healthy	12	11	-8.00	8.21	9	-7.00	13.93	-1.00 [-11.31; 9.31]	0.4
Thomas 2022 (USA)	38	34	FT=10h	Healthy	39	18	2.20	4.30	20	3.20	19.30	-1.00 [-9.69; 7.69]	0.5
Liu 2022 (China)	32	32	FT=8h	Healthy	52	69	-3.50	16.54	70	-2.90	16.54	-0.60 [-6.10; 4.90]	1.3
Manoogian 2022 (USA)	40	28	FT=10h	Healthy	12	70	0.19	6.99	66	0.14	6.99	0.05 [-2.30; 2.40]	6.9
Overall Effect						265			264			-0.74 [-2.43; 0.95]	13.4
Heterogeneity: $I^2 = 0\%$, $\tau^2 = 0$, $\chi^2_6 = 1.45$ ($p = 0.96$)													
Metabolic													
Che 2021 (China)	48	.	FT=10h	Metabolic	12	60	-26.49	34.86	60	-14.05	29.28	-12.44 [-23.96; -0.92]	0.3
Philips 2021 (Switzerland)	40	28	FT=12h	Metabolic	26	23	-3.06	10.22	18	2.16	10.22	-5.23 [-11.53; 1.08]	1.0
Wei 2023 (China)	32	32	FT=8h	Metabolic	52	45	-5.90	17.47	43	-0.80	17.47	-5.10 [-12.40; 2.20]	0.7
Suthutvoravut 2023 (Thailand)	55	30	FT=9h	Metabolic	12	49	-4.54	7.63	22	0.20	7.63	-4.74 [-8.58; -0.90]	2.6
Kunduraci & Ozbek 2020 (Turkey)	48	35	FT=8h	Metabolic	12	32	-15.47	37.70	33	-13.12	28.40	-2.35 [-18.61; 13.91]	0.1
Montero 2023 (Spain)	48	.	FT=8h	Metabolic	12	148	-1.92	9.40	49	0.34	9.57	-2.26 [-5.34; 0.82]	4.0
Pavlou 2023 (USA)	55	39	FT=8h	Metabolic	26	25	-0.72	1.26	25	0.19	1.26	-0.91 [-1.61; -0.21]	77.9
Overall Effect						382			250			-2.95 [-5.09; -0.82]	86.6
Heterogeneity: $I^2 = 44\%$, $\tau^2 = 2.8774$, $\chi^2_6 = 10.72$ ($p = 0.10$)													
**Overall Effect**						**647**			**514**			**-1.15 [-1.77; -0.53]**	**100.0**

Heterogeneity: $I^2 = 0\%$, $\tau^2 = 0$, $\chi^2_{13} = 12.43$ ($p = 0.49$)
Test for overall effect: $z = -3.64$ ($p < 0.01$)
Test for subgroup differences: $\chi^2_1 = 2.55$, df = 1 ($p = 0.11$)

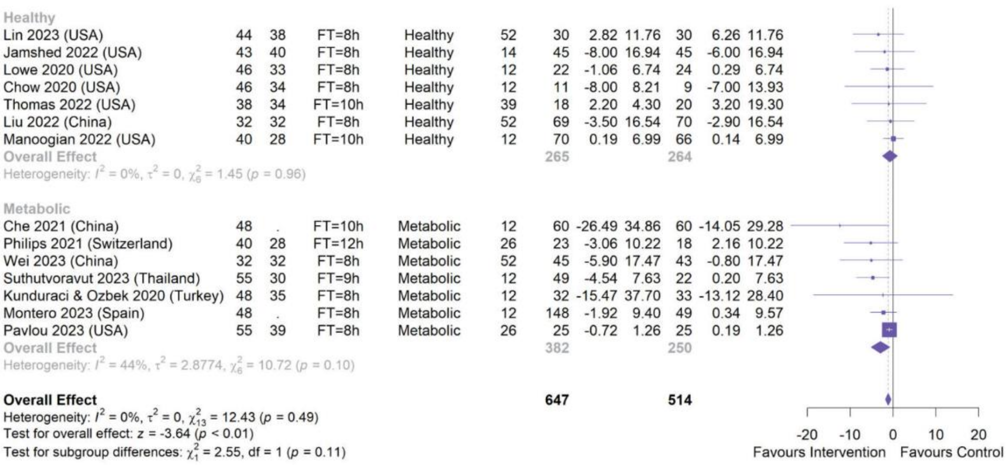

eFigure 146: Meta-analysis of difference in mean difference (95% CIs) for the effect of time-restricted eating on fasting glucose (mg/dL), grouped by health status

BMI: Body Mass Index; SD: Standard Deviation; CI: Confidence Interval; USA: United States of America; FT: Feeding Time; Duration: follow-up duration in weeks; 2M/3M/6M: 2, 3 or 6 meals; 3M+2S: 3 meals and 2 snacks; 3M+3S: 3 meals and 3 snacks; 10S: Grazing diet – 100kcal every 2-3 hours; HCB: High Calorie Breakfast; HCD: High Calorie Dinner; ED: Early Dinner; LD: Late Dinner; FL: Front Loading: EED: Equal Energy Distribution; ML: Middle Loading; BL: Back Loading.

© 2024 Liu HY et al. *JAMA Network Open.*

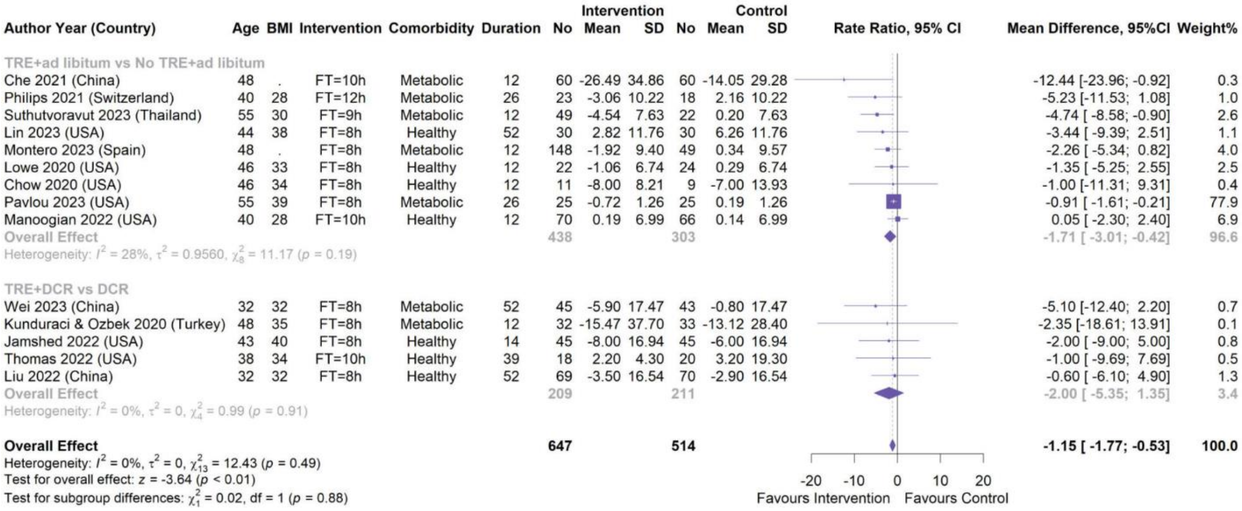

eFigure 147: Meta-analysis of difference in mean difference (95% CIs) for the effect of time-restricted eating on fasting glucose (mg/dL), grouped by energy prescription

BMI: Body Mass Index; SD: Standard Deviation; CI: Confidence Interval; USA: United States of America; FT: Feeding Time; Duration: follow-up duration in weeks; 2M/3M/6M: 2, 3 or 6 meals; 3M+2S: 3 meals and 2 snacks; 3M+3S: 3 meals and 3 snacks; 10S: Grazing diet – 100kcal every 2-3 hours; HCB: High Calorie Breakfast; HCD: High Calorie Dinner; ED: Early Dinner; LD: Late Dinner; FL: Front Loading: EED: Equal Energy Distribution; ML: Middle Loading; BL: Back Loading.

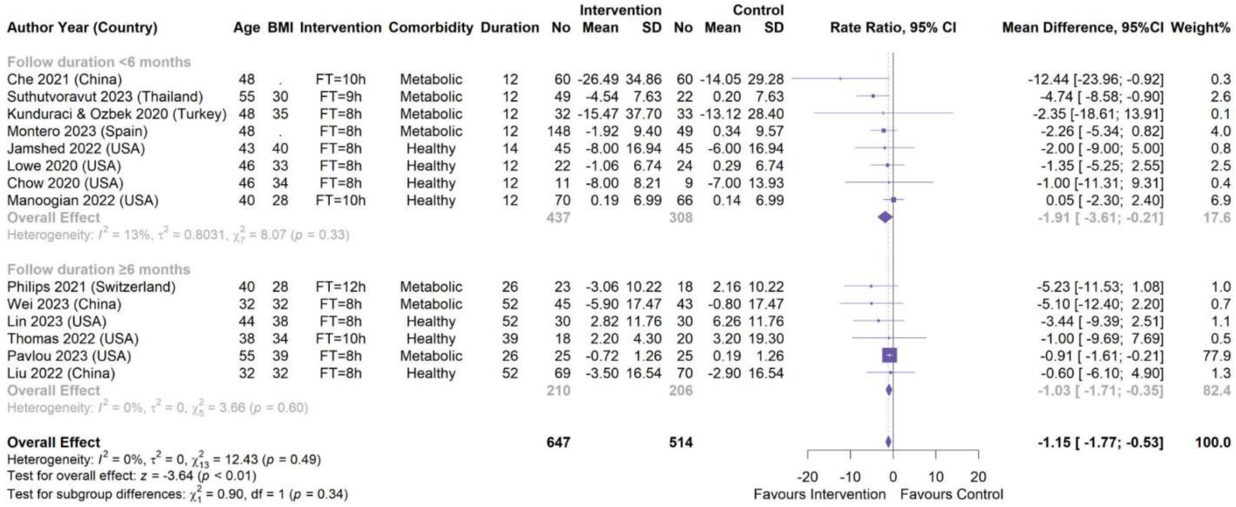

eFigure 148: Meta-analysis of difference in mean difference (95% CIs) for the effect of time-restricted eating on fasting glucose (mg/dL), grouped by follow duration

BMI: Body Mass Index; SD: Standard Deviation; CI: Confidence Interval; USA: United States of America; FT: Feeding Time; Duration: follow-up duration in weeks; 2M/3M/6M: 2, 3 or 6 meals; 3M+2S: 3 meals and 2 snacks; 3M+3S: 3 meals and 3 snacks; 10S: Grazing diet – 100kcal every 2-3 hours; HCB: High Calorie Breakfast; HCD: High Calorie Dinner; ED: Early Dinner; LD: Late Dinner; FL: Front Loading: EED: Equal Energy Distribution; ML: Middle Loading; BL: Back Loading.

© 2024 Liu HY et al. *JAMA Network Open.*

Author Year (Country)	Age	BMI	Intervention	Comorbidity	Duration	Intervention No	Intervention Mean	Intervention SD	Control No	Control Mean	Control SD	Mean Difference, 95%CI	Weight%
**Feeding time is > 8 hours**													
Che 2021 (China)	48	.	FT=10h	Metabolic	12	60	-26.49	34.86	60	-14.05	29.28	-12.44 [-23.96; -0.92]	0.3
Philips 2021 (Switzerland)	40	28	FT=12h	Metabolic	26	23	-3.06	10.22	18	2.16	10.22	-5.23 [-11.53; 1.08]	1.0
Suthutvoravut 2023 (Thailand)	55	30	FT=9h	Metabolic	12	49	-4.54	7.63	22	0.20	7.63	-4.74 [-8.58; -0.90]	2.6
Thomas 2022 (USA)	38	34	FT=10h	Healthy	39	18	2.20	4.30	20	3.20	19.30	-1.00 [-9.69; 7.69]	0.5
Manoogian 2022 (USA)	40	28	FT=10h	Healthy	12	70	0.19	6.99	66	0.14	6.99	0.05 [-2.30; 2.40]	6.9
Overall Effect						220			186			-3.31 [-6.86; 0.24]	11.2
Heterogeneity: $I^2 = 56\%$, $\tau^2 = 7.9966$, $\chi^2_4 = 9.07$ ($p = 0.06$)													
**Feeding time is ≤ 8 hours**													
Wei 2023 (China)	32	32	FT=8h	Metabolic	52	45	-5.90	17.47	43	-0.80	17.47	-5.10 [-12.40; 2.20]	0.7
Lin 2023 (USA)	44	38	FT=8h	Healthy	52	30	2.82	11.76	30	6.26	11.76	-3.44 [-9.39; 2.51]	1.1
Kunduraci & Ozbek 2020 (Turkey)	48	35	FT=8h	Metabolic	12	32	-15.47	37.70	33	-13.12	28.40	-2.35 [-18.61; 13.91]	0.1
Montero 2023 (Spain)	48	.	FT=8h	Metabolic	12	148	-1.92	9.40	49	0.34	9.57	-2.26 [-5.34; 0.82]	4.0
Jamshed 2022 (USA)	43	40	FT=8h	Healthy	14	45	-8.00	16.94	45	-6.00	16.94	-2.00 [-9.00; 5.00]	0.8
Lowe 2020 (USA)	46	33	FT=8h	Healthy	12	22	-1.06	6.74	24	0.29	6.74	-1.35 [-5.25; 2.55]	2.5
Chow 2020 (USA)	46	34	FT=8h	Healthy	12	11	-8.00	8.21	9	-7.00	13.93	-1.00 [-11.31; 9.31]	0.4
Pavlou 2023 (USA)	55	39	FT=8h	Metabolic	26	25	-0.72	1.26	25	0.19	1.26	-0.91 [-1.61; -0.21]	77.9
Liu 2022 (China)	32	32	FT=8h	Healthy	52	69	-3.50	16.54	70	-2.90	16.54	-0.60 [-6.10; 4.90]	1.3
Overall Effect						427			328			-1.06 [-1.71; -0.40]	88.8
Heterogeneity: $I^2 = 0\%$, $\tau^2 = 0$, $\chi^2_8 = 2.69$ ($p = 0.95$)													
**Overall Effect**						**647**			**514**			**-1.15 [-1.77; -0.53]**	**100.0**

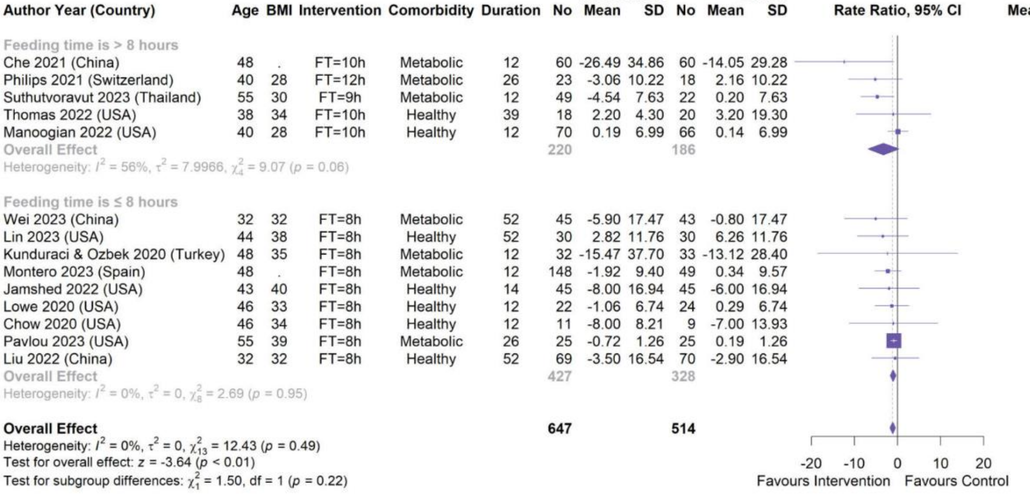

Heterogeneity: $I^2 = 0\%$, $\tau^2 = 0$, $\chi^2_{13} = 12.43$ ($p = 0.49$)
Test for overall effect: $z = -3.64$ ($p < 0.01$)
Test for subgroup differences: $\chi^2_1 = 1.50$, df = 1 ($p = 0.22$)

eFigure 149: Meta-analysis of difference in mean difference (95% CIs) for the effect of time-restricted eating on fasting glucose (mg/dL), grouped by eating window

BMI: Body Mass Index; SD: Standard Deviation; CI: Confidence Interval; USA: United States of America; FT: Feeding Time; Duration: follow-up duration in weeks; 2M/3M/6M: 2, 3 or 6 meals; 3M+2S: 3 meals and 2 snacks; 3M+3S: 3 meals and 3 snacks; 10S: Grazing diet – 100kcal every 2-3 hours; HCB: High Calorie Breakfast; HCD: High Calorie Dinner; ED: Early Dinner; LD: Late Dinner; FL: Front Loading: EED: Equal Energy Distribution; ML: Middle Loading; BL: Back Loading.

Author Year (Country)	Age	BMI	Intervention	Comorbidity	Duration	Intervention No	Intervention Mean	Intervention SD	Control No	Control Mean	Control SD	Mean Difference, 95%CI	Weight%
**Intervention involved < 1 session per week**													
Philips 2021 (Switzerland)	40	28	FT=12h	Metabolic	26	23	-3.06	10.22	18	2.16	10.22	-5.23 [-11.53; 1.08]	1.0
Suthutvoravut 2023 (Thailand)	55	30	FT=9h	Metabolic	12	49	-4.54	7.63	22	0.20	7.63	-4.74 [-8.58; -0.90]	2.6
Lin 2023 (USA)	44	38	FT=8h	Healthy	52	30	2.82	11.76	30	6.26	11.76	-3.44 [-9.39; 2.51]	1.1
Montero 2023 (Spain)	48	.	FT=8h	Metabolic	12	148	-1.92	9.40	49	0.34	9.57	-2.26 [-5.34; 0.82]	4.0
Lowe 2020 (USA)	46	33	FT=8h	Healthy	12	22	-1.06	6.74	24	0.29	6.74	-1.35 [-5.25; 2.55]	2.5
Chow 2020 (USA)	46	34	FT=8h	Healthy	12	11	-8.00	8.21	9	-7.00	13.93	-1.00 [-11.31; 9.31]	0.4
Thomas 2022 (USA)	38	34	FT=10h	Healthy	39	18	2.20	4.30	20	3.20	19.30	-1.00 [-9.69; 7.69]	0.5
Pavlou 2023 (USA)	55	39	FT=8h	Metabolic	26	25	-0.72	1.26	25	0.19	1.26	-0.91 [-1.61; -0.21]	77.9
Liu 2022 (China)	32	32	FT=8h	Healthy	52	69	-3.50	16.54	70	-2.90	16.54	-0.60 [-6.10; 4.90]	1.3
Manoogian 2022 (USA)	40	28	FT=10h	Healthy	12	70	0.19	6.99	66	0.14	6.99	0.05 [-2.30; 2.40]	6.9
Overall Effect						465			333			-1.08 [-1.70; -0.45]	98.1
Heterogeneity: $I^2 = 0\%$, $\tau^2 = 0$, $\chi^2_9 = 7.48$ ($p = 0.59$)													
**Intervention involved ≥ 1 session per week**													
Che 2021 (China)	48	.	FT=10h	Metabolic	12	60	-26.49	34.86	60	-14.05	29.28	-12.44 [-23.96; -0.92]	0.3
Wei 2023 (China)	32	32	FT=8h	Metabolic	52	45	-5.90	17.47	43	-0.80	17.47	-5.10 [-12.40; 2.20]	0.7
Kunduraci & Ozbek 2020 (Turkey)	48	35	FT=8h	Metabolic	12	32	-15.47	37.70	33	-13.12	28.40	-2.35 [-18.61; 13.91]	0.1
Jamshed 2022 (USA)	43	40	FT=8h	Healthy	14	45	-8.00	16.94	45	-6.00	16.94	-2.00 [-9.00; 5.00]	0.8
Overall Effect						182			181			-4.74 [-9.19; -0.29]	1.9
Heterogeneity: $I^2 = 0\%$, $\tau^2 = 0$, $\chi^2_3 = 2.4$ ($p = 0.49$)													
**Overall Effect**						**647**			**514**			**-1.15 [-1.77; -0.53]**	**100.0**

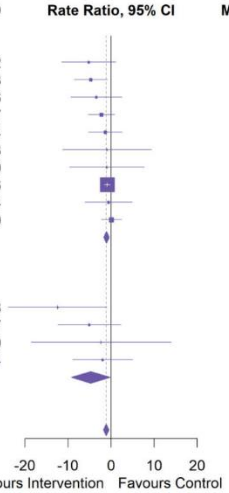

Heterogeneity: $I^2 = 0\%$, $\tau^2 = 0$, $\chi^2_{13} = 12.43$ ($p = 0.49$)
Test for overall effect: $z = -3.64$ ($p < 0.01$)
Test for subgroup differences: $\chi^2_1 = 2.55$, df = 1 ($p = 0.11$)

eFigure 150: Meta-analysis of difference in mean difference (95% CIs) for the effect of time-restricted eating on fasting glucose (mg/dL), grouped by frequency of contact

BMI: Body Mass Index; SD: Standard Deviation; CI: Confidence Interval; USA: United States of America; FT: Feeding Time; Duration: follow-up duration in weeks; 2M/3M/6M: 2, 3 or 6 meals; 3M+2S: 3 meals and 2 snacks; 3M+3S: 3 meals and 3 snacks; 10S: Grazing diet – 100kcal every 2-3 hours; HCB: High Calorie Breakfast; HCD: High Calorie Dinner; ED: Early Dinner; LD: Late Dinner; FL: Front Loading: EED: Equal Energy Distribution; ML: Middle Loading; BL: Back Loading.

© 2024 Liu HY et al. *JAMA Network Open.*

Author Year (Country)	Age	BMI	Intervention	Comorbidity	Duration	Intervention No	Intervention Mean	Intervention SD	Control No	Control Mean	Control SD	Mean Difference, 95%CI	Weight%
**Any health professional**													
Philips 2021 (Switzerland)	40	28	FT=12h	Metabolic	26	23	-3.06	10.22	18	2.16	10.22	-5.23 [-11.53; 1.08]	1.0
Suthutvoravut 2023 (Thailand)	55	30	FT=9h	Metabolic	12	49	-4.54	7.63	22	0.20	7.63	-4.74 [-8.58; -0.90]	2.6
Kunduraci & Ozbek 2020 (Turkey)	48	35	FT=8h	Metabolic	12	32	-15.47	37.70	33	-13.12	28.40	-2.35 [-18.61; 13.91]	0.1
Montero 2023 (Spain)	48	.	FT=8h	Metabolic	12	148	-1.92	9.40	49	0.34	9.57	-2.26 [-5.34; 0.82]	4.0
Lowe 2020 (USA)	46	33	FT=8h	Healthy	12	22	-1.06	6.74	24	0.29	6.74	-1.35 [-5.25; 2.55]	2.5
Chow 2020 (USA)	46	34	FT=8h	Healthy	12	11	-8.00	8.21	9	-7.00	13.93	-1.00 [-11.31; 9.31]	0.4
Liu 2022 (China)	32	32	FT=8h	Healthy	52	69	-3.50	16.54	70	-2.90	16.54	-0.60 [-6.10; 4.90]	1.3
Overall Effect						354			225			-2.64 [-4.43; -0.84]	11.8
Heterogeneity: $I^2 = 0\%$, $\tau^2 = 0$, $\chi^2_6 = 2.9$ ($p = 0.82$)													
**Trained specifically in nutrition**													
Che 2021 (China)	48	.	FT=10h	Metabolic	12	60	-26.49	34.86	60	-14.05	29.28	-12.44 [-23.96; -0.92]	0.3
Wei 2023 (China)	32	32	FT=8h	Metabolic	52	45	-5.90	17.47	43	-0.80	17.47	-5.10 [-12.40; 2.20]	0.7
Lin 2023 (USA)	44	38	FT=8h	Healthy	52	30	2.82	11.76	30	6.26	11.76	-3.44 [-9.39; 2.51]	1.1
Jamshed 2022 (USA)	43	40	FT=8h	Healthy	14	45	-8.00	16.94	45	-6.00	16.94	-2.00 [-9.00; 5.00]	0.8
Thomas 2022 (USA)	38	34	FT=10h	Healthy	39	18	2.20	4.30	20	3.20	19.30	-1.00 [-9.69; 7.69]	0.5
Pavlou 2023 (USA)	55	39	FT=8h	Metabolic	26	25	-0.72	1.26	25	0.19	1.26	-0.91 [-1.61; -0.21]	77.9
Manoogian 2022 (USA)	40	28	FT=10h	Healthy	12	70	0.19	6.99	66	0.14	6.99	0.05 [-2.30; 2.40]	6.9
Overall Effect						293			289			-1.04 [-2.10; 0.03]	88.2
Heterogeneity: $I^2 = 8\%$, $\tau^2 = 0.2810$, $\chi^2_6 = 6.53$ ($p = 0.37$)													
**Overall Effect**						**647**			**514**			**-1.15 [-1.77; -0.53]**	**100.0**
Heterogeneity: $I^2 = 0\%$, $\tau^2 = 0$, $\chi^2_{13} = 12.43$ ($p = 0.49$)													
Test for overall effect: $z = -3.64$ ($p < 0.01$)													
Test for subgroup differences: $\chi^2_1 = 2.25$, df = 1 ($p = 0.13$)													

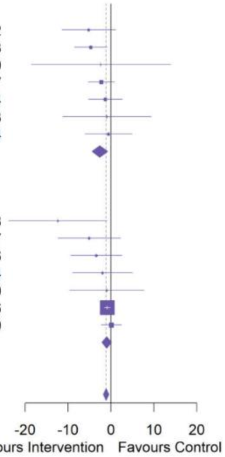

eFigure 151: Meta-analysis of difference in mean difference (95% CIs) for the effect of time-restricted eating on fasting glucose (mg/dL), grouped by delivery personnel

BMI: Body Mass Index; SD: Standard Deviation; CI: Confidence Interval; USA: United States of America; FT: Feeding Time; Duration: follow-up duration in weeks; 2M/3M/6M: 2, 3 or 6 meals; 3M+2S: 3 meals and 2 snacks; 3M+3S: 3 meals and 3 snacks; 10S: Grazing diet – 100kcal every 2-3 hours; HCB: High Calorie Breakfast; HCD: High Calorie Dinner; ED: Early Dinner; LD: Late Dinner; FL: Front Loading: EED: Equal Energy Distribution; ML: Middle Loading; BL: Back Loading.

Author Year (Country)	Age	BMI	Intervention	Comorbidity	Duration	Intervention No	Intervention Mean	Intervention SD	Control No	Control Mean	Control SD	Mean Difference, 95%CI	Weight%
**Additional resources were provided**													
Philips 2021 (Switzerland)	40	28	FT=12h	Metabolic	26	23	-3.06	10.22	18	2.16	10.22	-5.23 [-11.53; 1.08]	1.0
Wei 2023 (China)	32	32	FT=8h	Metabolic	52	45	-5.90	17.47	43	-0.80	17.47	-5.10 [-12.40; 2.20]	0.7
Kunduraci & Ozbek 2020 (Turkey)	48	35	FT=8h	Metabolic	12	32	-15.47	37.70	33	-13.12	28.40	-2.35 [-18.61; 13.91]	0.1
Pavlou 2023 (USA)	55	39	FT=8h	Metabolic	26	25	-0.72	1.26	25	0.19	1.26	-0.91 [-1.61; -0.21]	77.9
Liu 2022 (China)	32	32	FT=8h	Healthy	52	69	-3.50	16.54	70	-2.90	16.54	-0.60 [-6.10; 4.90]	1.3
Overall Effect						194			189			-1.00 [-1.68; -0.31]	81.0
Heterogeneity: $I^2 = 0\%$, $\tau^2 = 0$, $\chi^2_4 = 3.05$ ($p = 0.55$)													
**Resources were not provided**													
Che 2021 (China)	48	.	FT=10h	Metabolic	12	60	-26.49	34.86	60	-14.05	29.28	-12.44 [-23.96; -0.92]	0.3
Suthutvoravut 2023 (Thailand)	55	30	FT=9h	Metabolic	12	49	-4.54	7.63	22	0.20	7.63	-4.74 [-8.58; -0.90]	2.6
Lin 2023 (USA)	44	38	FT=8h	Healthy	52	30	2.82	11.76	30	6.26	11.76	-3.44 [-9.39; 2.51]	1.1
Montero 2023 (Spain)	48	.	FT=8h	Metabolic	12	148	-1.92	9.40	49	0.34	9.57	-2.26 [-5.34; 0.82]	4.0
Jamshed 2022 (USA)	43	40	FT=8h	Healthy	14	45	-8.00	16.94	45	-6.00	16.94	-2.00 [-9.00; 5.00]	0.8
Lowe 2020 (USA)	46	33	FT=8h	Healthy	12	22	-1.06	6.74	24	0.29	6.74	-1.35 [-5.25; 2.55]	2.5
Chow 2020 (USA)	46	34	FT=8h	Healthy	12	11	-8.00	8.21	9	-7.00	13.93	-1.00 [-11.31; 9.31]	0.4
Thomas 2022 (USA)	38	34	FT=10h	Healthy	39	18	2.20	4.30	20	3.20	19.30	-1.00 [-9.69; 7.69]	0.5
Manoogian 2022 (USA)	40	28	FT=10h	Healthy	12	70	0.19	6.99	66	0.14	6.99	0.05 [-2.30; 2.40]	6.9
Overall Effect						453			325			-1.87 [-3.36; -0.38]	19.0
Heterogeneity: $I^2 = 5\%$, $\tau^2 = 0.2654$, $\chi^2_8 = 8.4$ ($p = 0.40$)													
**Overall Effect**						**647**			**514**			**-1.15 [-1.77; -0.53]**	**100.0**
Heterogeneity: $I^2 = 0\%$, $\tau^2 = 0$, $\chi^2_{13} = 12.43$ ($p = 0.49$)													
Test for overall effect: $z = -3.64$ ($p < 0.01$)													
Test for subgroup differences: $\chi^2_1 = 1.08$, df = 1 ($p = 0.30$)													

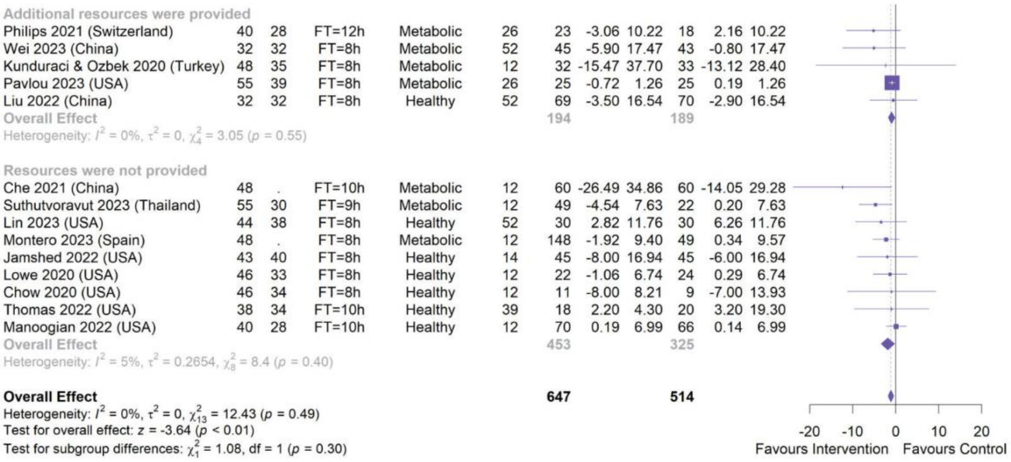

eFigure 152: Meta-analysis of difference in mean difference (95% CIs) for the effect of time-restricted eating on fasting glucose (mg/dL), grouped by resource provision

BMI: Body Mass Index; SD: Standard Deviation; CI: Confidence Interval; USA: United States of America; FT: Feeding Time; Duration: follow-up duration in weeks; 2M/3M/6M: 2, 3 or 6 meals; 3M+2S: 3 meals and 2 snacks; 3M+3S: 3 meals and 3 snacks; 10S: Grazing diet – 100kcal every 2-3 hours; HCB: High Calorie Breakfast; HCD: High Calorie Dinner; ED: Early Dinner; LD: Late Dinner; FL: Front Loading: EED: Equal Energy Distribution; ML: Middle Loading; BL: Back Loading.

© 2024 Liu HY et al. *JAMA Network Open.*

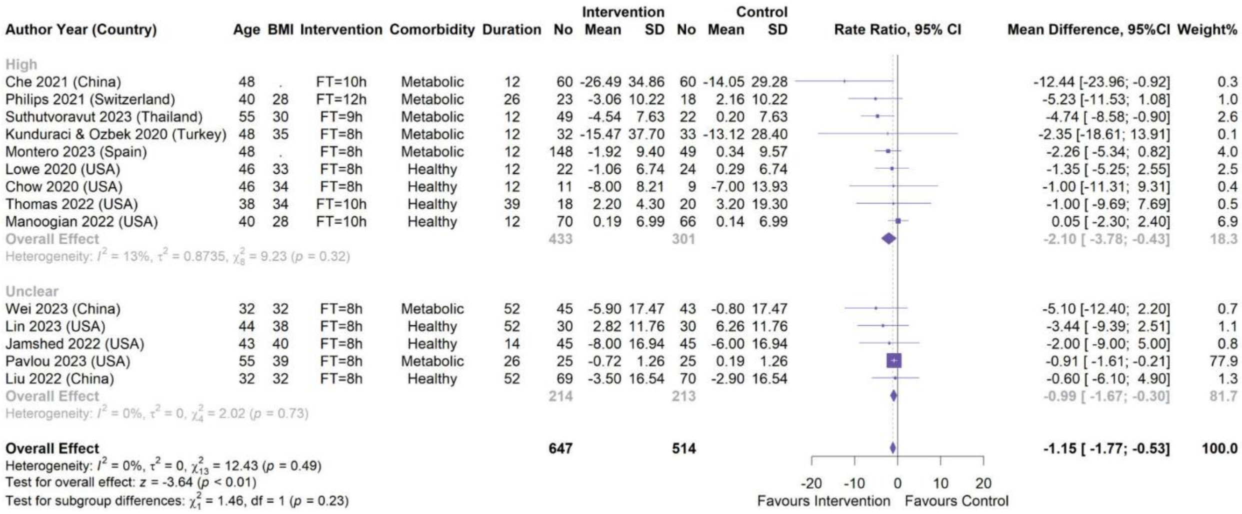

eFigure 153: Meta-analysis of difference in mean difference (95% CIs) for the effect of time-restricted eating on fasting glucose (mg/dL), grouped by risk of bias

BMI: Body Mass Index; SD: Standard Deviation; CI: Confidence Interval; USA: United States of America; FT: Feeding Time; Duration: follow-up duration in weeks; 2M/3M/6M: 2, 3 or 6 meals; 3M+2S: 3 meals and 2 snacks; 3M+3S: 3 meals and 3 snacks; 10S: Grazing diet – 100kcal every 2-3 hours; HCB: High Calorie Breakfast; HCD: High Calorie Dinner; ED: Early Dinner; LD: Late Dinner; FL: Front Loading: EED: Equal Energy Distribution; ML: Middle Loading; BL: Back Loading.

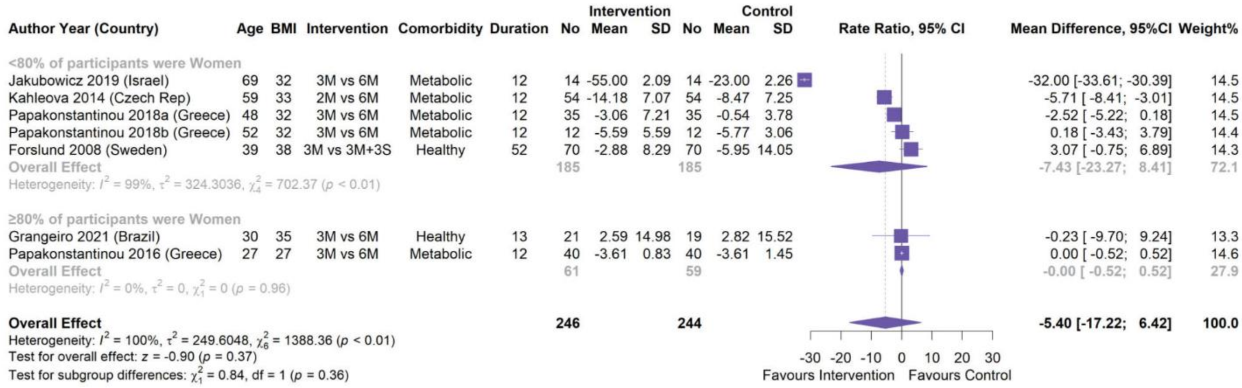

eFigure 154: Meta-analysis of difference in mean difference (95% CIs) for the effect of meal frequency on fasting glucose (mg/dL), grouped by gender proportion

BMI: Body Mass Index; SD: Standard Deviation; CI: Confidence Interval; USA: United States of America; FT: Feeding Time; Duration: follow-up duration in weeks; 2M/3M/6M: 2, 3 or 6 meals; 3M+2S: 3 meals and 2 snacks; 3M+3S: 3 meals and 3 snacks; 10S: Grazing diet – 100kcal every 2-3 hours; HCB: High Calorie Breakfast; HCD: High Calorie Dinner; ED: Early Dinner; LD: Late Dinner; FL: Front Loading: EED: Equal Energy Distribution; ML: Middle Loading; BL: Back Loading.

© 2024 Liu HY et al. *JAMA Network Open.*

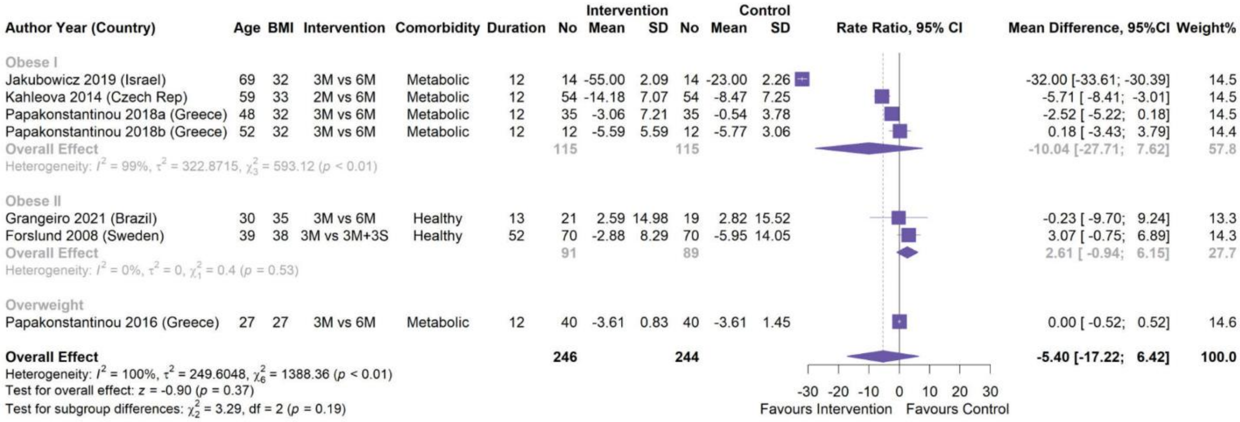

eFigure 155: Meta-analysis of difference in mean difference (95% CIs) for the effect of meal frequency on fasting glucose (mg/dL), grouped by baseline BMI status

BMI: Body Mass Index; SD: Standard Deviation; CI: Confidence Interval; USA: United States of America; FT: Feeding Time; Duration: follow-up duration in weeks; 2M/3M/6M: 2, 3 or 6 meals; 3M+2S: 3 meals and 2 snacks; 3M+3S: 3 meals and 3 snacks; 10S: Grazing diet – 100kcal every 2-3 hours; HCB: High Calorie Breakfast; HCD: High Calorie Dinner; ED: Early Dinner; LD: Late Dinner; FL: Front Loading: EED: Equal Energy Distribution; ML: Middle Loading; BL: Back Loading.

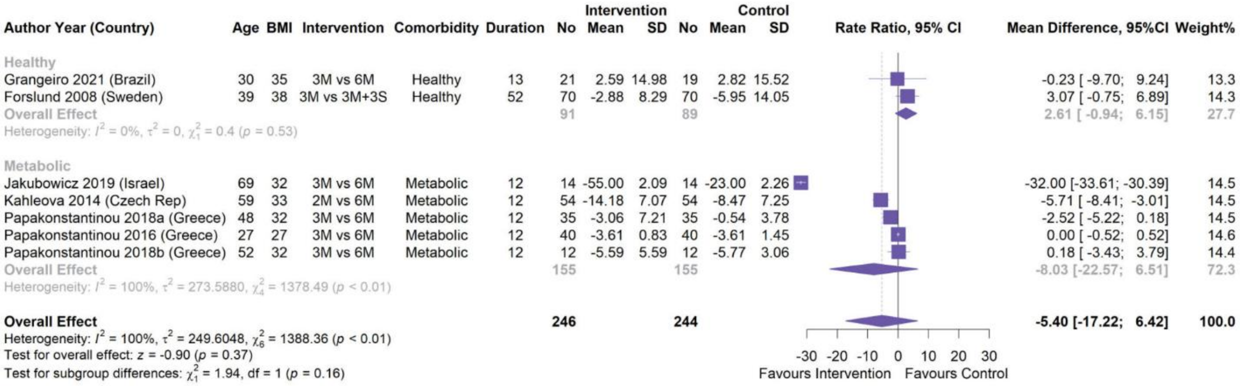

eFigure 156: Meta-analysis of difference in mean difference (95% CIs) for the effect of meal frequency on fasting glucose (mg/dL), grouped by health status

BMI: Body Mass Index; SD: Standard Deviation; CI: Confidence Interval; USA: United States of America; FT: Feeding Time; Duration: follow-up duration in weeks; 2M/3M/6M: 2, 3 or 6 meals; 3M+2S: 3 meals and 2 snacks; 3M+3S: 3 meals and 3 snacks; 10S: Grazing diet – 100kcal every 2-3 hours; HCB: High Calorie Breakfast; HCD: High Calorie Dinner; ED: Early Dinner; LD: Late Dinner; FL: Front Loading: EED: Equal Energy Distribution; ML: Middle Loading; BL: Back Loading.

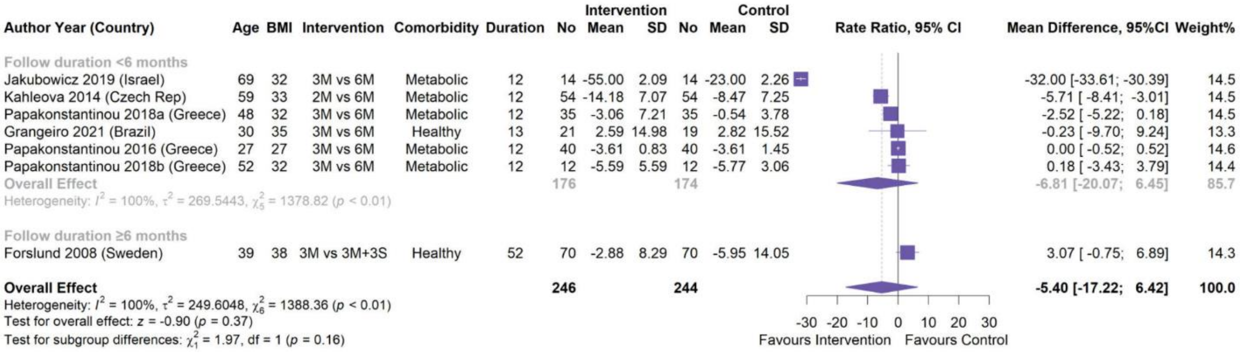

eFigure 157: Meta-analysis of difference in mean difference (95% CIs) for the effect of meal frequency on fasting glucose (mg/dL), grouped by follow duration

BMI: Body Mass Index; SD: Standard Deviation; CI: Confidence Interval; USA: United States of America; FT: Feeding Time; Duration: follow-up duration in weeks; 2M/3M/6M: 2, 3 or 6 meals; 3M+2S: 3 meals and 2 snacks; 3M+3S: 3 meals and 3 snacks; 10S: Grazing diet

– 100kcal every 2-3 hours; HCB: High Calorie Breakfast; HCD: High Calorie Dinner; ED: Early Dinner; LD: Late Dinner; FL: Front Loading: EED: Equal Energy Distribution; ML: Middle Loading; BL: Back Loading.

Author Year (Country)	Age	BMI	Intervention	Comorbidity	Duration	Intervention No	Intervention Mean	Intervention SD	Control No	Control Mean	Control SD	Mean Difference, 95%CI	Weight%
2M vs 6M													
Kahleova 2014 (Czech Rep)	59	33	2M vs 6M	Metabolic	12	54	-14.18	7.07	54	-8.47	7.25	-5.71 [-8.41; -3.01]	14.5
3M vs 3M+3S													
Forslund 2008 (Sweden)	39	38	3M vs 3M+3S	Healthy	52	70	-2.88	8.29	70	-5.95	14.05	3.07 [-0.75; 6.89]	14.3
3M vs 6M													
Jakubowicz 2019 (Israel)	69	32	3M vs 6M	Metabolic	12	14	-55.00	2.09	14	-23.00	2.26	-32.00 [-33.61; -30.39]	14.5
Papakonstantinou 2018a (Greece)	48	32	3M vs 6M	Metabolic	12	35	-3.06	7.21	35	-0.54	3.78	-2.52 [-5.22; 0.18]	14.5
Grangeiro 2021 (Brazil)	30	35	3M vs 6M	Healthy	13	21	2.59	14.98	19	2.82	15.52	-0.23 [-9.70; 9.24]	13.3
Papakonstantinou 2016 (Greece)	27	27	3M vs 6M	Metabolic	12	40	-3.61	0.83	40	-3.61	1.45	0.00 [-0.52; 0.52]	14.6
Papakonstantinou 2018b (Greece)	52	32	3M vs 6M	Metabolic	12	12	-5.59	5.59	12	-5.77	3.06	0.18 [-3.43; 3.79]	14.4
Overall Effect						122			120			-7.01 [-22.98; 8.95]	71.2
Heterogeneity: $I^2 = 100\%$, $\tau^2 = 325.9796$, $\chi^2_4 = 1374.83$ ($p < 0.01$)													
**Overall Effect**						**246**			**244**			**-5.40 [-17.22; 6.42]**	**100.0**

Heterogeneity: $I^2 = 100\%$, $\tau^2 = 249.6048$, $\chi^2_6 = 1388.36$ ($p < 0.01$)
Test for overall effect: $z = -0.90$ ($p = 0.37$)
Test for subgroup differences: $\chi^2_2 = 13.78$, df = 2 ($p < 0.01$)

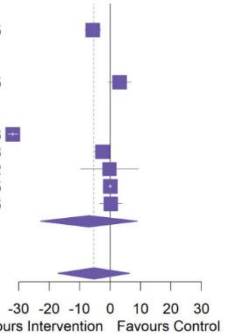

eFigure 158: Meta-analysis of difference in mean difference (95% CIs) for the effect of meal frequency on fasting glucose (mg/dL), grouped by intervention intensity

BMI: Body Mass Index; SD: Standard Deviation; CI: Confidence Interval; USA: United States of America; FT: Feeding Time; Duration: follow-up duration in weeks; 2M/3M/6M: 2, 3 or 6 meals; 3M+2S: 3 meals and 2 snacks; 3M+3S: 3 meals and 3 snacks; 10S: Grazing diet – 100kcal every 2-3 hours; HCB: High Calorie Breakfast; HCD: High Calorie Dinner; ED: Early Dinner; LD: Late Dinner; FL: Front Loading: EED: Equal Energy Distribution; ML: Middle Loading; BL: Back Loading.

Author Year (Country)	Age	BMI	Intervention	Comorbidity	Duration	Intervention No	Intervention Mean	Intervention SD	Control No	Control Mean	Control SD	Mean Difference, 95%CI	Weight%
Intervention involved < 1 session per week													
Papakonstantinou 2018a (Greece)	48	32	3M vs 6M	Metabolic	12	35	-3.06	7.21	35	-0.54	3.78	-2.52 [-5.22; 0.18]	14.5
Papakonstantinou 2018b (Greece)	52	32	3M vs 6M	Metabolic	12	12	-5.59	5.59	12	-5.77	3.06	0.18 [-3.43; 3.79]	14.4
Forslund 2008 (Sweden)	39	38	3M vs 3M+3S	Healthy	52	70	-2.88	8.29	70	-5.95	14.05	3.07 [-0.75; 6.89]	14.3
Overall Effect						117			117			0.02 [-3.23; 3.27]	43.2
Heterogeneity: $I^2 = 65\%$, $\tau^2 = 5.3051$, $\chi^2_2 = 5.64$ ($p = 0.06$)													
Intervention involved ≥ 1 session per week													
Jakubowicz 2019 (Israel)	69	32	3M vs 6M	Metabolic	12	14	-55.00	2.09	14	-23.00	2.26	-32.00 [-33.61; -30.39]	14.5
Kahleova 2014 (Czech Rep)	59	33	2M vs 6M	Metabolic	12	54	-14.18	7.07	54	-8.47	7.25	-5.71 [-8.41; -3.01]	14.5
Grangeiro 2021 (Brazil)	30	35	3M vs 6M	Healthy	13	21	2.59	14.98	19	2.82	15.52	-0.23 [-9.70; 9.24]	13.3
Papakonstantinou 2016 (Greece)	27	27	3M vs 6M	Metabolic	12	40	-3.61	0.83	40	-3.61	1.45	0.00 [-0.52; 0.52]	14.6
Overall Effect						129			127			-9.62 [-28.69; 9.45]	56.8
Heterogeneity: $I^2 = 100\%$, $\tau^2 = 372.2998$, $\chi^2_3 = 1375.62$ ($p < 0.01$)													
**Overall Effect**						**246**			**244**			**-5.40 [-17.22; 6.42]**	**100.0**

Heterogeneity: $I^2 = 100\%$, $\tau^2 = 249.6048$, $\chi^2_6 = 1388.36$ ($p < 0.01$)
Test for overall effect: $z = -0.90$ ($p = 0.37$)
Test for subgroup differences: $\chi^2_1 = 0.95$, df = 1 ($p = 0.33$)

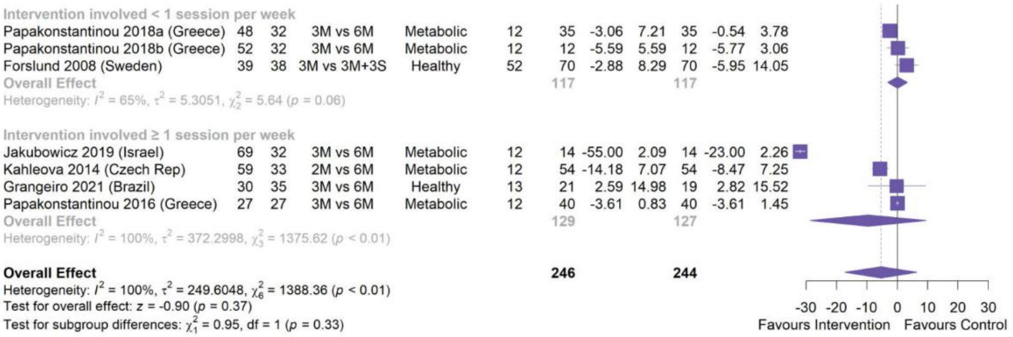

eFigure 159: Meta-analysis of difference in mean difference (95% CIs) for the effect of meal frequency on fasting glucose (mg/dL), grouped by frequency of contact

BMI: Body Mass Index; SD: Standard Deviation; CI: Confidence Interval; USA: United States of America; FT: Feeding Time; Duration: follow-up duration in weeks; 2M/3M/6M: 2, 3 or 6 meals; 3M+2S: 3 meals and 2 snacks; 3M+3S: 3 meals and 3 snacks; 10S: Grazing diet – 100kcal every 2-3 hours; HCB: High Calorie Breakfast; HCD: High Calorie Dinner; ED: Early Dinner; LD: Late Dinner; FL: Front Loading: EED: Equal Energy Distribution; ML: Middle Loading; BL: Back Loading.

Author Year (Country)	Age	BMI	Intervention	Comorbidity	Duration	Intervention No	Intervention Mean	Intervention SD	Control No	Control Mean	Control SD	Mean Difference, 95%CI	Weight%
Any health professional													
Jakubowicz 2019 (Israel)	69	32	3M vs 6M	Metabolic	12	14	-55.00	2.09	14	-23.00	2.26	-32.00 [-33.61; -30.39]	14.5
Kahleova 2014 (Czech Rep)	59	33	2M vs 6M	Metabolic	12	54	-14.18	7.07	54	-8.47	7.25	-5.71 [-8.41; -3.01]	14.5
Papakonstantinou 2018a (Greece)	48	32	3M vs 6M	Metabolic	12	35	-3.06	7.21	35	-0.54	3.78	-2.52 [-5.22; 0.18]	14.5
Papakonstantinou 2018b (Greece)	52	32	3M vs 6M	Metabolic	12	12	-5.59	5.59	12	-5.77	3.06	0.18 [-3.43; 3.79]	14.4
Overall Effect						115			115			-10.04 [-27.71; 7.62]	57.8
Heterogeneity: $I^2 = 99\%$, $\tau^2 = 322.8715$, $\chi^2_3 = 593.12$ ($p < 0.01$)													
Trained specifically in nutrition													
Grangeiro 2021 (Brazil)	30	35	3M vs 6M	Healthy	13	21	2.59	14.98	19	2.82	15.52	-0.23 [-9.70; 9.24]	13.3
Papakonstantinou 2016 (Greece)	27	27	3M vs 6M	Metabolic	12	40	-3.61	0.83	40	-3.61	1.45	0.00 [-0.52; 0.52]	14.6
Forslund 2008 (Sweden)	39	38	3M vs 3M+3S	Healthy	52	70	-2.88	8.29	70	-5.95	14.05	3.07 [-0.75; 6.89]	14.3
Overall Effect						131			129			0.44 [-1.15; 2.03]	42.2
Heterogeneity: $I^2 = 18\%$, $\tau^2 = 0.7292$, $\chi^2_2 = 2.44$ ($p = 0.30$)													
**Overall Effect**						**246**			**244**			**-5.40 [-17.22; 6.42]**	**100.0**

Heterogeneity: $I^2 = 100\%$, $\tau^2 = 249.6048$, $\chi^2_6 = 1388.36$ ($p < 0.01$)
Test for overall effect: $z = -0.90$ ($p = 0.37$)
Test for subgroup differences: $\chi^2_1 = 1.34$, df = 1 ($p = 0.25$)

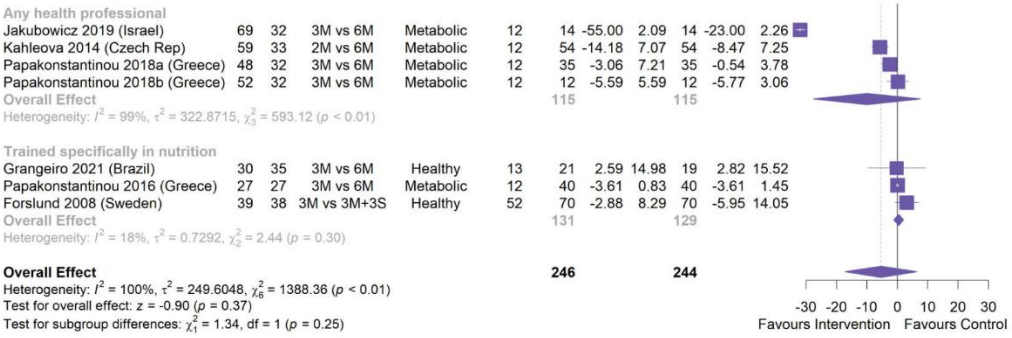

eFigure 160: Meta-analysis of difference in mean difference (95% CIs) for the effect of meal frequency on fasting glucose (mg/dL), grouped by delivery personnel

© 2024 Liu HY et al. *JAMA Network Open.*

BMI: Body Mass Index; SD: Standard Deviation; CI: Confidence Interval; USA: United States of America; FT: Feeding Time; Duration: follow-up duration in weeks; 2M/3M/6M: 2, 3 or 6 meals; 3M+2S: 3 meals and 2 snacks; 3M+3S: 3 meals and 3 snacks; 10S: Grazing diet – 100kcal every 2-3 hours; HCB: High Calorie Breakfast; HCD: High Calorie Dinner; ED: Early Dinner; LD: Late Dinner; FL: Front Loading: EED: Equal Energy Distribution; ML: Middle Loading; BL: Back Loading.

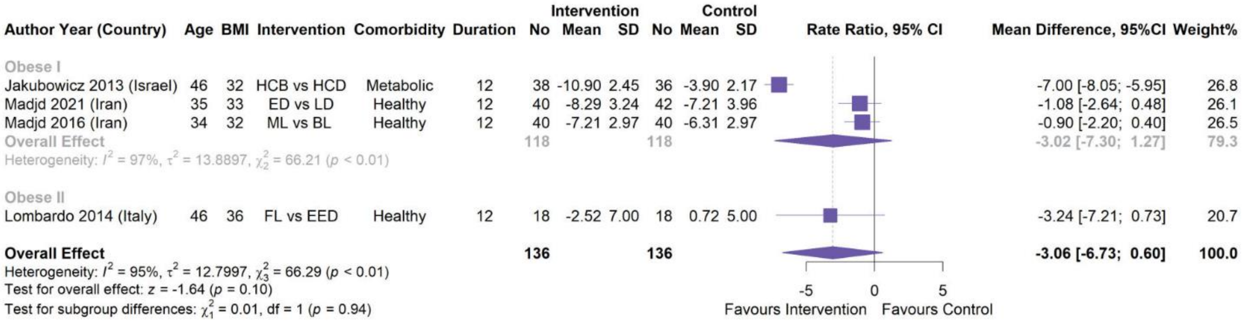

eFigure 161: Meta-analysis of difference in mean difference (95% CIs) for the effect of meal distribution on fasting glucose (mg/dL), grouped by baseline BMI status

BMI: Body Mass Index; SD: Standard Deviation; CI: Confidence Interval; USA: United States of America; FT: Feeding Time; Duration: follow-up duration in weeks; 2M/3M/6M: 2, 3 or 6 meals; 3M+2S: 3 meals and 2 snacks; 3M+3S: 3 meals and 3 snacks; 10S: Grazing diet – 100kcal every 2-3 hours; HCB: High Calorie Breakfast; HCD: High Calorie Dinner; ED: Early Dinner; LD: Late Dinner; FL: Front Loading: EED: Equal Energy Distribution; ML: Middle Loading; BL: Back Loading.

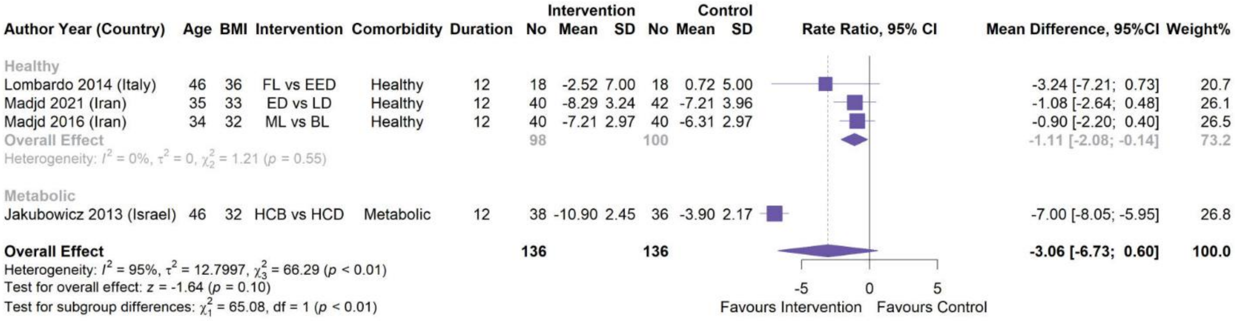

eFigure 162: Meta-analysis of difference in mean difference (95% CIs) for the effect of meal distribution on fasting glucose (mg/dL), grouped by health status

BMI: Body Mass Index; SD: Standard Deviation; CI: Confidence Interval; USA: United States of America; FT: Feeding Time; Duration: follow-up duration in weeks; 2M/3M/6M: 2, 3 or 6 meals; 3M+2S: 3 meals and 2 snacks; 3M+3S: 3 meals and 3 snacks; 10S: Grazing diet – 100kcal every 2-3 hours; HCB: High Calorie Breakfast; HCD: High Calorie Dinner; ED: Early Dinner; LD: Late Dinner; FL: Front Loading: EED: Equal Energy Distribution; ML: Middle Loading; BL: Back Loading.

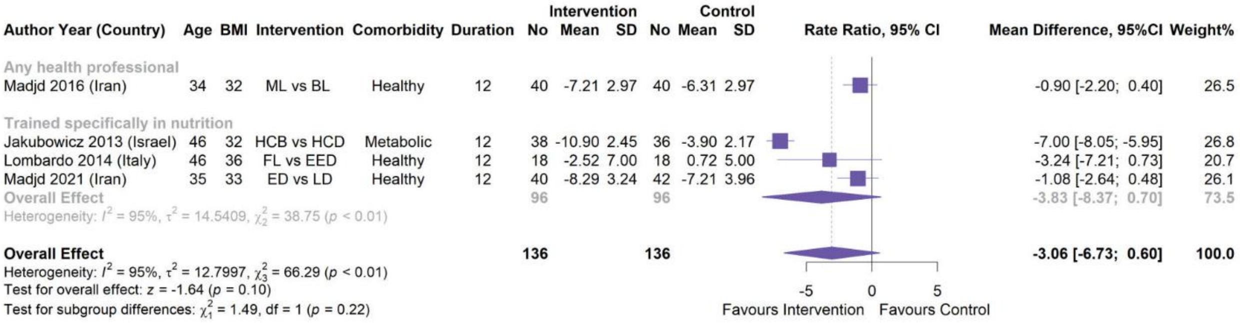

eFigure 163: Meta-analysis of difference in mean difference (95% CIs) for the effect of meal distribution on fasting glucose (mg/dL), grouped by delivery personnel

BMI: Body Mass Index; SD: Standard Deviation; CI: Confidence Interval; USA: United States of America; FT: Feeding Time; Duration: follow-up duration in weeks; 2M/3M/6M: 2, 3 or 6 meals; 3M+2S: 3 meals and 2 snacks; 3M+3S: 3 meals and 3 snacks; 10S: Grazing diet – 100kcal every 2-3 hours; HCB: High Calorie Breakfast; HCD: High Calorie Dinner; ED: Early Dinner; LD: Late Dinner; FL: Front Loading: EED: Equal Energy Distribution; ML: Middle Loading; BL: Back Loading.

© 2024 Liu HY et al. *JAMA Network Open.*

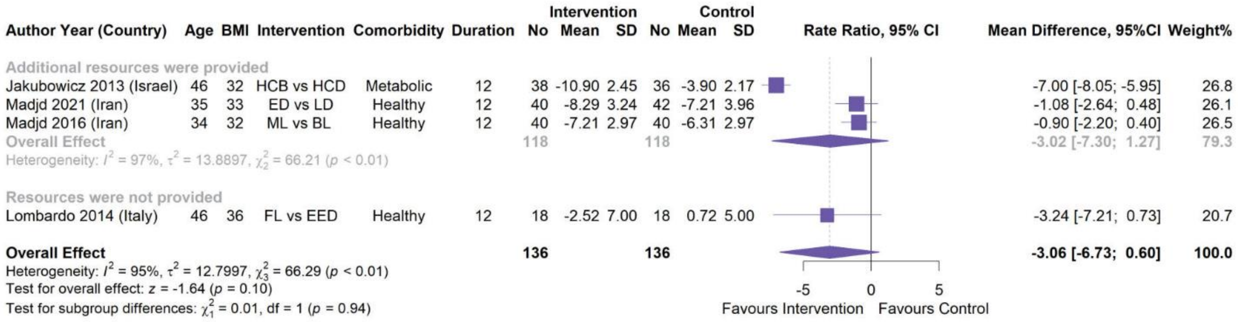

eFigure 164: Meta-analysis of difference in mean difference (95% CIs) for the effect of meal distribution on fasting glucose (mg/dL), grouped by resource provision

BMI: Body Mass Index; SD: Standard Deviation; CI: Confidence Interval; USA: United States of America; FT: Feeding Time; Duration: follow-up duration in weeks; 2M/3M/6M: 2, 3 or 6 meals; 3M+2S: 3 meals and 2 snacks; 3M+3S: 3 meals and 3 snacks; 10S: Grazing diet – 100kcal every 2-3 hours; HCB: High Calorie Breakfast; HCD: High Calorie Dinner; ED: Early Dinner; LD: Late Dinner; FL: Front Loading: EED: Equal Energy Distribution; ML: Middle Loading; BL: Back Loading.

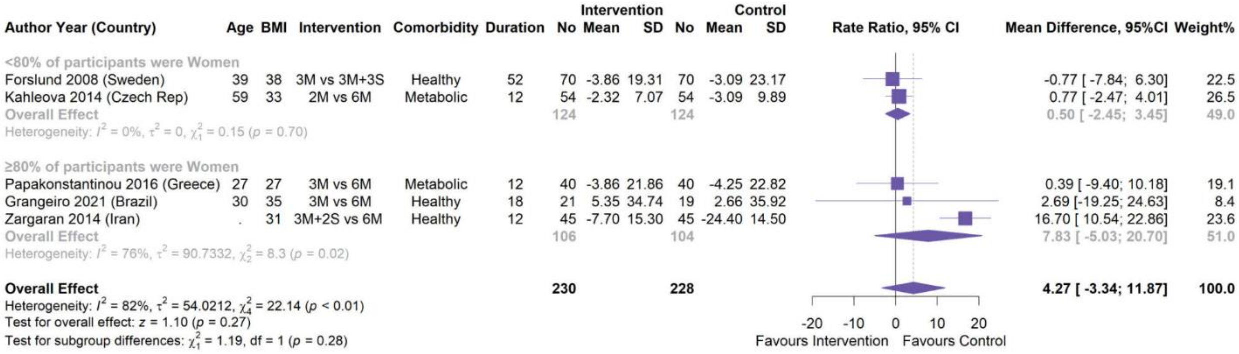

eFigure 165: Meta-analysis of difference in mean difference (95% CIs) for the effect of meal frequency on LDL (mg/dL), grouped by gender proportion

BMI: Body Mass Index; SD: Standard Deviation; CI: Confidence Interval; USA: United States of America; FT: Feeding Time; Duration: follow-up duration in weeks; 2M/3M/6M: 2, 3 or 6 meals; 3M+2S: 3 meals and 2 snacks; 3M+3S: 3 meals and 3 snacks; 10S: Grazing diet – 100kcal every 2-3 hours; HCB: High Calorie Breakfast; HCD: High Calorie Dinner; ED: Early Dinner; LD: Late Dinner; FL: Front Loading: EED: Equal Energy Distribution; ML: Middle Loading; BL: Back Loading.

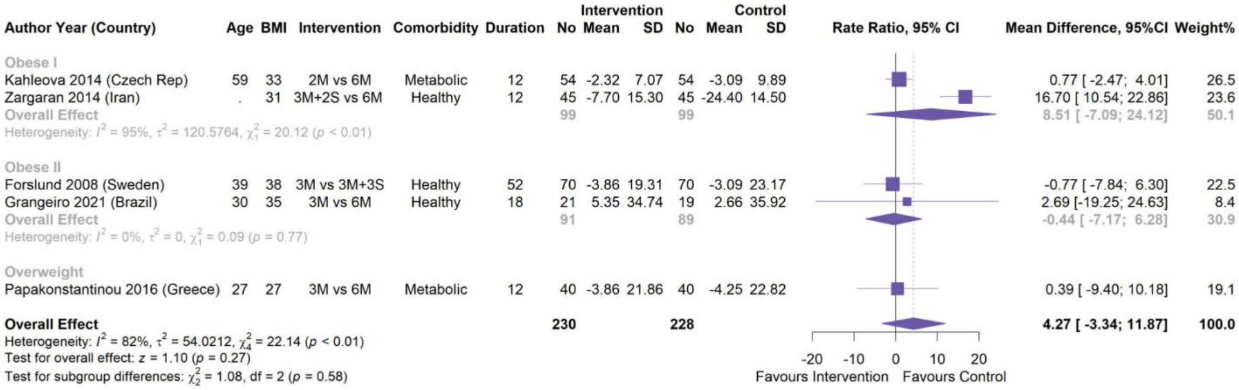

eFigure 166: Meta-analysis of difference in mean difference (95% CIs) for the effect of meal frequency on LDL (mg/dL), grouped by baseline BMI status

BMI: Body Mass Index; SD: Standard Deviation; CI: Confidence Interval; USA: United States of America; FT: Feeding Time; Duration: follow-up duration in weeks; 2M/3M/6M: 2, 3 or 6 meals; 3M+2S: 3 meals and 2 snacks; 3M+3S: 3 meals and 3 snacks; 10S: Grazing diet – 100kcal every 2-3 hours; HCB: High Calorie Breakfast; HCD: High Calorie Dinner; ED: Early Dinner; LD: Late Dinner; FL: Front Loading: EED: Equal Energy Distribution; ML: Middle Loading; BL: Back Loading.

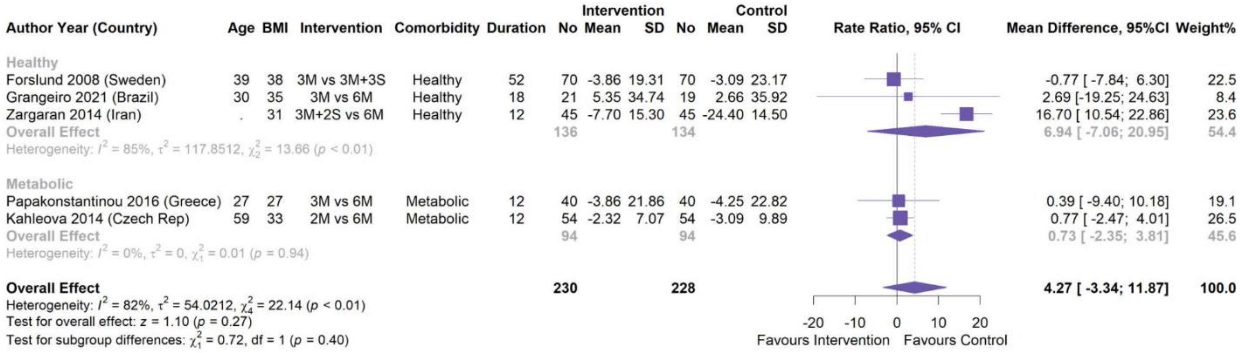

eFigure 167: Meta-analysis of difference in mean difference (95% CIs) for the effect of meal frequency on LDL (mg/dL), grouped by health status

BMI: Body Mass Index; SD: Standard Deviation; CI: Confidence Interval; USA: United States of America; FT: Feeding Time; Duration: follow-up duration in weeks; 2M/3M/6M: 2, 3 or 6 meals; 3M+2S: 3 meals and 2 snacks; 3M+3S: 3 meals and 3 snacks; 10S: Grazing diet – 100kcal every 2-3 hours; HCB: High Calorie Breakfast; HCD: High Calorie Dinner; ED: Early Dinner; LD: Late Dinner; FL: Front Loading: EED: Equal Energy Distribution; ML: Middle Loading; BL: Back Loading.

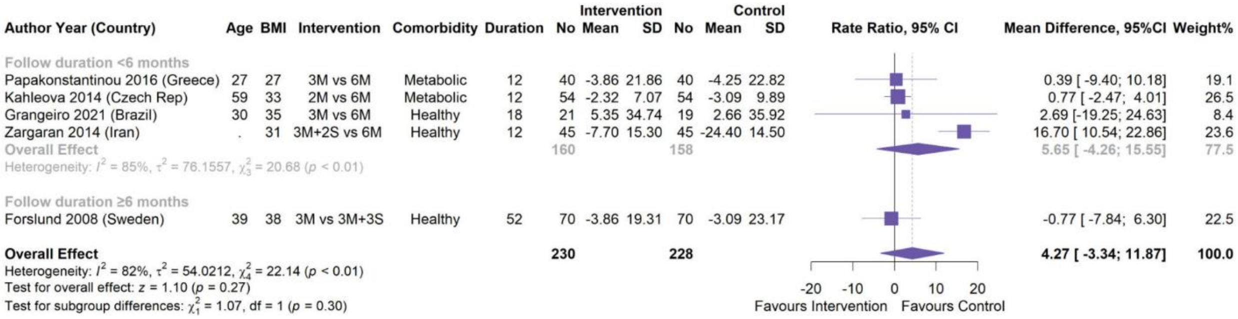

eFigure 168: Meta-analysis of difference in mean difference (95% CIs) for the effect of meal frequency on LDL (mg/dL), grouped by follow duration

BMI: Body Mass Index; SD: Standard Deviation; CI: Confidence Interval; USA: United States of America; FT: Feeding Time; Duration: follow-up duration in weeks; 2M/3M/6M: 2, 3 or 6 meals; 3M+2S: 3 meals and 2 snacks; 3M+3S: 3 meals and 3 snacks; 10S: Grazing diet – 100kcal every 2-3 hours; HCB: High Calorie Breakfast; HCD: High Calorie Dinner; ED: Early Dinner; LD: Late Dinner; FL: Front Loading: EED: Equal Energy Distribution; ML: Middle Loading; BL: Back Loading.

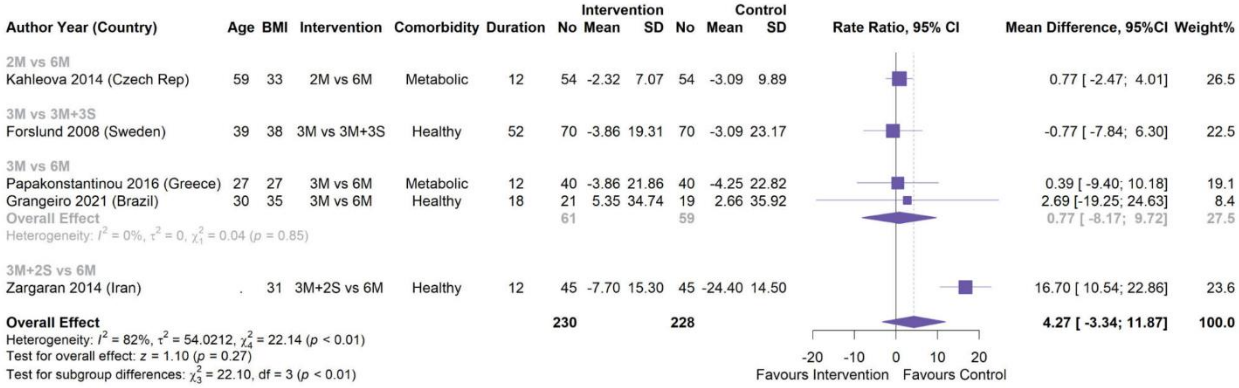

eFigure 169: Meta-analysis of difference in mean difference (95% CIs) for the effect of meal frequency on LDL (mg/dL), grouped by intervention intensity

BMI: Body Mass Index; SD: Standard Deviation; CI: Confidence Interval; USA: United States of America; FT: Feeding Time; Duration: follow-up duration in weeks; 2M/3M/6M: 2, 3 or 6 meals; 3M+2S: 3 meals and 2 snacks; 3M+3S: 3 meals and 3 snacks; 10S: Grazing diet – 100kcal every 2-3 hours; HCB: High Calorie Breakfast; HCD: High Calorie Dinner; ED: Early Dinner; LD: Late Dinner; FL: Front Loading: EED: Equal Energy Distribution; ML: Middle Loading; BL: Back Loading.

© 2024 Liu HY et al. *JAMA Network Open.*

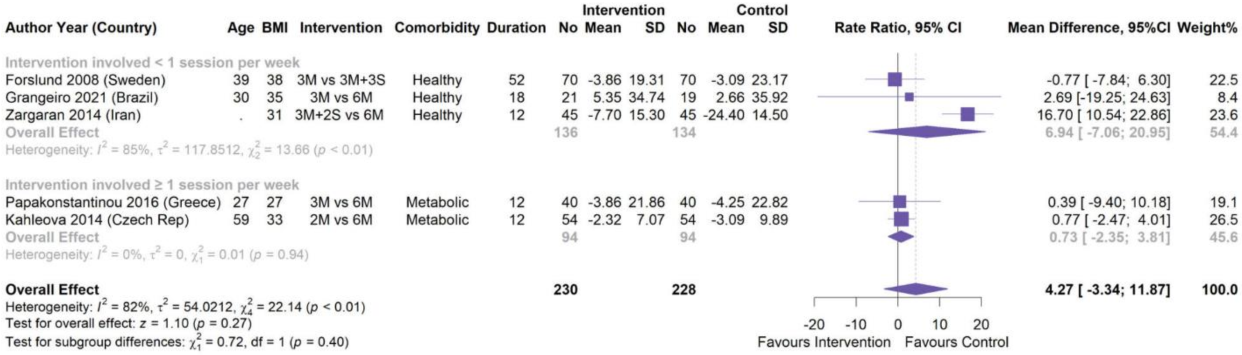

eFigure 170: Meta-analysis of difference in mean difference (95% CIs) for the effect of meal frequency on LDL (mg/dL), grouped by frequency of contact

BMI: Body Mass Index; SD: Standard Deviation; CI: Confidence Interval; USA: United States of America; FT: Feeding Time; Duration: follow-up duration in weeks; 2M/3M/6M: 2, 3 or 6 meals; 3M+2S: 3 meals and 2 snacks; 3M+3S: 3 meals and 3 snacks; 10S: Grazing diet – 100kcal every 2-3 hours; HCB: High Calorie Breakfast; HCD: High Calorie Dinner; ED: Early Dinner; LD: Late Dinner; FL: Front Loading: EED: Equal Energy Distribution; ML: Middle Loading; BL: Back Loading.

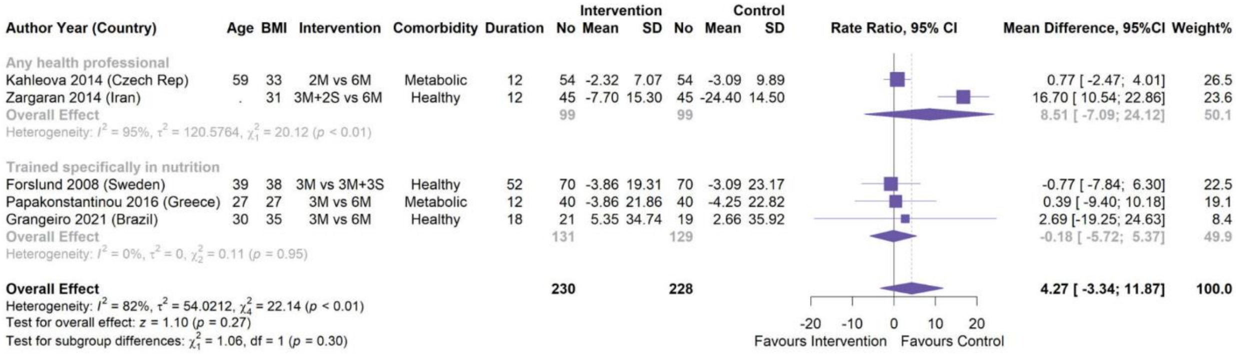

eFigure 171: Meta-analysis of difference in mean difference (95% CIs) for the effect of meal frequency on LDL (mg/dL), grouped by delivery personnel

BMI: Body Mass Index; SD: Standard Deviation; CI: Confidence Interval; USA: United States of America; FT: Feeding Time; Duration: follow-up duration in weeks; 2M/3M/6M: 2, 3 or 6 meals; 3M+2S: 3 meals and 2 snacks; 3M+3S: 3 meals and 3 snacks; 10S: Grazing diet – 100kcal every 2-3 hours; HCB: High Calorie Breakfast; HCD: High Calorie Dinner; ED: Early Dinner; LD: Late Dinner; FL: Front Loading: EED: Equal Energy Distribution; ML: Middle Loading; BL: Back Loading.

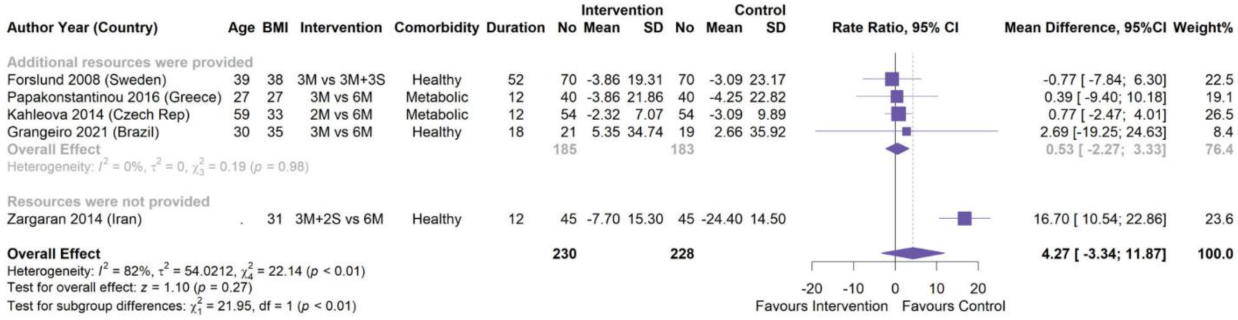

eFigure 172: Meta-analysis of difference in mean difference (95% CIs) for the effect of meal frequency on LDL (mg/dL), grouped by resource provision

BMI: Body Mass Index; SD: Standard Deviation; CI: Confidence Interval; USA: United States of America; FT: Feeding Time; Duration: follow-up duration in weeks; 2M/3M/6M: 2, 3 or 6 meals; 3M+2S: 3 meals and 2 snacks; 3M+3S: 3 meals and 3 snacks; 10S: Grazing diet – 100kcal every 2-3 hours; HCB: High Calorie Breakfast; HCD: High Calorie Dinner; ED: Early Dinner; LD: Late Dinner; FL: Front Loading: EED: Equal Energy Distribution; ML: Middle Loading; BL: Back Loading.

© 2024 Liu HY et al. *JAMA Network Open.*

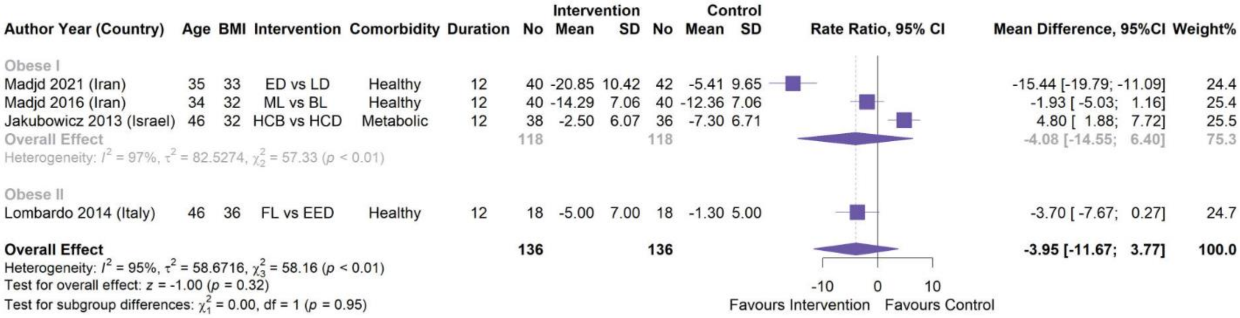

eFigure 173: Meta-analysis of difference in mean difference (95% CIs) for the effect of meal distribution on LDL (mg/dL), grouped by baseline BMI status

BMI: Body Mass Index; SD: Standard Deviation; CI: Confidence Interval; USA: United States of America; FT: Feeding Time; Duration: follow-up duration in weeks; 2M/3M/6M: 2, 3 or 6 meals; 3M+2S: 3 meals and 2 snacks; 3M+3S: 3 meals and 3 snacks; 10S: Grazing diet – 100kcal every 2-3 hours; HCB: High Calorie Breakfast; HCD: High Calorie Dinner; ED: Early Dinner; LD: Late Dinner; FL: Front Loading: EED: Equal Energy Distribution; ML: Middle Loading; BL: Back Loading.

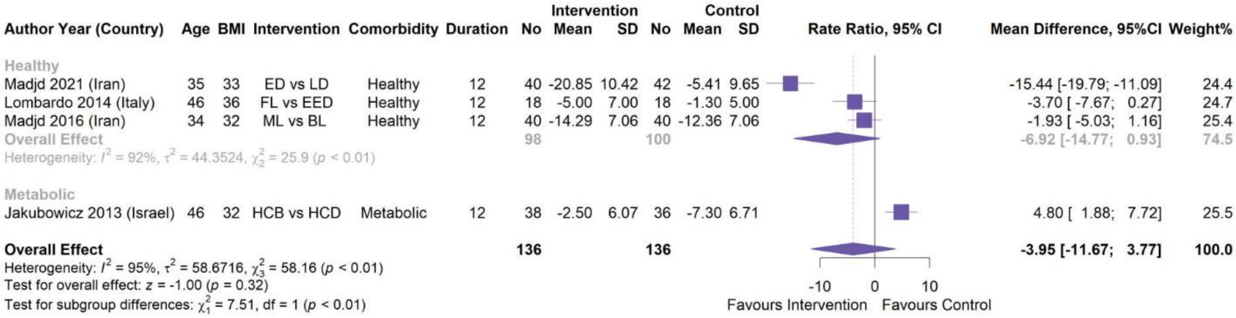

eFigure 174: Meta-analysis of difference in mean difference (95% CIs) for the effect of meal distribution on LDL (mg/dL), grouped by health status

BMI: Body Mass Index; SD: Standard Deviation; CI: Confidence Interval; USA: United States of America; FT: Feeding Time; Duration: follow-up duration in weeks; 2M/3M/6M: 2, 3 or 6 meals; 3M+2S: 3 meals and 2 snacks; 3M+3S: 3 meals and 3 snacks; 10S: Grazing diet – 100kcal every 2-3 hours; HCB: High Calorie Breakfast; HCD: High Calorie Dinner; ED: Early Dinner; LD: Late Dinner; FL: Front Loading: EED: Equal Energy Distribution; ML: Middle Loading; BL: Back Loading.

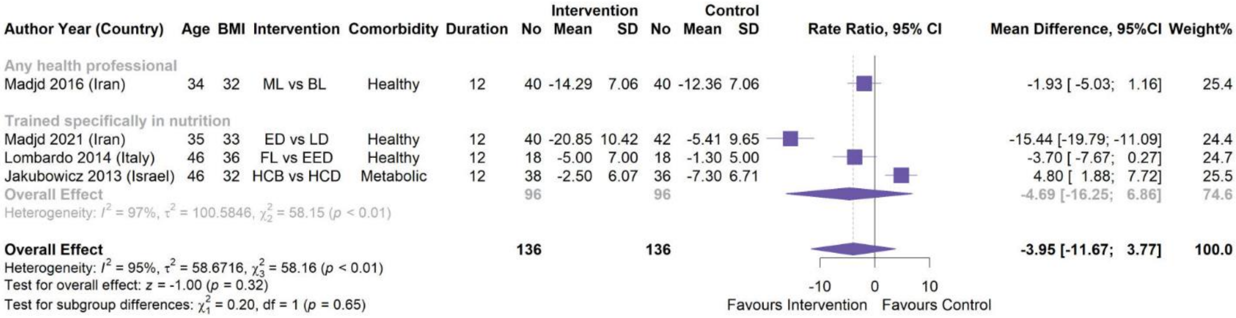

eFigure 175: Meta-analysis of difference in mean difference (95% CIs) for the effect of meal distribution on LDL (mg/dL), grouped by delivery personnel

BMI: Body Mass Index; SD: Standard Deviation; CI: Confidence Interval; USA: United States of America; FT: Feeding Time; Duration: follow-up duration in weeks; 2M/3M/6M: 2, 3 or 6 meals; 3M+2S: 3 meals and 2 snacks; 3M+3S: 3 meals and 3 snacks; 10S: Grazing diet – 100kcal every 2-3 hours; HCB: High Calorie Breakfast; HCD: High Calorie Dinner; ED: Early Dinner; LD: Late Dinner; FL: Front Loading: EED: Equal Energy Distribution; ML: Middle Loading; BL: Back Loading.

© 2024 Liu HY et al. *JAMA Network Open.*

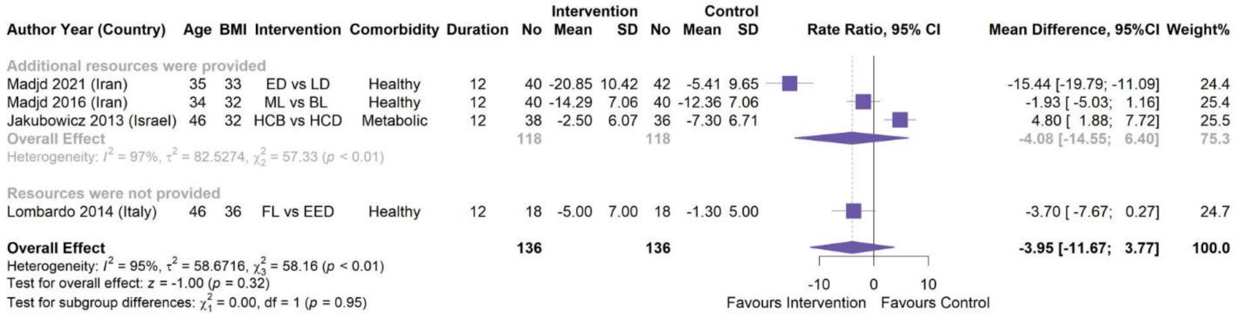

eFigure 176: Meta-analysis of difference in mean difference (95% CIs) for the effect of meal distribution on LDL (mg/dL), grouped by resource provision

BMI: Body Mass Index; SD: Standard Deviation; CI: Confidence Interval; USA: United States of America; FT: Feeding Time; Duration: follow-up duration in weeks; 2M/3M/6M: 2, 3 or 6 meals; 3M+2S: 3 meals and 2 snacks; 3M+3S: 3 meals and 3 snacks; 10S: Grazing diet – 100kcal every 2-3 hours; HCB: High Calorie Breakfast; HCD: High Calorie Dinner; ED: Early Dinner; LD: Late Dinner; FL: Front Loading: EED: Equal Energy Distribution; ML: Middle Loading; BL: Back Loading.

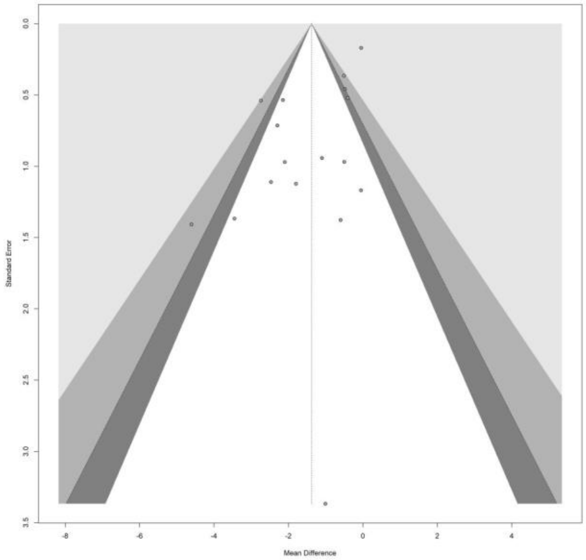

eFigure 177: Funnel plots for weight (TRE vs control)

© 2024 Liu HY et al. *JAMA Network Open.*

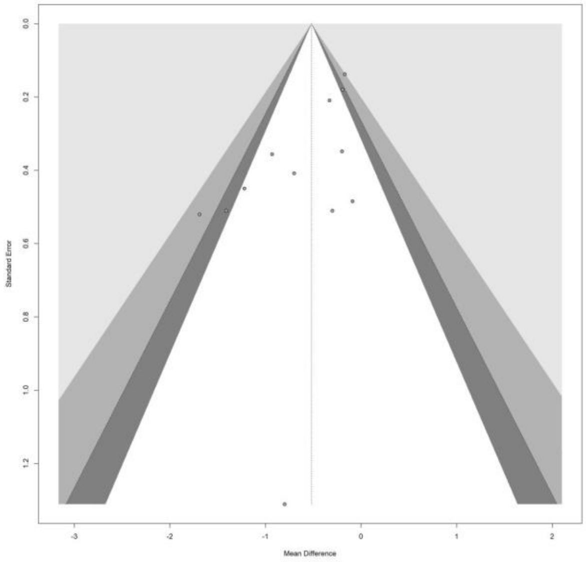

eFigure 178: Funnel plots for BMI (TRE vs control)

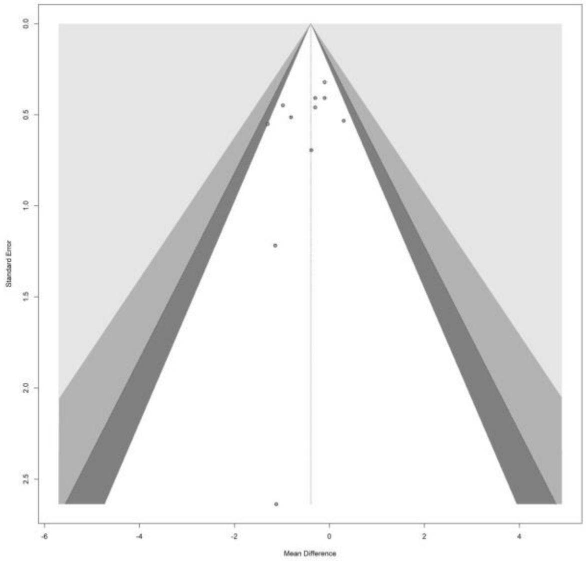

eFigure 179: Funnel plots for lean mass (TRE vs control)

© 2024 Liu HY et al. *JAMA Network Open.*

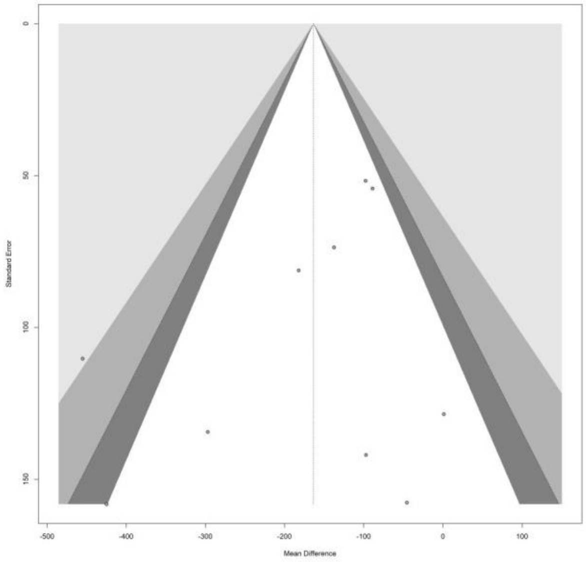

eFigure 180: Funnel plots for energy intake (TRE vs control)

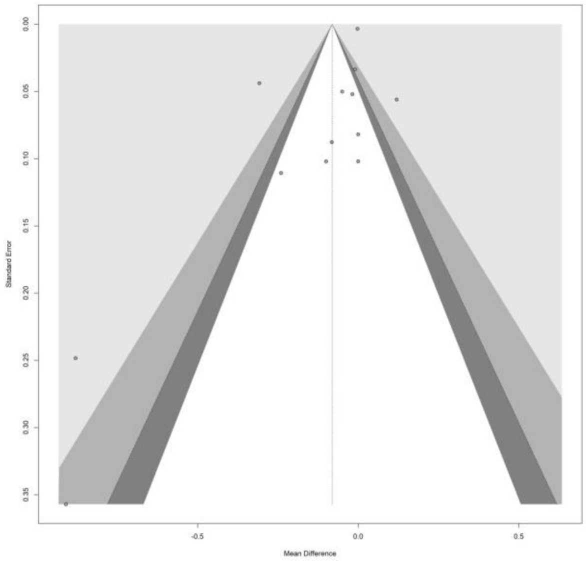

eFigure 181: Funnel plots for HbA1c (TRE vs control)

© 2024 Liu HY et al. *JAMA Network Open.*

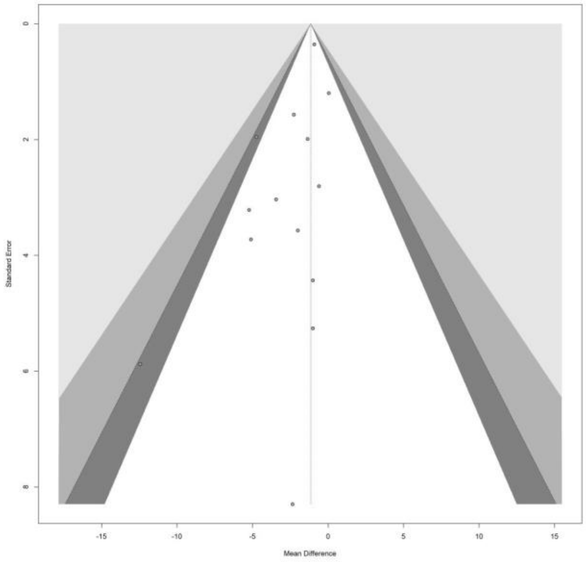

eFigure 182: Funnel plots for fasting glucose (TRE vs control)

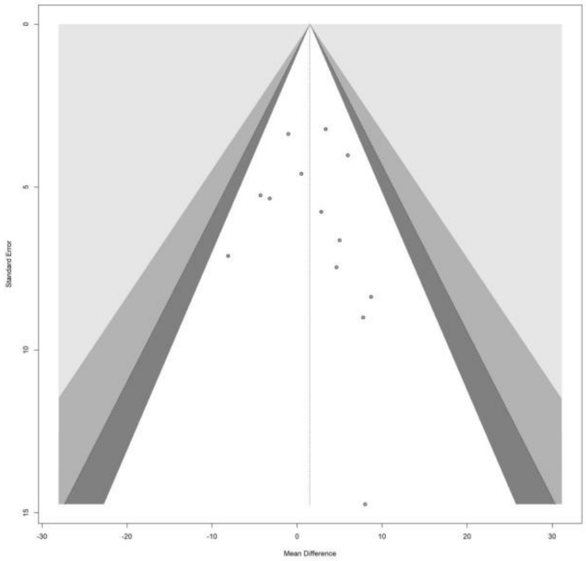

eFigure 183: Funnel plots for LDL (TRE vs control)

© 2024 Liu HY et al. *JAMA Network Open.*

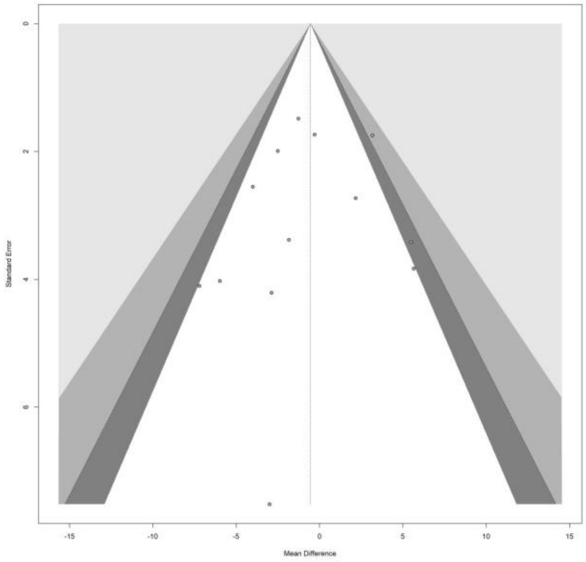

eFigure 184: Funnel plots for systolic blood pressure (TRE vs control)

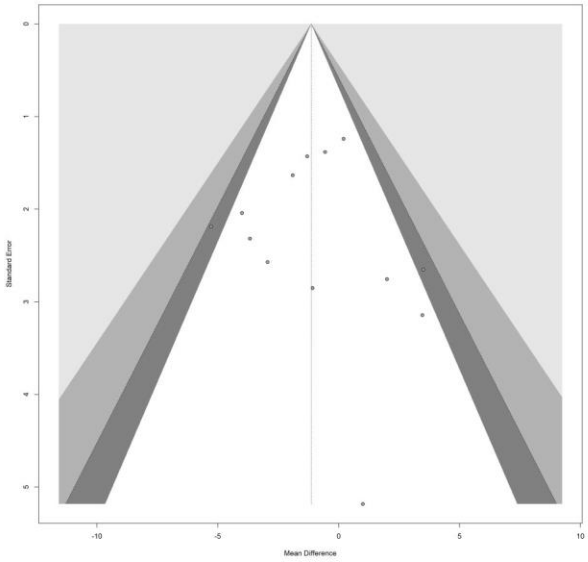

eFigure 185: Funnel plots for diastolic blood pressure (TRE vs control)

© 2024 Liu HY et al. *JAMA Network Open.*
