# Supplementary material for: Meal Timing and Anthropometric and Metabolic Outcomes: A Systematic Review and Meta-Analysis
Source: JAMA Netw Open. 2024 Nov 1;7(11):e2442163. doi: 10.1001/jamanetworkopen.2024.42163 (PMC11530941; doi:10.1001/jamanetworkopen.2024.42163)
Supplement: Supplement 2. — Data Sharing Statement [file jamanetwopen-e2442163-s002.pdf]

Data Sharing Statement

Liu. Meal Timing and Anthropometric and Metabolic Outcomes. *JAMA Netw Open*. Published November 01, 2024. doi:10.1001/jamanetworkopen.2024.42163

Data
Data available: Yes
Data types: Data (not involving human participants)
How to access data: extracted data from included studies
When available: With publication

Supporting Documents
Document types: None

Additional Information
Who can access the data: upon reasonable request from the corresponding author
Types of analyses: upon reasonable request from the corresponding author
Mechanisms of data availability: upon reasonable request from the corresponding author
